# Supplementary material for: PeptideMiner—neuropeptide discovery across the animal kingdom
Source: Gigascience. 2025 Aug 12;14:giaf078. doi: 10.1093/gigascience/giaf078 (PMC12343078; doi:10.1093/gigascience/giaf078)
Supplement: giaf078_Supplemental_File [file giaf078_supplemental_file.pdf]

Supplementary Materials

PeptideMiner – Neuropeptide Discovery across the Animal Kingdom

Helen C. Mendel[1,a], Gene Hopping[1,a], Eivind A. B. Undheim[2], Johannes Zuegg[1], Richard J. Lewis[1], Briony E. Forbes[3], Quentin Kaas[1*], Markus Muttenthaler[1,4*]

1. Institute for Molecular Bioscience, The University of Queensland, Brisbane, Australia

2. Centre for Ecological and Evolutionary Synthesis, Department of Biosciences, The University of Oslo, Oslo, Norway.

3. Discipline of Medical Biochemistry, Flinders Health and Medical Research Institute, Flinders University, Adelaide, Australia.

4. Institute of Biological Chemistry, Faculty of Chemistry, University of Vienna, Vienna, Austria.

[a] Contributed equally to the manuscript.

[*] Corresponding authors: Markus Muttenthaler and Quentin Kaas

Email: m.muttenthaler@uq.edu.au, quentin.kaas@syngenta.com

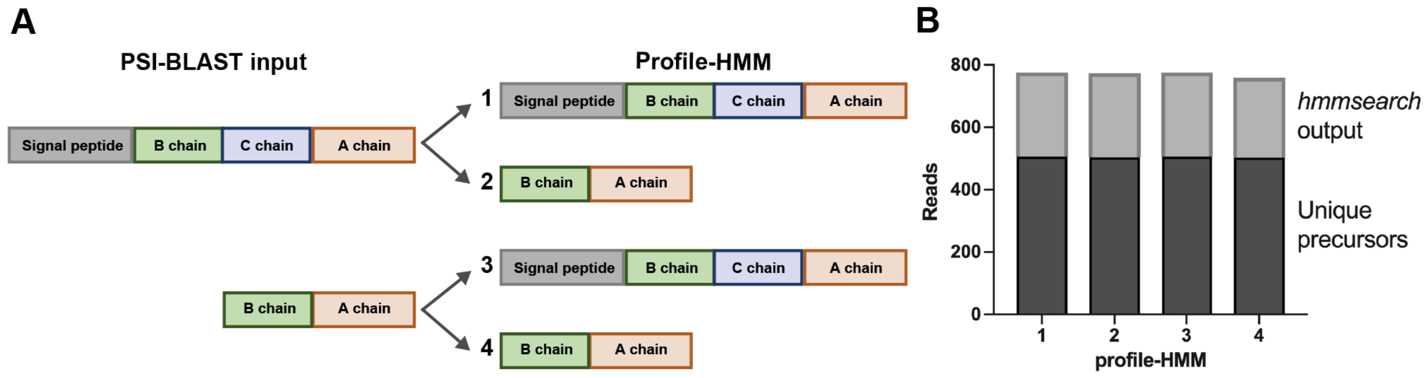

Figure S1. Profile-HMM construction and hmmsearch output. **A.** Schematic illustrating profile-HMM construction for human insulin. **B.** The number of reads and sequences returned for each profile-HMM before and after pipeline processing is similar across all four profile-HMMs.

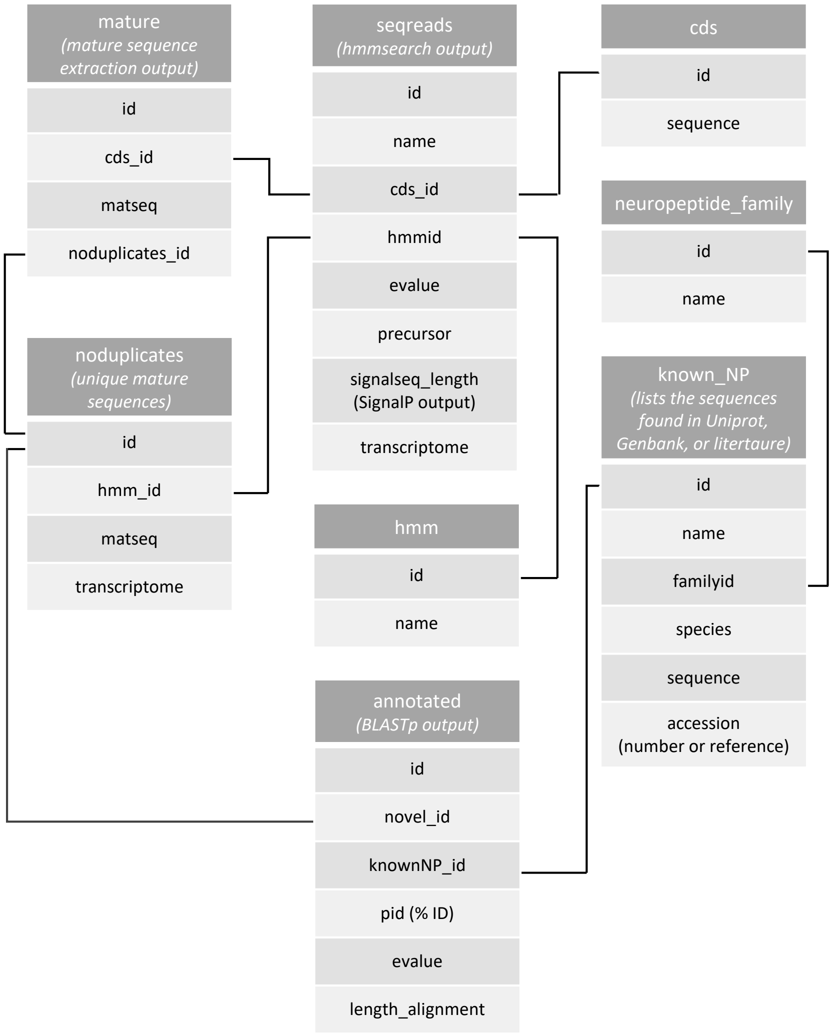

Figure S2. SQLite3 database schematic. Table structure and content of the relational SQLite3 database created to sort and manage the data generated by PeptideMiner. The SQLite database is created in the user-defined output directory using the PeptideMiner.sql file provided by PeptideMiner (in the /data subdirectory). The name of each table is highlighted by the dark shading, with a short description of the contents in italics underneath, where required. Lines between the tables indicate the relationship between the tables, more specifically, to the columns within each table.

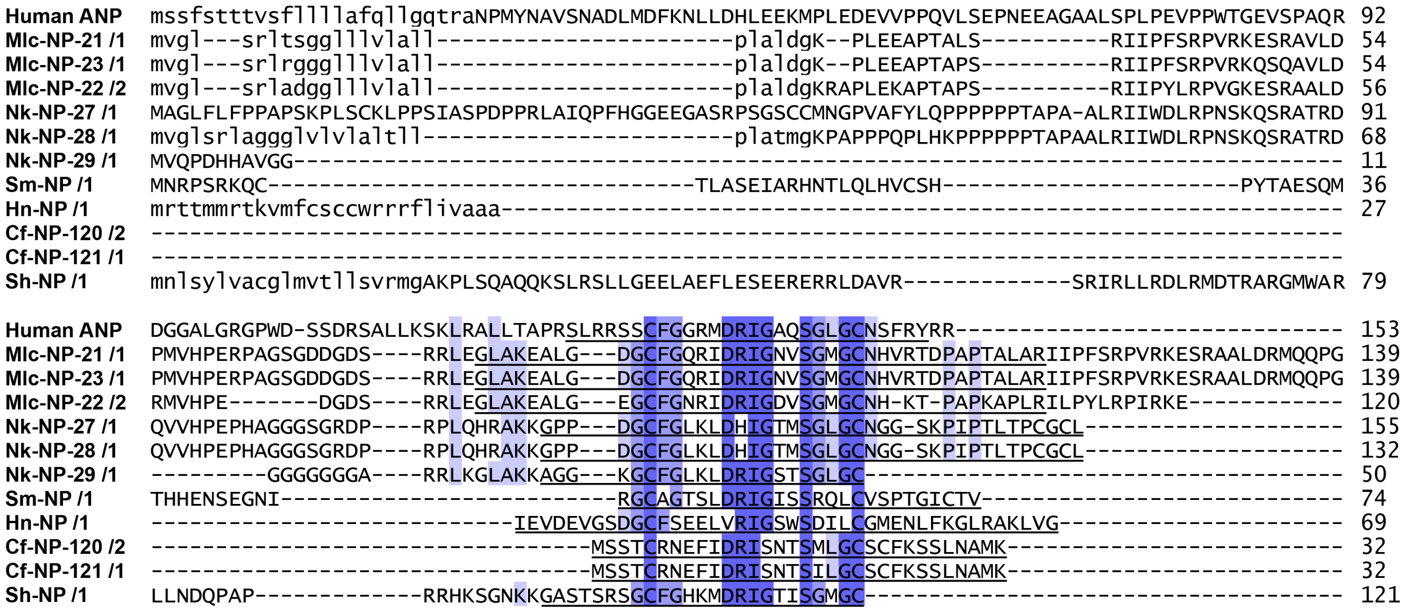

Figure S3. Multiple sequence alignment of natriuretic peptide precursors in venom gland transcriptomes aligned with human atrial natriuretic peptide (ANP). Predicted signal peptides (SignalP) are in lowercase. Mature peptides are underlined. Blue shading indicates the level of conservation for the mature natriuretic peptides, while darker shading indicates a higher level of conservation. Gaps are represented by '-'. The number of reads found for each precursor is listed next to the precursor name, following '/'.

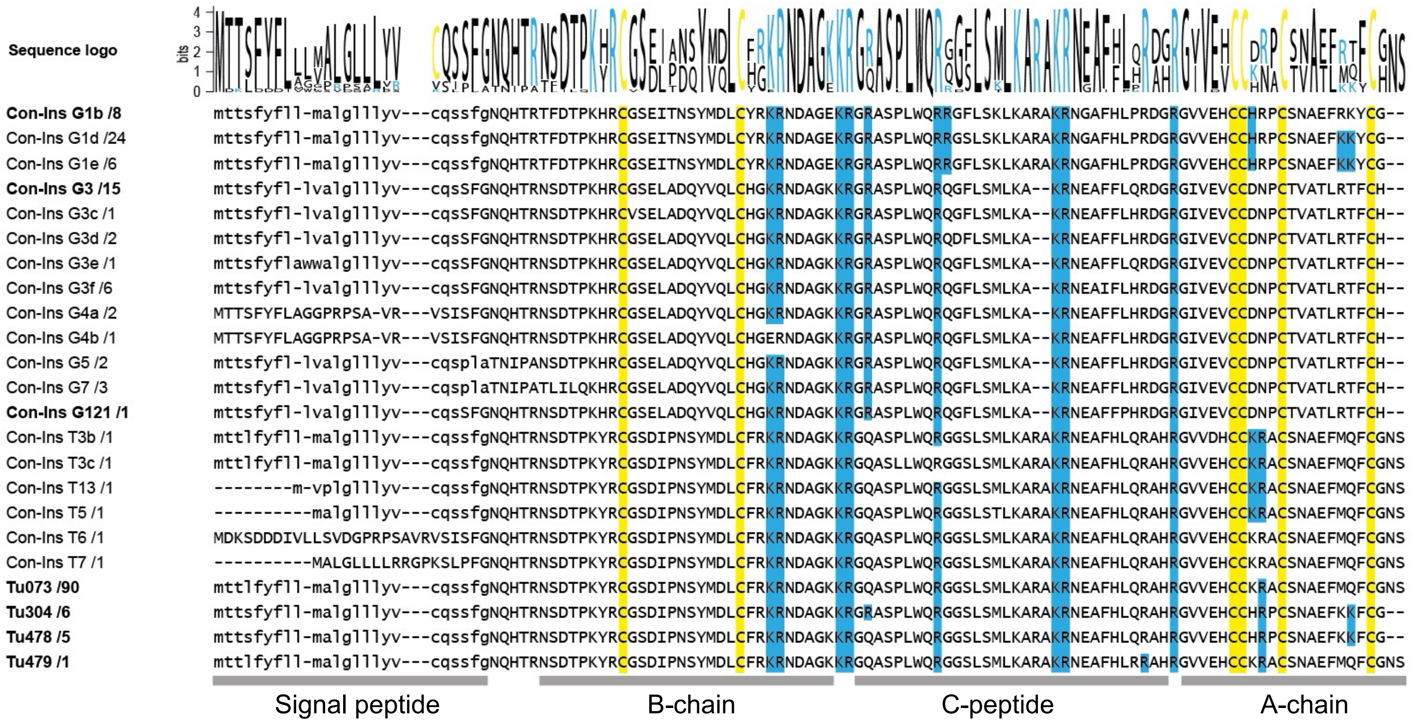

Figure S4. Alignment of the 23 vertebrate-like conoinsulin precursors identified in this study. Known conoinsulins are in bold. Sequence logo and approximate location of the insulin signal peptide, A- and B-chain and C peptide regions are depicted above and below the alignment, respectively. Alignment was generated using ClustalO, and the logo was generated using WebLogo. Cysteines are highlighted in yellow, and cleavage sites predicted by NeuroPred in blue. Gaps are represented by '-'. The number of reads found for each precursor is listed next to the precursor name, following '/'.

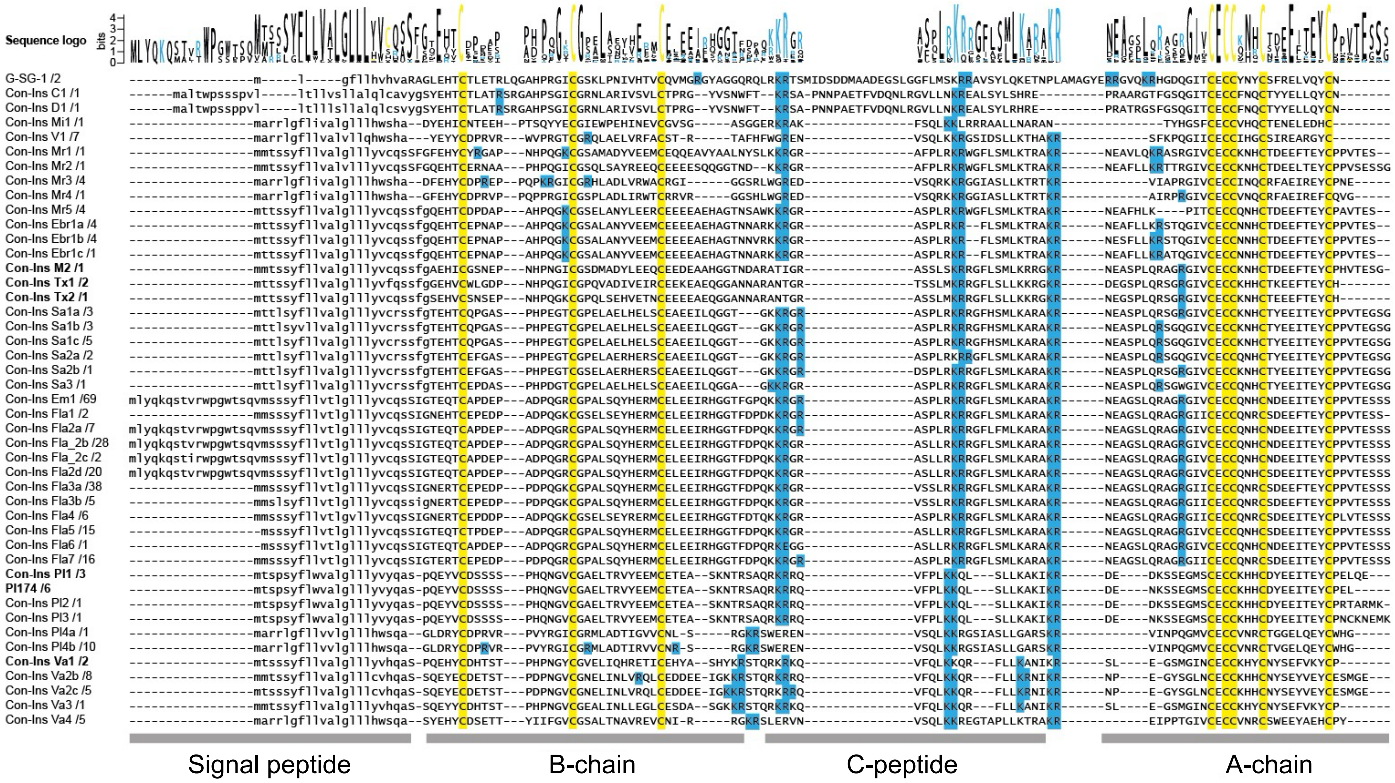

Figure S5. Alignment of the 45 MIP-like conoinsulin precursors identified in this study. Known conoinsulins are in bold. The sequence logo and approximate location of the insulin signal peptide, A- and B-chain, and C-peptide regions are depicted above and below the alignment, respectively. Alignment was generated using ClustalO, and the logo was generated using WebLogo. Cysteines are highlighted in yellow and cleavage sites predicted by NeuroPred in blue. Gaps are represented by '-'. The number of reads found for each precursor is listed next to the precursor name, following '/'.

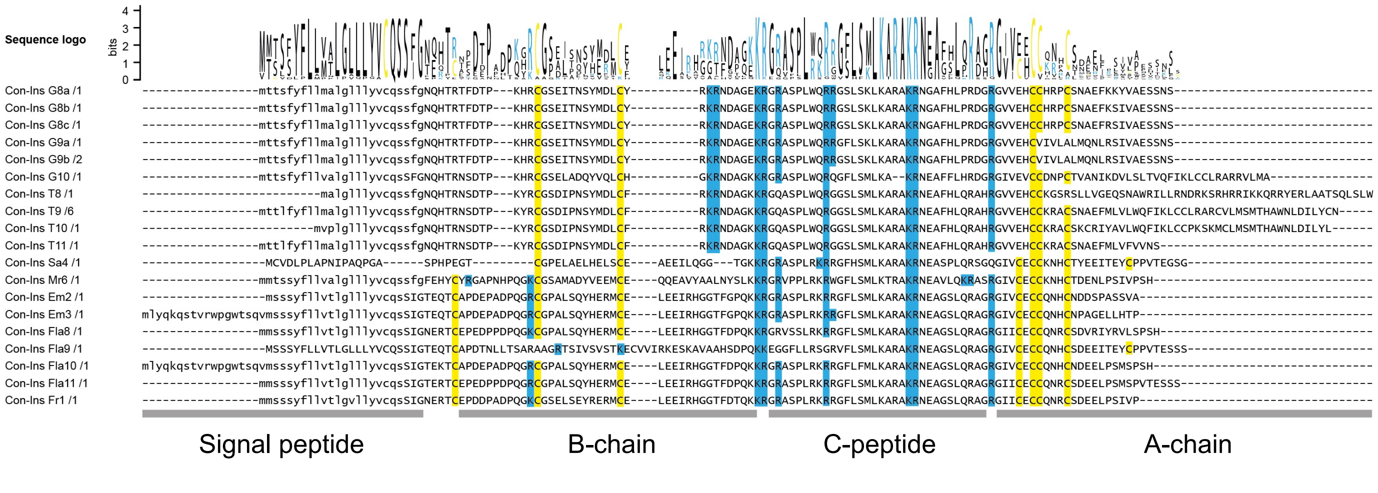

Figure S6. Alignment of the 19 conoinsulin precursors identified in this study that do not fit in with vertebrate-like, or MIP-like conoinsulin precursors. Sequence logo and approximate location of the insulin signal peptide, A- and B-chain and C-peptide regions are depicted above and below the alignment, respectively. Alignment was generated using ClustalO, and the logo was generated using WebLogo. Cysteines are highlighted in yellow and cleavage sites predicted by NeuroPred in blue. Gaps are represented by '-'. The number of reads found for each precursor is listed next to the precursor name, following '/'.

Table S1. 10-fold cross-validation for natriuretic peptide and insulin neuropeptide family.

	**Natriuretic peptide**	**Insulin**
Mature peptide E-value cut-off	0.001	0.1
Mature peptide max length	110	150
Average MCC [a]	0.99944	0.93599
Average TPR [b]	0.99888	0.99737
Average FPR [c]	0.00000	0.00013

[a] Matthews correlation coefficient; [b] True positive rate; [c] False positive rate.

Table S2. Performance comparison of PeptideMiner vs BLASTp using the natriuretic peptide and insulin neuropeptide family profile-HMMs.

	**MCC [a]**	**TPR [b]**	**FPR [c]**
**Natriuretic peptide**			
PeptideMiner	0.999	0.999	0.000000
BLASTp Precursor	0.985	0.976	0.000017
BLASTp Mature	0.992	0.984	0.000000
**Insulin**			
PeptideMiner	0.936	0.997	0.000130
BLASTp Precursor	0.763	0.587	0.000028
BLASTp A-chain	0.692	0.482	0.000003
BLASTp B-chain	0.659	0.438	0.000007

[a] Matthews correlation coefficient; [b] True positive rate; [c] False positive rate.

Table S3. Transcriptomes that were used in this study. The National Center for Biotechnology Information Sequence Read Archive (SRA) database reference number is provided. Previously unpublished transcriptomes are bolded.

**Phylum**	**Taxon**	**Species**	**Common name**	**SRA#**	**Tissue**
Annelida	Clitellata	*Hirudo nipponia*	Medicinal leech	SRR6371134	Salivary gland
Annelida	polycheata	*Glycera tridactyla*	Bloodworm	SRR5167051	Venom gland
Arthropoda	Arachnid – Scorpion	*Megacormus gertschi*	Scorpion	SRR3657526	Venom gland
Arthropoda	Arachnid – Scorpion	*Mesobuthus martensii*	Scorpion	SRR4188636	Venom gland
Arthropoda	Arachnid – Scorpion	*Superstitionia donensis*	Scorpion	SRR4381683	Venom gland
Arthropoda	Insecta – Diptera	*Machimus arthriticus*	Breck robberfly	SRR5185498	Thoracic glands
Arthropoda	Insecta – Hymenoptera	*Apis cerana*	Eastern honeybee	SRR1406762	Venom gland
Arthropoda	Insecta – Hymenoptera	*Microplitis demolitor*	Native wasp	SRR955397	Venom gland
Arthropoda	Insecta – Hymenoptera	*Nasonia giraulti*	Wasp	SRR4419178	Venom apparatus (venom gland and reservoir)
Arthropoda	Insecta – Hymenoptera	*Tetramorium bicarinatum*	Ant	SRR1106145	Venom gland
Arthropoda	Chilopod – Centipede	*Craterostigmus tasmanianus*	Tasmanian remarkable centipede	SRR8998267	Venom gland
Arthropoda	Chilopod – Centipede	*Lithobius forficatus*	Brown centipede	SRR28607002	Venom gland
Arthropoda	Chilopod – Centipede	*Lithobius forficatus*	Brown centipede	SRR8998265	Legs
Arthropoda	Chilopod – Centipede	*Scolopendra morsitans*	Tanzanian blue ringleg	SRR8998268	Venom gland
Arthropoda	Chilopod – Centipede	*Scolopendra subspinipes*	Vietnamese centipede	SRR6377864	Venom gland and muscle
Arthropoda	Chilopod – Centipede	*Scutigera coleoptrata*	House centipede	SRR8998264	Venom gland
Chordata	Actinopterygii	*Pterois volitans*	Red lionfish	SRR5141018	Dorsal spines
Chordata	Actinopterygii	*Synanceia horrida*	Estuarine stonefish	**SRR33038228**	Venom gland

Chordata	Actinopterygii – Bagridae	*Tachysurus fulvidraco*	Yellowhead catfish	SRR1987144	Venom gland
Chordata	Chondrichthyes – Dasyatidae	*Neotrygon kuhlii*	Bluespotted stingray	SRR1182699	Venom gland
Chordata	Chondrichthyes – Potamotrygonidae	*Potamotrygon amandae Potamotrygon falkneri*	Amanda's river stingray and Large-spot river stingray	SRR2039259	Venom gland
Chordata	Monotremes	*Tachyglossus aculeatus*	Echidna	SRR931704	Venom gland
Chordata	Reptilia – Serpentes	*Dispholidus typus*	Boomslang	SRR4379994	Venom gland
Chordata	Reptilia – Serpentes	*Micrurus lemniscatus carvalhoi*	South American Coral snake	DRR089665	Venom gland
Chordata	Reptilia – Serpentes	*Naja kaouthia*	Monocled cobra	SRR2917658	Venom gland
Cnidaria	Anthozoa	*Actinia tenebrosa*	Waratah anemone	SRR6282389	Tentacles
Cnidaria	Anthozoa	*Macrodactyla doreensis*	Corkscrew tentacle sea anemone	SRR14115222 SRR14115223 SRR14115224 SRR14115225	Tentacles
Cnidaria	Anthozoa	*Stichodactyla haddoni*	Haddon's sea anemone	SRR5397293	Tentacles
Cnidaria	Cubozoa	*Chironex fleckeri*	Sea wasp (box jellyfish)	SRR1819888	Tentacles
Mollusca	Gastropoda	*Conus catus*	Cat cone	**SRR32928198**	Venom duct
Mollusca	Gastropoda	*Conus catus*	Cat cone	**SRR32928197**	Nerve ring
Mollusca	Gastropoda	*Conus distans*	Distant cone	**SRR32928186**	Venom duct
Mollusca	Gastropoda	*Conus ebraeus*	Hebrew cone	**SRR32928182**	Venom duct
Mollusca	Gastropoda	*Conus emaciatus*	False virgin cone	**SRR32928181**	Venom duct
Mollusca	Gastropoda	*Conus flavidus*	Flavid pacific cone	**SRR32928180**	Venom duct
Mollusca	Gastropoda	*Conus frigidus*	Frigid cone	**SRR32928179**	Venom duct
Mollusca	Gastropoda	*Conus geographus*	Geographer cone	**SRR32928178**	Venom duct
Mollusca	Gastropoda	*Conus geographus*	Geographer cone	**SRR32928177**	Salivary gland
Mollusca	Gastropoda	*Conus imperialis*	Imperial cone	**SRR32928176 SRR32928196**	Venom duct
Mollusca	Gastropoda	*Conus marmoreus*	Marbled cone	**SRR32928195**	Venom duct
Mollusca	Gastropoda	*Conus miles*	Soldier cone	**SRR32928194**	Venom duct
Mollusca	Gastropoda	*Conus miliaris*	Thousand spot cone	**SRR32928193**	Venom duct
Mollusca	Gastropoda	*Conus planorbis*	Ringed cone	**SRR32928192**	Venom duct
Mollusca	Gastropoda	*Conus rattus*	Rat cone	**SRR32928191**	Venom duct
Mollusca	Gastropoda	*Conus sanguinolentus*	Blood-stained cone	**SRR32928190**	Venom duct
Mollusca	Gastropoda	*Conus textile*	Cloth of gold cone	**SRR32928189**	Venom duct
Mollusca	Gastropoda	*Conus textile*	Cloth of gold cone	**SRR32928188**	Nerve ring
Mollusca	Gastropoda	*Conus tulipa*	Tulip cone	**SRR32928185 SRR32928187**	Venom duct
Mollusca	Gastropoda	*Conus varius*	Freckled cone	**SRR32928184**	Venom duct
Mollusca	Gastropoda	*Conus vexillum*	Flag cone	**SRR32928183**	Venom duct
Mollusca	Mollusca – Cephalopoda	*Hapalochlaena maculosa*	Southern blue-ringed octopus	SRR3105558	Posterior salivary glands
Mollusca	Mollusca – Cephalopoda	*Octopus bimaculoides*	California two-spot octopus	SRR2047107	Posterior salivary glands

Table S4. Novel mature conoinsulins discovered in this study.

Name	B-Chain	A-Chain
	**Vertebrate-like**	
G3c	NSDTPKHRCVSELADQYVQLCHG	GIVEVCCDNPCTVATLRTFCH
G4b	NSDTPKHRCGSELADQYVQLCHGERNDA	GIVEVCCDNPCTVATLRTFCH
G5	TNIPANSDTPKHRCGSELADQYVQLCHG	GIVEVCCDNPCTVATLRTFCH
G7	TNIPATLILQKHRCGSELADQYVQLCHG	GIVEVCCDNPCTVATLRTFCH
T3b	NSDTPKYRCGSDIPNSYMDLCFR	GVVDHCCKRACSNAEFMQFCGNS
	**MIP-like**	
C1	SYEHTCTLATRSRGAHPSGICGRNLARIVSVLCTPRGYVSNWFT	EALSYLSHREPRAARGTFGSQGITCECCFNQCTYYELLQYCN
D1	SYEHTCTLATRSRGAHPSGICGRNLARIVSVLCTPRGYVSNWFT	EALSYLRHREPRATRGSFGSQGITCECCFNQCTYYELLQYCN
Ebr3	QEHTCEPNAPAHPQGKCGSALANYVEEMCEEEEAEHAGTNNA	ATQGIVCECCNNHCTDEEFTEYCPPVTES
Em1	SIGTEQTCAPDEPADPQGRCGPALSQYHERMCELEEIRHGGTFGPQ	GIVCECCQNHCNDEEITEYCPPVTESSS
Fla1	NEHTCEPEDPADPQGKCGSELSEYHERMCELEEIRHGGTFDPD	GIICECCQNRCSDEEFTEYCPPVTES
Fla2a	SIGTEQTCAPDEPADPQGRCGPALSQYHERMCELEEIRHGGTFDPQ	GIVCECCQNHCNDEEITEYCPPVTESSS
Fla2d	SIGTEQTCAPDEPADPQGRCGPALSQYHERMCELEEIRHGGTFDPQ	GIVCECCQNHCSDEEITEYCPPVTESSS
Fla3a	SIGNERTCEPEDPPDPQGKCGPALSQYHERMCELEEIRHGGTFDPQ	GIICECCQNRCSDEEFTEYCPPVTESSS
Fla3b	NERTCEPEDPPDPQGKCGPALSQYHERMCELEEIRHGGTFDPQ	GIICECCQNRCSDEEFTEYCPPVTESSS
Fla4	SIGNERTCEPDDPADPQGKCGSELSEYRERMCELEEIRHGGTFDTQ	GIICECCQNRCSDEEITEYCPLVTESSS
Fla5	SIGTEQTCTPDEPADPQGRCGPALSQYHERMCELEEIRHGGTFDPQ	GIVCECCQNHCSDEEITEYCPPVTESSS
Fla7	SIGTERTCEPEDPPDPQGRCGPALSQYHERMCELEEIRHGGTFDPQ	GIICECCQNRCSDEEITEYCPPVTESSS
Mi1	DYEHICNTEEHPTSQYYECGIEWPEHINEVCGVSGASGGE	LRRRAALLNARANTYHGSFCECCVHQCTENELEDHC
Mr1	SFEHYCYRGAPNHPQGKCGSAMADYVEEMCEQQEAVYAALNYSL	ASRGIVCECCKNHCTDEEFTEYCPPVTES
Mr2	SFGQEHTCERNAAPHPQGICGSQLSAYREEQCEEEESQQGGTND	TTRGIVCECCKNHCTDEELTEYCPPVSESSG
Mr3	DFEHYCDPREPPQPKRGICGRHLADLVRWACRGIGGSRLWG	VIAPRGIVCECCINQCRFAEIREYCPNE
Mr4	GFEHYCDPRVPPQPPRGICGSPLADLIRWTCRRVRGGSHLWG	GIVCECCVNQCRFAEIREFCQVG
Mr5	QEHTCDPDAPAHPQGKCGSELANYLEERCEEEEAEHAGTNSAW	NEAFHLKPITCECCQNHCTDEEFTEYCPAVTES
Pl2	QEYVCDSSSSPHQNGVCGAELTRVYEEMCETEASKNTRSAQ	DEDKSSEGMSCECCKHHCDYEEITEYCPRTARMK
Pl3	QEYVCDSSSSPHQNGVCGAELTRVYEEMCETEASKNTRSAQ	DENKSSEGMSCECCKHHCDYEEITEYCPNCKNEMK
Pl4a	GLDRYCDPRVRPVYRGICGRMLADTIGVVCNLSRG	VINPQGMVCECCVNRCTGGELQEYCWHG
Pl4b	GLDRYCDPRVRPVYRGICGRMLADTIRVVCN	VINPQGMVCECCVNRCTVGELQEYCWHG
Sa1a	TEHTCQPGASPHPEGTCGPELAELHELSCEAEEILQGGTG	GIVCECCKNHCTYEEITEYCPPVTEGSG
Sa1b	TEHTCQPGASPHPEGTCGPELAELHELSCEAEEILQGGTG	SGQGIVCECCKNHCTYEEITEYCPPVTEGSG
Sa2a	TEHTCEFGASPHPEGTCGPELAERHERSCEAEEILQGGTG	SGQGIVCECCQNHCTYEEITEYCPPVTEGSG
Sa2b	TEHTCEFGASPHPEGTCGSELAERHERSCEAEEILQGGTG	GIVCECCKNHCTYEEITEYCPPVTEGSG
Sa3	TEHTCEPDASPHPDGTCGPELAELHELSCEAEEILQGGAG	SGWGIVCECCQNHCTYEEITEYCPPVTEGSG
V1	YEYYCDPRVRWVPRGTCGRQLAELVRFACSTRTAFHFWG	SFKPQGIICECCIHGCSIREARGYC
Va3	SSQEYYCDHTSTPHPNGVCGEALINLLEGLCESDASG	SLEGSMGINCECCKHYCNYSEFVKYCP
Va4	SYEHYCDSETTYIIFGVCGSALTNAVREVCNIRRG	EIPPTGIVCECCVNRCSWEEYAEHCPY
G-SG-1	RAGLEHTCTLETRLQGAHPRGICGSKLPNIVHTVCQVMG	HGDQGITCECCYNYCSFRELVQYCN
	**Other**	
Em2	SIGTEQTCAPDEPADPQGRCGPALSQYHERMCELEEIRHGGTFGPQ	GIVCECCQNHCNDDSPASSVA
Em3	SIGTEQTCAPDEPADPQGRCGPALSQYHERMCELEEIRHGGTFGPQ	GIVCECCQNHCNPAGELLHTP
Fla10	SIGTEKTCAPDEPADPQGRCGPALSQYHERMCELEEIRHGGTFDPQ	GIVCECCQNHCNDEELPSMSPSH
Fla11	SIGTERTCEPEDPPDPQGRCGPALSQYHERMCELEEIRHGGTFDPQ	GIICECCQNRCSDEELPSMSPVTESSS
Fla8	SIGNERTCEPEDPPDPQGKCGPALSQYHERMCELEEIRHGGTFDPQ	GIICECCQNRCSDVRIYRVLSPSH
Fla9	SIGTEQTCAPDTNLLTSARAAG	GIVCECCQNHCSDEEITEYCPPVTESSS
Fr1	SIGNERTCEPDDPADPQGKCGSELSEYRERMCELEEIRHGGTFDTQ	GIICECCQNRCSDEELPSIVP
G10	SFGNQHTRNSDTPKHRCGSELADQYVQLCHG	GIVEVCCDNPCTVANIKDVLSLTVQFIKLCCLRARRVLMA
G8a	NQHTRTFDTPKHRCGSEITNSYMDLCY	GVVEHCCHRPCSNAEFKKYVAESSN
G8b	NQHTRTFDTPKHRCGSEITNSYMDLCY	GVVEHCCHRPCSNAEFKSIVAESSNS
G8c	NQHTRTFDTPKHRCGSEITNSYMDLCY	GVVEHCCHRPCSNAEFRSIVAESSNS
G9a	NQHTRTFDTPKHRCGSEITNSYMDLCY	GVVEHCVIVLALMQNLRSIVAESSNS
G9b	NQHTRTFDTPKHRCGSEITNSYMDLCY	GVVEHCVIVLALMQNLRSIVAESSN
Mr6	FEHYCYRGAPNHPQGKCGSAMADYVEEMCEQQEAVYAALNYSL	ASRGIVCECCKNHCTDENLPSIVPSH
Sa4	MCVDLPLAPNIPAQPGASPHPEGTCGPELAELHELSCEAEEILQGGTG	SGQGIVCECCKNHCTYEEITEYCPPVTEGSG
T10	NQHTRNSDTPKYRCGSDIPNSYMDLCF	NEAFHLQRAHRGVVEHCCKRACSKCRIYAVLWQFIKLCCPKSKMCLMSMTHAWNLDILYL
T11	NQHTRNSDTPKYRCGSDIPNSYMDLCF	NEAFHLQRAHRGVVEHCCKRACSNAEFMLVFVVNS
T8	NQHTRNSDTPKYRCGSDIPNSYMDLCF	NEAFHLQRAHRGVVEHCCKGS
T9	NQHTRNSDTPKYRCGSDIPNSYMDLCF	GVVEHCCKRACSNAEFMLVLWQFIKLCCLRARCVLMSMTHAWNLDILYCN
