# Supplementary material for: PeptideMiner—neuropeptide discovery across the animal kingdom
Source: Gigascience. 2025 Aug 12;14:giaf078. doi: 10.1093/gigascience/giaf078 (PMC12343078; doi:10.1093/gigascience/giaf078)
Supplement: giaf078_GIGA-D-24-00521_Revision_1 [file giaf078_giga-d-24-00521_revision_1.pdf]

GigaScience

PeptideMiner – Neuropeptide Discovery across the Animal Kingdom

--Manuscript Draft--

**Manuscript Number:**	GIGA-D-24-00521R1
**Full Title:**	PeptideMiner – Neuropeptide Discovery across the Animal Kingdom
**Article Type:**	Research
**Funding Information:**	H2020 European Research Council (714366) — Prof Markus Muttenthaler<br>Australian Research Council (DE150100784) — Prof Markus Muttenthaler<br>Australian Research Council (DP190101667) — Prof Markus Muttenthaler<br>Australian Research Council (FT210100266) — Prof Markus Muttenthaler<br>H2020 European Research Council (101039862) — Assoc Prof Eivind A. B. Undheim<br>Norwegian Research Council (287462) — Assoc Prof Eivind A. B. Undheim<br>National Health and Medical Research Council (2037680) — Prof Markus Muttenthaler
**Abstract:**	Neuropeptides represent the largest and most diverse class of cell-to-cell signaling molecules, holding important roles in animal physiology and behavior. They are evolutionarily ancient and widely distributed across the animal kingdom. Although over 200 neuropeptides have been identified, only a small fraction has been functionally characterized. A recognized bottleneck is the lack of effective tools to study their biological roles and therapeutic potential. Interestingly, neuropeptide-like peptides are also found in animal venoms, where they contribute to prey capture or defensive strategies. Mapping neuropeptide families across the animal kingdom is challenging due to their high sequence divergence and short mature peptide sequences. To address this, we developed PeptideMiner, a search tool that employs profile-hidden Markov models (profile-HMM) for family-specific peptide discovery. PeptideMiner was systematically validated and benchmarked against existing methods, demonstrating its superior performance. By applying PeptideMiner to several venom transcriptomes–including 24 previously unpublished datasets–we identified 10 novel natriuretic peptides from distantly related species and 57 novel insulin-like sequences from marine predatory cone snails. Chemical synthesis and structure-activity relationship studies of newly identified conoinsulins at human insulin receptors emphasized the value of our approach in elucidating ligand-receptor interactions and discovering new pharmacological probes and therapeutic leads. PeptideMiner offers a powerful platform for discovering new bioactive peptides and family-specific analogs, accelerating both natural product discovery and evolutionary research.
**Corresponding Author:**	Markus Muttenthaler<br>University of Vienna: Universitat Wien<br>Vienna, AUSTRIA
**Corresponding Author Secondary Information:**	
**Corresponding Author's Institution:**	University of Vienna: Universitat Wien
**Corresponding Author's Secondary Institution:**	
**First Author:**	Helen C. Mendel, PhD
**First Author Secondary Information:**	
**Order of Authors:**	Helen C. Mendel, PhD
	Gene Hopping, PhD

	Eivind A. B. Undheim, PhD
	Johannes Zuegg
	Richard J. Lewis, PhD
	Briony E. Forbes, PhD
	Quentin Kaas, PhD
	Markus Muttenthaler, PhD
**Order of Authors Secondary Information:**	
**Response to Reviewers:**	Please see the attached Response/Cover Letter.
**Additional Information:**	
**Question**	**Response**
Are you submitting this manuscript to a special series or article collection?	No
**Experimental design and statistics**<br><br>Full details of the experimental design and statistical methods used should be given in the Methods section, as detailed in our Minimum Standards Reporting Checklist. Information essential to interpreting the data presented should be made available in the figure legends.<br><br>Have you included all the information requested in your manuscript?	Yes
**Resources**<br><br>A description of all resources used, including antibodies, cell lines, animals and software tools, with enough information to allow them to be uniquely identified, should be included in the Methods section. Authors are strongly encouraged to cite Research Resource Identifiers (RRIDs) for antibodies, model organisms and tools, where possible.<br><br>Have you included the information requested as detailed in our Minimum Standards Reporting Checklist?	Yes
**Availability of data and materials**	Yes

Powered by Editorial Manager® and ProduXion Manager® from Aries Systems Corporation

All datasets and code on which the conclusions of the paper rely must be either included in your submission or deposited in publicly available repositories (where available and ethically appropriate), referencing such data using a unique identifier in the references and in the “Availability of Data and Materials” section of your manuscript.<br><br>Have you have met the above requirement as detailed in our Minimum Standards Reporting Checklist?	
GigaScience has policies and guidelines in place for the use of generative AI-writing tools such as ChatGPT. If you have used such writing tools to assist with writing the manuscript this must be declared and cited in the text. Authors should not list AI-writing tools and other AI-assisted technologies as an author or co-author and should acknowledge that they are fully responsible for text generated or refined by AI-writing tools.<p><br><br>A summary of use (particularly in the introduction or among methods) needs to be included at the end of the paper, and the outputs should also be included as a supplementary file hosted in GigaDB or other open repositories. Please read our guidelines for more information. <p><br><br>By submitting to GigaScience, you are aware of the journal's AI-writing tools policy, and if you have declared use of such tools below, you have acknowledged this where appropriate in your manuscript and have made a summary of use and outputs available. <p><br><b>AI-assisted writing tools have been used in the preparation of this manuscript?	No

Powered by Editorial Manager® and ProduXion Manager® from Aries Systems Corporation

Manuscript

Click here to access/download;Manuscript;Manuscript including figures_revised.pdf

PeptideMiner – Neuropeptide Discovery across the Animal Kingdom

Helen C. Mendel[1,a], Gene Hopping[1,a], Eivind A. B. Undheim[2], Johannes Zuegg[1], Richard J. Lewis[1], Briony E. Forbes[3], Quentin Kaas[1*], Markus Muttenthaler[1,4*]

[1.] Institute for Molecular Bioscience, The University of Queensland, Brisbane, Australia

[2.] Centre for Ecological and Evolutionary Synthesis, Department of Biosciences, The University of Oslo, Oslo, Norway.

[3.] Discipline of Medical Biochemistry, Flinders Health and Medical Research Institute, Flinders University, Adelaide, Australia.

[4.] Institute of Biological Chemistry, Faculty of Chemistry, University of Vienna, Vienna, Austria.

[a] Contributed equally to the manuscript.

[*] Corresponding authors: Markus Muttenthaler and Quentin Kaas

Email: m.muttenthaler@uq.edu.au, quentin.kaas@syngenta.com

Author Contributions: H.C.M., Q.K., and M.M. designed the research project. H.C.M., G.H., B.E.F., E.A.B.U., J.Z., and Q.K. performed experiments. H.C.M., Q.K., and M.M. analyzed the data. H.C.M., G.H., and M.M. wrote the paper.

Competing Interest Statement: The authors declare no competing financial interest.

Classification: Biological Sciences; Biophysics and Computational Biology

Keywords: Neuropeptide; venom; transcriptomics; natriuretic peptide; insulin.

Abstract

Neuropeptides represent the largest and most diverse class of cell-to-cell signaling molecules, holding important roles in animal physiology and behavior. They are evolutionarily ancient and widely distributed across the animal kingdom. Although over 200 neuropeptides have been identified, only a small fraction has been functionally characterized. A recognized bottleneck is the lack of effective tools to study their biological roles and therapeutic potential. Interestingly, neuropeptide-like peptides are also found in animal venoms, where they contribute to prey capture or defensive strategies. Mapping neuropeptide families across the animal kingdom is challenging due to their high sequence divergence and short mature peptide sequences. To address this, we developed PeptideMiner, a search tool that employs profile-hidden Markov models (profile-HMM) for family-specific peptide discovery. PeptideMiner was systematically validated and benchmarked against existing methods, demonstrating its superior performance. By applying PeptideMiner to several venom transcriptomes–including 24 previously unpublished datasets–we identified 10 novel natriuretic peptides from distantly related species and 57 novel insulin-like sequences from marine predatory cone snails. Chemical synthesis and structure-activity relationship studies of newly identified conoinsulins at human insulin receptors emphasized the value of our approach in elucidating ligand-receptor interactions and discovering new pharmacological probes and therapeutic leads. PeptideMiner offers a powerful platform for discovering new bioactive peptides and family-specific analogs, accelerating both natural product discovery and evolutionary research.

Introduction

Animal venoms represent an invaluable natural source for developing pharmacological probes and therapeutic agents [1-3]. Comprising intricate and diverse mixtures of bioactive peptides, venoms have evolved to serve the dual purpose of defense and prey immobilization. While venom peptides are primarily recognized for their ability to target ion channels to cause pain or paralysis in prey, they also exhibit efficacy against other target classes, including G protein-coupled receptors (GPCRs) [4], transporters [5], and soluble protein targets such as thrombin [6] and fibrinogen [7]. By identifying, isolating, synthesizing, and pharmacologically characterizing these structurally well-defined venom peptides, researchers have unveiled highly potent and selective molecules that have revolutionized ion channel and pain research [8-10]. Notably, this approach led to the FDA approval of ziconotide (Prialt), a venom peptide derived from the marine predatory cone snail of the genus *Conus*, as a peptide drug for managing severe chronic pain [11]. Another significant example was the discovery of exenatide, a venom peptide derived from the saliva of the Gila monster, which was approved as a peptide drug for treating type II diabetes (Byetta) [12], among others [3].

In addition to the well-studied venom peptides targeting ion channels, there is growing evidence of the presence of neuropeptides in animal venoms, albeit with limited characterization and unclear roles in envenomation. It is hypothesized that neuropeptide signaling systems and their corresponding membrane receptors could represent attractive targets for envenomation due to their crucial physiological roles and remarkable conservation across prey and predators. Notable examples of such venom peptides encompass conopressins (vasopressin, oxytocin) [13], contulakins (neurotensin) [14], conoinsulins (insulin) [15], conorfamides (RF-amide) [16], and natriuretic peptides [17].

Neuropeptides serve as signaling molecules secreted by neurons, orchestrating a wide array of functions encompassing fluid homeostasis, reproduction, appetite control, memory, learning, and complex social behavior [18, 19]. These ancient signaling systems are widely distributed throughout the animal kingdom, with at least 30 neuropeptide signaling systems traceable to a common bilaterian ancestor [20]. Despite their vital physiological roles, our understanding of these signaling systems in animals and humans remains limited, primarily due to a scarcity of pharmacological probes beyond the often nonspecific endogenous ligands required for dissecting the complex pathways and receptor subtypes.

Venoms, containing many diverse neuropeptide-like peptides with unique pharmacological profiles, hold immense promise for transforming neuropeptide research and facilitating the discovery of therapeutic leads for human diseases [19]. The systematic discovery of neuropeptide-like venom peptides is, however, challenging due to a lack of methodologies capable of reliably identifying neuropeptide families across evolutionarily

distant species.

Traditionally, venom research relied on venom collection, bioactivity-guided fractionation, and mass spectrometry. However, recent advances in proteomics, bioinformatics, and nucleotide sequencing [21] have reshaped the approach to studying venoms, culminating in an approach termed integrated venomics [22-25]. Integrated venomics involves generating venom gland transcriptomes that encompass all venom peptide sequences, including their precursors, and subsequently using bioinformatics to match these sequences against the crude venom proteome as well as annotated peptides and proteins in databases such as UniProt and NCBI. Currently, this matching process relies heavily on the NCBI protein-protein Basic Local Alignment Search Tool (BLASTp), which performs well for large homologous sequences but struggles with the short length and high precursor sequence diversity across divergent species characteristic of neuropeptides [26, 27]. Alternative probabilistic models exist, which parameterize complex position-specific models and are expected to be more effective in detecting distant homology [28-30]. One such model is the profile-hidden Markov model (profile-HMM), a probabilistic model of multiple sequence alignments represented as a series of amino acid-emitting *states,* with probabilistic connections between states that account for fully conserved segments, insertions, and deletions [31, 32].

A bioinformatic pipeline or search algorithm capable of reliably identifying homologous neuropeptides across a wide range of species would be of high value, facilitating the systematic mapping and characterization of neuropeptide-like venom peptides. Such an advancement would enhance our understanding of the evolutionary significance and distribution of neuropeptide-like venom peptides, ultimately accelerating the discovery of new pharmacological tools and therapeutic agents.

In this study, we present PeptideMiner, a neuropeptide discovery pipeline that utilizes neuropeptide family-specific profile-HMMs to efficiently identify neuropeptide sequences across different databases and sources. To highlight the application scope of PeptideMiner, we used it to identify new natriuretic and insulin-like peptides from venom gland transcriptomes of a broad range of venomous species, including 24 previously unpublished transcriptomes, and benchmarked it against existing methods of sequence homology searches. Finally, we chemically synthesized and tested newly identified insulin-like venom peptides against the human insulin receptor to demonstrate the translational potential of this new computational discovery pipeline.

Results

PeptideMiner Overview

PeptideMiner is a versatile new tool for neuropeptide discovery that integrates a suite of bioinformatic tools, including HMMER3 [33], SignalP [34], FASTA36 [35], BLAST [36], and SQLite3 [37] to search, filter, and annotate amino acid sequences. It is implemented in Python, a high-level programming language commonly used for bioinformatic pipelines due to its rapid implementation and seamless integration of multiple bioinformatic tools [38, 39]. By employing peptide/protein family-specific profile-HMMs, PeptideMiner is able to efficiently search translated transcriptome or genome sequences for a diverse range of amino acid sequences, including short and diverse (neuro)peptides as well as longer proteins.

Generation of precursor and mature profile-hidden Markov models

Neuropeptide sequences of interest were submitted as a query to the PSI-BLAST (Position-Specific Iterative Basic Local Alignment Tool) against the NCBI non-redundant database [36]. The hits from the first iteration generated a position-specific scoring matrix that was used to search the database for sequences matching the conservation pattern specified by the matrix. The process was iterated 10 times or until no more new sequences were detected. This preliminary training set of sequences was aligned using MUSCLE [40] or ClustalO [41] and trimmed in Jalview [42]. Each initial training set was trimmed to generate two training sets: in one, the sequences were trimmed to the whole precursor peptide, and in the other, to the mature peptide. Each training set was used to build a *precursor*-profile-HMM and a *mature*-profile-HMM using *hmmbuild* from the HMMER 3.0 package [43]. A similar number of reads were observed irrespective of whether the *precursor*-profile- or *mature*-profile-HMM was used, indicating the profile-HMM did not affect the efficiency or depth of the search (Figure S1). For this work, the products of both *precursor*- and *mature*-profile-HMMs were combined, and duplicate product sequences were removed.

PeptideMiner Workflow

To initiate the PeptideMiner workflow (Figure 1), users must first input one or more profile-HMMs of neuropeptide families of interest, as well as the database of amino acid sequences that should be searched as FASTA files. *Hmmsearch* from the HMMER3 package is then used to search the database using the profile-HMMs. *Hmmsearch* results are stored in an SQLite database (Figure S2). The protein-coding sequences (CDS) of the hypothetical neuropeptide precursors are predicted by extracting the sequence between the methionine 'M' start codon and the stop codon or, if there is no stop codon, the end of the contig. SignalP [34] is then used to identify the signal peptides of the predicted CDS and, if present, excised from the precursor sequence to

facilitate mature peptide identification. FASTA36 [35] is used to align all the processed CDS to a manually curated list of known mature peptides, taken from the UniProt database, of the neuropeptide family of interest, followed by a precursor cleavage site prediction using the algorithm from the ConoServer annotation pipeline [44]. The predicted mature peptides for all sequences from the same transcriptome are then compiled, and duplicate mature peptides are removed. BLASTp is then used to annotate the predicted mature peptides by homology using a list of known neuropeptide amino acid sequences.

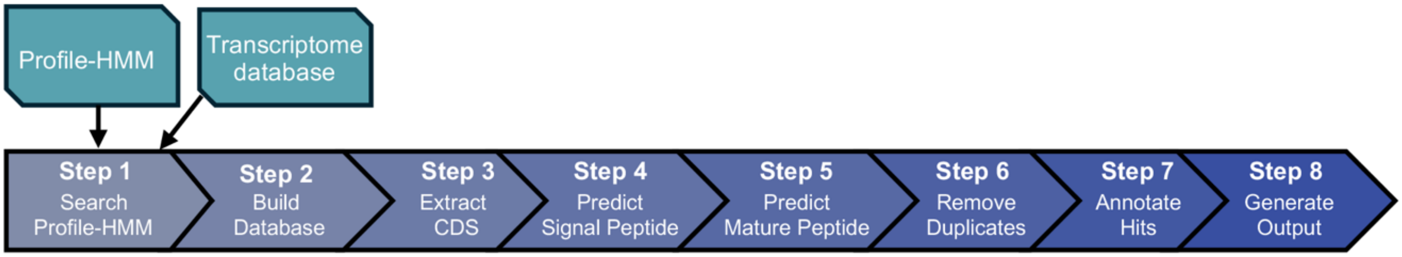

Figure 1. PeptideMiner pipeline. PeptideMiner uses *hmmsearch* in the HMMER 3 package to search through the transcriptomes with the profile-HMMs and saves the output to an SQLite database. The protein-coding sequences (CDS) are extracted from the hits and submitted to SignalP to determine whether a signal peptide is present. Next, mature peptides are predicted and compared to a list of known sequences belonging to the neuropeptide family of interest. The final output consists of a list of precursors, the transcriptomes, predicted mature peptides, and known sequences they are most similar to. The user can specify the minimum CDS length required, the signal peptide cut-off, the minimum length of the signal peptide, the FASTA36 E-value cut-off, and the minimum and maximum length of the mature peptide.

PeptideMiner Performance Evaluation

To assess the pipeline performance, a 10-fold cross-validation analysis was conducted using a test database consisting of 300,000 randomly selected sequences from various eukaryotic species acquired from the UniProtKB database [45]. This database was chosen to provide a comprehensive representation of different species. For the evaluation, the natriuretic peptide and insulin neuropeptide families were used as representative model systems (Figure 2A,B). These neuropeptide families were selected due to their wide-ranging diversity, extensive literature, and presence in vertebrates and invertebrates, making them well-represented in the UniProtKB database. For each neuropeptide family, a negative dataset was created from the dataset by removing all known sequences for the respective neuropeptide family (885 natriuretic peptides and 2,660 insulin sequences).

The pipeline was able to precisely identify hormone peptides and non-hormone peptides, as evidenced by the Matthews correlation coefficient (MCC) of 0.99 for the natriuretic peptide family and 0.93 for the insulin neuropeptide family (Table S1). Moreover, the pipeline was very sensitive, with a true positive rate (TPR) of 0.99 for both peptide families. Notably, no false positives were detected for the natriuretic peptide family, and the false positive rate (FPR) for insulin was extremely low at 1.3×10^{-4}.

PeptideMiner was then benchmarked against the widely used sequence similarity search algorithm BLASTp, using the sequences of human natriuretic peptide A and human insulin. Both human precursor proteins of these neuropeptides were queried against the same database employed for the 10-fold cross-validation using protein-protein BLAST (BLASTp, Figure 2, Table S2). PeptideMiner outperformed BLASTp for the insulin peptide family and was marginally better for the natriuretic peptide family. Specifically, BLASTp exhibited an MCC of only 0.76 and a TPR of 0.58, whereas PeptideMiner achieved an MCC of 0.93 and a TPR of 0.99, indicating an overall superior performance of PeptideMiner. Notably, both BLASTp and PeptideMiner demonstrated negligible FPR for both peptide families.

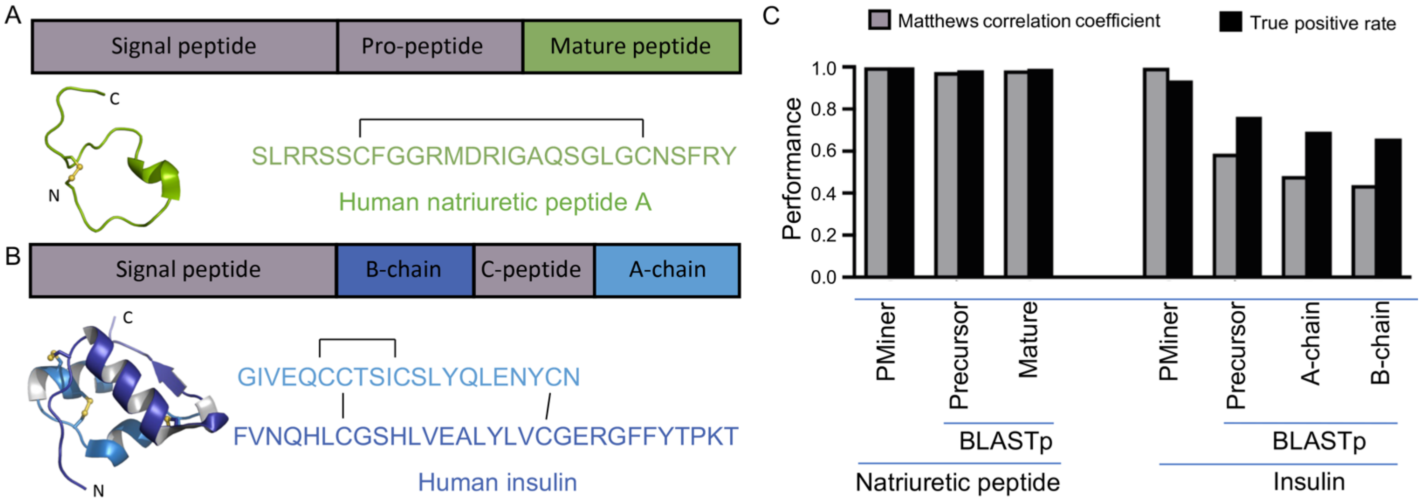

Figure 2. PeptideMiner performance comparison with BLASTp using human insulin and natriuretic peptide A as representative models. **A.** Representation of the precursor protein organization, 3D structure (PDB:7brj), and mature peptide sequence and disulfide bond connectivity of human natriuretic peptide A. **B.** Representation of the precursor protein organization, 3D structure (PDB:3w7y), and mature peptide sequence and disulfide connectivity of human insulin. **C.** Results of the 10-fold cross-validation performance comparison of PeptideMiner (PMiner) versus BLASTp from a library of 300,000 random sequences (Table S2) based on Matthews correlation coefficient and true positive rates. In panels A and B, disulfide bonds are depicted as black lines in the peptide sequence and as yellow balls and sticks in the 3D structures.

Discovery of novel natriuretic peptides

Natriuretic peptides have variable lengths, but all display a 17-amino-acid loop cyclized by a disulfide bridge [46]. These peptides play crucial roles in renal functions, cardiovascular system homeostasis, endothelial cell proliferation, and sympathetic outflow, among other physiological processes [47]. In mammals, there are three main members of the natriuretic peptide family: atrial natriuretic peptide (ANP), B-type natriuretic peptide (BNP), and C-type natriuretic peptide (CNP). NPR-A serves as the primary receptor for ANP and BNP, while NPR-B acts as the primary receptor for CNP. All three natriuretic peptides bind to NPR-C, which primarily functions as a clearance receptor [46]. By contrast, knowledge about invertebrate natriuretic peptide signaling systems is limited. Some evidence for invertebrate natriuretic peptides has been found in the hearts of oysters, blue crabs, and the earthworm *Lumbricus terrestis* [48, 49]. Natriuretic peptide receptors have also been identified in insects, crustaceans, arachnids, mollusks, and even cnidarians, although research on these systems is scarce. Natriuretic peptides are commonly observed in snake venoms, where they affect the prey's cardiovascular system to induce hypotension [50-52]. Furthermore, natriuretic peptides have been observed in the venoms of the platypus *Ornithorhynchus anatinus*, the scorpion *Tityus serrulatus*, the stone fish *Synanceia horrida*, and several lizard species [53-56].

In our search for natriuretic venom peptides across 49 species (Table S3), we identified 11 precursors that displayed the characteristic natriuretic peptide motif C-X_{15}-C (Figure S3). These precursors were identified in snakes (*Micrurus lemniscatus carvalhoi* with three precursors and *Naja Kaouthia* with three precursors), jellyfish (*Chironex fleckeri* with two precursors), leech *(Hirudo nipponia* with one precursor), centipede (*Scolopendra morsitans* with one precursor), and stone fish (*Synanceia horrida* with one precursor) (Figure 3). The natriuretic peptide Sh-NP from *Synanceia horrida* was recently independently identified at the nucleic acid level [56]. The remaining predicted mature peptides have not been previously described from these species and are considered novel putative natriuretic peptides. While the 15 amino acids between the two cysteine residues show relative conservation, the N- and C-terminal tails exhibit considerable variation in length and composition (Figure 3).

Natriuretic peptides in snakes

The three precursors of the South American coral snake *Micrurus lemniscatus carvalhoi* (Mlc) contain two predicted mature natriuretic peptides, Mlc-NP-I and Mlc-NP-II (Figure 3). Natriuretic peptides in snake venoms are hypothesized to contribute to the rapid loss of consciousness of prey [51, 57]. Both exhibit long N-terminal tails (10 residues) and C-terminal tails (14 and 12 residues, respectively), typical of elapid natriuretic peptides. Mlc-NP-I and Mlc-NP-II are highly similar to known natriuretic peptides Mc-NP (both

98% identity) from the painted coral snake *Micrurus corallinus* and the Uruguayan coral snake *Micrurus altirostris* Ma-NP (75% and 95% identity, respectively) [58, 59]. Mlc-NP-I differs only by one amino acid (Gly^{10} vs. Cys^{10}; residue numbering indicates the position within the conserved cysteine residues for ease of comparison) from Mc-NP. Gly^{10} is highly conserved, and the role and impact of the cysteine substitution in Mc-NP is unclear.

In the case of the monocled cobra *Naja kaouthia* (Nk), the three precursors are predicted to produce two mature peptides Nk-NP-I and Nk-NP-II, with only Nk-NP-II being previously described (Figure 3) [60]. Similar to the Mlc-NPs, Nk-NP-I has a five-residue N-terminal tail and a 16-residue C-terminal tail. Interestingly, the C-terminal tail contains two additional cysteine residues in a C-X_1-C pattern, suggesting a natriuretic peptide with an additional disulfide bond. The significance of this is not yet known. By contrast, Nk-NP-II has a short N-terminal tail (five residues) and no C-terminal tail, which is more typical of CNPs such as those found in Viperid snake venom [51, 61]. Nk-NP-II shares an identical sequence to a transcript annotated as waglerin peptide 1, identified in the venom transcriptome of the Bornean-keeled green pit viper, *Tropidolaemus subannulatus* [60]. The waglerins are lethal peptides identified in the venom of *Tropidolaemus wagleri* [62] targeting nicotinic acetylcholine receptors [63]. This transcript contains two peptides with the characteristic Cys-X_3-Cys motif of the waglerin peptides in addition to a natriuretic peptide Cys-X_{15}-Cys motif, identified by PeptideMiner. The encoding of waglerins and CNPs as multi-domain precursor proteins has previously been observed in viperid venom [64], but was not identified in this particular elapid transcript [60]. Nk-NP-II also displays high similarity to the Okinawa habu pit viper *Protobothrops flavoviridis* Pf-CNP (91% identity) but has a Met^2Gly and a Leu^9His substitution, removing a positive charge. Nk-NP-II is the first reported CNP observed in an elapid venom.

Natriuretic peptides in jellyfish

Two natriuretic peptide precursors were identified in the jellyfish *Chironex fleckeri* (Cf), which contain a different but closely related predicted mature natriuretic peptide: Cf-NP-I and Cf-NP-II (Figure 3). These venom peptides exhibit less than 50% identity with any known natriuretic peptides but share several conserved residues in the C-X_{15}-C motif, including Ile^6, Asp^7, Arg^8, Ile^9, Ser^{13}, Leu^{15} and Gly^{14}. Notably, neither Cf-NP-I nor Cf-NP-II has a predicted signal peptide, and alignment with other natriuretic peptides suggests that these are partial precursors (Figure S3). This discovery marks the first evidence of a natriuretic peptide in a Cnidarian. Previous genome annotation identified natriuretic peptide receptors in a single species in this phylum, although this has yet to be confirmed at the protein level [65].

Natriuretic peptides in leeches

A single natriuretic peptide precursor was discovered in the leech *Hirudo nipponia*, which gives rise to a predicted 42-amino-acid-long natriuretic peptide, Hn-NP-I. Although Hn-NP-I has low similarity to known natriuretic peptides, it contains five of the most conserved amino acids within the C-X_{15}-C motif, including Phe^{2}, Arg^{8}, Ile^{9}, Gly^{10} and Ser^{13}, and we therefore consider this a natriuretic peptide-like sequence, representing the first of its kind in the salivary gland of an annelid. While annelids possess the natriuretic peptide signaling system, no endogenous ligands have been reported [49].

Natriuretic peptides in centipedes

In the centipede *Scolopendra morsitans*, a single natriuretic precursor was identified, giving rise to a predicted 27-residue-long natriuretic peptide, Sm-NP-I. Of the 15 amino acids within the C-X_{15}-C motif, 7 are conserved in Sm-NP-I. Furthermore, Sm-NP-I possesses a C-terminal tail but lacks an N-terminal tail. This marks the first observation of a natriuretic peptide in the venom of a centipede. Notably, natriuretic peptides in arthropod venoms are rare, with Ts-NP from the scorpion *Tityus serrulatus* being the only previously reported arthropod venom natriuretic peptide [61]. Sm-NP-I and TsNP exhibit notable differences, with their mature peptides sharing an observed identity of 55% [53]. Unlike Sm-NP, TsNP lacks a C-terminal tail but possesses an N-terminal tail [61]. In humans, residues important for binding NP receptors lie within the intramolecular ring formed by the disulfide bond. However, in human ANP, the C-terminal tail is additionally required for binding to NPR-A. Thus, the N- and C-terminal tails of natriuretic peptides could be important for modulating binding and selectivity.

A

Species	Name	Mature natriuretic peptide	Length	UniProt
Homo sapiens	ANP	----SLRRSSCFGGRMDRIGAQSGLGCNSFRY------------	28	P01160
	BNP	-SPKMVQGSGCFGRKMDRISSSSGLGCKVLRRH-----------	32	P16860
	CNP	------LSKGCFGLKLDRIGSMSGLGC-----------------	21	P23582
Micrurus lemniscatus carvalhoi	**Mlc-NP-I**	GLAKEALGDGCFGQRIDRIGNVSGMGCNHVRTDPAPTALAR---	41	
	**Mlc-NP-II**	GLAKEALGEGCFGNRIDRIGDVSGMGCNHKTPAPKAPLR-----	39	
Naja kaouthia	**Nk-NP-I**	-----GPPDGCFGLKLDHIGTMSGLGCNGGSK-PIPTLTPCGCL	38	
	**Nk-NP-II**	-----AGGKGCFGLKLDRIGSTSGLGC-----------------	38	
Scolopendra morsitans	**Sm-NP-I**	---------GCAGTSLDRIGISSRQLCVSPTGICTV--------	27	
Hirudo nipponia	**Hn-NP-I**	IEVDEVGSDGCFSEELVRIGSWSDILCGMENLFKGLRAKLVG--	42	
Chironex fleckeri	**Cf-NP-I**	------MSSTCRNEFIDRISNTSMLGCSCFKSSLNAMK------	32	
	**Cf-NP-II**	------MSSTCRNEFIDRISNTSILGCSCFKSSLNAMK------	32	
Synanceia horrida	**Sh-NP**	--GASTSRSGCFGHKMDRIGTISGMGC-----------------	25	
Micrurus altirostris	Ma-NP	GLAKEALGEGCFGNRIDRIGDVSGMGCNRRTPAPKAPLR-----	39	F5CPE8
Micrurus corallinus	Mc-NP	GLAKEALGDGCFGQRIDRICNVSGMGCNHVRTDPAPTALAR---	41	P79799
Protobothrops flavoviridis	Pf-CNP	-------GKGCFGHKLDRIGSTSGLGC-----------------	20	P0C7P5
Bothrops jararaca	Bj-CNP	-----GAAKGCFGLKLDRIGTMSGLGC-----------------	22	Q6LEM5
Lachesis muta muta	Lmm-CNP	-----RVGDGCFGLKLDRIGSMSGLGC-----------------	22	Q27J49
Tityus serrulatis	TsNP	------KLSGCFGFKLDRIGTMSGLGC-----------------	21	P0DMD6

B

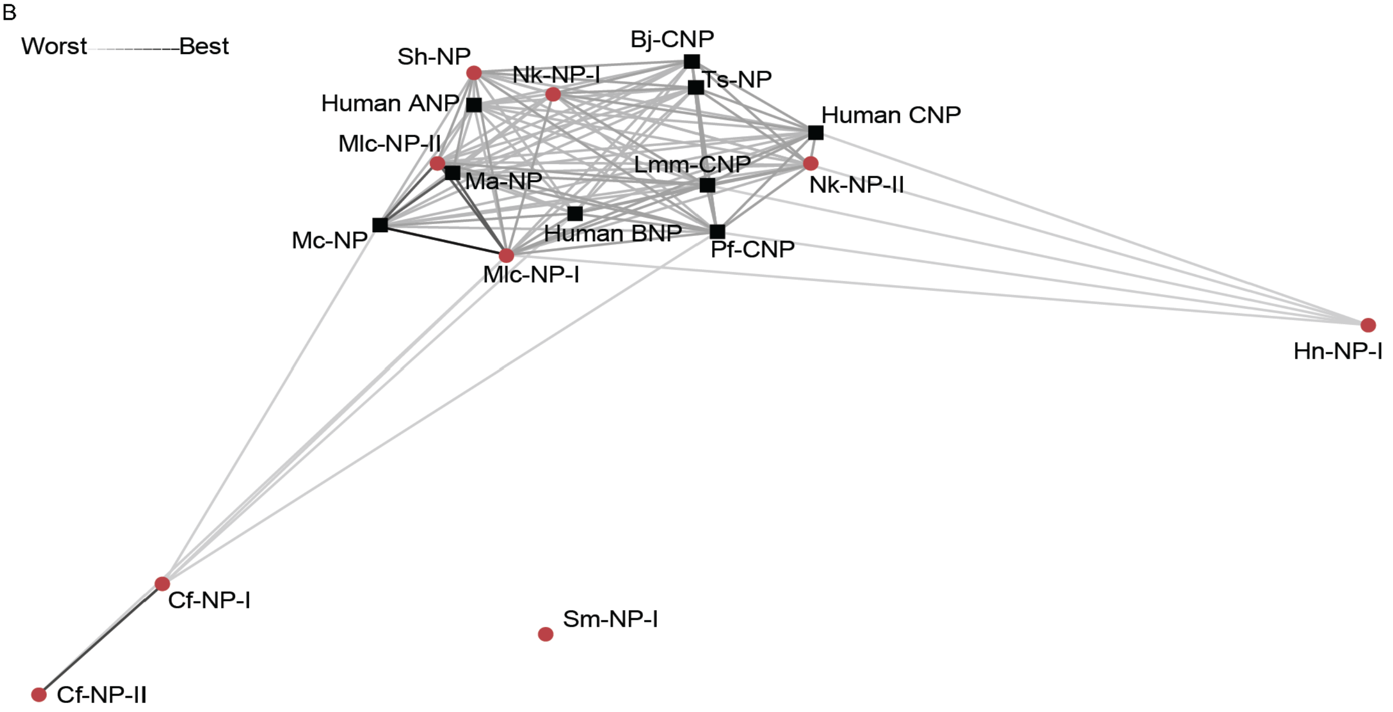

Figure 3. Sequence alignment and clustering of mature natriuretic peptides identified in venom gland transcriptomes aligned with human atrial natriuretic peptide (ANP). A. In the sequence alignment, cysteine residues are shaded yellow, N-terminal tail residues are colored green, and C-terminal tail residues gray. Novel natriuretic peptides identified in this study are bolded. Selected published natriuretic peptide sequences are shown in the lower panel for comparison. Sh-NP was novel when the study was conducted, but

was recently independently identified [56], validating our approach. B. Pairwise blast clustering of natriuretic peptides with human ANP, BNP, and CNP. Sequence similarity is denoted by line length and thickness. Human ANP sequences and previously reported NPs are represented as black squares, and novel sequences are red colored circles.

Discovery of novel Conus venom insulins

Insulin belongs to the insulin superfamily, which is highly conserved throughout the animal kingdom [66]. In humans, insulin is produced by the pancreatic β-cells in the Islets of Langerhans and plays a crucial role in regulating glucose homeostasis by facilitating glucose uptake into liver, fat, and skeletal muscle cells and suppressing gluconeogenesis in the liver [67, 68]. Insulin comprises two peptide chains (A and B) connected by two interchain disulfide bonds (C^{7}_{A}-C^{7}_{B} and C^{20}_{A}-C^{19}_{B}) (Figure 2).

The 21-residue A-chain has an additional intrachain disulfide bond (C^{6}_{A}-C^{11}_{A}) and displays an α-helical secondary structure on the N-terminal and C-terminal ends. The 30-residue B-chain features a central α-helix and a β-strand [67] that together form a characteristic and evolutionarily conserved 3D structure (Figure 2) [69]. Mature insulin is derived from a single-chain precursor protein with a signal peptide, followed by the A-chain, C-peptide, and B-chain [67]. After translation in the rough endoplasmic reticulum as preproinsulin, the signal peptide is cleaved, and proinsulin is folded and sorted into immature secretory granules for C-peptide excision and subsequent processing into mature insulin [70]. At higher micromolar concentrations, insulin dimerizes and forms hexamers in the presence of zinc, enhancing its stability and preventing fibrilization [71, 72]. Vertebrate-like conoinsulins share a cysteine framework similar to that of human insulins [73]. Conversely, molluskan insulin peptide (MIP)-like conoinsulins possess a cysteine motif resembling endogenous mollusk insulins with three A-B interchain disulfide bonds and one A intrachain disulfide bond (Figure 4) [74].

Marine predatory cone snails (genus *Conus*) are a group of venomous snails that utilize their venom for predation and defense [75-77]. The diet of cone snails varies and is broadly classified as fish-hunting (piscivorous), mollusk-hunting (molluscivorous), or worm-hunting (vermivorous). Fish-hunting cone snails have developed three distinct hunting strategies: “taser and tether”, “net engulfment”, and “strike and stalk” [78]. Their venoms comprise a wide diversity of bioactive peptides, and insulin-like peptides have been observed in several species [15, 79-85]. In this study, PeptideMiner was used to search 23 previously unpublished venom transcriptomes for new insulin homologs. We searched 20 venom duct transcriptomes (including two *C. imperialis* and two *C. tulipa* transcriptomes), two nerve ring transcriptomes (*C. catus and C.*

textile), and one salivary gland transcriptome (*C. geographus*) across 18 cone snail species (Table S3). After removing incomplete precursors, PeptideMiner identified 87 unique precursors from 16 of the 18 studied cone snail species (Figure 4, Figures S4-7). Neither *C. rattus*, nor *C. imperialis*, both worm hunters, returned any conoinsulin precursors. The predicted mature peptides (connected B- and A-chains without the C-peptide) were manually assessed and cross-checked with cleavage sites predicted by NeuroPred [86] and identified sequences of conoinsulins at the protein level (e.g., Con-Ins G1, G3 [87], G121 [75] from *C. geographus*). Insulin precursor sequences are typically cleaved at dibasic cleavage sites (Arg-Arg or Lys-Arg) or, in some cases, at single basic sites (Arg) [88].

To assess whether any of the 87 conoinsulin precursors were novel, we compared them to known conoinsulin and insulin sequences obtained from UniProt and relevant literature. A BLASTp search revealed that only 15 of the 87 precursors were previously annotated, including 8 MIP-like insulins (Con-Ins Pl1, M2, Tx1, Tx2, Va1, Pl174, Ebr1a and Ebr1b) and 7 vertebrate-like insulins (Con-Ins G1b, G3, G121 Tu478, Tu479, Tu304, Tu073), yielding 72 novel conoinsulin precursors. Removal of identical sequences in the predicted B- and A-chains from these 72 precursors resulted in 57 novel and unique mature conoinsulins across 14 species, including 5 vertebrate-like, 33 MIP-like, and 19 'other' conoinsulins that contained an odd number of cysteine residues (Table S4).

Of the 87 conoinsulin sequences discovered, 23 were vertebrate-like conoinsulins with the same three disulfide bond pattern as observed in human insulin (Figure S4). Vertebrate-like precursors from fish-hunters were predominantly identified in *C. geographus* and *C. tulipa*. 45 conoinsulins had 8 cysteine residues and were therefore categorized as MIP-like conoinsulins (Figure S5). 19 conoinsulins could not be classified as either vertebrate or molluskan conoinsulins as they featured an odd number of cysteine residues in the A- or B-chain (Figure S6), the significance of which is not known at present.

A single distinct conoinsulin precursor was identified in the salivary gland transcriptome of *C. geographus,* which we named G-SG-1 (Figure 4). It is similar to the MIP-like venom conoinsulins Im1 and *C. ebreaus* hormone insulin-related peptide [89]. It is distinct from both *C. geographus* signaling insulin (NR-G1) [85] and all other *C. geographus* venom conoinsulins with low precursor sequence conservation apart from the cysteine framework (Figure 4) [79]. G-SG-1 is the first conoinsulin identified from the salivary gland of a cone snail. Cone snail venom peptides were previously identified in the salivary glands of several species, including *C. pulicarius,*[90] *C. episcopatus,*[91] and *C. quercinus.*[92] Their role in envenomation remains unclear, but they have been proposed to function endogenously or enhance venom potency [92].

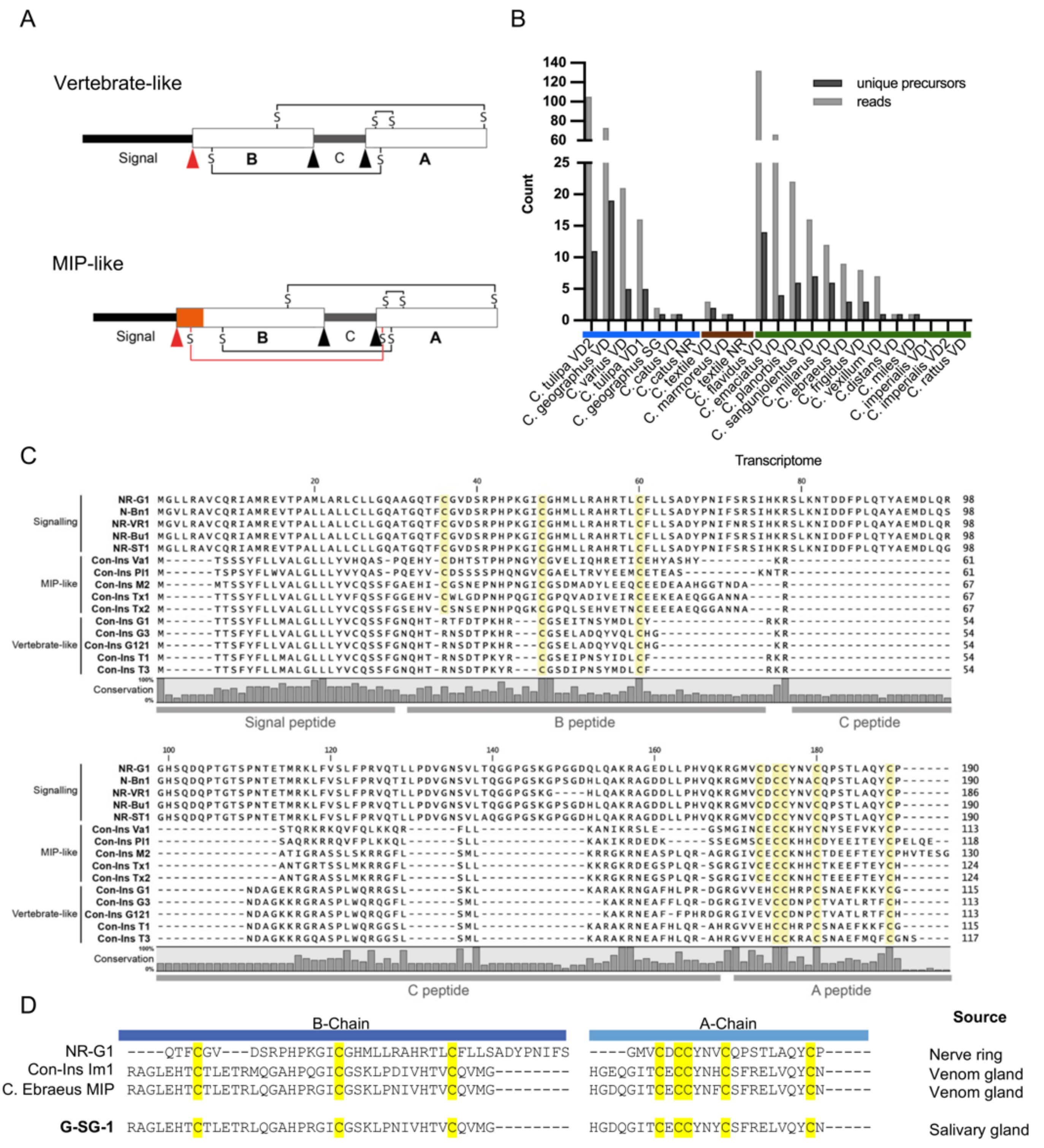

Figure 4. Discovery of novel conoinsulins. A. Schematic representation of the secondary structure of vertebrate-like and MIP-like conoinsulin precursors. All sequences contain a signal peptide, B-chain, C-peptide, and A-chain. Predicted proteolytic cleavage sites are indicated with black arrows, and predicted processing sites for removal of the signal sequence are indicated with red arrows. The orange box indicates amino acid insertions, including the extra cysteine residue forming the 4th disulfide bond in MIP-like insulins.

B. The total number of different reads per transcriptome (gray) and the number of unique precursors (black). Transcriptome tissues: VD, venom duct; SG, salivary gland. Prey of species is denoted by colored bars: fish-hunting (blue); mollusk-hunting (brown); worm-hunting (green). **C.** Sequence alignment of endogenous cone snail insulin precursors with selected venom insulins (for the complete list, see Figures S4-6). Cysteine residues are highlighted in yellow; the level of conservation is illustrated below the alignment. **D.** Sequence alignment of non-venom conoinsulins. A- and B-chains are labeled, and cysteine residues are shaded yellow. Sequences discovered in this work are bolded, and the tissue of their discovery is indicated on the right.

Pharmacological characterization of vertebrate-like conoinsulin at the human insulin receptor

As a proof-of-concept of using PeptideMiner to discover new venom neuropeptides with pharmacological or even therapeutic value, we synthesized several vertebrate-like conoinsulins using solid-phase peptide synthesis (SPPS), folded them, and tested them at the human insulin receptor-B (hIR-B), the most pharmaceutically relevant human insulin receptor subtype [93, 94]. A competition binding analysis to hIR-B of selected conoinsulins against Eu-labelled insulin was carried out. Results are expressed in IC_{50}, reflecting the concentration of the competing ligand (Con-Ins) displacing 50% of the specific binding of Eu-insulin.

We focused on vertebrate-like conoinsulins G3c, G4b, and G7 due to their overall structural similarity to human insulin and insulin's clinical significance in treating diabetes [95]. Con-Ins G4b and G7 bound to hIR-B with nanomolar affinities (IC_{50} 35.4 nM and 124.2 nM, respectively), while no competition with human insulin was evidenced for Con-Ins G3c at up to 3 μM (Figure 5). The IC_{50} of Con-Ins G4b (35.4 nM) was similar to Con-Ins G3 (46.8 nM [80]. Con-Ins G4b differs from Con-Ins G3 by a six-residue C-terminal extension of the B-chain, akin to human insulin, albeit with low sequence similarity. This extension has little effect on hIR-B binding. The main difference between Con-Ins G7 and Con-Ins G3 is a five-residue N-terminal extension, also displayed by human insulin. Remarkably, Con-Ins G7 showed potent hIR-B binding yet no improvements in affinity compared to human insulin. Notably, Con-Ins G3c differs from Con-Ins G3 only at a single residue in the B-chain ($Gly_B{}^{10}Val$), which completely abolishes hIR-B binding. This result highlights the importance of $Gly_B{}^{10}$, which is highly conserved within venom conoinsulins as well as across species in endogenous insulins and is identified as part of the hIR S1 binding site [96].

The three-dimensional structures of the three tested conoinsulins were predicted using AlphaFold2 [97] and compared to the crystal structure of human insulin (Figure 5B). Cα–root mean square deviation (Cα–RMSD) for all residues vs the human insulin crystal structure (PDB: 3wy7) were 0.8, 1.7, and 1.0 Å for G3c, G4b, and

G7, respectively (Cα–RMSD for the AlphaFold2 predicted human insulin structure was 0.5 Å for comparison). These three conoinsulins are predicted to adopt the insulin-like fold, the most notable deviation being the elongation of the B-chain termini.

A

	B-Chain	A-Chain	%ID compared to human insulin: B-chain	A-chain
Human Insulin	-------FVNQHLC$_1$GSHLVEALYLVC$_2$GE-RGFFYTPKT	GIVEQC$_3$C$_4$TSIC$_5$SLYQLENYC$_6$N--		
**Con-Ins G3c**	-----NSDTPKHRC$_1$VSELADQYVQLC$_2$H*----------	GIVγVC$_3$C$_4$DNPC$_5$TVATLRTFC$_6$H--	25%	43%
**Con-Ins G4b**	-----NSDTPKHRC$_1$GSELADQYVYLC$_2$HGERNDA*----	GIVγVC$_3$C$_4$DNPC$_5$TVATLRTFC$_6$H--	23%	43%
**Con-Ins G5**	TNIPANSDTPKHRC$_1$GSELADQYVQLC$_2$H*----------	GIVγVC$_3$C$_4$DNPC$_5$TVATLRTFC$_6$H--	30%	43%
**Con-Ins G7**	TNIPATLILQKHRC$_1$GSELADQYVQLC$_2$H*----------	GIVγVC$_3$C$_4$DNPC$_5$TVATLRTFC$_6$H--	30%	43%
**Con-Ins T3b**	-----NSDTOKYRC$_1$GSDIPNSYMDLC$_2$F-----------	GVVDHC$_3$C$_4$KRAC$_5$SNAEFMQFC$_6$GNS	20%	33%
Con-Ins G1	-----TFDTOKHRC$_1$GSγITNSYMDLC$_2$YR----------	GVVγHC$_3$C$_4$HRPC$_5$SNAEFKKYC$_6$*--	24%	45%
Con-Ins G3	-----NSDTPKHRC$_1$GSELADQYVQLC$_2$H*----------	GIVγVC$_3$C$_4$DNPC$_5$TVATLRTFC$_6$H--	30%	43%

B

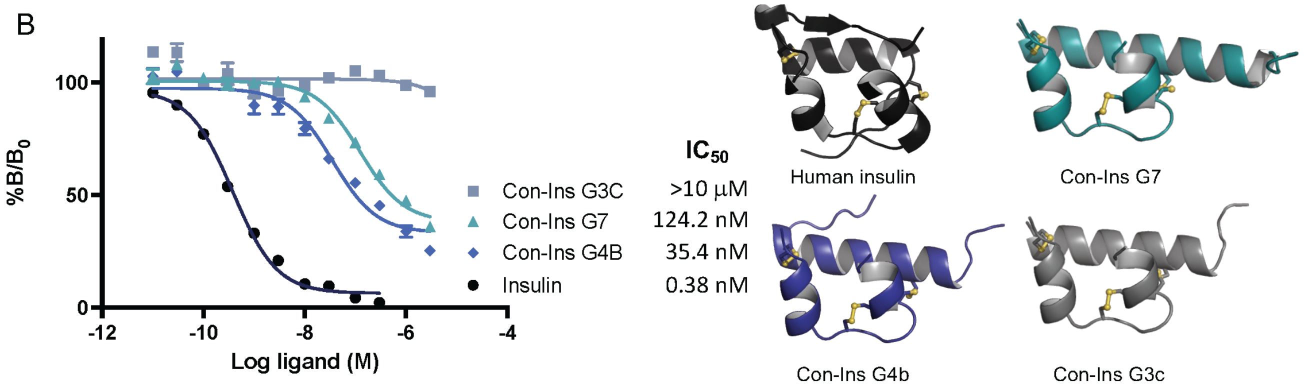

Figure 5. Sequence alignment and relative binding affinity of newly identified and synthesized conoinsulins to the human insulin receptor B (hIR-B). A. Amino acid sequence alignment of the A- and B-chains of selected conoinsulins and human insulin. **B.** Competition binding affinity to hIR-B between conoinsulins and Eu-labelled human insulin, and AlphaFold2 predicted three-dimensional structures of Con-Ins G7 (green), Con-Ins G4b (blue), and Con-Ins G3c (gray) are presented next to the human insulin crystal structure (black, PDB:3w7y). In panel A, the percentage amino acid sequence identity (%ID) to human insulin calculated by ClustalO [41] is displayed on the right for both the B- and A-chains. Predicted post-translational modifications are highlighted in red: O, 4-hydroxoproline; γ, 4-carboxyglutamate; *, C-terminal amidation. The cysteine connectivity for all sequences is C_1-C_4; C_2-C_6; C_3-C_5. Sequences discovered in this work are in bold. In panel B, results are expressed as a percentage of Eu-insulin bound without competing ligands (%B/B_0). Plotted values represent means +SEM (n=3, each comprising three technical replicates). IC_{50} is the concentration of the competing ligand (Con-Ins) displacing 50% of the specific binding of Eu-insulin.

Discussion

Neuropeptides are often referred to as the signaling molecules of life due to their ancient character and pivotal roles in various biological processes, including neurotransmission, inter- and intra-cellular signaling, and regulating complex behaviors. Their involvement spans a wide array of physiological functions, including pain perception, feeding behavior, stress and fear response, cardiovascular functions, fluid balance, and reproduction, amongst many others [19, 20, 98]. Their evolutionary conservation across diverse species underscores this fundamental physiological importance, and their involvement in numerous diseases has attracted substantial scientific interest.

The high degree of conservation and physiological significance of neuropeptides may also explain the presence of neuropeptide-like peptides in venoms, where they presumably contribute to prey capture or predator deterrence [99]. The unexplored diversity of neuropeptides within venoms presents a unique opportunity to discover novel pharmacological tools and therapeutic leads. However, the efficient identification and mapping of neuropeptides using traditional search algorithms has been challenging due to the short lengths and high sequence variations of these signaling molecules [26]. We, therefore, developed PeptideMiner, a highly efficient peptide search tool capable of overcoming these bottlenecks and facilitating the identification and mapping of neuropeptides across a wide range of species and databases.

PeptideMiner, an enhanced computational tool to efficiently search for neuropeptides

PeptideMiner is a highly versatile, robust, Python-based pipeline that harnesses peptide family-specific profile hidden Markov models (profile-HMMs) to effectively search translated transcriptomic, genomic, and proteomic data for neuropeptides or peptides in general. PeptideMiner demonstrated exceptional performance with remarkably low false-positive and false-negative rates when tested against representative neuropeptide model systems, surpassing the widely utilized BLASTp method. Notably, PeptideMiner exhibits clear advantages, particularly evident in the insulin family, attributed to its extensive and divergent nature, with over 2,000 variants across the animal kingdom, including diverse mature insulins featuring additional interchain disulfide bonds in certain species [100, 101]. The methodological disparities and existing limitations of BLASTp in this context are anticipated to be intensified by the ongoing influx of new sequence data from diverse species, spurred by the advancements and widespread adoption of ‘omics’ technologies [79, 102]. Conversely, PeptideMiner can handle large datasets, sequence variations, and distant homology, thereby enabling more accurate and comprehensive neuropeptide identification across species. PeptideMiner also supports simultaneous searches across multiple species and is compatible with any peptide/protein database. Importantly, PeptideMiner is open access, written in Python, and integrates publicly available bioinformatic

tools, ensuring ease of access and user-friendliness for the broader scientific community. While primarily developed to advance the mapping of neuropeptide families across species and expedite the discovery of neuropeptides with sequence similarity and unique pharmacology or therapeutic potential, the open-access and user-friendly nature of PeptideMiner encourages its adoption by the scientific community for other applications.

The concept of profile-HMMs in computational biology emerged in the mid-90s [103] and has more recently found applications in studies involving peptides, including neuropeptides [104, 105]. For example, profile-HMMs were employed in predicting and classifying the 62 conotoxin superfamilies [104]. In another instance, a profile-HMM based on biological processing signals of neuropeptides, encompassing the signal peptide, pro-peptide cleavage site, and extracellular peptide features, was utilized to identify novel bioactive peptides, including neuropeptides, in the human proteome [105]. One further example is the use of small profile-HMMs known as 'tox-bits'. Combinations of 2-3 'tox-bits' could accurately discern toxins from non-toxin sequences in a machine-learning model [106]. These approaches, however, lack specificity to neuropeptides and do not all involve sequence homology.

By contrast, PeptideMiner specifically targets neuropeptides by constructing profile-HMMs using known precursor or mature peptide sequences from the neuropeptide family of interest. This neuropeptide-specific approach improves identification accuracy. Additionally, the neuropeptide sequences selected to build the seed alignment for the profile-HMM are not restricted to a specific taxonomic group, facilitating the detection of neuropeptide analogs with distant homology across evolutionarily diverse species. This capability is particularly suited for investigating neuropeptide evolution, identifying neuropeptides in uncharacterized species, and discovering highly divergent neuropeptides, such as those present in tissues with a high mutation rate, like venoms.

To ensure an effective search, thoughtful consideration needs to be given when constructing profile-HMMs [30, 103]. Manual curation of the multiple sequence alignments is essential for profile-HMM construction [107], which can then be publicly shared. Neuropeptide families with a limited number of known peptides or peptides from a limited taxonomic range are at a disadvantage compared to larger families present in many taxonomic lineages. It is, therefore, important to regularly update the seed alignments and profile-HMMs with novel sequence information, a process that can be automated.

In addition to the insulin and natriuretic peptide profile-HMMs discussed and utilized in this study, we have included three other neuropeptide profile-HMMs for the neurohypophyseal, tachykinin, and somatostatin families in the open-access PeptideMiner platform to facilitate profile-HMMs creation. While there are

parameters of HMMER3 that can be adjusted for profile-HMM construction (*hmmbuild*) and searching profile-HMMs against the database (*hmmsearch*) [33], PeptideMiner already demonstrates exceptional performance, and further investigations into these parameters are not expected to enhance performance substantially.

Natriuretic peptide discovery expanded to new species

Natriuretic peptides regulate fluid balance, blood pressure, and cardiovascular homeostasis [108, 109]. In humans, ANP and BNP are predominantly produced in the heart's atria (while CNP is more widely expressed in endothelial cells) and released in response to elevated blood volume and pressure, causing vasodilation. They also promote urine production and inhibit sodium reabsorption, ultimately facilitating natriuresis [108]. Furthermore, natriuretic peptides exhibit anti-inflammatory [110] and anti-fibrotic properties, rendering them promising candidates for treating heart failure, hypertension, and kidney diseases [109].

Natriuretic peptides have also been identified in animal venoms, likely owing to their cardiovascular effects for defense and prey capture. They are particularly abundant in snakes but have also been observed in the venoms of lizards, stonefish, platypuses, and scorpions [51, 53-56]. This study expanded our understanding of natriuretic peptide distribution in venomous animals, confirming that they are not limited to vertebrate venoms and underscoring their presence in diverse evolutionary lineages. Notably, natriuretic peptides were detected in the venom glands of the centipede *S. morsitans,* as well as in two previously unexplored phyla, namely the annelid *H. nippona* and the cnidarian *C. fleckeri* species. The identification of natriuretic peptides in the tentacles of *C. fleckeri* holds significance, as it provides further evidence of their presence in cnidarians beyond the genome annotation of the putative natriuretic peptide A receptor in the cnidarian *Thelohanellus kitauei* [65]. The identification of Cf-NP-I and Cf-NP-II supports the presence of natriuretic peptides in Cnidaria, opening the possibility for convergence, or that the ancestral gene encoding the natriuretic peptide precursor was present in the last common ancestor of cnidarians and bilateral animals 600-700 million years ago [98]. No natriuretic peptides were identified in the transcriptomes of mollusk venom glands or salivary glands, despite evidence of the natriuretic peptide signaling system in gastropods such as *Crassostrea virginica* and *Helix pomatia* [48, 111].

Conoinsulin discovery expands their diversity and underscores their role in envenomation

Insulin is a crucial peptide hormone regulating glucose levels and maintaining metabolic balance in humans. Its primary function lies in facilitating glucose uptake into cells, promoting its utilization for energy production and storage. Dysregulation of insulin production or impaired insulin function can lead to metabolic disorders, notably diabetes [67, 68]. Understanding the mechanisms underlying insulin action and the molecular interactions of ligands with the main human insulin receptor, hIR-B, is essential for developing more effective

and safer therapeutic strategies for managing diabetes and related conditions.

Venoms represent a new natural source for insulin-like peptides, and particularly, the venom of the marine predatory cone snail seems rich in venom insulins, playing a role in prey capture [15, 84, 85]. There is evidence for prey-specific selection pressures in shaping the variation of peptides found in cone snail venom [112], and the presence of vertebrate-like conoinsulins in piscivorous cone snails supports their use for fish capture. These vertebrate-like conoinsulins are similar to fish insulins, bind to zebrafish insulin receptors, and can induce hypoglycemic shock to facilitate prey capture [15]. This is further supported by the complete absence of vertebrate-like conoinsulins in the venom of molluscivorous or vermivorous cone snails. By contrast, mollusk hunters produce MIP-like conoinsulins characterized by an additional disulfide bond in the B-chain, suggesting an evolutionary adaptation for their specific molluskan target prey [15, 85] (Figure 6).

Cone snails employ their venom for defense as well; indeed, they can adjust their venom composition based on intended use, whether for predation or defense [75]. Cluster analysis alone cannot clearly distinguish between worm and molluskan insulins [79] (Figure 6). Piscivorous cone snails also produce MIP-like conoinsulins. Two fish-hunting species, *C. catus* and *C. varius,*, were found only to produce MIP-like conoinsulins, while *C. geographus* produces both MIP-like and vertebrate-like conoinsulins, albeit with vertebrate-like conoinsulins being predominant in the latter case (Figure 6, Figure S4). It is not well understood whether MIP-like conoinsulins are used for prey purposes or to defend against molluscan predators such as octopuses. However, based on the cluster analysis, there are no apparent similarities with octopus insulin, indicating that they may be used for prey capture or to defend against other mollusk-hunting cone snails (Figure 6).

The application of PeptideMiner substantially expanded the known diversity of conoinsulins, unveiling numerous new conoinsulin variants, thereby underscoring their role and significance in envenomation. Through the analysis of 18 cone snail species, we identified 78 novel insulin precursors and 59 novel mature conoinsulins (Figures S4-6), in addition to the previously identified MIP-like Con-Ins Pl1, M2, Tx1, Tx2, Va1, Pl174, and vertebrate-like Con-Ins G1b, G3, G121 Tu478, Tu479, Tu304, Tu073. This includes the documentation of conoinsulins in *C. catus, C. miles, C. distans, C. ebreaus, C. emaciatus, C. flavidus, C. miliaris, C. sanguinolentus* and *C. vexillum* for the first time. PeptideMiner also revealed several novel conoinsulin precursors and mature peptides in previously annotated transcriptomes of *C. catus*, *C. geographus*, *C. miles*, *C. planorbis*, *C. tulipa,* and *C. vexillum* that were not identified using BLASTp searches [75, 113-120], exemplifying the enhanced performance of PeptideMiner.

The absence of conoinsulins in *C. rattus and C. imperialis* is consistent with previous findings indicating that

conoinsulins are not universally recruited into venom glands [79]. Of note, MIP-like conoinsulin G2, identified previously [15], was not observed in our *C. geographus* transcriptomes (including a direct manual search). These results collectively highlight the diverse sequence variability and wide distribution of conoinsulins in the genus *Conus*.

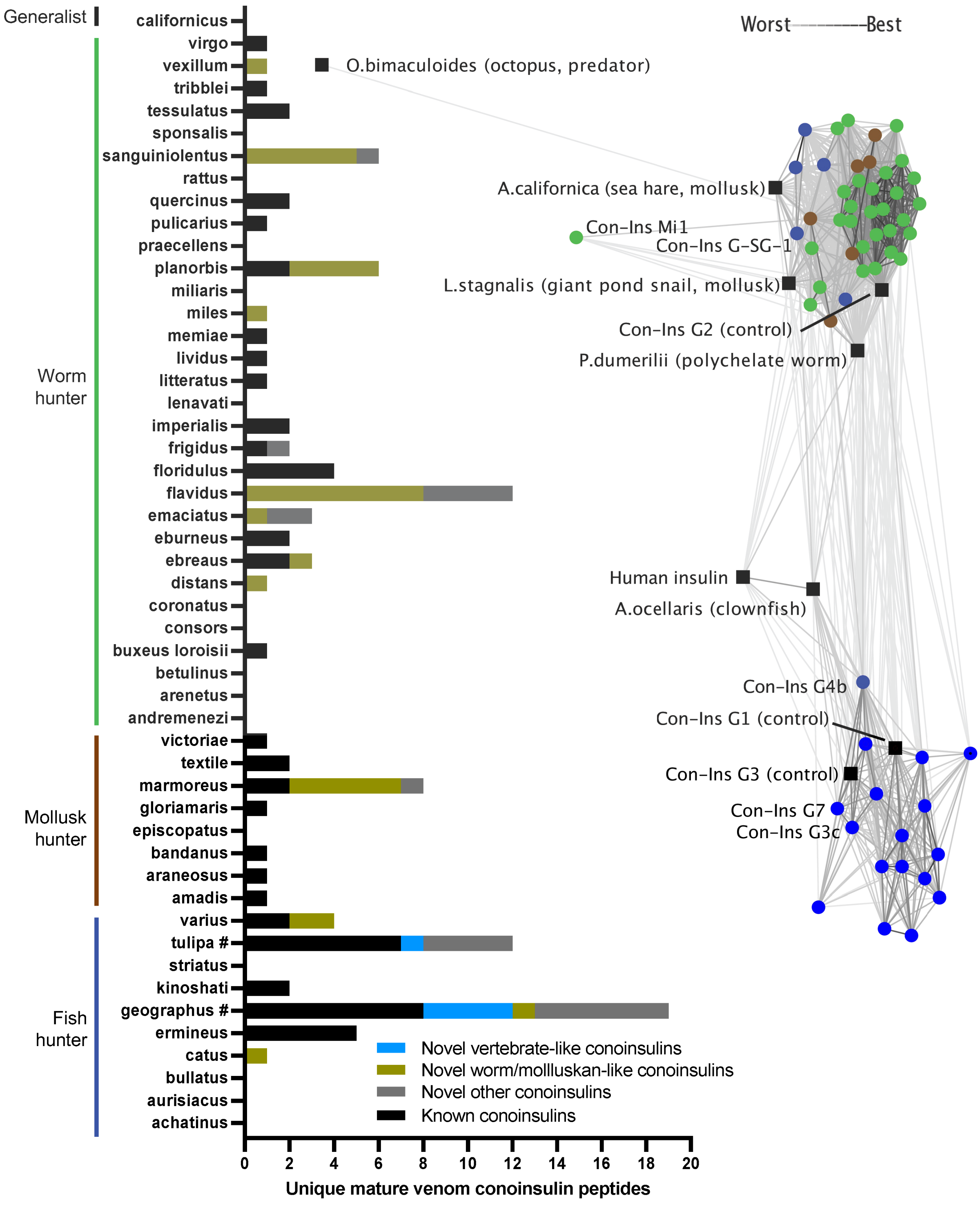

Figure 6. Number and similarity of known conoinsulin sequences and newly identified conoinsulin sequences by PeptideMiner. Known conoinsulin sequences (from UniProt, GenBank, or literature [114, 118, 121]) are presented in black, and newly identified conoinsulin sequences are further categorized as vertebrate-like (light blue), molluskan-like (olive), or other (gray). Species were grouped according to their diet: fish hunters (dark blue), mollusk hunters (brown), worm hunters (green), and generalists. # indicates net-hunters. All-against-all BLAST e-value clustering was performed using CLANS, and shows similarity between sequences (line length, and weight) of newly discovered conoinsulins (circles) and previously characterized sequences (squares).

Using PeptideMiner, we also identified five novel vertebrate-like mature conoinsulin sequences from two fish-hunting species, *C. geographus* and *C. tulipa*: Con-Ins G3c, G4b, G5, G7 and T3b (Figure 6), adding to the seven previously identified C. *geographus* and five previously identified C. *tulipa* conoinsulins [122]. We synthesized Con-Ins G3c, G4b, and G7 and assessed their binding affinity at the human insulin receptor. Con-Ins G4b and G7 demonstrated low nanomolar binding at hIR-B, underscoring the applicability of PeptideMiner and our approach for identifying new insulin-like analogs with activity at the human receptors. Con-Ins G3c was inactive at hIR-B; however, its SAR was still useful, particularly in highlighting the importance of Gly^{8}_{B} for human insulin receptor binding. Notably, the differences between Con-Ins G4b and G7 to human insulin do not involve any of the oligomerization surface residues (Ala^{12}_{A}, Leu^{13}_{A}, Glu^{17}_{A}, His^{10}_{B}, Glu^{13}_{B}, Leu^{17}_{B}), which could be exploited for the development of fast-acting insulin analogs [123, 124].

Taken together, these results underscore PeptideMiner as a powerful new discovery tool that enhances our capabilities in identifying and mapping neuropeptides across the animal kingdom and discovering new peptides with affinity for human receptors. These enhanced capabilities are expected to pave the way for developing new pharmacological probes or therapeutic leads, thereby offering promising prospects for advancing biomedical research and drug development.

Conclusions

PeptideMiner is a new and highly versatile computational discovery platform designed to efficiently identify (neuro)peptide families across diverse databases and species. Demonstrating superior performance compared to BLASTp, PeptideMiner offers several advantages in accurate neuropeptide discovery, adeptly handling short and divergent sequences, and efficiently mapping neuropeptides across evolutionarily distant species. This study highlights the untapped potential of venoms as a rich new source for neuropeptide analogs and provides a robust framework for their systematic and efficient exploration to discover valuable new pharmacological probes and therapeutic leads. PeptideMiner unveiled 36 unique conoinsulins (Figure S4) and 8 natriuretic peptides (Figure 3), substantially expanding our understanding of the molecular diversity within venoms across different animal species. Selected conoinsulins bound to the human insulin receptor, emphasizing the translational promise of this approach.

Taken together, PeptideMiner is an invaluable open-access tool poised to propel neuropeptide research forward, deepen our comprehension of neuropeptide signaling in health and disease, and expedite the discovery and development of novel pharmacological probes and therapeutic interventions.

Materials and Methods

Materials

Fmoc amino acids were from Iris Biotech GmbH (Marktredwitz, Germany), and Fmoc- Asp(t-Bu)-Wang resin (loading 0.81 mmol/g) and Fmoc-L-His(Trt)-AC TentaGel resin (loading 0.22 mmol/g) was from Rapp Polymere GmbH (Tübingen, Germany). Acetonitrile (ACN) was obtained from Merck (Bayswater, Australia). Dimethylformamide (DMF), methanol (MeOH), trifluoroacetic acid (TFA), and diethyl ether were obtained from Chem-Supply (Gillman, Australia). All solvents were obtained with the highest available purity and used without further purification. All other reagents, including N,N-diisopropylethylamine (DIEA), (O-(6-chlorobenzotriazol-1-yl)-N,N,N’,N’-tetramethyluronium hexafluorophosphate (HCTU), Anisole, 2,2′-(ethylenedioxy)diethanethiol (DODt), triisopropylsilane (TIPS), 2,2’-dipyridyldisulfide (DPDS) and trifluoromethanesulfonic acid (TFMSA) and solvents were obtained from Sigma Aldrich (Merck, Sydney, NSW, Australia) in the highest available purity. Solvents for reversed-phase HPLC (RP-HPLC) consisted of 0.05% TFA/H_2O (Solvent A) and 0.043% TFA/ACN (Solvent B). Analytical HPLC was performed with

column heating at 40°C and detection at 214 nm. Preparative HPLC was performed on a Vydac Protein and Peptide C_{18} preparative column, and crude and fractions were analyzed using RP-HPLC and electrospray ionization mass spectrometry (ESI-MS). Mass analysis of the final product was performed on a Q-Star Pulsar mass spectrometer (SCIEX, Ontario, Canada) with a Series 1100 solvent delivery system equipped with an auto-injector (Agilent Technologies Inc., Palo Alto, CA, USA) and a Kromasil Classic liquid chromatography MS (LC-MS) C_{18} column (100 Å, 3.5 µm, 150 mm x 2.1 mm). Data acquisition and processing were carried out using Analyst software v1.1 (SCIEX, Canada). Trizol was from ThermoFisher Scientific. Oligotex mRNA Mini Kit was from Qiagen (Valencia, CA, USA).

Transcriptome Preparation

The transcriptomes were sourced from unpublished in-house collections (23 *Conus* and *S. horrida* transcriptomes) or downloaded from the NCBI Sequence Read Archive (SRA) database. A full list of transcriptomes searched is provided in Table S3. For the in-house collections, total RNA was extracted from the stripped venom gland cells using Trizol (Invitrogen) reagent according to the manufacturer's instructions. mRNA was purified from the total RNA using Oligotex mRNA Mini Kit (Qiagen, Valencia, CA, USA) according to the manufacturer's instructions. cDNA library construction and sequencing were carried out using a Roche GS FLX Titanium sequencer at the Australian Genomic Research Facility (AGRF). Data assembly was carried out using Newbler 2.3 Life Science, Frederick, CO). In total, 57 animal venom organ transcriptomes from 48 species, including 39 invertebrates and 9 vertebrates across five phyla (Cnidaria, Arthropoda, Mollusca, Annelida, and Chordata) were searched for the natriuretic peptide neuropeptide family, and 23 cone snail transcriptomes comprising 20 venom ducts, two circumoesophageal nerve rings, and one salivary gland transcriptome for the conoinsulin family.

PeptideMiner Workflow

PeptideMiner is constructed as a series of steps with user-friendly output files created at each step. PeptideMiner takes as input one or more profile-HMMs of the neuropeptide family of interest and searches a database of amino acid sequences that the user can provide as multiple fasta files (for instance, one fasta file for each transcriptome) or combine into a single large fasta file database.

Hmmsearch from the HMMER3 package searches the profile-HMM against the amino acid database (in this work, translated transcriptomes). PeptideMiner runs hmmsearch and creates an output for each dataset searched

(Step 0). The hmmsearch output does not include the hit sequence; therefore, the hit read names are matched with their respective read name in the amino acid database, and the sequence is extracted. The sequence is combined with the hmmsearch output to generate a report of identified neuropeptide candidates. The bioinformatics pipeline subsequently filters, processes, and annotates this initial neuropeptide identification report.

The hmmsearch search results are stored in an SQLite database (Figure S2). To facilitate downstream processing, all hmmsearch search hits are combined into a single file (Step 2). If more than one profile-HMM was used for a single neuropeptide family, the combined hmmsearch output could contain duplicate reads. For this case, the hit with the lowest E-value is selected. The E-value is an estimate of the Expected number of errors. In other words, it represents the probability of observing such results by chance. The E-value of a read returned by hmmsearch represents the statistical significance of the hit. The lower the E-value, typically <<1, the more statistically significant the hit and the less likely it is a random hit [28].

In Step 3, the protein-coding sequences (CDS) of the hypothetical neuropeptide precursors are predicted using a Python script that extracts the sequence between the methionine, 'M', (encoding the start codon) and the stop codon or, if there is no stop codon, the end of the contig (Figure 1, Step 3). Multiple CDS can be identified from a single contig if multiple methionine residues are present. This step is relevant for protein sequences obtained by direct translation of nucleotide transcripts. The user can specify a minimum cut-off length for the CDS to minimize false positives.

The signal peptide of the predicted CDS is then identified using SignalP [34] (Figure 1, Step 4). If present, the signal peptide is subsequently excised from the sequence of the precursor to facilitate subsequent mature peptide identification.

Step 5 aims to identify the mature peptide(s) in the CDS; FASTA36 [35] aligns all the CDS to a list of known mature peptides of the neuropeptide family of interest, followed by cleavage site prediction using an algorithm from the ConoServer annotation pipeline [44] (Step 5).

The predicted mature peptides for all sequences from the same transcriptome are then compiled, and duplicate mature peptides are removed (Step 6). Protein-protein BLAST (BLASTp) is then used to annotate the predicted mature peptides by homology using a list of known neuropeptide amino acid sequences (Step 7).

PeptideMiner Search Parameters

The user can modify several parameters of PeptideMiner to optimize neuropeptide identification. Firstly, the user can specify the minimum length of the CDS in the number of amino acids, enabling the filtering of

sequence candidates that are too short (Step 3), for example, to exclude incomplete CDS or fragments.

Secondly, the user can modify the SignalP parameters to identify the signal peptide in CDS (Step 4). This step is important because the presence of a signal peptide is supportive of a neuropeptide. The user can change the SignalP D-value (the score used to discriminate signal peptides from non-signal peptides; peptides with no signal peptide have a very low D-value) and minimum signal peptide length, which are set to a default of 0.45 and 9, respectively, which is the default setting for non-transmembrane eukaryotic precursors [34]. Reducing the D-value would increase speed at the cost of sensitivity.

Finally, the user can alter three parameters for mature peptide identification (Step 5). FASTA36 [35] is used to align known mature peptides to the CDS to identify mature peptides in the CDS. The user can specify the E-value cut-off of the FASTA36 search as well as the minimum and maximum length allowed for the mature peptides. The E-value is typically dependent on the overall length of the alignment, the percentage identity, and the size of the database. Generally, a lower E-value indicates a better quality in the alignment between the mature peptide and the known peptide. The optimal E-value cut-off varies depending on the neuropeptide family because the various neuropeptides have different lengths and degrees of sequence conservation. In practice, an E-value range between $1x10^{-4}$–1 is typically used, although the cut-off will vary depending on the query sequence length and database size.

Pipeline Output

The pipeline creates several outputs for the user, including a final output file that lists all the hits and their similarity (% identity and BLASTp E-value) to known sequences (Step 8). In addition, PeptideMiner generates a report that lists the number of profile-HMMs used, the number of files searched, the number of hits the hmmsearch returned, and the final number of hits, including the number of CDS and mature peptides identified. A separate file reports the number of reads found with each profile-HMM used to search the database.

RNA isolation and transcriptome generation

All cone snails used to generate the transcriptomes were collected from the Great Barrier Reef, Queensland, Australia (GBRMP permit G10/33243.1). Cone snails were sacrificed and dissected immediately on ice. The whole venom gland was separated from the other tissues (not including the venom bulb at the proximal end and the proboscis at the distal end), and the venom gland cells were stripped out from the venom gland. Total RNA was extracted from the stripped venom gland cells using TRIzol reagent according to the manufacturer's instructions. mRNA was purified from the total RNA using an Oligotex mRNA Mini Kit according to the

manufacturer's instructions. cDNA library construction and sequencing were carried out using a Roche GS FLX Titanium sequencer at the Australian Genomic Research Facility (AGRF). Data assembly was carried out using Newbler 2.3 Life Science, Frederick, CO). Only one individual was used to generate the transcriptomes of larger species (*C. geographus, C. tulipa, C. planorbis, C. distans, C. sangunilantus, C. vexillum, C. marmoreus, C. miles, C. imperialis)* and multiple specimens were used for smaller species (e.g., six specimens for *C. catus*).

Solid-phase peptide synthesis

Synthesis of Con-Ins G3c, G4b, and G7

The linear conoinsulin A-chains were manually synthesized using Fmoc-SPPS on an Fmoc-L-His(Trt)-AC TentaGel resin on a 0.22 mmol scale. A regioselective folding strategy using acetamidomethyl (Acm), *tert*-butyl (tBu), and trityl (Trt) cysteine protecting groups was employed to achieve the A-chain intrachain and two A-B-chain interchain disulfide bonds. N-terminal Fmoc deprotection was achieved with 20% v/v piperidine in DMF for 2×5 min. Each Fmoc-protected amino acid (3 eq.) was coupled using DMF as the solvent and DIEA (1 M in DMF) with HCTU (3 eq.) as the activating agents for 50 min. The cycle of deprotection, washing, and coupling was repeated until the full-length peptide was obtained. The completed linear A-chain was cleaved by a cocktail of TFA/anisole/DODt/TIPS (94/3/2/1%) for 3 h. The cleavage mixture was then filtered, concentrated by a stream of N_2, precipitated in cold diethyl ether, and centrifuged for 5 min. The crude A-chain (544 mg) was collected as a white powder and used directly for further synthesis.

The A-chain intrachain disulfide bond was formed by dissolving the crude linear A-chain (0.102 mmol) in an H_2O/ACN mixture (4:1, 500 mL) with 5 drops of DIEA. After adding DPDS (1.0 eq.) in 1.0 mL MeOH, the mixture was stirred for 2.5 h at 40°C. The mixture was purified using a preparative RP-HPLC (15-55% solvent B in 30 min) and lyophilized.

The cyclized A-chain (31.5 μmol) and DPDS (4 eq.) were dissolved in an anisole/TFA mixture (1:9, 2 mL), and then a TFMSA/TFA mixture (1:4, 2 mL) was added. The resulting mixture was stirred at 0°C for 45 min, then precipitated in cold diethyl ether and centrifuged (5 repeats). The crude peptide was purified by a semi-preparative RP-HPLC (15-55% solvent B in 30 min) and lyophilized.

All three B-chains were purchased from GL Biochem (Shanghai, China). To a mixture of conoinsulin B-chain in acidic guanidium HCl buffer (6 M, 1 mL, pH = 5.0), conoinsulin A-chain in alkaline guanidium HCl buffer (6 M, 2 mL, pH = 8.5) was added dropwise. The resulting mixture was stirred for 15 min, purified using a semi-preparative RP-HPLC (15-45% solvent B in 30 min), and lyophilized.

To the lyophilized powder was added an aqueous solution of HCl (60 mM, 0.4 mL), acetic acid (3.15 mL), and an iodine solution (20 mM in acetic acid, 4.2 mL). The mixture was stirred for 1 h, then precipitated in cold ether and centrifuged. The crude peptide was purified by a semi-preparative RP-HPLC (15-45% solvent B in 30 min) and lyophilized.

Con-Ins G3c: A-chain (10 mg) and B-chain (10.5 mg) were used, yielding 0.5 mg of white powder. Con-Ins G7: A-chain (10 mg) and B-chain (12 mg) yielded 1.3 mg of white powder. Con-Ins G4b: A-chain (5.9 mg) and B-chain (7.5 mg) yielded 0.7 mg of white powder.

Human insulin receptor-B binding experiments

Receptor binding was measured as described previously [94]. Briefly, insulin-like growth factor 1 receptor (IGF-1R)-negative cells overexpressing the human insulin receptor-B (hIR-B) were generated. Cells were serum-starved for 4 h before lysis. Lysates were captured in a 96-well plate previously coated with anti-IR antibody. Approximately 500,000 fluorescent counts of europium-labeled human insulin (Eu-insulin) were added to each well along with increasing concentrations of unlabeled competitor and incubated for 16 h at 4°C. After washing, time-resolved fluorescence was measured using 340 nm excitation and 612 nm emission filters with the BMG Lab Technologies Polarstar fluorometer (Mornington, Australia). Insulin and synthetic conoinsulin analog curves are from three separate experiments, each point performed in triplicate. Binding affinity was expressed in IC_{50} and represents the ligand concentration (human insulin or conoinsulin) necessary to displace 50% of Eu-insulin from hIR-B.

Conoinsulin structure prediction

Three-dimensional structures for Con-Ins G3C, G4b and G7 were predicted using AlphaFold2 [97], implemented in ColabFold [125] running remotely on a Python 3 Google Compute Engine backend. The alphafold2_multimer_v3 model and no template information were used for the prediction. The highest-ranked, Amber-relaxed model was aligned with the crystal structure of human insulin (PDB:3w7y).

Clustering Analysis

To compare the identified insulin-like peptides and natriuretic peptides, we used CLANS [126] to cluster mature amino acid sequences based on all-against-all pairwise BLASTp E-values. Pairwise BLASTp searches were performed with the CLANS web-utility (https://toolkit.tuebingen.mpg.de/tools/clans) [127] using default parameters, while clustering and visualization of the resulting similarity matrix were completed with the Java-based CLANS tool, using P-values better than 1e-4 and otherwise default parameters.

Data availability

PeptideMiner is registered with bio.tools (biotools:peptideminer) and SciCrunch.org (RRID: SCR-026456), is publicly available for download from GitHub (https://www.github.com/muttenthalerlab/PeptideMiner), and is released under the GNU General Public License v3.0.

Acknowledgments

We thank Dr. Akhter Hossain for synthesizing Con-Ins G3c, G4b, and G7, Dr. Himaya Hewage for transcriptome details, A/Prof Lachlan Rash for access to the venom transcriptome of the King Baboon Spider, and Dr. Prashanth Jutty Rajan for initial training in transcriptome analysis. M.M. was supported by the European Research Council under the European Union's Horizon 2020 research and innovation program (714366) and by the Australian Research Council (DE150100784, DP190101667, FT210100266), and by the National Health and Medical Research Council (2037680). EABU was supported by the European Research Council (ERC-2021-STG under grant agreement No 101039862) and the Norwegian Research Council (FRIPRO-YRT Fellowship no. 287462).

References

1. King GF. Venoms as a platform for human drugs: translating toxins into therapeutics. Expert Opin Biol Th. 2011;11 11:1469-84.
2. Lewis RJ and Garcia ML. Therapeutic potential of venom peptides. Nat Rev Drug Discov. 2003;2 10:790-802.
3. Muttenthaler M, King GF, Adams DJ and Alewood PF. Trends in peptide drug discovery. Nature Reviews Drug Discovery. 2021;20 4:309-25.
4. Näreoja K and Näsman J. Selective targeting of G-protein-coupled receptor subtypes with venom peptides. Acta Physiologica. 2012;204 2:186-201.
5. Sharpe IA, Gehrmann J, Loughnan ML, Thomas L, Adams DA, Atkins A, et al. Two new classes of conopeptides inhibit the α1-adrenoceptor and noradrenaline transporter. Nature Neuroscience. 2001;4 9:902-7.
6. Warkentin TE and Koster A. Bivalirudin: a review. Expert Opinion on Pharmacotherapy. 2005;6 8:1349-71.
7. Zhang L, Lu SH, Li L, Tao Y-G, Wan YL, Senga H, et al. Batroxobin Mobilizes Circulating Endothelial Progenitor Cells in Patients With Deep Vein Thrombosis. Clinical and Applied Thrombosis/Hemostasis. 2011;17 1:75-9.
8. Eagles DA, Saez NJ, Krishnarjuna B, Bradford JJ, Chin YK-Y, Starobova H, et al. A peptide toxin in ant venom mimics vertebrate EGF-like hormones to cause long-lasting hypersensitivity in mammals. Proceedings of the National Academy of Sciences. 2022;119 7:e2112630119.
9. Jami S, Erickson A, Brierley S and Vetter I. Pain-Causing Venom Peptides: Insights into Sensory Neuron Pharmacology. Toxins. 2017;10 1:15.
10. Netirojjanakul C and Miranda LP. Progress and challenges in the optimization of toxin peptides for development as pain therapeutics. Curr Opin Chem Biol. 2017;38:70-9.
11. Miljanich GP. Ziconotide: neuronal calcium channel blocker for treating severe chronic pain. Curr Med Chem. 2004;11 23:3029-40.
12. Taylor K, Kim D, Nielsen LL, Aisporna M, Baron AD and Fineman MS. Day-long Subcutaneous Infusion of Exenatide Lowers Glycemia in Patients with Type 2 Diabetes. Hormone and Metabolic Research. 2005;37 10:627-32.
13. Cruz LJ, de Santos V, Zafaralla GC, Ramilo CA, Zeikus R, Gray WR and Olivera BM. Invertebrate vasopressin/oxytocin homologs. Characterization of peptides from Conus geographus and Conus straitus venoms. J Biol Chem. 1987;262 33:15821-4.
14. Craig AG, Norberg T, Griffin D, Hoeger C, Akhtar M, Schmidt K, et al. Contulakin-G, an O-Glycosylated Invertebrate Neurotensin. Journal of Biological Chemistry. 1999;274 20:13752-9.
15. Safavi-Hemami H, Gajewiak J, Karanth S, Robinson SD, Ueberheide B, Douglass AD, et al. Specialized insulin is used for chemical warfare by fish-hunting cone snails. Proceedings of the National Academy of Sciences. 2015;112 6:1743-8.
16. Robinson SD, Safavi-Hemami H, Raghuraman S, Imperial JS, Papenfuss AT, Teichert RW, et al. Discovery by proteogenomics and characterization of an RF-amide neuropeptide from cone snail venom. J Proteomics. 2015;114:38-47.
17. Schweitz H, Vigne P, Moinier D, Frelin C and Lazdunski M. A New Member of the Natriuretic Peptide Family Is Present in the Venom of the Green Mamba (Dendroaspis-Angusticeps). Journal of Biological Chemistry. 1992;267 20:13928-32.
18. Hokfelt T, Broberger C, Xu ZQ, Sergeyev V, Ubink R and Diez M. Neuropeptides--an overview. Neuropharmacology. 2000;39 8:1337-56.
19. Mendel HC, Kaas Q and Muttenthaler M. Neuropeptide signalling systems – An underexplored target for venom drug discovery. Biochemical Pharmacology. 2020;181:114129.
20. Elphick MR, Mirabeau O and Larhammar D. Evolution of neuropeptide signalling systems. J Exp Biol. 2018;221 Pt 3.
21. Buermans HP and den Dunnen JT. Next generation sequencing technology: Advances and applications. Biochim Biophys Acta. 2014;1842 10:1932-41.
22. Calvete JJ. Venomics: integrative venom proteomics and beyond. Biochem J. 2017;474:611-34.

23. Chen YP and Chen F. Identifying targets for drug discovery using bioinformatics. Expert Opin Ther Targets. 2008;12 4:383-9.
24. Escoubas P and King GF. Venomics as a drug discovery platform. Expert Rev Proteomic. 2009;6 3:221-4.
25. Oldrati V, Arrell M, Violette A, Perret F, Sprungli X, Wolfender JL and Stocklin R. Advances in venomics. Mol Biosyst. 2016;12 12:3530-43.
26. Clynen E, Liu F, Husson SJ, Landuyt B, Hayakawa E, Baggerman G, et al. Bioinformatic approaches to the identification of novel neuropeptide precursors. Methods in molecular biology. 2010;615:357-74.
27. Caers J, Verlinden H, Zels S, Vandersmissen HP, Vuerinckx K and Schoofs L. More than two decades of research on insect neuropeptide GPCRs: an overview. Front Endocrinol (Lausanne). 2012;3:151.
28. Eddy SR. Accelerated Profile HMM Searches. PLoS Comput Biol. 2011;7 10:e1002195.
29. Durbin R, Eddy SR, Krogh A and Mitchison GJ. Biological Sequence Analysis: Probabilistic Models of Proteins and Nucleic Acids. In: 1998.
30. Park J, Karplus K, Barrett C, Hughey R, Haussler D, Hubbard T and Chothia C. Sequence comparisons using multiple sequences detect three times as many remote homologues as pairwise methods. J Mol Biol. 1998;284 4:1201-10.
31. Yoon BJ. Hidden Markov Models and their Applications in Biological Sequence Analysis. Curr Genomics. 2009;10 6:402-15.
32. Eddy SR. Profile hidden Markov models. Bioinformatics. 1998;14 9:755-63.
33. Eddy SR: HMMER 3.1b. hmmer.org (2015).
34. Petersen TN, Brunak S, von Heijne G and Nielsen H. SignalP 4.0: discriminating signal peptides from transmembrane regions. Nat Methods. 2011;8 10:785-6.
35. Pearson WR. Searching protein sequence libraries: comparison of the sensitivity and selectivity of the Smith-Waterman and FASTA algorithms. Genomics. 1991;11 3:635-50.
36. Altschul SF, Madden TL, Schaffer AA, Zhang J, Zhang Z, Miller W and Lipman DJ. Gapped BLAST and PSI-BLAST: a new generation of protein database search programs. Nucleic Acids Res. 1997;25 17:3389-402.
37. Hipp DR, Kennedy D and Mistachkin J. SQLite (Version 3.27.2). SQLite Development Team, 2015.
38. Southey BR, Sweedler JV and Rodriguez-Zas SL. A python analytical pipeline to identify prohormone precursors and predict prohormone cleavage sites. Front Neuroinform. 2008;2:7.
39. Bassi S. A primer on python for life science researchers. PLoS Comput Biol. 2007;3 11:e199.
40. Edgar RC. MUSCLE: multiple sequence alignment with high accuracy and high throughput. Nucleic Acids Res. 2004;32 5:1792-7.
41. Sievers F, Wilm A, Dineen D, Gibson TJ, Karplus K, Li W, et al. Fast, scalable generation of high-quality protein multiple sequence alignments using Clustal Omega. Mol Syst Biol. 2011;7:539.
42. Waterhouse AM, Procter JB, Martin DM, Clamp M and Barton GJ. Jalview Version 2--a multiple sequence alignment editor and analysis workbench. Bioinformatics. 2009;25 9:1189-91.
43. Eddy SR. A new generation of homology search tools based on probabilistic inference. Genome Inform. 2009;23 1:205-11.
44. Kaas Q, Yu R, Jin AH, Dutertre S and Craik DJ. ConoServer: updated content, knowledge, and discovery tools in the conopeptide database. Nucleic Acids Res. 2012;40 Database issue:D325-30.
45. Suzek BE, Wang Y, Huang H, McGarvey PB, Wu CH and UniProt C. UniRef clusters: a comprehensive and scalable alternative for improving sequence similarity searches. Bioinformatics. 2015;31 6:926-32.
46. Potter LR, Yoder AR, Flora DR, Antos LK and Dickey DM. Natriuretic peptides: their structures, receptors, physiologic functions and therapeutic applications. Handb Exp Pharmacol. 2009; 191:341-66.
47. Pandit K, Mukhopadhyay P, Ghosh S and Chowdhury S. Natriuretic peptides: Diagnostic and therapeutic use. Indian J Endocrinol Metab. 2011;15 Suppl 4:S345-53.
48. Poulos JE, Gower WR, Jr., Friedl FE and Vesely DL. Atrial natriuretic peptide gene expression within invertebrate hearts. Gen Comp Endocrinol. 1995;100 1:61-8.
49. Vesely DL and Giordano AT. The most primitive heart in the animal kingdom contains the atrial natriuretic peptide hormonal system. Comp Biochem Physiol C. 1992;101 2:325-9.

50. Peterfi O, Boda F, Szabo Z, Ferencz E and Baba L. Hypotensive Snake Venom Components-A Mini-Review. Molecules. 2019;24 15.
51. Higuchi S, Murayama N, Saguchi K, Ohi H, Fujita Y, Camargo AC, et al. Bradykinin-potentiating peptides and C-type natriuretic peptides from snake venom. Immunopharmacology. 1999;44 1-2:129-35.
52. Ichiki T, Dzhoyashvili N and Burnett JC, Jr. Natriuretic peptide based therapeutics for heart failure: Cenderitide: A novel first-in-class designer natriuretic peptide. Int J Cardiol. 2018.
53. Alves RS, Ximenes RM, Jorge AR, Nascimento NR, Martins RD, Rabello MM, et al. Isolation, homology modeling and renal effects of a C-type natriuretic peptide from the venom of the Brazilian yellow scorpion (Tityus serrulatus). Toxicon. 2013;74:19-26.
54. de Plater GM, Martin RL and Milburn PJ. A C-type natriuretic peptide from the venom of the platypus (Ornithorhynchus anatinus): structure and pharmacology. Comp Biochem Physiol C Pharmacol Toxicol Endocrinol. 1998;120 1:99-110.
55. Fry BG, Roelants K, Winter K, Hodgson WC, Griesman L, Kwok HF, et al. Novel venom proteins produced by differential domain-expression strategies in beaded lizards and gila monsters (genus Heloderma). Mol Biol Evol. 2010;27 2:395-407.
56. Ziegman R, Undheim EAB, Baillie G, Jones A and Alewood PF. Investigation of the estuarine stonefish (Synanceia horrida) venom composition. J Proteomics. 2019;201:12-26.
57. Vink S, Jin AH, Poth KJ, Head GA and Alewood PF. Natriuretic peptide drug leads from snake venom. Toxicon. 2012;59 4:434-45.
58. Schweitz H, Vigne P, Moinier D, Frelin C and Lazdunski M. A new member of the natriuretic peptide family is present in the venom of the green mamba (Dendroaspis angusticeps). J Biol Chem. 1992;267 20:13928-32.
59. Zhang Y, Wu J, Yu G, Chen Z, Zhou X, Zhu S, et al. A novel natriuretic peptide from the cobra venom. Toxicon. 2011;57 1:134-40.
60. Xie B, Dashevsky D, Rokyta D, Ghezellou P, Fathinia B, Shi Q, et al. Dynamic genetic differentiation drives the widespread structural and functional convergent evolution of snake venom proteinaceous toxins. BMC Biology. 2022;20 1.
61. Soares MR, Oliveira-Carvalho AL, Wermelinger LS, Zingali RB, Ho PL, Junqueira-de-Azevedo IL and Diniz MR. Identification of novel bradykinin-potentiating peptides and C-type natriuretic peptide from Lachesis muta venom. Toxicon. 2005;46 1:31-8.
62. Schmidt JJ, Weinstein SA and Smith LA. Molecular properties and structure-function relationships of lethal peptides from venom of Wagler's pit viper, Trimeresurus wagleri. Toxicon. 1992;30 9:1027-36.
63. Tsai MC, Hsieh WH, Smith LA and Lee CY. Effects of waglerin-I on neuromuscular transmission of mouse nerve-muscle preparations. Toxicon. 1995;33 3:363-71.
64. Tan CH, Tan KY and Tan NH. De Novo Assembly of Venom Gland Transcriptome of Tropidolaemus wagleri (Temple Pit Viper, Malaysia) and Insights into the Origin of Its Major Toxin, Waglerin. Toxins (Basel). 2023;15 9.
65. Yang Y, Xiong J, Zhou Z, Huo F, Miao W, Ran C, et al. The genome of the myxosporean Thelohanellus kitauei shows adaptations to nutrient acquisition within its fish host. Genome Biol Evol. 2014;6 12:3182-98.
66. Shabanpoor F, Separovic F and Wade JD. The human insulin superfamily of polypeptide hormones. Vitam Horm. 2009;80:1-31.
67. Weiss M, Steiner DF and Philipson LH. Insulin Biosynthesis, Secretion, Structure, and Structure-Activity Relationships. In: De Groot LJ, Chrousos G, Dungan K, Feingold KR, Grossman A, Hershman JM, et al., editors. Endotext. South Dartmouth (MA); 2000.
68. Chan SJ and Steiner DF. Insulin through the ages: Phylogeny of a growth promoting and metabolic regulatory hormone. Am Zool. 2000;40 2:213-22.
69. De Meyts P. Insulin and its receptor: structure, function and evolution. Bioessays. 2004;26 12:1351-62.
70. Tokarz VL, Macdonald PE and Klip A. The cell biology of systemic insulin function. Journal of Cell Biology. 2018;217 7:2273-89.

71. Adams MJ, Blundell TL, Dodson EJ, Dodson GG, Vijayan M, Baker EN, et al. Structure of Rhombohedral 2 Zinc Insulin Crystals. Nature. 1969;224 5218:491-&.
72. Lisi GP, Png CYM and Wilcox DE. Thermodynamic Contributions to the Stability of the Insulin Hexamer. Biochemistry. 2014;53 22:3576-84.
73. Blumenthal S. From insulin and insulin-like activity to the insulin superfamily of growth-promoting peptides: a 20th-century odyssey. Perspect Biol Med. 2010;53 4:491-508.
74. Smit AB, van Kesteren RE, Li KW, Van Minnen J, Spijker S, Van Heerikhuizen H and Geraerts WP. Towards understanding the role of insulin in the brain: lessons from insulin-related signaling systems in the invertebrate brain. Prog Neurobiol. 1998;54 1:35-54.
75. Dutertre S, Jin AH, Vetter I, Hamilton B, Sunagar K, Lavergne V, et al. Evolution of separate predation- and defence-evoked venoms in carnivorous cone snails. Nat Commun. 2014;5:3521.
76. Akondi KB, Muttenthaler M, Dutertre S, Kaas Q, Craik DJ, Lewis RJ and Alewood PF. Discovery, Synthesis, and Structure-Activity Relationships of Conotoxins. Chem Rev. 2014;114 11:5815-47.
77. Jin A-H, Muttenthaler M, Dutertre S, Himaya SWA, Kaas Q, Craik DJ, et al. Conotoxins: Chemistry and Biology. Chem Rev. 2019;119 21:11510-49.
78. Olivera BM, Seger J, Horvath MP and Fedosov AE. Prey-Capture Strategies of Fish-Hunting Cone Snails: Behavior, Neurobiology and Evolution. Brain Behav Evol. 2015;86 1:58-74.
79. Safavi-Hemami H, Lu A, Li Q, Fedosov AE, Biggs J, Showers Corneli P, et al. Venom Insulins of Cone Snails Diversify Rapidly and Track Prey Taxa. Mol Biol Evol. 2016;33 11:2924-34.
80. Ahorukomeye P, Disotuar MM, Gajewiak J, Karanth S, Watkins M, Robinson SD, et al. Fish-hunting cone snail venoms are a rich source of minimized ligands of the vertebrate insulin receptor. eLife. 2019;8.
81. Dutertre S, Jin A-H, Vetter I, Hamilton B, Sunagar K, Lavergne V, et al. Evolution of separate predation- and defence-evoked venoms in carnivorous cone snails. Nature Communications. 2014;5 1.
82. Laugesen SH, Chou DHC and Safavi-Hemami H. Unconventional insulins from predators and pathogens. Nat Chem Biol. 2022;18 7:688-97.
83. Menting JG, Gajewiak J, Macraild CA, Chou DH-C, Disotuar MM, Smith NA, et al. A minimized human insulin-receptor-binding motif revealed in a Conus geographus venom insulin. Nature Structural & Molecular Biology. 2016;23 10:916-20.
84. Robinson SD and Safavi-Hemami H. Insulin as a weapon. Toxicon. 2016;123:56-61.
85. Safavi-Hemami H, Lu A, Li Q, Fedosov AE, Biggs J, Showers Corneli P, et al. Venom Insulins of Cone Snails Diversify Rapidly and Track Prey Taxa. Molecular Biology and Evolution. 2016;33 11:2924-34.
86. Southey BR, Amare A, Zimmerman TA, Rodriguez-Zas SL and Sweedler JV. NeuroPred: a tool to predict cleavage sites in neuropeptide precursors and provide the masses of the resulting peptides. Nucleic Acids Res. 2006;34 Web Server issue:W267-72.
87. Safavi-Hemami H, Gajewiak J, Karanth S, Robinson SD, Ueberheide B, Douglass AD, et al. Specialized insulin is used for chemical warfare by fish-hunting cone snails. Proc Natl Acad Sci USA. 2015;112 6:1743-8.
88. Rholam M, Brakch N, Germain D, Thomas DY, Fahy C, Boussetta H, et al. Role of amino acid sequences flanking dibasic cleavage sites in precursor proteolytic processing. The importance of the first residue C-terminal of the cleavage site. Eur J Biochem. 1995;227 3:707-14.
89. Pardos-Blas JR, Tenorio MJ, Galindo JCG and Zardoya R. Comparative Venomics of the Cryptic Cone Snail Species Virroconus ebraeus and Virroconus judaeus. Marine Drugs. 2022;20 2:149.
90. Biggs JS, Olivera BM and Kantor YI. Alpha-conopeptides specifically expressed in the salivary gland of Conus pulicarius. Toxicon. 2008;52 1:101-5.
91. Lavergne V, Harliwong I, Jones A, Miller D, Taft RJ and Alewood PF. Optimized deep-targeted proteotranscriptomic profiling reveals unexplored Conus toxin diversity and novel cysteine frameworks (vol 112, pg E3782, 2015). P Natl Acad Sci USA. 2015;112 45:E6253-E.
92. Gao BM, Peng C, Zhu YB, Sun YH, Zhao T, Huang Y and Shi Q. High Throughput Identification of Novel Conotoxins from the Vermivorous Oak Cone Snail (Conus quercinus) by Transcriptome Sequencing. Int J Mol Sci. 2018;19 12.

93. Escribano O, Beneit N, Rubio-Longás C, López-Pastor AR and Gómez-Hernández A. The Role of Insulin Receptor Isoforms in Diabetes and Its Metabolic and Vascular Complications. Journal of Diabetes Research. 2017;2017:1-12.
94. Denley A, Bonython ER, Booker GW, Cosgrove LJ, Forbes BE, Ward CW and Wallace JC. Structural determinants for high-affinity binding of insulin-like growth factor II to insulin receptor (IR)-A, the exon 11 minus isoform of the IR. Mol Endocrinol. 2004;18 10:2502-12.
95. Sims EK, Carr ALJ, Oram RA, Dimeglio LA and Evans-Molina C. 100 years of insulin: celebrating the past, present and future of diabetes therapy. Nature Medicine. 2021;27 7:1154-64.
96. De Meyts P. Insulin/receptor binding: the last piece of the puzzle? What recent progress on the structure of the insulin/receptor complex tells us (or not) about negative cooperativity and activation. Bioessays. 2015;37 4:389-97.
97. Jumper J, Evans R, Pritzel A, Green T, Figurnov M, Ronneberger O, et al. Highly accurate protein structure prediction with AlphaFold. Nature. 2021;596 7873:583-+.
98. Grimmelikhuijzen CJ and Hauser F. Mini-review: the evolution of neuropeptide signaling. Regul Pept. 2012;177 Suppl:S6-9.
99. Robinson SD, Li Q, Bandyopadhyay PK, Gajewiak J, Yandell M, Papenfuss AT, et al. Hormone-like peptides in the venoms of marine cone snails. Gen Comp Endocrinol. 2017;244:11-8.
100. Ebberink RHM, Smit AB and Vanminnen J. The Insulin Family - Evolution of Structure and Function in Vertebrates and Invertebrates. Biol Bull. 1989;177 2:176-82.
101. UniProt C. UniProt: a worldwide hub of protein knowledge. Nucleic Acids Res. 2019;47 D1:D506-D15.
102. Floyd PD, Li L, Rubakhin SS, Sweedler JV, Horn CC, Kupfermann I, et al. Insulin prohormone processing, distribution, and relation to metabolism in Aplysia californica. J Neurosci. 1999;19 18:7732-41.
103. Krogh A, Brown M, Mian IS, Sjolander K and Haussler D. Hidden Markov models in computational biology. Applications to protein modeling. J Mol Biol. 1994;235 5:1501-31.
104. Laht S, Koua D, Kaplinski L, Lisacek F, Stocklin R and Remm M. Identification and classification of conopeptides using profile Hidden Markov Models. Biochim Biophys Acta. 2012;1824 3:488-92.
105. Mirabeau O, Perlas E, Severini C, Audero E, Gascuel O, Possenti R, et al. Identification of novel peptide hormones in the human proteome by hidden Markov model screening. Genome Res. 2007;17 3:320-7.
106. Gacesa R, Barlow D and Long PF. Machine learning can differentiate venom toxins from other proteins havingnon-toxic physiological functions. PeerJ Comput Sci. 2016;2:e90.
107. Madera M and Gough J. A comparison of profile hidden Markov model procedures for remote homology detection. Nucleic Acids Res. 2002;30 19:4321-8.
108. Potter LR, Yoder AR, Flora DR, Antos LK and Dickey DM. Natriuretic Peptides: Their Structures, Receptors, Physiologic Functions and Therapeutic Applications. cGMP: Generators, Effectors and Therapeutic Implications. Springer Berlin Heidelberg; 2009. p. 341-66.
109. Sangaralingham SJ, Kuhn M, Cannone V, Chen HH and Burnett JC. Natriuretic peptide pathways in heart failure: further therapeutic possibilities. Cardiovasc Res. 2023;118 18:3416-33.
110. Fu H, Zhang J, Cai Q, He Y and Yang D. Pleiotropic Roles of Atrial Natriuretic Peptide in Anti-Inflammation and Anti-Cancer Activity. Cancers. 2022;14 16:3981.
111. Bystrova OA, Parfenov VN and Martynova MG. Atrial natriuretic peptide in the granular cells of the snail heart. Tsitologiia. 2002;44 2:115-9.
112. Koch TL, Robinson SD, Salcedo PF, Chase K, Biggs J, Fedosov AE, et al. Prey Shifts Drive Venom Evolution in Cone Snails. Mol Biol Evol. 2024;41 8.
113. Dutertre S, Jin AH, Kaas Q, Jones A, Alewood PF and Lewis RJ. Deep venomics reveals the mechanism for expanded peptide diversity in cone snail venom. Mol Cell Proteomics. 2013;12 2:312-29.
114. Dutt M, Dutertre S, Jin AH, Lavergne V, Alewood PF and Lewis RJ. Venomics Reveals Venom Complexity of the Piscivorous Cone Snail, Conus tulipa. Mar Drugs. 2019;17 1.

115. Himaya SW, Jin AH, Dutertre S, Giacomotto J, Mohialdeen H, Vetter I, et al. Comparative Venomics Reveals the Complex Prey Capture Strategy of the Piscivorous Cone Snail Conus catus. J Proteome Res. 2015;14 10:4372-81.
116. Jin AH, Dutertre S, Dutt M, Lavergne V, Jones A, Lewis RJ and Alewood PF. Transcriptomic-Proteomic Correlation in the Predation-Evoked Venom of the Cone Snail, Conus imperialis. Marine Drugs. 2019;17 3.
117. Jin AH, Dutertre S, Kaas Q, Lavergne V, Kubala P, Lewis RJ and Alewood PF. Transcriptomic messiness in the venom duct of Conus miles contributes to conotoxin diversity. Mol Cell Proteomics. 2013;12 12:3824-33.
118. Jin AH, Vetter I, Himaya SW, Alewood PF, Lewis RJ and Dutertre S. Transcriptome and proteome of Conus planorbis identify the nicotinic receptors as primary target for the defensive venom. Proteomics. 2015;15 23-24:4030-40.
119. Prashanth JR and Lewis RJ. An efficient transcriptome analysis pipeline to accelerate venom peptide discovery and characterisation. Toxicon. 2015;107 Pt B:282-9.
120. Prashanth JR, Dutertre S, Jin AH, Lavergne V, Hamilton B, Cardoso FC, et al. The role of defensive ecological interactions in theevolution of conotoxins. Mol Ecol. 2016;25 2:598-615.
121. Robinson SD, Li Q, Lu AP, Bandyopadhyay PK, Yandell M, Olivera BM and Safavi-Hemami H. The Venom Repertoire of Conus gloriamaris (Chemnitz, 1777), the Glory of the Sea. Marine Drugs. 2017;15 5.
122. Guo Q, Huang M, Li M, Chen J, Cheng S, Ma L and Gao B. Diversity and Evolutionary Analysis of Venom Insulin Derived from Cone Snails. Toxins. 2024;16 1:34.
123. Bao SJ, Xie DL, Zhang JP, Chang WR and Liang DC. Crystal structure of desheptapeptide(B24-B30)insulin at 1.6 angstrom resolution: Implications for receptor binding. P Natl Acad Sci USA. 1997;94 7:2975-80.
124. Owens DR. New horizons--alternative routes for insulin therapy. Nat Rev Drug Discov. 2002;1 7:529-40.
125. Mirdita M, Schütze K, Moriwaki Y, Heo L, Ovchinnikov S and Steinegger M. ColabFold: making protein folding accessible to all. Nature Methods. 2022;19 6:679-+.
126. Frickey T and Lupas A. CLANS: a Java application for visualizing protein families based on pairwise similarity. Bioinformatics. 2004;20 18:3702-4.
127. Gabler F, Nam SZ, Till S, Mirdita M, Steinegger M, Soding J, et al. Protein Sequence Analysis Using the MPI Bioinformatics Toolkit. Curr Protoc Bioinformatics. 2020;72 1:e108.

Manuscript
Click here to access/download;Manuscript;Manuscript including figures_revised.docx

PeptideMiner – Neuropeptide Discovery across the Animal Kingdom

Helen C. Mendel[1,a], Gene Hopping[1,a], Eivind A. B. Undheim[2], Johannes Zuegg[1], Richard J. Lewis[1], Briony E. Forbes[3], Quentin Kaas[1*], Markus Muttenthaler[1,4*]

1. Institute for Molecular Bioscience, The University of Queensland, Brisbane, Australia

2. Centre for Ecological and Evolutionary Synthesis, Department of Biosciences, The University of Oslo, Oslo, Norway.

3. Discipline of Medical Biochemistry, Flinders Health and Medical Research Institute, Flinders University, Adelaide, Australia.

4. Institute of Biological Chemistry, Faculty of Chemistry, University of Vienna, Vienna, Austria.

[a] Contributed equally to the manuscript.

[*] Corresponding authors: Markus Muttenthaler and Quentin Kaas

Email: m.muttenthaler@uq.edu.au, quentin.kaas@syngenta.com

Author Contributions: H.C.M., Q.K., and M.M. designed the research project. H.C.M., G.H., B.E.F., E.A.B.U., J.Z., and Q.K. performed experiments. H.C.M., Q.K., and M.M. analyzed the data. H.C.M., G.H., and M.M. wrote the paper.

Competing Interest Statement: The authors declare no competing financial interest.

Classification: Biological Sciences; Biophysics and Computational Biology

Keywords: Neuropeptide; venom; transcriptomics; natriuretic peptide; insulin.

Abstract

Neuropeptides represent the largest and most diverse class of cell-to-cell signaling molecules, holding important roles in animal physiology and behavior. They are evolutionarily ancient and widely distributed across the animal kingdom. Although over 200 neuropeptides have been identified, only a small fraction has been functionally characterized. A recognized bottleneck is the lack of effective tools to study their biological roles and therapeutic potential. Interestingly, neuropeptide-like peptides are also found in animal venoms, where they contribute to prey capture or defensive strategies. Mapping neuropeptide families across the animal kingdom is challenging due to their high sequence divergence and short mature peptide sequences. To address this, we developed PeptideMiner, a search tool that employs profile-hidden Markov models (profile-HMM) for family-specific peptide discovery. PeptideMiner was systematically validated and benchmarked against existing methods, demonstrating its superior performance. By applying PeptideMiner to several venom transcriptomes–including 24 previously unpublished datasets–we identified 10 novel natriuretic peptides from distantly related species and 57 novel insulin-like sequences from marine predatory cone snails. Chemical synthesis and structure-activity relationship studies of newly identified conoinsulins at human insulin receptors emphasized the value of our approach in elucidating ligand-receptor interactions and discovering new pharmacological probes and therapeutic leads. PeptideMiner offers a powerful platform for discovering new bioactive peptides and family-specific analogs, accelerating both natural product discovery and evolutionary research.

Introduction

Animal venoms represent an invaluable natural source for developing pharmacological probes and therapeutic agents [1-3]. Comprising intricate and diverse mixtures of bioactive peptides, venoms have evolved to serve the dual purpose of defense and prey immobilization. While venom peptides are primarily recognized for their ability to target ion channels to cause pain or paralysis in prey, they also exhibit efficacy against other target classes, including G protein-coupled receptors (GPCRs) [4], transporters [5], and soluble protein targets such as thrombin [6] and fibrinogen [7]. By identifying, isolating, synthesizing, and pharmacologically characterizing these structurally well-defined venom peptides, researchers have unveiled highly potent and selective molecules that have revolutionized ion channel and pain research [8-10]. Notably, this approach led to the FDA approval of ziconotide (Prialt), a venom peptide derived from the marine predatory cone snail of the genus *Conus*, as a peptide drug for managing severe chronic pain [11]. Another significant example was the discovery of exenatide, a venom peptide derived from the saliva of the Gila monster, which was approved as a peptide drug for treating type II diabetes (Byetta) [12], among others [3].

In addition to the well-studied venom peptides targeting ion channels, there is growing evidence of the presence of neuropeptides in animal venoms, albeit with limited characterization and unclear roles in envenomation. It is hypothesized that neuropeptide signaling systems and their corresponding membrane receptors could represent attractive targets for envenomation due to their crucial physiological roles and remarkable conservation across prey and predators. Notable examples of such venom peptides encompass conopressins (vasopressin, oxytocin) [13], contulakins (neurotensin) [14], conoinsulins (insulin) [15], conorfamides (RF-amide) [16], and natriuretic peptides [17].

Neuropeptides serve as signaling molecules secreted by neurons, orchestrating a wide array of functions encompassing fluid homeostasis, reproduction, appetite control, memory, learning, and complex social behavior [18, 19]. These ancient signaling systems are widely distributed throughout the animal kingdom, with at least 30 neuropeptide signaling systems traceable to a common bilaterian ancestor [20]. Despite their vital physiological roles, our understanding of these signaling systems in animals and humans remains limited, primarily due to a scarcity of pharmacological probes beyond the often nonspecific endogenous ligands required for dissecting the complex pathways and receptor subtypes.

Venoms, containing many diverse neuropeptide-like peptides with unique pharmacological profiles, hold immense promise for transforming neuropeptide research and facilitating the discovery of therapeutic leads for human diseases [19]. The systematic discovery of neuropeptide-like venom peptides is, however, challenging due to a lack of methodologies capable of reliably identifying neuropeptide families across evolutionarily

distant species.

Traditionally, venom research relied on venom collection, bioactivity-guided fractionation, and mass spectrometry. However, recent advances in proteomics, bioinformatics, and nucleotide sequencing [21] have reshaped the approach to studying venoms, culminating in an approach termed integrated venomics [22-25]. Integrated venomics involves generating venom gland transcriptomes that encompass all venom peptide sequences, including their precursors, and subsequently using bioinformatics to match these sequences against the crude venom proteome as well as annotated peptides and proteins in databases such as UniProt and NCBI. Currently, this matching process relies heavily on the NCBI protein-protein Basic Local Alignment Search Tool (BLASTp), which performs well for large homologous sequences but struggles with the short length and high precursor sequence diversity across divergent species characteristic of neuropeptides [26, 27]. Alternative probabilistic models exist, which parameterize complex position-specific models and are expected to be more effective in detecting distant homology [28-30]. One such model is the profile-hidden Markov model (profile-HMM), a probabilistic model of multiple sequence alignments represented as a series of amino acid-emitting *states*, with probabilistic connections between states that account for fully conserved segments, insertions, and deletions [31, 32].

A bioinformatic pipeline or search algorithm capable of reliably identifying homologous neuropeptides across a wide range of species would be of high value, facilitating the systematic mapping and characterization of neuropeptide-like venom peptides. Such an advancement would enhance our understanding of the evolutionary significance and distribution of neuropeptide-like venom peptides, ultimately accelerating the discovery of new pharmacological tools and therapeutic agents.

In this study, we present PeptideMiner, a neuropeptide discovery pipeline that utilizes neuropeptide family-specific profile-HMMs to efficiently identify neuropeptide sequences across different databases and sources. To highlight the application scope of PeptideMiner, we used it to identify new natriuretic and insulin-like peptides from venom gland transcriptomes of a broad range of venomous species, including 24 previously unpublished transcriptomes, and benchmarked it against existing methods of sequence homology searches. Finally, we chemically synthesized and tested newly identified insulin-like venom peptides against the human insulin receptor to demonstrate the translational potential of this new computational discovery pipeline.

Results

PeptideMiner Overview

PeptideMiner is a versatile new tool for neuropeptide discovery that integrates a suite of bioinformatic tools, including HMMER3 [33], SignalP [34], FASTA36 [35], BLAST [36], and SQLite3 [37] to search, filter, and annotate amino acid sequences. It is implemented in Python, a high-level programming language commonly used for bioinformatic pipelines due to its rapid implementation and seamless integration of multiple bioinformatic tools [38, 39]. By employing peptide/protein family-specific profile-HMMs, PeptideMiner is able to efficiently search translated transcriptome or genome sequences for a diverse range of amino acid sequences, including short and diverse (neuro)peptides as well as longer proteins.

Generation of precursor and mature profile-hidden Markov models

Neuropeptide sequences of interest were submitted as a query to the PSI-BLAST (Position-Specific Iterative Basic Local Alignment Tool) against the NCBI non-redundant database [36]. The hits from the first iteration generated a position-specific scoring matrix that was used to search the database for sequences matching the conservation pattern specified by the matrix. The process was iterated 10 times or until no more new sequences were detected. This preliminary training set of sequences was aligned using MUSCLE [40] or ClustalO [41] and trimmed in Jalview [42]. Each initial training set was trimmed to generate two training sets: in one, the sequences were trimmed to the whole precursor peptide, and in the other, to the mature peptide. Each training set was used to build a *precursor*-profile-HMM and a *mature*-profile-HMM using *hmmbuild* from the HMMER 3.0 package [43]. A similar number of reads were observed irrespective of whether the *precursor*-profile- or *mature*-profile-HMM was used, indicating the profile-HMM did not affect the efficiency or depth of the search (Figure S1). For this work, the products of both *precursor*- and *mature*-profile-HMMs were combined, and duplicate product sequences were removed.

PeptideMiner Workflow

To initiate the PeptideMiner workflow (Figure 1), users must first input one or more profile-HMMs of neuropeptide families of interest, as well as the database of amino acid sequences that should be searched as FASTA files. *Hmmsearch* from the HMMER3 package is then used to search the database using the profile-HMMs. *Hmmsearch* results are stored in an SQLite database (Figure S2). The protein-coding sequences (CDS) of the hypothetical neuropeptide precursors are predicted by extracting the sequence between the methionine 'M' start codon and the stop codon or, if there is no stop codon, the end of the contig. SignalP [34] is then used to identify the signal peptides of the predicted CDS and, if present, excised from the precursor sequence to

facilitate mature peptide identification. FASTA36 [35] is used to align all the processed CDS to a manually curated list of known mature peptides, taken from the UniProt database, of the neuropeptide family of interest, followed by a precursor cleavage site prediction using the algorithm from the ConoServer annotation pipeline [44]. The predicted mature peptides for all sequences from the same transcriptome are then compiled, and duplicate mature peptides are removed. BLASTp is then used to annotate the predicted mature peptides by homology using a list of known neuropeptide amino acid sequences.

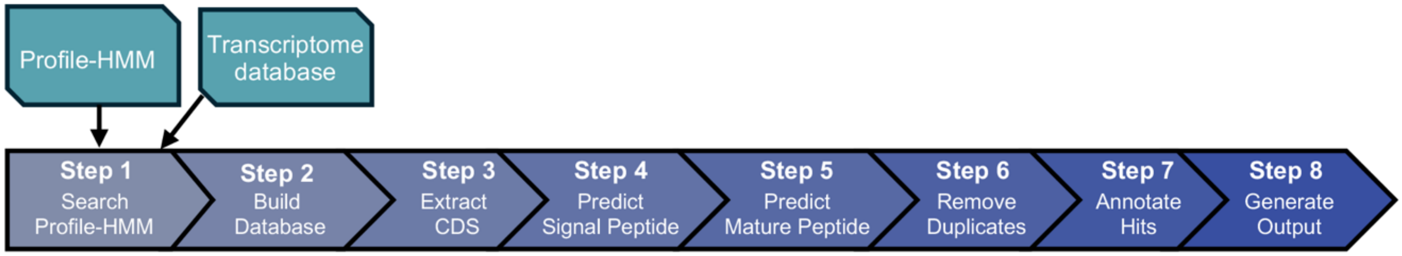

Figure 1. PeptideMiner pipeline. PeptideMiner uses *hmmsearch* in the HMMER 3 package to search through the transcriptomes with the profile-HMMs and saves the output to an SQLite database. The protein-coding sequences (CDS) are extracted from the hits and submitted to SignalP to determine whether a signal peptide is present. Next, mature peptides are predicted and compared to a list of known sequences belonging to the neuropeptide family of interest. The final output consists of a list of precursors, the transcriptomes, predicted mature peptides, and known sequences they are most similar to. The user can specify the minimum CDS length required, the signal peptide cut-off, the minimum length of the signal peptide, the FASTA36 E-value cut-off, and the minimum and maximum length of the mature peptide.

PeptideMiner Performance Evaluation

To assess the pipeline performance, a 10-fold cross-validation analysis was conducted using a test database consisting of 300,000 randomly selected sequences from various eukaryotic species acquired from the UniProtKB database [45]. This database was chosen to provide a comprehensive representation of different species. For the evaluation, the natriuretic peptide and insulin neuropeptide families were used as representative model systems (Figure 2A,B). These neuropeptide families were selected due to their wide-ranging diversity, extensive literature, and presence in vertebrates and invertebrates, making them well-represented in the UniProtKB database. For each neuropeptide family, a negative dataset was created from the dataset by removing all known sequences for the respective neuropeptide family (885 natriuretic peptides and 2,660 insulin sequences).

The pipeline was able to precisely identify hormone peptides and non-hormone peptides, as evidenced by the Matthews correlation coefficient (MCC) of 0.99 for the natriuretic peptide family and 0.93 for the insulin neuropeptide family (Table S1). Moreover, the pipeline was very sensitive, with a true positive rate (TPR) of 0.99 for both peptide families. Notably, no false positives were detected for the natriuretic peptide family, and the false positive rate (FPR) for insulin was extremely low at 1.3 x 10^{-4}.

PeptideMiner was then benchmarked against the widely used sequence similarity search algorithm BLASTp, using the sequences of human natriuretic peptide A and human insulin. Both human precursor proteins of these neuropeptides were queried against the same database employed for the 10-fold cross-validation using protein-protein BLAST (BLASTp, Figure 2, Table S2). PeptideMiner outperformed BLASTp for the insulin peptide family and was marginally better for the natriuretic peptide family. Specifically, BLASTp exhibited an MCC of only 0.76 and a TPR of 0.58, whereas PeptideMiner achieved an MCC of 0.93 and a TPR of 0.99, indicating an overall superior performance of PeptideMiner. Notably, both BLASTp and PeptideMiner demonstrated negligible FPR for both peptide families.

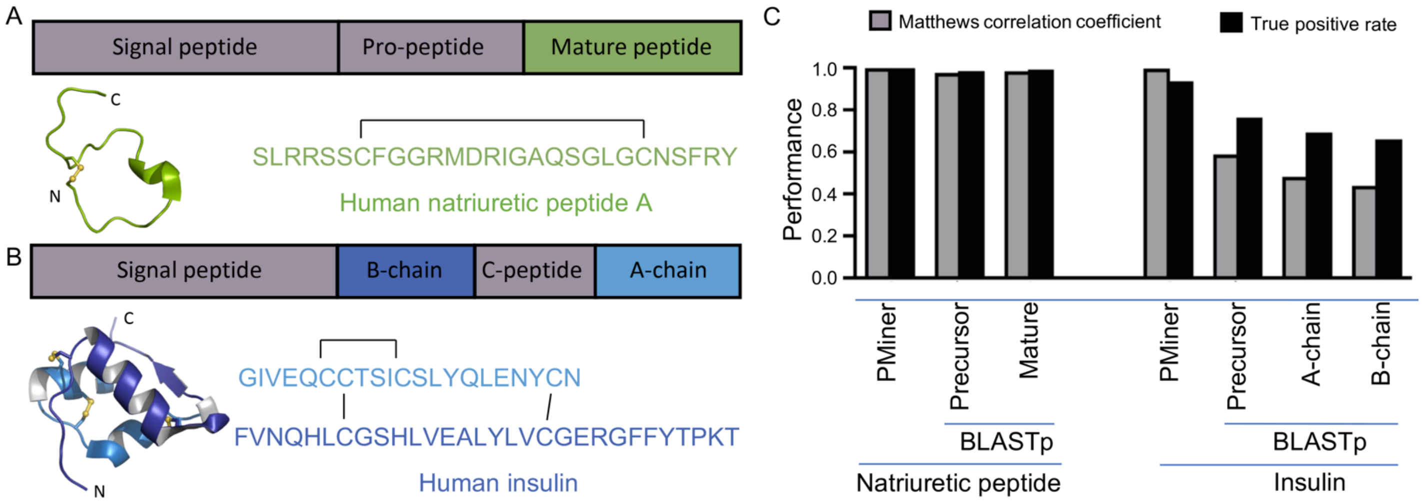

Figure 2. PeptideMiner performance comparison with BLASTp using human insulin and natriuretic peptide A as representative models. **A.** Representation of the precursor protein organization, 3D structure (PDB:7brj), and mature peptide sequence and disulfide bond connectivity of human natriuretic peptide A. **B.** Representation of the precursor protein organization, 3D structure (PDB:3w7y), and mature peptide sequence and disulfide connectivity of human insulin. **C.** Results of the 10-fold cross-validation performance comparison of PeptideMiner (PMiner) versus BLASTp from a library of 300,000 random sequences (Table S2) based on Matthews correlation coefficient and true positive rates. In panels A and B, disulfide bonds are depicted as black lines in the peptide sequence and as yellow balls and sticks in the 3D structures.

Discovery of novel natriuretic peptides

Natriuretic peptides have variable lengths, but all display a 17-amino-acid loop cyclized by a disulfide bridge [46]. These peptides play crucial roles in renal functions, cardiovascular system homeostasis, endothelial cell proliferation, and sympathetic outflow, among other physiological processes [47]. In mammals, there are three main members of the natriuretic peptide family: atrial natriuretic peptide (ANP), B-type natriuretic peptide (BNP), and C-type natriuretic peptide (CNP). NPR-A serves as the primary receptor for ANP and BNP, while NPR-B acts as the primary receptor for CNP. All three natriuretic peptides bind to NPR-C, which primarily functions as a clearance receptor [46]. By contrast, knowledge about invertebrate natriuretic peptide signaling systems is limited. Some evidence for invertebrate natriuretic peptides has been found in the hearts of oysters, blue crabs, and the earthworm *Lumbricus terrestis* [48, 49]. Natriuretic peptide receptors have also been identified in insects, crustaceans, arachnids, mollusks, and even cnidarians, although research on these systems is scarce. Natriuretic peptides are commonly observed in snake venoms, where they affect the prey's cardiovascular system to induce hypotension [50-52]. Furthermore, natriuretic peptides have been observed in the venoms of the platypus *Ornithorhynchus anatinus*, the scorpion *Tityus serrulatus*, the stone fish *Synanceia horrida*, and several lizard species [53-56].

In our search for natriuretic venom peptides across 49 species (Table S3), we identified 11 precursors that displayed the characteristic natriuretic peptide motif C-X_{15}-C (Figure S3). These precursors were identified in snakes (*Micrurus lemniscatus carvalhoi* with three precursors and *Naja Kaouthia* with three precursors), jellyfish (*Chironex fleckeri* with two precursors), leech *(Hirudo nipponia* with one precursor), centipede (*Scolopendra morsitans* with one precursor), and stone fish (*Synanceia horrida* with one precursor) (Figure 3). The natriuretic peptide Sh-NP from *Synanceia horrida* was recently independently identified at the nucleic acid level [56]. The remaining predicted mature peptides have not been previously described from these species and are considered novel putative natriuretic peptides. While the 15 amino acids between the two cysteine residues show relative conservation, the N- and C-terminal tails exhibit considerable variation in length and composition (Figure 3).

Natriuretic peptides in snakes

The three precursors of the South American coral snake *Micrurus lemniscatus carvalhoi* (Mlc) contain two predicted mature natriuretic peptides, Mlc-NP-I and Mlc-NP-II (Figure 3). Natriuretic peptides in snake venoms are hypothesized to contribute to the rapid loss of consciousness of prey [51, 57]. Both exhibit long N-terminal tails (10 residues) and C-terminal tails (14 and 12 residues, respectively), typical of elapid natriuretic peptides. Mlc-NP-I and Mlc-NP-II are highly similar to known natriuretic peptides Mc-NP (both

98% identity) from the painted coral snake *Micrurus corallinus* and the Uruguayan coral snake *Micrurus altirostris* Ma-NP (75% and 95% identity, respectively) [58, 59]. Mlc-NP-I differs only by one amino acid (Gly^{10} vs. Cys^{10}; residue numbering indicates the position within the conserved cysteine residues for ease of comparison) from Mc-NP. Gly^{10} is highly conserved, and the role and impact of the cysteine substitution in Mc-NP is unclear.

In the case of the monocled cobra *Naja kaouthia* (Nk), the three precursors are predicted to produce two mature peptides Nk-NP-I and Nk-NP-II, with only Nk-NP-II being previously described (Figure 3) [60]. Similar to the Mlc-NPs, Nk-NP-I has a five-residue N-terminal tail and a 16-residue C-terminal tail. Interestingly, the C-terminal tail contains two additional cysteine residues in a C-X_1-C pattern, suggesting a natriuretic peptide with an additional disulfide bond. The significance of this is not yet known. By contrast, Nk-NP-II has a short N-terminal tail (five residues) and no C-terminal tail, which is more typical of CNPs such as those found in Viperid snake venom [51, 61]. Nk-NP-II shares an identical sequence to a transcript annotated as waglerin peptide 1, identified in the venom transcriptome of the Bornean-keeled green pit viper, *Tropidolaemus subannulatus* [60]. The waglerins are lethal peptides identified in the venom of *Tropidolaemus wagleri* [62] targeting nicotinic acetylcholine receptors [63]. This transcript contains two peptides with the characteristic Cys-X_3-Cys motif of the waglerin peptides in addition to a natriuretic peptide Cys-X_{15}-Cys motif, identified by PeptideMiner. The encoding of waglerins and CNPs as multi-domain precursor proteins has previously been observed in viperid venom [64], but was not identified in this particular elapid transcript [60]. Nk-NP-II also displays high similarity to the Okinawa habu pit viper *Protobothrops flavoviridis* Pf-CNP (91% identity) but has a Met^2Gly and a Leu^9His substitution, removing a positive charge. Nk-NP-II is the first reported CNP observed in an elapid venom.

Natriuretic peptides in jellyfish

Two natriuretic peptide precursors were identified in the jellyfish *Chironex fleckeri* (Cf), which contain a different but closely related predicted mature natriuretic peptide: Cf-NP-I and Cf-NP-II (Figure 3). These venom peptides exhibit less than 50% identity with any known natriuretic peptides but share several conserved residues in the C-X_{15}-C motif, including Ile^6, Asp^7, Arg^8, Ile^9, Ser^{13}, Leu^{15} and Gly^{14}. Notably, neither Cf-NP-I nor Cf-NP-II has a predicted signal peptide, and alignment with other natriuretic peptides suggests that these are partial precursors (Figure S3). This discovery marks the first evidence of a natriuretic peptide in a Cnidarian. Previous genome annotation identified natriuretic peptide receptors in a single species in this phylum, although this has yet to be confirmed at the protein level [65].

Natriuretic peptides in leeches

A single natriuretic peptide precursor was discovered in the leech *Hirudo nipponia*, which gives rise to a predicted 42-amino-acid-long natriuretic peptide, Hn-NP-I. Although Hn-NP-I has low similarity to known natriuretic peptides, it contains five of the most conserved amino acids within the C-X_{15}-C motif, including Phe^{2}, Arg^{8}, Ile^{9}, Gly^{10} and Ser^{13}, and we therefore consider this a natriuretic peptide-like sequence, representing the first of its kind in the salivary gland of an annelid. While annelids possess the natriuretic peptide signaling system, no endogenous ligands have been reported [49].

Natriuretic peptides in centipedes

In the centipede *Scolopendra morsitans*, a single natriuretic precursor was identified, giving rise to a predicted 27-residue-long natriuretic peptide, Sm-NP-I. Of the 15 amino acids within the C-X_{15}-C motif, 7 are conserved in Sm-NP-I. Furthermore, Sm-NP-I possesses a C-terminal tail but lacks an N-terminal tail. This marks the first observation of a natriuretic peptide in the venom of a centipede. Notably, natriuretic peptides in arthropod venoms are rare, with Ts-NP from the scorpion *Tityus serrulatus* being the only previously reported arthropod venom natriuretic peptide [61]. Sm-NP-I and TsNP exhibit notable differences, with their mature peptides sharing an observed identity of 55% [53]. Unlike Sm-NP, TsNP lacks a C-terminal tail but possesses an N-terminal tail [61]. In humans, residues important for binding NP receptors lie within the intramolecular ring formed by the disulfide bond. However, in human ANP, the C-terminal tail is additionally required for binding to NPR-A. Thus, the N- and C-terminal tails of natriuretic peptides could be important for modulating binding and selectivity.

A

Species	Name	Mature natriuretic peptide	Length	UniProt
Homo sapiens	ANP	----SLRRSSCFGGRMDRIGAQSGLGCNSFRY------------	28	P01160
	BNP	-SPKMVQGSGCFGRKMDRISSSSGLGCKVLRRH-----------	32	P16860
	CNP	------LSKGCFGLKLDRIGSMSGLGC-----------------	21	P23582
Micrurus lemniscatus carvalhoi	**Mlc-NP-I**	GLAKEALGDGCFGQRIDRIGNVSGMGCNHVRTDPAPTALAR---	41	
	**Mlc-NP-II**	GLAKEALGEGCFGNRIDRIGDVSGMGCNHKTPAPKAPLR-----	39	
Naja kaouthia	**Nk-NP-I**	-----GPPDGCFGLKLDHIGTMSGLGCNGGSK-PIPTLTPCGCL	38	
	**Nk-NP-II**	-----AGGKGCFGLKLDRIGSTSGLGC-----------------	38	
Scolopendra morsitans	**Sm-NP-I**	---------GCAGTSLDRIGISSRQLCVSPTGICTV--------	27	
Hirudo nipponia	**Hn-NP-I**	IEVDEVGSDGCFSEELVRIGSWSDILCGMENLFKGLRAKLVG--	42	
Chironex fleckeri	**Cf-NP-I**	------MSSTCRNEFIDRISNTSMLGCSCFKSSLNAMK------	32	
	**Cf-NP-II**	------MSSTCRNEFIDRISNTSILGCSCFKSSLNAMK------	32	
Synanceia horrida	**Sh-NP**	--GASTSRSGCFGHKMDRIGTISGMGC-----------------	25	
Micrurus altirostris	Ma-NP	GLAKEALGEGCFGNRIDRIGDVSGMGCNRRTPAPKAPLR-----	39	F5CPE8
Micrurus corallinus	Mc-NP	GLAKEALGDGCFGQRIDRICNVSGMGCNHVRTDPAPTALAR---	41	P79799
Protobothrops flavoviridis	Pf-CNP	-------GKGCFGHKLDRIGSTSGLGC-----------------	20	P0C7P5
Bothrops jararaca	Bj-CNP	-----GAAKGCFGLKLDRIGTMSGLGC-----------------	22	Q6LEM5
Lachesis muta muta	Lmm-CNP	-----RVGDGCFGLKLDRIGSMSGLGC-----------------	22	Q27J49
Tityus serrulatis	TsNP	------KLSGCFGFKLDRIGTMSGLGC-----------------	21	P0DMD6

B

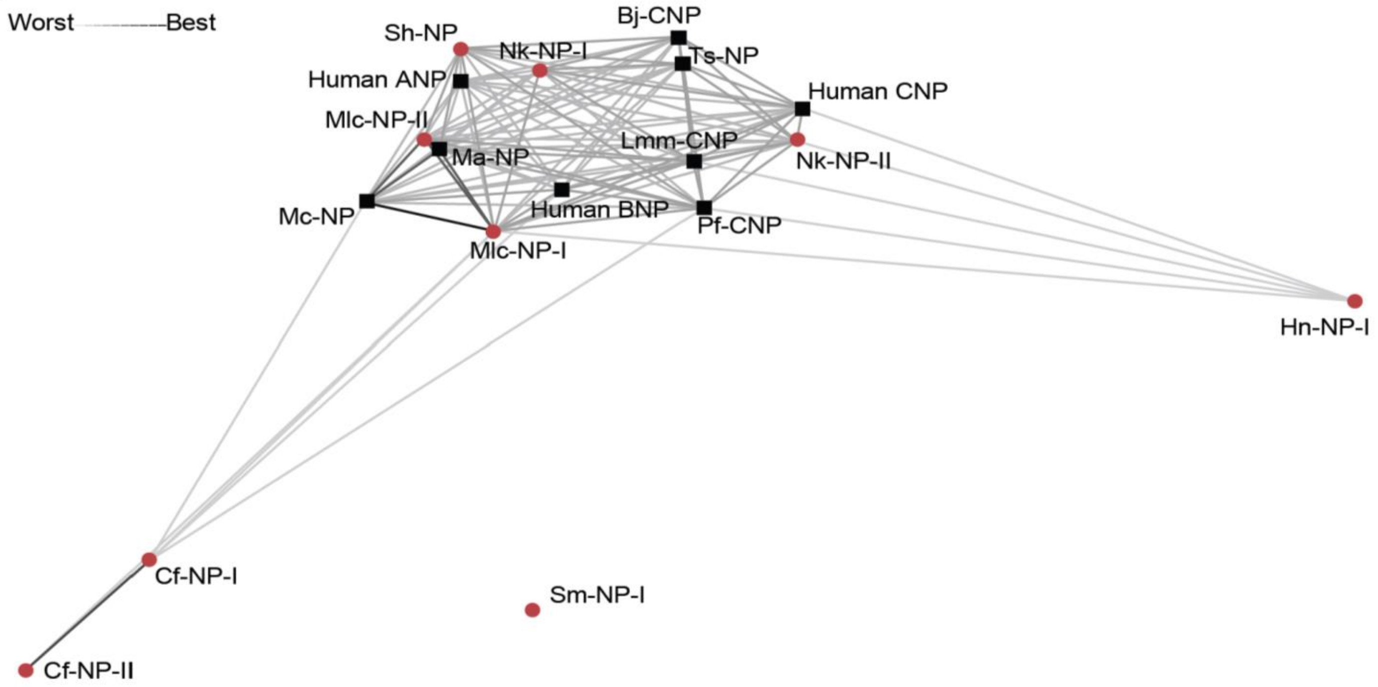

Figure 3. Sequence alignment and clustering of mature natriuretic peptides identified in venom gland transcriptomes aligned with human atrial natriuretic peptide (ANP). A. In the sequence alignment, cysteine residues are shaded yellow, N-terminal tail residues are colored green, and C-terminal tail residues gray. Novel natriuretic peptides identified in this study are bolded. Selected published natriuretic peptide sequences are shown in the lower panel for comparison. Sh-NP was novel when the study was conducted, but

was recently independently identified [56], validating our approach. B. Pairwise blast clustering of natriuretic peptides with human ANP, BNP, and CNP. Sequence similarity is denoted by line length and thickness. Human ANP sequences and previously reported NPs are represented as black squares, and novel sequences are red colored circles.

Discovery of novel Conus venom insulins

Insulin belongs to the insulin superfamily, which is highly conserved throughout the animal kingdom [66]. In humans, insulin is produced by the pancreatic β-cells in the Islets of Langerhans and plays a crucial role in regulating glucose homeostasis by facilitating glucose uptake into liver, fat, and skeletal muscle cells and suppressing gluconeogenesis in the liver [67, 68]. Insulin comprises two peptide chains (A and B) connected by two interchain disulfide bonds (C^7_A-C^7_B and C^{20}_A-C^{19}_B) (Figure 2).

The 21-residue A-chain has an additional intrachain disulfide bond (C^6_A-C^{11}_A) and displays an α-helical secondary structure on the N-terminal and C-terminal ends. The 30-residue B-chain features a central α-helix and a β-strand [67] that together form a characteristic and evolutionarily conserved 3D structure (Figure 2) [69]. Mature insulin is derived from a single-chain precursor protein with a signal peptide, followed by the A-chain, C-peptide, and B-chain [67]. After translation in the rough endoplasmic reticulum as preproinsulin, the signal peptide is cleaved, and proinsulin is folded and sorted into immature secretory granules for C-peptide excision and subsequent processing into mature insulin [70]. At higher micromolar concentrations, insulin dimerizes and forms hexamers in the presence of zinc, enhancing its stability and preventing fibrilization [71, 72]. Vertebrate-like conoinsulins share a cysteine framework similar to that of human insulins [73]. Conversely, molluskan insulin peptide (MIP)-like conoinsulins possess a cysteine motif resembling endogenous mollusk insulins with three A-B interchain disulfide bonds and one A intrachain disulfide bond (Figure 4) [74].

Marine predatory cone snails (genus *Conus*) are a group of venomous snails that utilize their venom for predation and defense [75-77]. The diet of cone snails varies and is broadly classified as fish-hunting (piscivorous), mollusk-hunting (molluscivorous), or worm-hunting (vermivorous). Fish-hunting cone snails have developed three distinct hunting strategies: “taser and tether”, “net engulfment”, and “strike and stalk” [78]. Their venoms comprise a wide diversity of bioactive peptides, and insulin-like peptides have been observed in several species [15, 79-85]. In this study, PeptideMiner was used to search 23 previously unpublished venom transcriptomes for new insulin homologs. We searched 20 venom duct transcriptomes (including two *C. imperialis* and two *C. tulipa* transcriptomes), two nerve ring transcriptomes (*C. catus and C.*

textile), and one salivary gland transcriptome (*C. geographus*) across 18 cone snail species (Table S3). After removing incomplete precursors, PeptideMiner identified 87 unique precursors from 16 of the 18 studied cone snail species (Figure 4, Figures S4-7). Neither *C. rattus*, nor *C. imperialis*, both worm hunters, returned any conoinsulin precursors. The predicted mature peptides (connected B- and A-chains without the C-peptide) were manually assessed and cross-checked with cleavage sites predicted by NeuroPred [86] and identified sequences of conoinsulins at the protein level (e.g., Con-Ins G1, G3 [87], G121 [75] from *C. geographus*). Insulin precursor sequences are typically cleaved at dibasic cleavage sites (Arg-Arg or Lys-Arg) or, in some cases, at single basic sites (Arg) [88].

To assess whether any of the 87 conoinsulin precursors were novel, we compared them to known conoinsulin and insulin sequences obtained from UniProt and relevant literature. A BLASTp search revealed that only 15 of the 87 precursors were previously annotated, including 8 MIP-like insulins (Con-Ins Pl1, M2, Tx1, Tx2, Va1, Pl174, Ebr1a and Ebr1b) and 7 vertebrate-like insulins (Con-Ins G1b, G3, G121 Tu478, Tu479, Tu304, Tu073), yielding 72 novel conoinsulin precursors. Removal of identical sequences in the predicted B- and A-chains from these 72 precursors resulted in 57 novel and unique mature conoinsulins across 14 species, including 5 vertebrate-like, 33 MIP-like, and 19 'other' conoinsulins that contained an odd number of cysteine residues (Table S4).

Of the 87 conoinsulin sequences discovered, 23 were vertebrate-like conoinsulins with the same three disulfide bond pattern as observed in human insulin (Figure S4). Vertebrate-like precursors from fish-hunters were predominantly identified in *C. geographus* and *C. tulipa*. 45 conoinsulins had 8 cysteine residues and were therefore categorized as MIP-like conoinsulins (Figure S5). 19 conoinsulins could not be classified as either vertebrate or molluskan conoinsulins as they featured an odd number of cysteine residues in the A- or B-chain (Figure S6), the significance of which is not known at present.

A single distinct conoinsulin precursor was identified in the salivary gland transcriptome of *C. geographus*, which we named G-SG-1 (Figure 4). It is similar to the MIP-like venom conoinsulins Im1 and *C. ebreaus* hormone insulin-related peptide [89]. It is distinct from both *C. geographus* signaling insulin (NR-G1) [85] and all other *C. geographus* venom conoinsulins with low precursor sequence conservation apart from the cysteine framework (Figure 4) [79]. G-SG-1 is the first conoinsulin identified from the salivary gland of a cone snail. Cone snail venom peptides were previously identified in the salivary glands of several species, including *C. pulicarius*,[90] *C. episcopatus*,[91] and *C. quercinus*.[92] Their role in envenomation remains unclear, but they have been proposed to function endogenously or enhance venom potency [92].

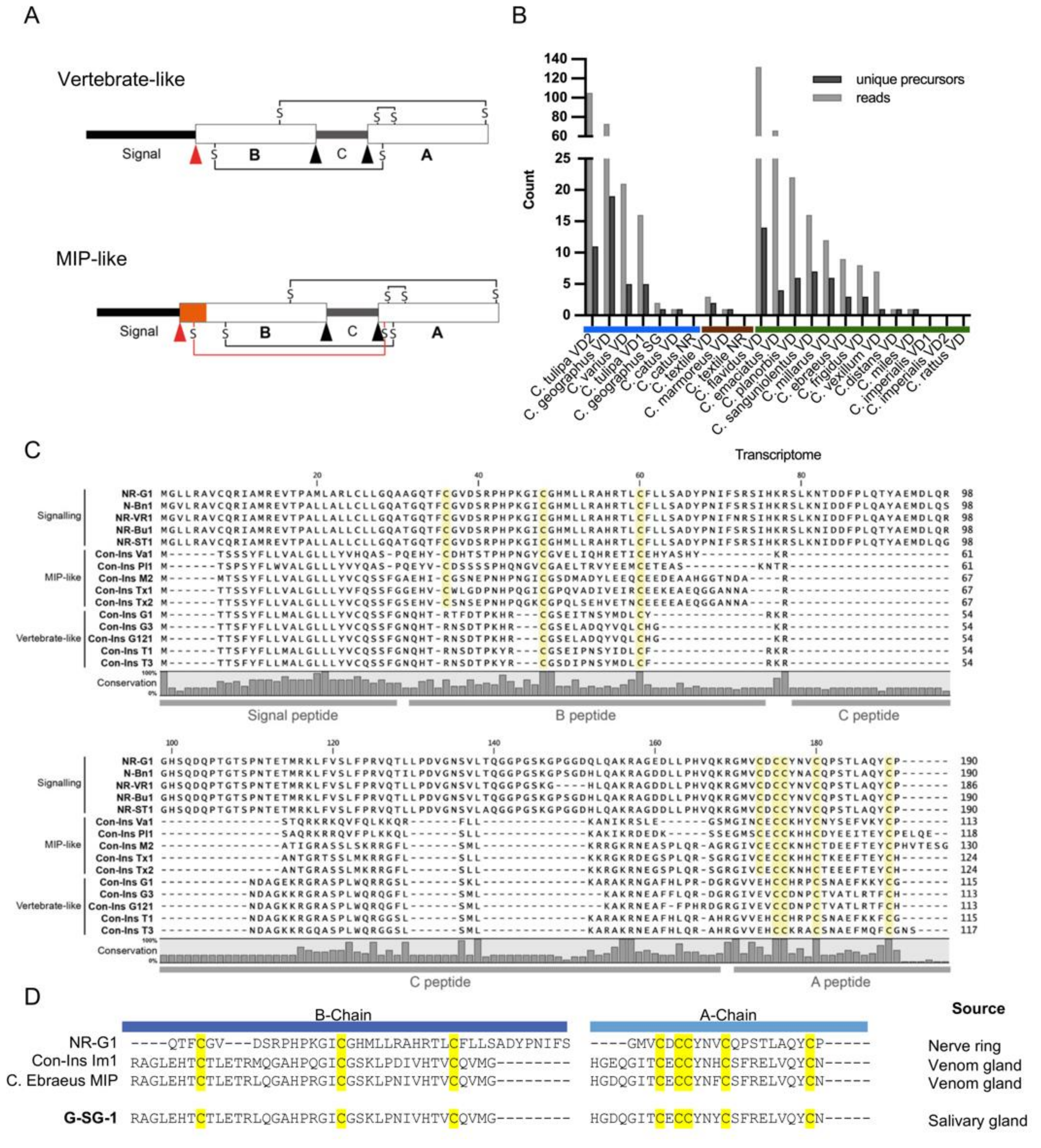

Figure 4. Discovery of novel conoinsulins. A. Schematic representation of the secondary structure of vertebrate-like and MIP-like conoinsulin precursors. All sequences contain a signal peptide, B-chain, C-peptide, and A-chain. Predicted proteolytic cleavage sites are indicated with black arrows, and predicted processing sites for removal of the signal sequence are indicated with red arrows. The orange box indicates amino acid insertions, including the extra cysteine residue forming the 4th disulfide bond in MIP-like insulins.

B. The total number of different reads per transcriptome (gray) and the number of unique precursors (black). Transcriptome tissues: VD, venom duct; SG, salivary gland. Prey of species is denoted by colored bars: fish-hunting (blue); mollusk-hunting (brown); worm-hunting (green). **C.** Sequence alignment of endogenous cone snail insulin precursors with selected venom insulins (for the complete list, see Figures S4-6). Cysteine residues are highlighted in yellow; the level of conservation is illustrated below the alignment. **D.** Sequence alignment of non-venom conoinsulins. A- and B-chains are labeled, and cysteine residues are shaded yellow. Sequences discovered in this work are bolded, and the tissue of their discovery is indicated on the right.

Pharmacological characterization of vertebrate-like conoinsulin at the human insulin receptor

As a proof-of-concept of using PeptideMiner to discover new venom neuropeptides with pharmacological or even therapeutic value, we synthesized several vertebrate-like conoinsulins using solid-phase peptide synthesis (SPPS), folded them, and tested them at the human insulin receptor-B (hIR-B), the most pharmaceutically relevant human insulin receptor subtype [93, 94]. A competition binding analysis to hIR-B of selected conoinsulins against Eu-labelled insulin was carried out. Results are expressed in IC_{50}, reflecting the concentration of the competing ligand (Con-Ins) displacing 50% of the specific binding of Eu-insulin.

We focused on vertebrate-like conoinsulins G3c, G4b, and G7 due to their overall structural similarity to human insulin and insulin's clinical significance in treating diabetes [95]. Con-Ins G4b and G7 bound to hIR-B with nanomolar affinities (IC_{50} 35.4 nM and 124.2 nM, respectively), while no competition with human insulin was evidenced for Con-Ins G3c at up to 3 μM (Figure 5). The IC_{50} of Con-Ins G4b (35.4 nM) was similar to Con-Ins G3 (46.8 nM [80]. Con-Ins G4b differs from Con-Ins G3 by a six-residue C-terminal extension of the B-chain, akin to human insulin, albeit with low sequence similarity. This extension has little effect on hIR-B binding. The main difference between Con-Ins G7 and Con-Ins G3 is a five-residue N-terminal extension, also displayed by human insulin. Remarkably, Con-Ins G7 showed potent hIR-B binding yet no improvements in affinity compared to human insulin. Notably, Con-Ins G3c differs from Con-Ins G3 only at a single residue in the B-chain (Gly_B^{10}Val), which completely abolishes hIR-B binding. This result highlights the importance of Gly_B^{10}, which is highly conserved within venom conoinsulins as well as across species in endogenous insulins and is identified as part of the hIR S1 binding site [96].

The three-dimensional structures of the three tested conoinsulins were predicted using AlphaFold2 [97] and compared to the crystal structure of human insulin (Figure 5B). Cα–root mean square deviation (Cα–RMSD) for all residues vs the human insulin crystal structure (PDB: 3wy7) were 0.8, 1.7, and 1.0 Å for G3c, G4b, and

G7, respectively (Cα–RMSD for the AlphaFold2 predicted human insulin structure was 0.5 Å for comparison). These three conoinsulins are predicted to adopt the insulin-like fold, the most notable deviation being the elongation of the B-chain termini.

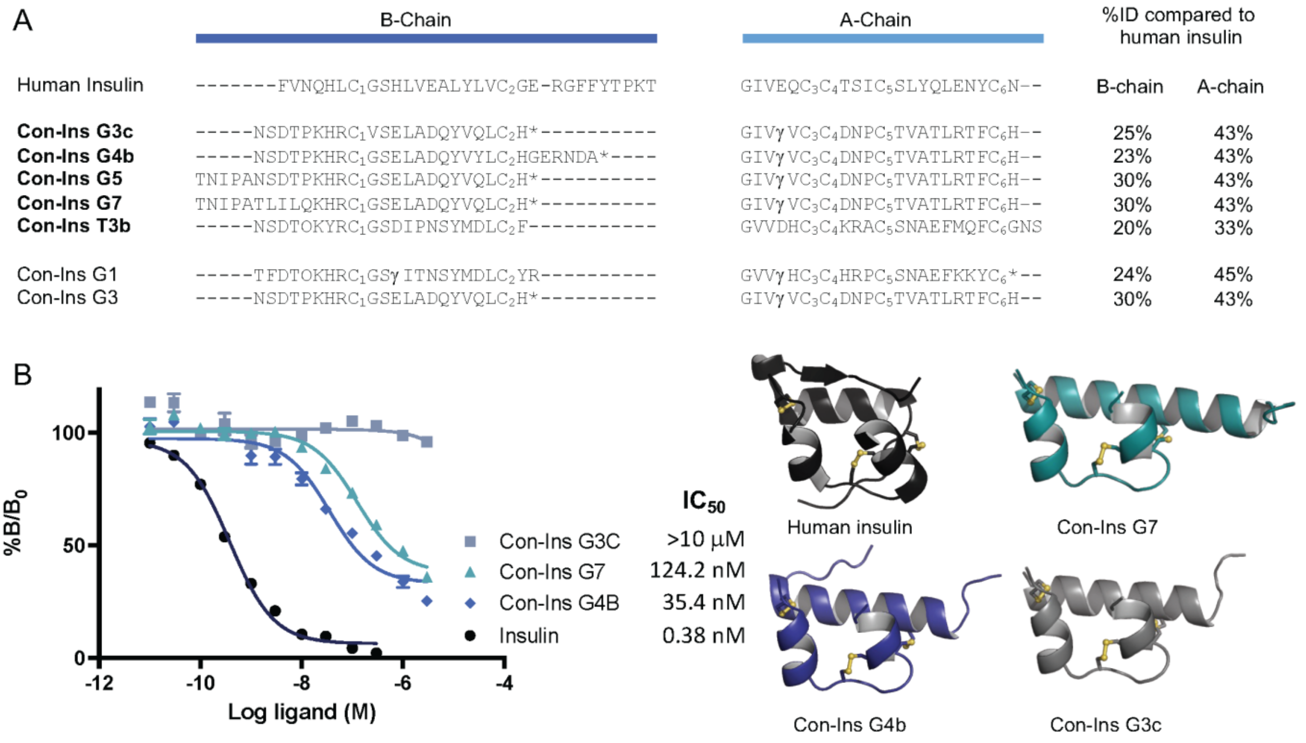

Figure 5. Sequence alignment and relative binding affinity of newly identified and synthesized conoinsulins to the human insulin receptor B (hIR-B). A. Amino acid sequence alignment of the A- and B-chains of selected conoinsulins and human insulin. **B.** Competition binding affinity to hIR-B between conoinsulins and Eu-labelled human insulin, and AlphaFold2 predicted three-dimensional structures of Con-Ins G7 (green), Con-Ins G4b (blue), and Con-Ins G3c (gray) are presented next to the human insulin crystal structure (black, PDB:3w7y). In panel A, the percentage amino acid sequence identity (%ID) to human insulin calculated by ClustalO [41] is displayed on the right for both the B- and A-chains. Predicted post-translational modifications are highlighted in red: O, 4-hydroxoproline; γ, 4-carboxyglutamate; *, C-terminal amidation. The cysteine connectivity for all sequences is C_1-C_4; C_2-C_6; C_3-C_5. Sequences discovered in this work are in bold. In panel B, results are expressed as a percentage of Eu-insulin bound without competing ligands (%B/B_0). Plotted values represent means +SEM (n=3, each comprising three technical replicates). IC_{50} is the concentration of the competing ligand (Con-Ins) displacing 50% of the specific binding of Eu-insulin.

Discussion

Neuropeptides are often referred to as the signaling molecules of life due to their ancient character and pivotal roles in various biological processes, including neurotransmission, inter- and intra-cellular signaling, and regulating complex behaviors. Their involvement spans a wide array of physiological functions, including pain perception, feeding behavior, stress and fear response, cardiovascular functions, fluid balance, and reproduction, amongst many others [19, 20, 98]. Their evolutionary conservation across diverse species underscores this fundamental physiological importance, and their involvement in numerous diseases has attracted substantial scientific interest.

The high degree of conservation and physiological significance of neuropeptides may also explain the presence of neuropeptide-like peptides in venoms, where they presumably contribute to prey capture or predator deterrence [99]. The unexplored diversity of neuropeptides within venoms presents a unique opportunity to discover novel pharmacological tools and therapeutic leads. However, the efficient identification and mapping of neuropeptides using traditional search algorithms has been challenging due to the short lengths and high sequence variations of these signaling molecules [26]. We, therefore, developed PeptideMiner, a highly efficient peptide search tool capable of overcoming these bottlenecks and facilitating the identification and mapping of neuropeptides across a wide range of species and databases.

PeptideMiner, an enhanced computational tool to efficiently search for neuropeptides

PeptideMiner is a highly versatile, robust, Python-based pipeline that harnesses peptide family-specific profile hidden Markov models (profile-HMMs) to effectively search translated transcriptomic, genomic, and proteomic data for neuropeptides or peptides in general. PeptideMiner demonstrated exceptional performance with remarkably low false-positive and false-negative rates when tested against representative neuropeptide model systems, surpassing the widely utilized BLASTp method. Notably, PeptideMiner exhibits clear advantages, particularly evident in the insulin family, attributed to its extensive and divergent nature, with over 2,000 variants across the animal kingdom, including diverse mature insulins featuring additional interchain disulfide bonds in certain species [100, 101]. The methodological disparities and existing limitations of BLASTp in this context are anticipated to be intensified by the ongoing influx of new sequence data from diverse species, spurred by the advancements and widespread adoption of 'omics' technologies [79, 102]. Conversely, PeptideMiner can handle large datasets, sequence variations, and distant homology, thereby enabling more accurate and comprehensive neuropeptide identification across species. PeptideMiner also supports simultaneous searches across multiple species and is compatible with any peptide/protein database. Importantly, PeptideMiner is open access, written in Python, and integrates publicly available bioinformatic

tools, ensuring ease of access and user-friendliness for the broader scientific community. While primarily developed to advance the mapping of neuropeptide families across species and expedite the discovery of neuropeptides with sequence similarity and unique pharmacology or therapeutic potential, the open-access and user-friendly nature of PeptideMiner encourages its adoption by the scientific community for other applications.

The concept of profile-HMMs in computational biology emerged in the mid-90s [103] and has more recently found applications in studies involving peptides, including neuropeptides [104, 105]. For example, profile-HMMs were employed in predicting and classifying the 62 conotoxin superfamilies [104]. In another instance, a profile-HMM based on biological processing signals of neuropeptides, encompassing the signal peptide, pro-peptide cleavage site, and extracellular peptide features, was utilized to identify novel bioactive peptides, including neuropeptides, in the human proteome [105]. One further example is the use of small profile-HMMs known as ‘tox-bits’. Combinations of 2-3 ‘tox-bits’ could accurately discern toxins from non-toxin sequences in a machine-learning model [106]. These approaches, however, lack specificity to neuropeptides and do not all involve sequence homology.

By contrast, PeptideMiner specifically targets neuropeptides by constructing profile-HMMs using known precursor or mature peptide sequences from the neuropeptide family of interest. This neuropeptide-specific approach improves identification accuracy. Additionally, the neuropeptide sequences selected to build the seed alignment for the profile-HMM are not restricted to a specific taxonomic group, facilitating the detection of neuropeptide analogs with distant homology across evolutionarily diverse species. This capability is particularly suited for investigating neuropeptide evolution, identifying neuropeptides in uncharacterized species, and discovering highly divergent neuropeptides, such as those present in tissues with a high mutation rate, like venoms.

To ensure an effective search, thoughtful consideration needs to be given when constructing profile-HMMs [30, 103]. Manual curation of the multiple sequence alignments is essential for profile-HMM construction [107], which can then be publicly shared. Neuropeptide families with a limited number of known peptides or peptides from a limited taxonomic range are at a disadvantage compared to larger families present in many taxonomic lineages. It is, therefore, important to regularly update the seed alignments and profile-HMMs with novel sequence information, a process that can be automated.

In addition to the insulin and natriuretic peptide profile-HMMs discussed and utilized in this study, we have included three other neuropeptide profile-HMMs for the neurohypophyseal, tachykinin, and somatostatin families in the open-access PeptideMiner platform to facilitate profile-HMMs creation. While there are

parameters of HMMER3 that can be adjusted for profile-HMM construction (*hmmbuild*) and searching profile-HMMs against the database (*hmmsearch*) [33], PeptideMiner already demonstrates exceptional performance, and further investigations into these parameters are not expected to enhance performance substantially.

Natriuretic peptide discovery expanded to new species

Natriuretic peptides regulate fluid balance, blood pressure, and cardiovascular homeostasis [108, 109]. In humans, ANP and BNP are predominantly produced in the heart's atria (while CNP is more widely expressed in endothelial cells) and released in response to elevated blood volume and pressure, causing vasodilation. They also promote urine production and inhibit sodium reabsorption, ultimately facilitating natriuresis [108]. Furthermore, natriuretic peptides exhibit anti-inflammatory [110] and anti-fibrotic properties, rendering them promising candidates for treating heart failure, hypertension, and kidney diseases [109].

Natriuretic peptides have also been identified in animal venoms, likely owing to their cardiovascular effects for defense and prey capture. They are particularly abundant in snakes but have also been observed in the venoms of lizards, stonefish, platypuses, and scorpions [51, 53-56]. This study expanded our understanding of natriuretic peptide distribution in venomous animals, confirming that they are not limited to vertebrate venoms and underscoring their presence in diverse evolutionary lineages. Notably, natriuretic peptides were detected in the venom glands of the centipede *S. morsitans*, as well as in two previously unexplored phyla, namely the annelid *H. nippona* and the cnidarian *C. fleckeri* species. The identification of natriuretic peptides in the tentacles of *C. fleckeri* holds significance, as it provides further evidence of their presence in cnidarians beyond the genome annotation of the putative natriuretic peptide A receptor in the cnidarian *Thelohanellus kitauei* [65]. The identification of Cf-NP-I and Cf-NP-II supports the presence of natriuretic peptides in Cnidaria, opening the possibility for convergence, or that the ancestral gene encoding the natriuretic peptide precursor was present in the last common ancestor of cnidarians and bilateral animals 600-700 million years ago [98]. No natriuretic peptides were identified in the transcriptomes of mollusk venom glands or salivary glands, despite evidence of the natriuretic peptide signaling system in gastropods such as *Crassostrea virginica* and *Helix pomatia* [48, 111].

Conoinsulin discovery expands their diversity and underscores their role in envenomation

Insulin is a crucial peptide hormone regulating glucose levels and maintaining metabolic balance in humans. Its primary function lies in facilitating glucose uptake into cells, promoting its utilization for energy production and storage. Dysregulation of insulin production or impaired insulin function can lead to metabolic disorders, notably diabetes [67, 68]. Understanding the mechanisms underlying insulin action and the molecular interactions of ligands with the main human insulin receptor, hIR-B, is essential for developing more effective

and safer therapeutic strategies for managing diabetes and related conditions.

Venoms represent a new natural source for insulin-like peptides, and particularly, the venom of the marine predatory cone snail seems rich in venom insulins, playing a role in prey capture [15, 84, 85]. There is evidence for prey-specific selection pressures in shaping the variation of peptides found in cone snail venom [112], and the presence of vertebrate-like conoinsulins in piscivorous cone snails supports their use for fish capture. These vertebrate-like conoinsulins are similar to fish insulins, bind to zebrafish insulin receptors, and can induce hypoglycemic shock to facilitate prey capture [15]. This is further supported by the complete absence of vertebrate-like conoinsulins in the venom of molluscivorous or vermivorous cone snails. By contrast, mollusk hunters produce MIP-like conoinsulins characterized by an additional disulfide bond in the B-chain, suggesting an evolutionary adaptation for their specific molluskan target prey [15, 85] (Figure 6).

Cone snails employ their venom for defense as well; indeed, they can adjust their venom composition based on intended use, whether for predation or defense [75]. Cluster analysis alone cannot clearly distinguish between worm and molluskan insulins [79] (Figure 6). Piscivorous cone snails also produce MIP-like conoinsulins. Two fish-hunting species, *C. catus* and *C. varius*,, were found only to produce MIP-like conoinsulins, while *C. geographus* produces both MIP-like and vertebrate-like conoinsulins, albeit with vertebrate-like conoinsulins being predominant in the latter case (Figure 6, Figure S4). It is not well understood whether MIP-like conoinsulins are used for prey purposes or to defend against molluscan predators such as octopuses. However, based on the cluster analysis, there are no apparent similarities with octopus insulin, indicating that they may be used for prey capture or to defend against other mollusk-hunting cone snails (Figure 6).

The application of PeptideMiner substantially expanded the known diversity of conoinsulins, unveiling numerous new conoinsulin variants, thereby underscoring their role and significance in envenomation. Through the analysis of 18 cone snail species, we identified 78 novel insulin precursors and 59 novel mature conoinsulins (Figures S4-6), in addition to the previously identified MIP-like Con-Ins Pl1, M2, Tx1, Tx2, Va1, Pl174, and vertebrate-like Con-Ins G1b, G3, G121 Tu478, Tu479, Tu304, Tu073. This includes the documentation of conoinsulins in *C. catus, C. miles, C. distans, C. ebreaus, C. emaciatus, C. flavidus, C. miliaris, C. sanguinolentus* and *C. vexillum* for the first time. PeptideMiner also revealed several novel conoinsulin precursors and mature peptides in previously annotated transcriptomes of *C. catus*, *C. geographus*, *C. miles*, *C. planorbis*, *C. tulipa,* and *C. vexillum* that were not identified using BLASTp searches [75, 113-120], exemplifying the enhanced performance of PeptideMiner.

The absence of conoinsulins in *C. rattus and C. imperialis* is consistent with previous findings indicating that

conoinsulins are not universally recruited into venom glands [79]. Of note, MIP-like conoinsulin G2, identified previously [15], was not observed in our *C. geographus* transcriptomes (including a direct manual search). These results collectively highlight the diverse sequence variability and wide distribution of conoinsulins in the genus *Conus*.

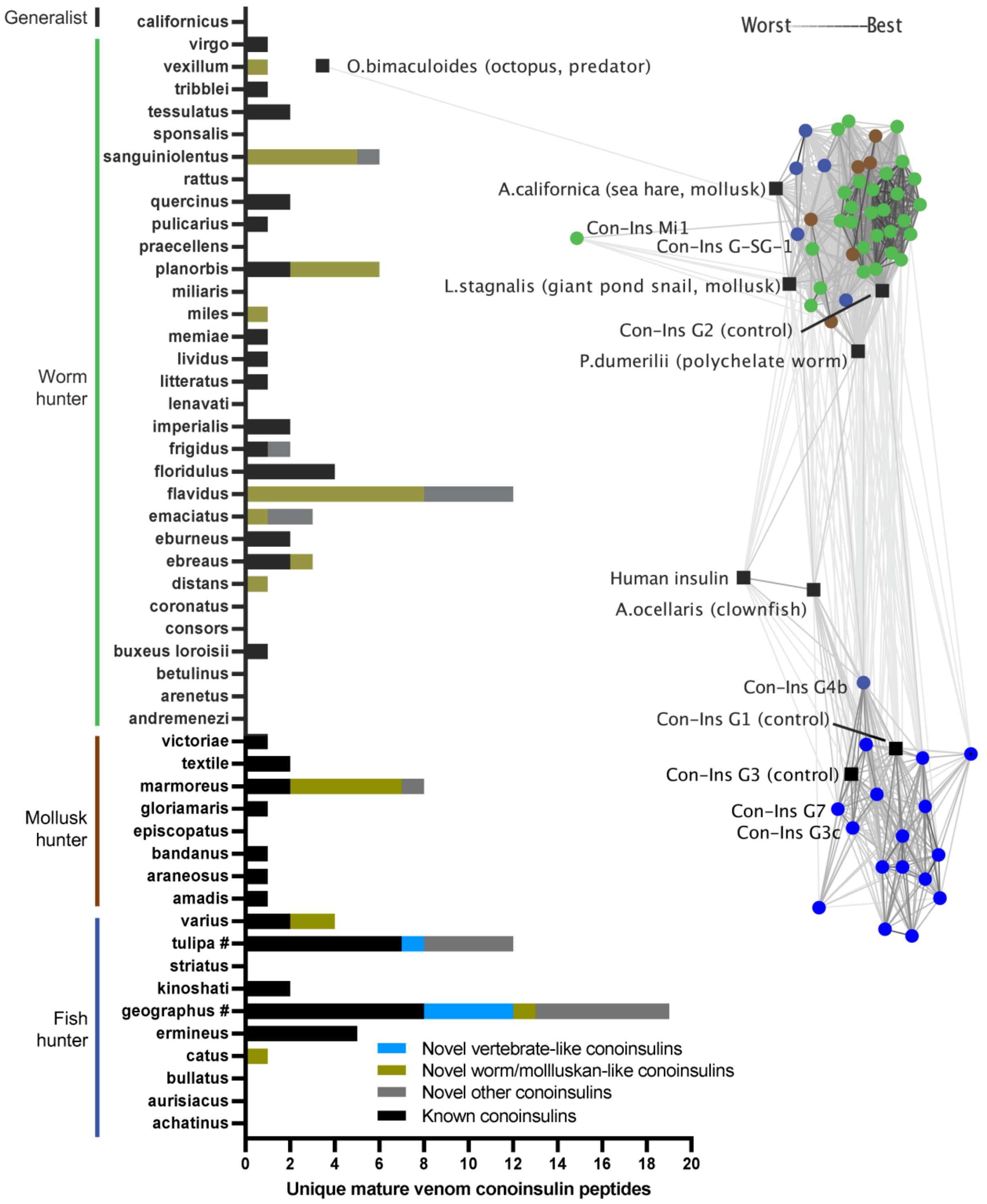

Figure 6. Number and similarity of known conoinsulin sequences and newly identified conoinsulin sequences by PeptideMiner. Known conoinsulin sequences (from UniProt, GenBank, or literature [114, 118, 121]) are presented in black, and newly identified conoinsulin sequences are further categorized as vertebrate-like (light blue), molluskan-like (olive), or other (gray). Species were grouped according to their diet: fish hunters (dark blue), mollusk hunters (brown), worm hunters (green), and generalists. # indicates net-hunters. All-against-all BLAST e-value clustering was performed using CLANS, and shows similarity between sequences (line length, and weight) of newly discovered conoinsulins (circles) and previously characterized sequences (squares).

Using PeptideMiner, we also identified five novel vertebrate-like mature conoinsulin sequences from two fish-hunting species, *C. geographus* and *C. tulipa*: Con-Ins G3c, G4b, G5, G7 and T3b (Figure 6), adding to the seven previously identified C. *geographus* and five previously identified C. *tulipa* conoinsulins [122]. We synthesized Con-Ins G3c, G4b, and G7 and assessed their binding affinity at the human insulin receptor. Con-Ins G4b and G7 demonstrated low nanomolar binding at hIR-B, underscoring the applicability of PeptideMiner and our approach for identifying new insulin-like analogs with activity at the human receptors. Con-Ins G3c was inactive at hIR-B; however, its SAR was still useful, particularly in highlighting the importance of Gly^{8}_{B} for human insulin receptor binding. Notably, the differences between Con-Ins G4b and G7 to human insulin do not involve any of the oligomerization surface residues (Ala^{12}_{A}, Leu^{13}_{A}, Glu^{17}_{A}, His^{10}_{B}, Glu^{13}_{B}, Leu^{17}_{B}), which could be exploited for the development of fast-acting insulin analogs [123, 124].

Taken together, these results underscore PeptideMiner as a powerful new discovery tool that enhances our capabilities in identifying and mapping neuropeptides across the animal kingdom and discovering new peptides with affinity for human receptors. These enhanced capabilities are expected to pave the way for developing new pharmacological probes or therapeutic leads, thereby offering promising prospects for advancing biomedical research and drug development.

Conclusions

PeptideMiner is a new and highly versatile computational discovery platform designed to efficiently identify (neuro)peptide families across diverse databases and species. Demonstrating superior performance compared to BLASTp, PeptideMiner offers several advantages in accurate neuropeptide discovery, adeptly handling short and divergent sequences, and efficiently mapping neuropeptides across evolutionarily distant species. This study highlights the untapped potential of venoms as a rich new source for neuropeptide analogs and provides a robust framework for their systematic and efficient exploration to discover valuable new pharmacological probes and therapeutic leads. PeptideMiner unveiled 36 unique conoinsulins (Figure S4) and 8 natriuretic peptides (Figure 3), substantially expanding our understanding of the molecular diversity within venoms across different animal species. Selected conoinsulins bound to the human insulin receptor, emphasizing the translational promise of this approach.

Taken together, PeptideMiner is an invaluable open-access tool poised to propel neuropeptide research forward, deepen our comprehension of neuropeptide signaling in health and disease, and expedite the discovery and development of novel pharmacological probes and therapeutic interventions.

Materials and Methods

Materials

Fmoc amino acids were from Iris Biotech GmbH (Marktredwitz, Germany), and Fmoc- Asp(t-Bu)-Wang resin (loading 0.81 mmol/g) and Fmoc-L-His(Trt)-AC TentaGel resin (loading 0.22 mmol/g) was from Rapp Polymere GmbH (Tübingen, Germany). Acetonitrile (ACN) was obtained from Merck (Bayswater, Australia). Dimethylformamide (DMF), methanol (MeOH), trifluoroacetic acid (TFA), and diethyl ether were obtained from Chem-Supply (Gillman, Australia). All solvents were obtained with the highest available purity and used without further purification. All other reagents, including N,N-diisopropylethylamine (DIEA), (O-(6-chlorobenzotriazol-1-yl)-N,N,N’,N’-tetramethyluronium hexafluorophosphate (HCTU), Anisole, 2,2′-(ethylenedioxy)diethanethiol (DODt), triisopropylsilane (TIPS), 2,2’-dipyridyldisulfide (DPDS) and trifluoromethanesulfonic acid (TFMSA) and solvents were obtained from Sigma Aldrich (Merck, Sydney, NSW, Australia) in the highest available purity. Solvents for reversed-phase HPLC (RP-HPLC) consisted of 0.05% TFA/H_2O (Solvent A) and 0.043% TFA/ACN (Solvent B). Analytical HPLC was performed with

column heating at 40°C and detection at 214 nm. Preparative HPLC was performed on a Vydac Protein and Peptide C_{18} preparative column, and crude and fractions were analyzed using RP-HPLC and electrospray ionization mass spectrometry (ESI-MS). Mass analysis of the final product was performed on a Q-Star Pulsar mass spectrometer (SCIEX, Ontario, Canada) with a Series 1100 solvent delivery system equipped with an auto-injector (Agilent Technologies Inc., Palo Alto, CA, USA) and a Kromasil Classic liquid chromatography MS (LC-MS) C_{18} column (100 Å, 3.5 µm, 150 mm x 2.1 mm). Data acquisition and processing were carried out using Analyst software v1.1 (SCIEX, Canada). Trizol was from ThermoFisher Scientific. Oligotex mRNA Mini Kit was from Qiagen (Valencia, CA, USA).

Transcriptome Preparation

The transcriptomes were sourced from unpublished in-house collections (23 *Conus* and *S. horrida* transcriptomes) or downloaded from the NCBI Sequence Read Archive (SRA) database. A full list of transcriptomes searched is provided in Table S3. For the in-house collections, total RNA was extracted from the stripped venom gland cells using Trizol (Invitrogen) reagent according to the manufacturer's instructions. mRNA was purified from the total RNA using Oligotex mRNA Mini Kit (Qiagen, Valencia, CA, USA) according to the manufacturer's instructions. cDNA library construction and sequencing were carried out using a Roche GS FLX Titanium sequencer at the Australian Genomic Research Facility (AGRF). Data assembly was carried out using Newbler 2.3 Life Science, Frederick, CO). In total, 57 animal venom organ transcriptomes from 48 species, including 39 invertebrates and 9 vertebrates across five phyla (Cnidaria, Arthropoda, Mollusca, Annelida, and Chordata) were searched for the natriuretic peptide neuropeptide family, and 23 cone snail transcriptomes comprising 20 venom ducts, two circumoesophageal nerve rings, and one salivary gland transcriptome for the conoinsulin family.

PeptideMiner Workflow

PeptideMiner is constructed as a series of steps with user-friendly output files created at each step. PeptideMiner takes as input one or more profile-HMMs of the neuropeptide family of interest and searches a database of amino acid sequences that the user can provide as multiple fasta files (for instance, one fasta file for each transcriptome) or combine into a single large fasta file database.

Hmmsearch from the HMMER3 package searches the profile-HMM against the amino acid database (in this work, translated transcriptomes). PeptideMiner runs hmmsearch and creates an output for each dataset searched

(Step 0). The hmmsearch output does not include the hit sequence; therefore, the hit read names are matched with their respective read name in the amino acid database, and the sequence is extracted. The sequence is combined with the hmmsearch output to generate a report of identified neuropeptide candidates. The bioinformatics pipeline subsequently filters, processes, and annotates this initial neuropeptide identification report.

The hmmsearch search results are stored in an SQLite database (Figure S2). To facilitate downstream processing, all hmmsearch search hits are combined into a single file (Step 2). If more than one profile-HMM was used for a single neuropeptide family, the combined hmmsearch output could contain duplicate reads. For this case, the hit with the lowest E-value is selected. The E-value is an estimate of the Expected number of errors. In other words, it represents the probability of observing such results by chance. The E-value of a read returned by hmmsearch represents the statistical significance of the hit. The lower the E-value, typically <<1, the more statistically significant the hit and the less likely it is a random hit [28].

In Step 3, the protein-coding sequences (CDS) of the hypothetical neuropeptide precursors are predicted using a Python script that extracts the sequence between the methionine, 'M', (encoding the start codon) and the stop codon or, if there is no stop codon, the end of the contig (Figure 1, Step 3). Multiple CDS can be identified from a single contig if multiple methionine residues are present. This step is relevant for protein sequences obtained by direct translation of nucleotide transcripts. The user can specify a minimum cut-off length for the CDS to minimize false positives.

The signal peptide of the predicted CDS is then identified using SignalP [34] (Figure 1, Step 4). If present, the signal peptide is subsequently excised from the sequence of the precursor to facilitate subsequent mature peptide identification.

Step 5 aims to identify the mature peptide(s) in the CDS; FASTA36 [35] aligns all the CDS to a list of known mature peptides of the neuropeptide family of interest, followed by cleavage site prediction using an algorithm from the ConoServer annotation pipeline [44] (Step 5).

The predicted mature peptides for all sequences from the same transcriptome are then compiled, and duplicate mature peptides are removed (Step 6). Protein-protein BLAST (BLASTp) is then used to annotate the predicted mature peptides by homology using a list of known neuropeptide amino acid sequences (Step 7).

PeptideMiner Search Parameters

The user can modify several parameters of PeptideMiner to optimize neuropeptide identification. Firstly, the user can specify the minimum length of the CDS in the number of amino acids, enabling the filtering of

sequence candidates that are too short (Step 3), for example, to exclude incomplete CDS or fragments.

Secondly, the user can modify the SignalP parameters to identify the signal peptide in CDS (Step 4). This step is important because the presence of a signal peptide is supportive of a neuropeptide. The user can change the SignalP D-value (the score used to discriminate signal peptides from non-signal peptides; peptides with no signal peptide have a very low D-value) and minimum signal peptide length, which are set to a default of 0.45 and 9, respectively, which is the default setting for non-transmembrane eukaryotic precursors [34]. Reducing the D-value would increase speed at the cost of sensitivity.

Finally, the user can alter three parameters for mature peptide identification (Step 5). FASTA36 [35] is used to align known mature peptides to the CDS to identify mature peptides in the CDS. The user can specify the E-value cut-off of the FASTA36 search as well as the minimum and maximum length allowed for the mature peptides. The E-value is typically dependent on the overall length of the alignment, the percentage identity, and the size of the database. Generally, a lower E-value indicates a better quality in the alignment between the mature peptide and the known peptide. The optimal E-value cut-off varies depending on the neuropeptide family because the various neuropeptides have different lengths and degrees of sequence conservation. In practice, an E-value range between $1x10^{-4}$–1 is typically used, although the cut-off will vary depending on the query sequence length and database size.

Pipeline Output

The pipeline creates several outputs for the user, including a final output file that lists all the hits and their similarity (% identity and BLASTp E-value) to known sequences (Step 8). In addition, PeptideMiner generates a report that lists the number of profile-HMMs used, the number of files searched, the number of hits the hmmsearch returned, and the final number of hits, including the number of CDS and mature peptides identified. A separate file reports the number of reads found with each profile-HMM used to search the database.

RNA isolation and transcriptome generation

All cone snails used to generate the transcriptomes were collected from the Great Barrier Reef, Queensland, Australia (GBRMP permit G10/33243.1). Cone snails were sacrificed and dissected immediately on ice. The whole venom gland was separated from the other tissues (not including the venom bulb at the proximal end and the proboscis at the distal end), and the venom gland cells were stripped out from the venom gland. Total RNA was extracted from the stripped venom gland cells using TRIzol reagent according to the manufacturer's instructions. mRNA was purified from the total RNA using an Oligotex mRNA Mini Kit according to the

manufacturer's instructions. cDNA library construction and sequencing were carried out using a Roche GS FLX Titanium sequencer at the Australian Genomic Research Facility (AGRF). Data assembly was carried out using Newbler 2.3 Life Science, Frederick, CO). Only one individual was used to generate the transcriptomes of larger species (*C. geographus*, *C. tulipa*, *C. planorbis*, *C. distans*, *C. sangunilantus*, *C. vexillum*, *C. marmoreus*, *C. miles*, *C. imperialis)* and multiple specimens were used for smaller species (e.g., six specimens for *C. catus*).

Solid-phase peptide synthesis

Synthesis of Con-Ins G3c, G4b, and G7

The linear conoinsulin A-chains were manually synthesized using Fmoc-SPPS on an Fmoc-L-His(Trt)-AC TentaGel resin on a 0.22 mmol scale. A regioselective folding strategy using acetamidomethyl (Acm), *tert*-butyl (tBu), and trityl (Trt) cysteine protecting groups was employed to achieve the A-chain intrachain and two A-B-chain interchain disulfide bonds. N-terminal Fmoc deprotection was achieved with 20% v/v piperidine in DMF for 2×5 min. Each Fmoc-protected amino acid (3 eq.) was coupled using DMF as the solvent and DIEA (1 M in DMF) with HCTU (3 eq.) as the activating agents for 50 min. The cycle of deprotection, washing, and coupling was repeated until the full-length peptide was obtained. The completed linear A-chain was cleaved by a cocktail of TFA/anisole/DODt/TIPS (94/3/2/1%) for 3 h. The cleavage mixture was then filtered, concentrated by a stream of N_2, precipitated in cold diethyl ether, and centrifuged for 5 min. The crude A-chain (544 mg) was collected as a white powder and used directly for further synthesis.

The A-chain intrachain disulfide bond was formed by dissolving the crude linear A-chain (0.102 mmol) in an H_2O/ACN mixture (4:1, 500 mL) with 5 drops of DIEA. After adding DPDS (1.0 eq.) in 1.0 mL MeOH, the mixture was stirred for 2.5 h at 40°C. The mixture was purified using a preparative RP-HPLC (15-55% solvent B in 30 min) and lyophilized.

The cyclized A-chain (31.5 μmol) and DPDS (4 eq.) were dissolved in an anisole/TFA mixture (1:9, 2 mL), and then a TFMSA/TFA mixture (1:4, 2 mL) was added. The resulting mixture was stirred at 0°C for 45 min, then precipitated in cold diethyl ether and centrifuged (5 repeats). The crude peptide was purified by a semi-preparative RP-HPLC (15-55% solvent B in 30 min) and lyophilized.

All three B-chains were purchased from GL Biochem (Shanghai, China). To a mixture of conoinsulin B-chain in acidic guanidium HCl buffer (6 M, 1 mL, pH = 5.0), conoinsulin A-chain in alkaline guanidium HCl buffer (6 M, 2 mL, pH = 8.5) was added dropwise. The resulting mixture was stirred for 15 min, purified using a semi-preparative RP-HPLC (15-45% solvent B in 30 min), and lyophilized.

To the lyophilized powder was added an aqueous solution of HCl (60 mM, 0.4 mL), acetic acid (3.15 mL), and an iodine solution (20 mM in acetic acid, 4.2 mL). The mixture was stirred for 1 h, then precipitated in cold ether and centrifuged. The crude peptide was purified by a semi-preparative RP-HPLC (15-45% solvent B in 30 min) and lyophilized.

Con-Ins G3c: A-chain (10 mg) and B-chain (10.5 mg) were used, yielding 0.5 mg of white powder. Con-Ins G7: A-chain (10 mg) and B-chain (12 mg) yielded 1.3 mg of white powder. Con-Ins G4b: A-chain (5.9 mg) and B-chain (7.5 mg) yielded 0.7 mg of white powder.

Human insulin receptor-B binding experiments

Receptor binding was measured as described previously [94]. Briefly, insulin-like growth factor 1 receptor (IGF-1R)-negative cells overexpressing the human insulin receptor-B (hIR-B) were generated. Cells were serum-starved for 4 h before lysis. Lysates were captured in a 96-well plate previously coated with anti-IR antibody. Approximately 500,000 fluorescent counts of europium-labeled human insulin (Eu-insulin) were added to each well along with increasing concentrations of unlabeled competitor and incubated for 16 h at 4°C. After washing, time-resolved fluorescence was measured using 340 nm excitation and 612 nm emission filters with the BMG Lab Technologies Polarstar fluorometer (Mornington, Australia). Insulin and synthetic conoinsulin analog curves are from three separate experiments, each point performed in triplicate. Binding affinity was expressed in IC_{50} and represents the ligand concentration (human insulin or conoinsulin) necessary to displace 50% of Eu-insulin from hIR-B.

Conoinsulin structure prediction

Three-dimensional structures for Con-Ins G3C, G4b and G7 were predicted using AlphaFold2 [97], implemented in ColabFold [125] running remotely on a Python 3 Google Compute Engine backend. The alphafold2_multimer_v3 model and no template information were used for the prediction. The highest-ranked, Amber-relaxed model was aligned with the crystal structure of human insulin (PDB:3w7y).

Clustering Analysis

To compare the identified insulin-like peptides and natriuretic peptides, we used CLANS [126] to cluster mature amino acid sequences based on all-against-all pairwise BLASTp E-values. Pairwise BLASTp searches were performed with the CLANS web-utility (https://toolkit.tuebingen.mpg.de/tools/clans) [127] using default parameters, while clustering and visualization of the resulting similarity matrix were completed with the Java-based CLANS tool, using P-values better than 1e-4 and otherwise default parameters.

Data availability

PeptideMiner is registered with bio.tools (biotools:peptideminer) and SciCrunch.org (RRID: SCR-026456), is publicly available for download from GitHub (https://www.github.com/muttenthalerlab/PeptideMiner), and is released under the GNU General Public License v3.0.

Acknowledgments

We thank Dr. Akhter Hossain for synthesizing Con-Ins G3c, G4b, and G7, Dr. Himaya Hewage for transcriptome details, A/Prof Lachlan Rash for access to the venom transcriptome of the King Baboon Spider, and Dr. Prashanth Jutty Rajan for initial training in transcriptome analysis. M.M. was supported by the European Research Council under the European Union's Horizon 2020 research and innovation program (714366) and by the Australian Research Council (DE150100784, DP190101667, FT210100266), and by the National Health and Medical Research Council (2037680). EABU was supported by the European Research Council (ERC-2021-STG under grant agreement No 101039862) and the Norwegian Research Council (FRIPRO-YRT Fellowship no. 287462).

References

1. King GF. Venoms as a platform for human drugs: translating toxins into therapeutics. Expert Opin Biol Th. 2011;11 11:1469-84.
2. Lewis RJ and Garcia ML. Therapeutic potential of venom peptides. Nat Rev Drug Discov. 2003;2 10:790-802.
3. Muttenthaler M, King GF, Adams DJ and Alewood PF. Trends in peptide drug discovery. Nature Reviews Drug Discovery. 2021;20 4:309-25.
4. Näreoja K and Näsman J. Selective targeting of G-protein-coupled receptor subtypes with venom peptides. Acta Physiologica. 2012;204 2:186-201.
5. Sharpe IA, Gehrmann J, Loughnan ML, Thomas L, Adams DA, Atkins A, et al. Two new classes of conopeptides inhibit the α1-adrenoceptor and noradrenaline transporter. Nature Neuroscience. 2001;4 9:902-7.
6. Warkentin TE and Koster A. Bivalirudin: a review. Expert Opinion on Pharmacotherapy. 2005;6 8:1349-71.
7. Zhang L, Lu SH, Li L, Tao Y-G, Wan YL, Senga H, et al. Batroxobin Mobilizes Circulating Endothelial Progenitor Cells in Patients With Deep Vein Thrombosis. Clinical and Applied Thrombosis/Hemostasis. 2011;17 1:75-9.
8. Eagles DA, Saez NJ, Krishnarjuna B, Bradford JJ, Chin YK-Y, Starobova H, et al. A peptide toxin in ant venom mimics vertebrate EGF-like hormones to cause long-lasting hypersensitivity in mammals. Proceedings of the National Academy of Sciences. 2022;119 7:e2112630119.
9. Jami S, Erickson A, Brierley S and Vetter I. Pain-Causing Venom Peptides: Insights into Sensory Neuron Pharmacology. Toxins. 2017;10 1:15.
10. Netirojjanakul C and Miranda LP. Progress and challenges in the optimization of toxin peptides for development as pain therapeutics. Curr Opin Chem Biol. 2017;38:70-9.
11. Miljanich GP. Ziconotide: neuronal calcium channel blocker for treating severe chronic pain. Curr Med Chem. 2004;11 23:3029-40.
12. Taylor K, Kim D, Nielsen LL, Aisporna M, Baron AD and Fineman MS. Day-long Subcutaneous Infusion of Exenatide Lowers Glycemia in Patients with Type 2 Diabetes. Hormone and Metabolic Research. 2005;37 10:627-32.
13. Cruz LJ, de Santos V, Zafaralla GC, Ramilo CA, Zeikus R, Gray WR and Olivera BM. Invertebrate vasopressin/oxytocin homologs. Characterization of peptides from Conus geographus and Conus straitus venoms. J Biol Chem. 1987;262 33:15821-4.
14. Craig AG, Norberg T, Griffin D, Hoeger C, Akhtar M, Schmidt K, et al. Contulakin-G, an O-Glycosylated Invertebrate Neurotensin. Journal of Biological Chemistry. 1999;274 20:13752-9.
15. Safavi-Hemami H, Gajewiak J, Karanth S, Robinson SD, Ueberheide B, Douglass AD, et al. Specialized insulin is used for chemical warfare by fish-hunting cone snails. Proceedings of the National Academy of Sciences. 2015;112 6:1743-8.
16. Robinson SD, Safavi-Hemami H, Raghuraman S, Imperial JS, Papenfuss AT, Teichert RW, et al. Discovery by proteogenomics and characterization of an RF-amide neuropeptide from cone snail venom. J Proteomics. 2015;114:38-47.
17. Schweitz H, Vigne P, Moinier D, Frelin C and Lazdunski M. A New Member of the Natriuretic Peptide Family Is Present in the Venom of the Green Mamba (Dendroaspis-Angusticeps). Journal of Biological Chemistry. 1992;267 20:13928-32.
18. Hokfelt T, Broberger C, Xu ZQ, Sergeyev V, Ubink R and Diez M. Neuropeptides--an overview. Neuropharmacology. 2000;39 8:1337-56.
19. Mendel HC, Kaas Q and Muttenthaler M. Neuropeptide signalling systems – An underexplored target for venom drug discovery. Biochemical Pharmacology. 2020;181:114129.
20. Elphick MR, Mirabeau O and Larhammar D. Evolution of neuropeptide signalling systems. J Exp Biol. 2018;221 Pt 3.
21. Buermans HP and den Dunnen JT. Next generation sequencing technology: Advances and applications. Biochim Biophys Acta. 2014;1842 10:1932-41.
22. Calvete JJ. Venomics: integrative venom proteomics and beyond. Biochem J. 2017;474:611-34.

23. Chen YP and Chen F. Identifying targets for drug discovery using bioinformatics. Expert Opin Ther Targets. 2008;12 4:383-9.
24. Escoubas P and King GF. Venomics as a drug discovery platform. Expert Rev Proteomic. 2009;6 3:221-4.
25. Oldrati V, Arrell M, Violette A, Perret F, Sprungli X, Wolfender JL and Stocklin R. Advances in venomics. Mol Biosyst. 2016;12 12:3530-43.
26. Clynen E, Liu F, Husson SJ, Landuyt B, Hayakawa E, Baggerman G, et al. Bioinformatic approaches to the identification of novel neuropeptide precursors. Methods in molecular biology. 2010;615:357-74.
27. Caers J, Verlinden H, Zels S, Vandersmissen HP, Vuerinckx K and Schoofs L. More than two decades of research on insect neuropeptide GPCRs: an overview. Front Endocrinol (Lausanne). 2012;3:151.
28. Eddy SR. Accelerated Profile HMM Searches. PLoS Comput Biol. 2011;7 10:e1002195.
29. Durbin R, Eddy SR, Krogh A and Mitchison GJ. Biological Sequence Analysis: Probabilistic Models of Proteins and Nucleic Acids. In: 1998.
30. Park J, Karplus K, Barrett C, Hughey R, Haussler D, Hubbard T and Chothia C. Sequence comparisons using multiple sequences detect three times as many remote homologues as pairwise methods. J Mol Biol. 1998;284 4:1201-10.
31. Yoon BJ. Hidden Markov Models and their Applications in Biological Sequence Analysis. Curr Genomics. 2009;10 6:402-15.
32. Eddy SR. Profile hidden Markov models. Bioinformatics. 1998;14 9:755-63.
33. Eddy SR: HMMER 3.1b. hmmer.org (2015).
34. Petersen TN, Brunak S, von Heijne G and Nielsen H. SignalP 4.0: discriminating signal peptides from transmembrane regions. Nat Methods. 2011;8 10:785-6.
35. Pearson WR. Searching protein sequence libraries: comparison of the sensitivity and selectivity of the Smith-Waterman and FASTA algorithms. Genomics. 1991;11 3:635-50.
36. Altschul SF, Madden TL, Schaffer AA, Zhang J, Zhang Z, Miller W and Lipman DJ. Gapped BLAST and PSI-BLAST: a new generation of protein database search programs. Nucleic Acids Res. 1997;25 17:3389-402.
37. Hipp DR, Kennedy D and Mistachkin J. SQLite (Version 3.27.2). SQLite Development Team, 2015.
38. Southey BR, Sweedler JV and Rodriguez-Zas SL. A python analytical pipeline to identify prohormone precursors and predict prohormone cleavage sites. Front Neuroinform. 2008;2:7.
39. Bassi S. A primer on python for life science researchers. PLoS Comput Biol. 2007;3 11:e199.
40. Edgar RC. MUSCLE: multiple sequence alignment with high accuracy and high throughput. Nucleic Acids Res. 2004;32 5:1792-7.
41. Sievers F, Wilm A, Dineen D, Gibson TJ, Karplus K, Li W, et al. Fast, scalable generation of high-quality protein multiple sequence alignments using Clustal Omega. Mol Syst Biol. 2011;7:539.
42. Waterhouse AM, Procter JB, Martin DM, Clamp M and Barton GJ. Jalview Version 2--a multiple sequence alignment editor and analysis workbench. Bioinformatics. 2009;25 9:1189-91.
43. Eddy SR. A new generation of homology search tools based on probabilistic inference. Genome Inform. 2009;23 1:205-11.
44. Kaas Q, Yu R, Jin AH, Dutertre S and Craik DJ. ConoServer: updated content, knowledge, and discovery tools in the conopeptide database. Nucleic Acids Res. 2012;40 Database issue:D325-30.
45. Suzek BE, Wang Y, Huang H, McGarvey PB, Wu CH and UniProt C. UniRef clusters: a comprehensive and scalable alternative for improving sequence similarity searches. Bioinformatics. 2015;31 6:926-32.
46. Potter LR, Yoder AR, Flora DR, Antos LK and Dickey DM. Natriuretic peptides: their structures, receptors, physiologic functions and therapeutic applications. Handb Exp Pharmacol. 2009; 191:341-66.
47. Pandit K, Mukhopadhyay P, Ghosh S and Chowdhury S. Natriuretic peptides: Diagnostic and therapeutic use. Indian J Endocrinol Metab. 2011;15 Suppl 4:S345-53.
48. Poulos JE, Gower WR, Jr., Friedl FE and Vesely DL. Atrial natriuretic peptide gene expression within invertebrate hearts. Gen Comp Endocrinol. 1995;100 1:61-8.
49. Vesely DL and Giordano AT. The most primitive heart in the animal kingdom contains the atrial natriuretic peptide hormonal system. Comp Biochem Physiol C. 1992;101 2:325-9.

50. Peterfi O, Boda F, Szabo Z, Ferencz E and Baba L. Hypotensive Snake Venom Components-A Mini-Review. Molecules. 2019;24 15.
51. Higuchi S, Murayama N, Saguchi K, Ohi H, Fujita Y, Camargo AC, et al. Bradykinin-potentiating peptides and C-type natriuretic peptides from snake venom. Immunopharmacology. 1999;44 1-2:129-35.
52. Ichiki T, Dzhoyashvili N and Burnett JC, Jr. Natriuretic peptide based therapeutics for heart failure: Cenderitide: A novel first-in-class designer natriuretic peptide. Int J Cardiol. 2018.
53. Alves RS, Ximenes RM, Jorge AR, Nascimento NR, Martins RD, Rabello MM, et al. Isolation, homology modeling and renal effects of a C-type natriuretic peptide from the venom of the Brazilian yellow scorpion (Tityus serrulatus). Toxicon. 2013;74:19-26.
54. de Plater GM, Martin RL and Milburn PJ. A C-type natriuretic peptide from the venom of the platypus (Ornithorhynchus anatinus): structure and pharmacology. Comp Biochem Physiol C Pharmacol Toxicol Endocrinol. 1998;120 1:99-110.
55. Fry BG, Roelants K, Winter K, Hodgson WC, Griesman L, Kwok HF, et al. Novel venom proteins produced by differential domain-expression strategies in beaded lizards and gila monsters (genus Heloderma). Mol Biol Evol. 2010;27 2:395-407.
56. Ziegman R, Undheim EAB, Baillie G, Jones A and Alewood PF. Investigation of the estuarine stonefish (Synanceia horrida) venom composition. J Proteomics. 2019;201:12-26.
57. Vink S, Jin AH, Poth KJ, Head GA and Alewood PF. Natriuretic peptide drug leads from snake venom. Toxicon. 2012;59 4:434-45.
58. Schweitz H, Vigne P, Moinier D, Frelin C and Lazdunski M. A new member of the natriuretic peptide family is present in the venom of the green mamba (Dendroaspis angusticeps). J Biol Chem. 1992;267 20:13928-32.
59. Zhang Y, Wu J, Yu G, Chen Z, Zhou X, Zhu S, et al. A novel natriuretic peptide from the cobra venom. Toxicon. 2011;57 1:134-40.
60. Xie B, Dashevsky D, Rokyta D, Ghezellou P, Fathinia B, Shi Q, et al. Dynamic genetic differentiation drives the widespread structural and functional convergent evolution of snake venom proteinaceous toxins. BMC Biology. 2022;20 1.
61. Soares MR, Oliveira-Carvalho AL, Wermelinger LS, Zingali RB, Ho PL, Junqueira-de-Azevedo IL and Diniz MR. Identification of novel bradykinin-potentiating peptides and C-type natriuretic peptide from Lachesis muta venom. Toxicon. 2005;46 1:31-8.
62. Schmidt JJ, Weinstein SA and Smith LA. Molecular properties and structure-function relationships of lethal peptides from venom of Wagler's pit viper, Trimeresurus wagleri. Toxicon. 1992;30 9:1027-36.
63. Tsai MC, Hsieh WH, Smith LA and Lee CY. Effects of waglerin-I on neuromuscular transmission of mouse nerve-muscle preparations. Toxicon. 1995;33 3:363-71.
64. Tan CH, Tan KY and Tan NH. De Novo Assembly of Venom Gland Transcriptome of Tropidolaemus wagleri (Temple Pit Viper, Malaysia) and Insights into the Origin of Its Major Toxin, Waglerin. Toxins (Basel). 2023;15 9.
65. Yang Y, Xiong J, Zhou Z, Huo F, Miao W, Ran C, et al. The genome of the myxosporean Thelohanellus kitauei shows adaptations to nutrient acquisition within its fish host. Genome Biol Evol. 2014;6 12:3182-98.
66. Shabanpoor F, Separovic F and Wade JD. The human insulin superfamily of polypeptide hormones. Vitam Horm. 2009;80:1-31.
67. Weiss M, Steiner DF and Philipson LH. Insulin Biosynthesis, Secretion, Structure, and Structure-Activity Relationships. In: De Groot LJ, Chrousos G, Dungan K, Feingold KR, Grossman A, Hershman JM, et al., editors. Endotext. South Dartmouth (MA); 2000.
68. Chan SJ and Steiner DF. Insulin through the ages: Phylogeny of a growth promoting and metabolic regulatory hormone. Am Zool. 2000;40 2:213-22.
69. De Meyts P. Insulin and its receptor: structure, function and evolution. Bioessays. 2004;26 12:1351-62.
70. Tokarz VL, Macdonald PE and Klip A. The cell biology of systemic insulin function. Journal of Cell Biology. 2018;217 7:2273-89.

71. Adams MJ, Blundell TL, Dodson EJ, Dodson GG, Vijayan M, Baker EN, et al. Structure of Rhombohedral 2 Zinc Insulin Crystals. Nature. 1969;224 5218:491-&.
72. Lisi GP, Png CYM and Wilcox DE. Thermodynamic Contributions to the Stability of the Insulin Hexamer. Biochemistry. 2014;53 22:3576-84.
73. Blumenthal S. From insulin and insulin-like activity to the insulin superfamily of growth-promoting peptides: a 20th-century odyssey. Perspect Biol Med. 2010;53 4:491-508.
74. Smit AB, van Kesteren RE, Li KW, Van Minnen J, Spijker S, Van Heerikhuizen H and Geraerts WP. Towards understanding the role of insulin in the brain: lessons from insulin-related signaling systems in the invertebrate brain. Prog Neurobiol. 1998;54 1:35-54.
75. Dutertre S, Jin AH, Vetter I, Hamilton B, Sunagar K, Lavergne V, et al. Evolution of separate predation- and defence-evoked venoms in carnivorous cone snails. Nat Commun. 2014;5:3521.
76. Akondi KB, Muttenthaler M, Dutertre S, Kaas Q, Craik DJ, Lewis RJ and Alewood PF. Discovery, Synthesis, and Structure-Activity Relationships of Conotoxins. Chem Rev. 2014;114 11:5815-47.
77. Jin A-H, Muttenthaler M, Dutertre S, Himaya SWA, Kaas Q, Craik DJ, et al. Conotoxins: Chemistry and Biology. Chem Rev. 2019;119 21:11510-49.
78. Olivera BM, Seger J, Horvath MP and Fedosov AE. Prey-Capture Strategies of Fish-Hunting Cone Snails: Behavior, Neurobiology and Evolution. Brain Behav Evol. 2015;86 1:58-74.
79. Safavi-Hemami H, Lu A, Li Q, Fedosov AE, Biggs J, Showers Corneli P, et al. Venom Insulins of Cone Snails Diversify Rapidly and Track Prey Taxa. Mol Biol Evol. 2016;33 11:2924-34.
80. Ahorukomeye P, Disotuar MM, Gajewiak J, Karanth S, Watkins M, Robinson SD, et al. Fish-hunting cone snail venoms are a rich source of minimized ligands of the vertebrate insulin receptor. eLife. 2019;8.
81. Dutertre S, Jin A-H, Vetter I, Hamilton B, Sunagar K, Lavergne V, et al. Evolution of separate predation- and defence-evoked venoms in carnivorous cone snails. Nature Communications. 2014;5 1.
82. Laugesen SH, Chou DHC and Safavi-Hemami H. Unconventional insulins from predators and pathogens. Nat Chem Biol. 2022;18 7:688-97.
83. Menting JG, Gajewiak J, Macraild CA, Chou DH-C, Disotuar MM, Smith NA, et al. A minimized human insulin-receptor-binding motif revealed in a Conus geographus venom insulin. Nature Structural & Molecular Biology. 2016;23 10:916-20.
84. Robinson SD and Safavi-Hemami H. Insulin as a weapon. Toxicon. 2016;123:56-61.
85. Safavi-Hemami H, Lu A, Li Q, Fedosov AE, Biggs J, Showers Corneli P, et al. Venom Insulins of Cone Snails Diversify Rapidly and Track Prey Taxa. Molecular Biology and Evolution. 2016;33 11:2924-34.
86. Southey BR, Amare A, Zimmerman TA, Rodriguez-Zas SL and Sweedler JV. NeuroPred: a tool to predict cleavage sites in neuropeptide precursors and provide the masses of the resulting peptides. Nucleic Acids Res. 2006;34 Web Server issue:W267-72.
87. Safavi-Hemami H, Gajewiak J, Karanth S, Robinson SD, Ueberheide B, Douglass AD, et al. Specialized insulin is used for chemical warfare by fish-hunting cone snails. Proc Natl Acad Sci USA. 2015;112 6:1743-8.
88. Rholam M, Brakch N, Germain D, Thomas DY, Fahy C, Boussetta H, et al. Role of amino acid sequences flanking dibasic cleavage sites in precursor proteolytic processing. The importance of the first residue C-terminal of the cleavage site. Eur J Biochem. 1995;227 3:707-14.
89. Pardos-Blas JR, Tenorio MJ, Galindo JCG and Zardoya R. Comparative Venomics of the Cryptic Cone Snail Species Virroconus ebraeus and Virroconus judaeus. Marine Drugs. 2022;20 2:149.
90. Biggs JS, Olivera BM and Kantor YI. Alpha-conopeptides specifically expressed in the salivary gland of Conus pulicarius. Toxicon. 2008;52 1:101-5.
91. Lavergne V, Harliwong I, Jones A, Miller D, Taft RJ and Alewood PF. Optimized deep-targeted proteotranscriptomic profiling reveals unexplored Conus toxin diversity and novel cysteine frameworks (vol 112, pg E3782, 2015). P Natl Acad Sci USA. 2015;112 45:E6253-E.
92. Gao BM, Peng C, Zhu YB, Sun YH, Zhao T, Huang Y and Shi Q. High Throughput Identification of Novel Conotoxins from the Vermivorous Oak Cone Snail (Conus quercinus) by Transcriptome Sequencing. Int J Mol Sci. 2018;19 12.

93. Escribano O, Beneit N, Rubio-Longás C, López-Pastor AR and Gómez-Hernández A. The Role of Insulin Receptor Isoforms in Diabetes and Its Metabolic and Vascular Complications. Journal of Diabetes Research. 2017;2017:1-12.
94. Denley A, Bonython ER, Booker GW, Cosgrove LJ, Forbes BE, Ward CW and Wallace JC. Structural determinants for high-affinity binding of insulin-like growth factor II to insulin receptor (IR)-A, the exon 11 minus isoform of the IR. Mol Endocrinol. 2004;18 10:2502-12.
95. Sims EK, Carr ALJ, Oram RA, Dimeglio LA and Evans-Molina C. 100 years of insulin: celebrating the past, present and future of diabetes therapy. Nature Medicine. 2021;27 7:1154-64.
96. De Meyts P. Insulin/receptor binding: the last piece of the puzzle? What recent progress on the structure of the insulin/receptor complex tells us (or not) about negative cooperativity and activation. Bioessays. 2015;37 4:389-97.
97. Jumper J, Evans R, Pritzel A, Green T, Figurnov M, Ronneberger O, et al. Highly accurate protein structure prediction with AlphaFold. Nature. 2021;596 7873:583-+.
98. Grimmelikhuijzen CJ and Hauser F. Mini-review: the evolution of neuropeptide signaling. Regul Pept. 2012;177 Suppl:S6-9.
99. Robinson SD, Li Q, Bandyopadhyay PK, Gajewiak J, Yandell M, Papenfuss AT, et al. Hormone-like peptides in the venoms of marine cone snails. Gen Comp Endocrinol. 2017;244:11-8.
100. Ebberink RHM, Smit AB and Vanminnen J. The Insulin Family - Evolution of Structure and Function in Vertebrates and Invertebrates. Biol Bull. 1989;177 2:176-82.
101. UniProt C. UniProt: a worldwide hub of protein knowledge. Nucleic Acids Res. 2019;47 D1:D506-D15.
102. Floyd PD, Li L, Rubakhin SS, Sweedler JV, Horn CC, Kupfermann I, et al. Insulin prohormone processing, distribution, and relation to metabolism in Aplysia californica. J Neurosci. 1999;19 18:7732-41.
103. Krogh A, Brown M, Mian IS, Sjolander K and Haussler D. Hidden Markov models in computational biology. Applications to protein modeling. J Mol Biol. 1994;235 5:1501-31.
104. Laht S, Koua D, Kaplinski L, Lisacek F, Stocklin R and Remm M. Identification and classification of conopeptides using profile Hidden Markov Models. Biochim Biophys Acta. 2012;1824 3:488-92.
105. Mirabeau O, Perlas E, Severini C, Audero E, Gascuel O, Possenti R, et al. Identification of novel peptide hormones in the human proteome by hidden Markov model screening. Genome Res. 2007;17 3:320-7.
106. Gacesa R, Barlow D and Long PF. Machine learning can differentiate venom toxins from other proteins havingnon-toxic physiological functions. PeerJ Comput Sci. 2016;2:e90.
107. Madera M and Gough J. A comparison of profile hidden Markov model procedures for remote homology detection. Nucleic Acids Res. 2002;30 19:4321-8.
108. Potter LR, Yoder AR, Flora DR, Antos LK and Dickey DM. Natriuretic Peptides: Their Structures, Receptors, Physiologic Functions and Therapeutic Applications. cGMP: Generators, Effectors and Therapeutic Implications. Springer Berlin Heidelberg; 2009. p. 341-66.
109. Sangaralingham SJ, Kuhn M, Cannone V, Chen HH and Burnett JC. Natriuretic peptide pathways in heart failure: further therapeutic possibilities. Cardiovasc Res. 2023;118 18:3416-33.
110. Fu H, Zhang J, Cai Q, He Y and Yang D. Pleiotropic Roles of Atrial Natriuretic Peptide in Anti-Inflammation and Anti-Cancer Activity. Cancers. 2022;14 16:3981.
111. Bystrova OA, Parfenov VN and Martynova MG. Atrial natriuretic peptide in the granular cells of the snail heart. Tsitologiia. 2002;44 2:115-9.
112. Koch TL, Robinson SD, Salcedo PF, Chase K, Biggs J, Fedosov AE, et al. Prey Shifts Drive Venom Evolution in Cone Snails. Mol Biol Evol. 2024;41 8.
113. Dutertre S, Jin AH, Kaas Q, Jones A, Alewood PF and Lewis RJ. Deep venomics reveals the mechanism for expanded peptide diversity in cone snail venom. Mol Cell Proteomics. 2013;12 2:312-29.
114. Dutt M, Dutertre S, Jin AH, Lavergne V, Alewood PF and Lewis RJ. Venomics Reveals Venom Complexity of the Piscivorous Cone Snail, Conus tulipa. Mar Drugs. 2019;17 1.

115. Himaya SW, Jin AH, Dutertre S, Giacomotto J, Mohialdeen H, Vetter I, et al. Comparative Venomics Reveals the Complex Prey Capture Strategy of the Piscivorous Cone Snail Conus catus. J Proteome Res. 2015;14 10:4372-81.
116. Jin AH, Dutertre S, Dutt M, Lavergne V, Jones A, Lewis RJ and Alewood PF. Transcriptomic-Proteomic Correlation in the Predation-Evoked Venom of the Cone Snail, Conus imperialis. Marine Drugs. 2019;17 3.
117. Jin AH, Dutertre S, Kaas Q, Lavergne V, Kubala P, Lewis RJ and Alewood PF. Transcriptomic messiness in the venom duct of Conus miles contributes to conotoxin diversity. Mol Cell Proteomics. 2013;12 12:3824-33.
118. Jin AH, Vetter I, Himaya SW, Alewood PF, Lewis RJ and Dutertre S. Transcriptome and proteome of Conus planorbis identify the nicotinic receptors as primary target for the defensive venom. Proteomics. 2015;15 23-24:4030-40.
119. Prashanth JR and Lewis RJ. An efficient transcriptome analysis pipeline to accelerate venom peptide discovery and characterisation. Toxicon. 2015;107 Pt B:282-9.
120. Prashanth JR, Dutertre S, Jin AH, Lavergne V, Hamilton B, Cardoso FC, et al. The role of defensive ecological interactions in theevolution of conotoxins. Mol Ecol. 2016;25 2:598-615.
121. Robinson SD, Li Q, Lu AP, Bandyopadhyay PK, Yandell M, Olivera BM and Safavi-Hemami H. The Venom Repertoire of Conus gloriamaris (Chemnitz, 1777), the Glory of the Sea. Marine Drugs. 2017;15 5.
122. Guo Q, Huang M, Li M, Chen J, Cheng S, Ma L and Gao B. Diversity and Evolutionary Analysis of Venom Insulin Derived from Cone Snails. Toxins. 2024;16 1:34.
123. Bao SJ, Xie DL, Zhang JP, Chang WR and Liang DC. Crystal structure of desheptapeptide(B24-B30)insulin at 1.6 angstrom resolution: Implications for receptor binding. P Natl Acad Sci USA. 1997;94 7:2975-80.
124. Owens DR. New horizons--alternative routes for insulin therapy. Nat Rev Drug Discov. 2002;1 7:529-40.
125. Mirdita M, Schütze K, Moriwaki Y, Heo L, Ovchinnikov S and Steinegger M. ColabFold: making protein folding accessible to all. Nature Methods. 2022;19 6:679-+.
126. Frickey T and Lupas A. CLANS: a Java application for visualizing protein families based on pairwise similarity. Bioinformatics. 2004;20 18:3702-4.
127. Gabler F, Nam SZ, Till S, Mirdita M, Steinegger M, Soding J, et al. Protein Sequence Analysis Using the MPI Bioinformatics Toolkit. Curr Protoc Bioinformatics. 2020;72 1:e108.

Manuscript

Click here to access/download;Manuscript;Manuscript including figures_revised_tracked.docx

PeptideMiner – Neuropeptide Discovery across the Animal Kingdom

Helen C. Mendel[1,a], Gene Hopping[1,a], Eivind A. B. Undheim[2], Johannes Zuegg[1], Richard J. Lewis[1], Briony E. Forbes[3], Quentin Kaas[1*], Markus Muttenthaler[1,4*]

Formatted: Superscript

[1.] Institute for Molecular Bioscience, The University of Queensland, Brisbane, Australia

[2.] Centre for Ecological and Evolutionary Synthesis, Department of Biosciences, The University of Oslo, Oslo, Norway.

[3.] Discipline of Medical Biochemistry, Flinders Health and Medical Research Institute, Flinders University, Adelaide, Australia.

[4.] Institute of Biological Chemistry, Faculty of Chemistry, University of Vienna, Vienna, Austria.

[a] Contributed equally to the manuscript.

[*] Corresponding authors: Markus Muttenthaler and Quentin Kaas

Email: m.muttenthaler@uq.edu.au, quentin.kaas@syngenta.com

Author Contributions: H.C.M., Q.K., and M.M. designed the research project. H.C.M., G.H., B.E.F., E.A.B.U., J.Z., and Q.K. performed experiments. H.C.M., Q.K., and M.M. analyzed the data. H.C.M., G.H., and M.M. wrote the paper.

Competing Interest Statement: The authors declare no competing financial interest.

Classification: Biological Sciences; Biophysics and Computational Biology

Keywords: Neuropeptide; venom; transcriptomics; natriuretic peptide; insulin.

Abstract

Neuropeptides ~~are~~ represent the largest and most diverse class of cell-to-cell signaling molecules, holding important roles in animal physiology and behavior. They are evolutionarily ancient and ~~ubiquitously present~~widely distributed across the animal kingdom. Although over 200 neuropeptides have been identified, only a small fraction has been functionally characterized. ~~Over 200 human neuropeptides are known, yet only a handful are well understood.~~ A recognized bottleneck is ~~the~~ the lack of ~~probes~~ effective tools to study their ~~function~~ biological roles and therapeutic potential. Interestingly, ~~N~~neuropeptide-like ~~homologs~~peptides are also ~~observed~~ found in animal venoms, where they ~~can facilitate~~contribute to prey capture or defensive strategies. Mapping neuropeptide families across the animal kingdom is challenging due to their high sequence divergence and short mature peptide sequences. ~~Therefore~~To address this, we developed PeptideMiner, a search tool that employs profile-hidden Markov models (profile-HMM) ~~that allow~~for family-specific peptide discovery. PeptideMiner was systematically validated and benchmarked ~~to~~ against existing methods, demonstrating its ~~efficiency~~superior performance. By ~~A~~applying PeptideMiner to several venom transcriptomes including 24 previously unpublished datasets~~,~~ we ~~discovered~~ identified 10 novel natriuretic peptides ~~sequences~~ from ~~evolutionarily~~ distantly related ~~organisms~~ species and 57 novel insulin-like sequences from marine predatory cone snails. Chemical synthesis ~~followed by~~and structure-activity relationship studies of newly identified conoinsulins at ~~the~~ human insulin receptors emphasize~~d~~s the value of ~~this~~ our approach in ~~providing~~ elucidating ~~new insights into~~ ligand-receptor interactions and ~~identifying~~ discovering new pharmacological probes and therapeutic leads. PeptideMiner ~~is~~ offers a powerful platform ~~a powerful tool~~ for discovering new bioactive peptides and family-specific ~~homologs~~analogs, accelerating both natural product discovery and evolutionary ~~analyses~~research.

Introduction

Animal venoms represent an invaluable natural source for developing pharmacological probes and therapeutic agents [1-3]. Comprising intricate and diverse mixtures of bioactive peptides, venoms have evolved to serve the dual purpose of defense and prey immobilization. While venom peptides are primarily recognized for their ability to target ion channels to cause pain or paralysis in prey, they also exhibit efficacy against other target classes, including G protein-coupled receptors (GPCRs) [4], transporters [5], and soluble protein targets such as thrombin [6] and fibrinogen [7]. By identifying, isolating, synthesizing, and pharmacologically characterizing these structurally well-defined venom peptides, researchers have unveiled highly potent and selective molecules that have revolutionized ion channel and pain research [8-10]. Notably, this approach led to the FDA approval of ziconotide (Prialt), a venom peptide derived from the marine predatory cone snail of the genus *Conus*, as a peptide drug for managing severe chronic pain [11]. Another significant example was the discovery of exenatide, a venom peptide derived from the saliva of the Gila monster, which was approved as a peptide drug for treating type II diabetes (Byetta) [12], among others [3].

In addition to the well-studied venom peptides targeting ion channels, there is growing evidence of the presence of neuropeptides ~~homologs~~ in animal venoms, albeit with limited characterization and unclear roles in envenomation. It is hypothesized that neuropeptide signaling systems and their corresponding membrane receptors could represent attractive targets for envenomation due to their crucial physiological roles and remarkable conservation across prey and predators. Notable examples of such venom peptides encompass conopressins (vasopressin, oxytocin) [13], contulakins (neurotensin) [14], conoinsulins (insulin) [15], conorfamides (RF-amide) [16], and natriuretic peptides [17].

Neuropeptides serve as signaling molecules secreted by neurons, orchestrating a wide array of functions encompassing fluid homeostasis, reproduction, appetite control, memory, learning, and complex social behavior [18, 19]. These ancient signaling systems are widely distributed throughout the animal kingdom, with at least 30 neuropeptide signaling systems traceable to a common bilaterian ancestor [20]. Despite their vital physiological roles, our understanding of these signaling systems in animals and humans remains limited, primarily due to a scarcity of pharmacological probes beyond the often ~~–~~nonspecific endogenous ligands required for dissecting the complex pathways and receptor subtypes.

Venoms, containing many diverse ~~putative neuropeptide homologs~~ neuropeptide-like peptides with unique pharmacological profiles, hold immense promise for transforming neuropeptide research and facilitating the discovery of therapeutic leads for human diseases [19]. The systematic discovery of neuropeptide-like venom peptides is, however, challenging due to a lack of methodologies capable of reliably identifying neuropeptide

families across evolutionarily distant species.

Traditionally, venom research relied on venom collection, bioactivity-guided fractionation, and mass spectrometry. However, recent advances in proteomics, bioinformatics, and nucleotide sequencing [21] have reshaped the approach to studying venoms, culminating in an approach termed integrated venomics [22-25]. Integrated venomics involves generating venom gland transcriptomes that encompass all venom peptide sequences, including their precursors, and subsequently using bioinformatics to match these sequences against the crude venom proteome as well as annotated peptides and proteins in databases such as UniProt and NCBI. Currently, this matching process relies heavily on the NCBI protein-protein Basic Local Alignment Search Tool (BLASTp), which performs well for large homologous sequences but struggles with the short length and high precursor sequence diversity across divergent species characteristic of neuropeptides [26, 27]. Alternative probabilistic models exist, which parameterize complex position-specific models and are expected to be more effective in detecting distant homology [28-30]. One such model is the profile-hidden Markov model (profile-HMM), a probabilistic model of multiple sequence alignments represented as a series of amino acid-emitting *states*, with probabilistic connections between states that account for fully conserved segments, insertions, and deletions [31, 32].

A bioinformatic pipeline or search algorithm capable of reliably identifying homologous neuropeptides across a wide range of species would be of high value, facilitating the systematic mapping and characterization of neuropeptide-like venom peptides. Such an advancement would enhance our understanding of the evolutionary significance and distribution of neuropeptide-like venom peptides, ultimately accelerating the discovery of new pharmacological tools and therapeutic agents.

In this study, we present PeptideMiner, a neuropeptide discovery pipeline that utilizes neuropeptide family-specific profile-HMMs to efficiently identify neuropeptide sequences across different databases and sources. To highlight the application scope of PeptideMiner, we used it to identify new natriuretic and insulin-like peptides from venom gland transcriptomes of ~~different~~ a broad range of ven~~e~~omous species, including 24 previously unpublished transcriptomes, and benchmarked it against existing methods of sequence homology searches. Finally, we chemically synthesized and tested newly identified insulin-like venom peptides against the human insulin receptor to demonstrate the translational potential of this new computational discovery pipeline.

Results

PeptideMiner Overview

PeptideMiner is a versatile new tool for neuropeptide discovery that integrates a suite of bioinformatic tools, including HMMER3 [33], SignalP [34], FASTA36 [35], BLAST [36], and SQLite3 [37] to search, filter, and annotate amino acid sequences. It is ~~written~~ implemented in Python, a high-level programming language commonly used for bioinformatic pipelines due to its rapid implementation and seamless integration of multiple bioinformatic tools [38, 39]. By employing peptide/protein family-specific profile-HMMs, PeptideMiner ~~can~~ is ~~be~~ able ~~used~~ to efficiently search translated transcriptome or genome sequences for a diverse range of amino acid sequences, including short and diverse (neuro)peptides as well as longer proteins.

Generation of precursor and mature profile-hidden Markov models

Neuropeptide sequences of interest were submitted as a query to the PSI-BLAST (Position-Specific Iterative Basic Local Alignment Tool) against the NCBI non-redundant database [36]. The hits from the first iteration generated a position-specific scoring matrix that was used to search the database for sequences matching the conservation pattern specified by the matrix. The process was iterated 10 times or until no more new sequences were detected. This preliminary training set of sequences was aligned using MUSCLE [40]~~(135)~~ or ClustalO [41]~~[40]~~ and trimmed in Jalview [42]~~[41]~~. Each initial training set was trimmed to generate two training sets: in one, the sequences were trimmed to the whole precursor peptide, and in the other, to the mature peptide. Each training set was used to build a *precursor*-profile-HMM and a *mature*-profile-HMM using *hmmbuild* from the HMMER 3.0 package [43]~~[42]~~. A similar number of reads were observed irrespective of whether the *precursor*-profile- or *mature*-profile-HMM was used, indicating the profile-HMM did not affect the efficiency or depth of the search (Figure S1). For this work, the products of both *precursor*- and *mature*-profile-HMMs were combined, and duplicate product sequences were removed.

PeptideMiner Workflow

To initiate the PeptideMiner workflow (Figure 1), users must first input one or more profile-HMMs of neuropeptide families of interest, as well as the database of amino acid sequences that should be searched as ~~fasta~~ FASTA files. *Hmmsearch* from the HMMER3 package is then used to search the database using the profile-HMMs. *Hmmsearch* results are stored in an SQLite database (Figure S2). The protein-coding sequences (CDS) of the hypothetical neuropeptide precursors are predicted ~~using a Python script that~~by extract~~s~~ings the sequence between the methionine 'M' start codon and the stop codon or, if there is no stop codon, the end of the contig. SignalP [34] is then used to identify the signal peptides of the predicted CDS and, if present, excised

Field Code Changed

Field Code Changed

Field Code Changed

from the precursor sequence to facilitate mature peptide identification. FASTA36 [35] is used to align all the processed CDS to a manually curated list of known mature peptides, taken from the UniProt database, of the neuropeptide family of interest, followed by a precursor cleavage site prediction using the algorithm from the ConoServer annotation pipeline [44][43]. The predicted mature peptides for all sequences from the same transcriptome are then compiled, and duplicate mature peptides are removed. BLASTp is then used to annotate the predicted mature peptides by homology using a list of known neuropeptide amino acid sequences.

Field Code Changed

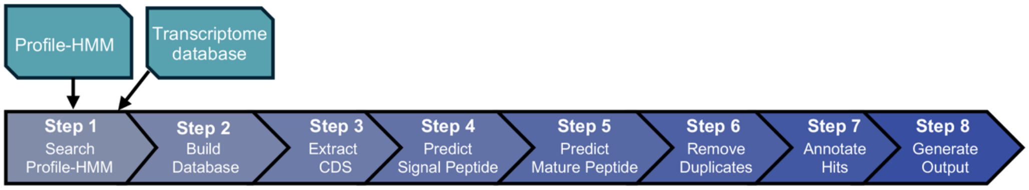

Figure 1. PeptideMiner pipeline. PeptideMiner uses *hmmsearch* in the HMMER 3 package to search through the transcriptomes with the profile-HMMs and saves the output to an SQLite database. The protein-coding sequences (CDS) are extracted from the hits and submitted to SignalP to determine whether a signal peptide is present. Next, mature peptides are predicted and compared to a list of known sequences belonging to the neuropeptide family of interest. The final output consists of a list of precursors, the transcriptomes, predicted mature peptides, and known sequences they are most similar to. The user can specify the minimum CDS length required, the signal peptide cut-off, the minimum length of the signal peptide, the FASTA36 E-value cut-off, and the minimum and maximum length of the mature peptide.

PeptideMiner Performance Evaluation

To assess the pipeline performance, a 10-fold cross-validation analysis was conducted using a test database consisting of 300,000 randomly selected sequences from various eukaryotic species acquired from the UniProtKB database [45][44]. This database was chosen to provide a comprehensive representation of different species. For the evaluation, the natriuretic peptide and insulin neuropeptide families were used as representative model systems (Figure 2A,B). These neuropeptide families were selected due to their wide-ranging diversity, extensive literature, and presence in vertebrates and invertebrates, making them well-represented in the UniProtKB database. For each neuropeptide family, a negative dataset was created from the dataset by

Field Code Changed

removing all known sequences for the respective neuropeptide family (885 natriuretic peptides and 2,660 insulin sequences).

The pipeline was able to precisely identify hormone peptides and non-hormone peptides, as evidenced by the Matthews correlation coefficient (MCC) of 0.99 for the natriuretic peptide family and 0.93 for the insulin neuropeptide family (Table S1). Moreover, the pipeline was very sensitive, with a true positive rate (TPR) of 0.99 for both peptide families. Notably, no false positives were detected for the natriuretic peptide family, and the false positive rate (FPR) for insulin was extremely low at 1.3 x 10^{-4}.

PeptideMiner was then benchmarked against the widely used ~~homology~~ sequence similarity search algorithm BLASTp, using the sequences of human natriuretic peptide A and human insulin. Both human precursor proteins of these neuropeptides were queried against the same database employed for the 10-fold cross-validation using protein-protein BLAST (BLASTp, Figure 2, Table S2). PeptideMiner outperformed BLASTp for the insulin peptide family and was marginally better for the natriuretic peptide family. Specifically, BLASTp exhibited an MCC of only 0.76 and a TPR of 0.58, whereas PeptideMiner achieved an MCC of 0.93 and a TPR of 0.99, indicating an overall superior performance of PeptideMiner. Notably, both BLASTp and PeptideMiner demonstrated negligible FPR for both peptide families.

Formatted: Font: 6 pt

Formatted: Centered

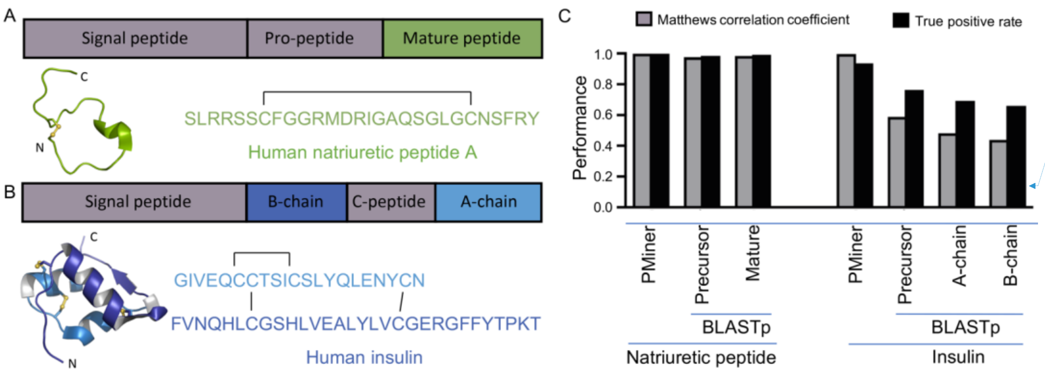

Figure 2. PeptideMiner performance comparison with BLASTp using human insulin and natriuretic peptide A as representative models. **A.** Representation of the precursor protein organization, 3D structure (PDB:7brj), and mature peptide sequence and disulfide bond connectivity of human natriuretic peptide A. **B.** Representation of the precursor protein organization, 3D structure (PDB:3w7y), and mature peptide

sequence and disulfide connectivity of human insulin. **C.** Results of the 10-fold cross-validation performance comparison of PeptideMiner (PMiner) versus BLASTp from a library of 300,000 random sequences (Table S2) based on Matthews correlation coefficient and true positive rates. In panels A and B, disulfide bonds are depicted as black lines in the peptide sequence and as yellow balls and sticks in the 3D structures.

Discovery of novel natriuretic peptides

Natriuretic peptides have variable lengths, but all display a 17-amino-acid loop cyclized by a disulfide bridge [46]~~[45]~~. These peptides play crucial roles in renal functions, cardiovascular system homeostasis, endothelial cell proliferation, and sympathetic outflow, among other physiological processes [47]~~[46]~~. In mammals, there are three main members of the natriuretic peptide family: atrial natriuretic peptide (ANP), B-type natriuretic peptide (BNP), and C-type natriuretic peptide (CNP). NPR-A serves as the primary receptor for ANP and BNP, while NPR-B acts as the primary receptor for CNP. All three natriuretic peptides bind to NPR-C, which primarily functions as a clearance receptor [46]~~[45]~~. By contrast, knowledge about invertebrate natriuretic peptide signaling systems is limited. Some evidence for invertebrate natriuretic peptides has been found in the hearts of oysters, blue crabs, and the earthworm *Lumbricus terrestis* [48, 49]~~[47, 48]~~. Natriuretic peptide receptors have also been identified in insects, crustaceans, arachnids, mollusks, and even cnidarians, although research on these systems is scarce. Natriuretic peptides are commonly observed in snake venoms, where they affect the prey's cardiovascular system to induce hypotension [50-52]~~[49-51]~~. Furthermore, natriuretic peptides have been observed in the venoms of the platypus *Ornithorhynchus anatinus*, the scorpion *Tityus serrulatus*, the stone fish *Synanceia horrida*, and several lizard species [53-56]~~[52-55]~~.

Field Code Changed

Field Code Changed

Field Code Changed

Field Code Changed

Field Code Changed

Field Code Changed

In our search for natriuretic venom peptides across 49~~62~~ species (Table S3), we identified 11 precursors that displayed the characteristic natriuretic peptide motif C-X_{15}-C (Figure S3). These precursors were identified in snakes (*Micrurus lemniscatus carvalhoi* with three precursors and *Naja Kaouthia* with three precursors), jellyfish (*Chironex fleckeri* with two precursors), leech *(Hirudo nipponia* with one precursor), centipede (*Scolopendra morsitans* with one precursor), and stone fish (*Synanceia horrida* with one precursor) (Figure 3). The natriuretic peptide Sh-NP from *Synanceia horrida* was recently independently identified at the nucleic acid level [56]~~[55]~~. The remaining predicted mature peptides have not been previously described from these species and are considered novel putative natriuretic peptides. While the 15 amino acids between the two cysteine residues show relative conservation, the N- and C-terminal tails exhibit considerable variation in length and composition (Figure 3).

Field Code Changed

Natriuretic peptides in snakes

The three precursors of the South American coral snake *Micrurus lemniscatus carvalhoi* (Mlc) contain two predicted mature natriuretic peptides, Mlc-NP-I and Mlc-NP-II (Figure 3). Natriuretic peptides in snake venoms are hypothesized to contribute to the rapid loss of consciousness of prey [51, 57]~~[50, 56]~~. Both exhibit long N-terminal tails (10 residues) and C-terminal tails (14 and 12 residues, respectively), typical of elapid natriuretic peptides. Mlc-NP-I and Mlc-NP-II are highly similar to known natriuretic peptides Mc-NP (both

Field Code Changed

98% identity) from the painted coral snake *Micrurus corallinus* and the Uruguayan coral snake *Micrurus altirostris* Ma-NP (75% and 95% identity, respectively) [58, 59][57, 58]. Mlc-NP-I differs only by one amino acid (Gly^{10} vs. Cys^{10}; residue numbering indicates the position within the conserved cysteine residues for ease of comparison) from Mc-NP. Gly^{10} is highly conserved, and the role and impact of the cysteine substitution in Mc-NP is unclear.

In the case of the monocled cobra *Naja kaouthia* (Nk), the three precursors are predicted to produce two mature peptides Nk-NP-I and Nk-NP-II, with only Nk-NP-II being previously described (Figure 3) [60][59]. Similar to the Mlc-NPs, Nk-NP-I has a five-residue N-terminal tail and a 16-residue C-terminal tail. Interestingly, the C-terminal tail contains two additional cysteine residues in a C-X_1-C pattern, suggesting a natriuretic peptide with an additional disulfide bond. The significance of this is not yet known. By contrast, Nk-NP-II has a short N-terminal tail (five residues) and no C-terminal tail, which is more typical of CNPs such as those found in Viperid snake venom [51, 61][50, 60]. Nk-NP-II shares an identical sequence to a transcript annotated as waglerin peptide 1, identified in the venom transcriptome of the Bornean-keeled green pit viper, *Tropidolaemus subannulatus* [60][59]. The waglerins are lethal peptides identified in the venom of *Tropidolaemus wagleri* [62][61] targeting nicotinic acetylcholine receptors [63][62]. This transcript contains two peptides with the characteristic Cys-X_3-Cys motif of the waglerin peptides in addition to a natriuretic peptide Cys-X_{15}-Cys motif, identified by PeptideMiner. The encoding of waglerins and CNPs as multi-domain precursor proteins has previously been observed in viperid venom [64][63], but was not identified in this particular elapid transcript [60][59]. Nk-NP-II also displays high similarity to the Okinawa habu pit viper *Protobothrops flavoviridis* Pf-CNP (91% identity) but has a Met^2Gly and a Leu^9His substitution, removing a positive charge. Nk-NP-II is the first reported CNP observed in an elapid venom.

Natriuretic peptides in jellyfish

Two natriuretic peptide precursors were identified in the jellyfish *Chironex fleckeri* (Cf), which contain a different but closely related predicted mature natriuretic peptide: Cf-NP-I and Cf-NP-II (Figure 3). These venom peptides exhibit less than 50% identity with any known natriuretic peptides but share several conserved residues in the C-X_{15}-C motif, including Ile^6, Asp^7, Arg^8, Ile^9, Ser^{13}, Leu^{15} and Gly^{14}. Notably, neither Cf-NP-I nor Cf-NP-II has a predicted signal peptide, and alignment with other natriuretic peptides suggests that these are partial precursors (Figure S3). This discovery marks the first evidence of a natriuretic peptide in a Cnidarian. Previous genome annotation identified natriuretic peptide receptors in a single species in this phylum, although this has yet to be confirmed at the protein level [65][64]. Thus, the identification of Cf-NP-I and Cf-NP-II supports the presence of natriuretic peptides in Cnidaria, indicating that the ancestral gene

Field Code Changed

~~encoding the natriuretic peptide precursor was present in the last common ancestor of cnidarians and bilateral animals 600-700 million years ago [65].~~

Natriuretic peptides in leeches

A single natriuretic peptide precursor was discovered in the leech *Hirudo nipponia*, which gives rise to a predicted 42-amino-acid-long natriuretic peptide, Hn-NP-I. Although Hn-NP-I has low similarity to known natriuretic peptides, it contains five of the most conserved amino acids within the C-X$_{15}$-C motif, including Phe^{2}, Arg^{8}, Ile^{9}, Gly^{10} and Ser^{13}, and we therefore consider this a ~~putative~~ natriuretic peptide-like ~~homolog~~sequence, representing the first of its kind in the salivary gland of an annelid. While annelids possess the natriuretic peptide signaling system, no endogenous ligands have been reported [49]~~[48]~~.

Natriuretic peptides in centipedes

In the centipede *Scolopendra morsitans*, a single natriuretic precursor was identified, giving rise to a predicted 27-residue-long natriuretic peptide, Sm-NP-I. Of the 15 amino acids within the C-X$_{15}$-C motif, 7 are conserved in Sm-NP-I. Furthermore, Sm-NP-I possesses a C-terminal tail but lacks an N-terminal tail. This marks the first observation of a natriuretic peptide in the venom of a centipede. Notably, natriuretic peptides in arthropod venoms are rare, with Ts-NP from the scorpion *Tityus serrulatus* being the only previously reported arthropod venom natriuretic peptide [61]~~[60]~~. Sm-NP-I and TsNP exhibit notable differences, with their mature peptides sharing an observed identity of 55%~~ identity~~ [53]~~[52]~~. Unlike Sm-NP, TsNP lacks a C-terminal tail but possesses an N-terminal tail [61]~~[60]~~. In humans, residues important for binding NP receptors lie within the intramolecular ring formed by the disulfide bond. However, in human ANP, the C-terminal tail is additionally required for binding to NPR-A. Thus, the N- and C-terminal tails of natriuretic peptides could be important for modulating binding and selectivity.

Field Code Changed

Field Code Changed

Field Code Changed

Field Code Changed

A

Species	Name	Mature natriuretic peptide	Length	UniProt
Homo sapiens	ANP	----SLRRSSCFGGRMDRIGAQSGLGCNSFRY-----------	28	P01160
	BNP	-SPKMVQGSGCFGRKMDRISSSSGLGCKVLRRH-----------	32	P16860
	CNP	------LSKGCFGLKLDRIGSMSGLGC----------------	21	P23582
Micrurus lemniscatus carvalhoi	**Mlc-NP-I**	GLAKEALGDGCFGQRIDRIGNVSGMGCNHVRTDPAPTALAR---	41	
	**Mlc-NP-II**	GLAKEALGEGCFGNRIDRIGDVSGMGCNHKTPAPKAPLR-----	39	
Naja kaouthia	**Nk-NP-I**	-----GPPDGCFGLKLDHIGTMSGLGCNGGSK-PIPTLTPCGCL	38	
	**Nk-NP-II**	-----AGGKGCFGLKLDRIGSTSGLGC----------------	38	
Scolopendra morsitans	**Sm-NP-I**	---------GCAGTSLDRIGISSRQLCVSPTGICTV--------	27	
Hirudo nipponia	**Hn-NP-I**	IEVDEVGSDGCFSEELVRIGSWSDILCGMENLFKGLRAKLVG--	42	
Chironex fleckeri	**Cf-NP-I**	------MSSTCRNEFIDRISNTSMLGCSCFKSSLNAMK------	32	
	**Cf-NP-II**	------MSSTCRNEFIDRISNTSILGCSCFKSSLNAMK------	32	
Synanceia horrida	**Sh-NP**	--GASTSRSGCFGHKMDRIGTISGMGC----------------	25	
Micrurus altirostris	Ma-NP	GLAKEALGEGCFGNRIDRIGDVSGMGCNRRTPAPKAPLR-----	39	F5CPE8
Micrurus corallinus	Mc-NP	GLAKEALGDGCFGQRIDRICNVSGMGCNHVRTDPAPTALAR---	41	P79799
Protobothrops flavoviridis	Pf-CNP	-------GKGCFGHKLDRIGSTSGLGC----------------	20	P0C7P5
Bothrops jararaca	Bj-CNP	-----GAAKGCFGLKLDRIGTMSGLGC----------------	22	Q6LEM5
Lachesis muta muta	Lmm-CNP	-----RVGDGCFGLKLDRIGSMSGLGC----------------	22	Q27J49
Tityus serrulatis	TsNP	------KLSGCFGFKLDRIGTMSGLGC----------------	21	P0DMD6

B

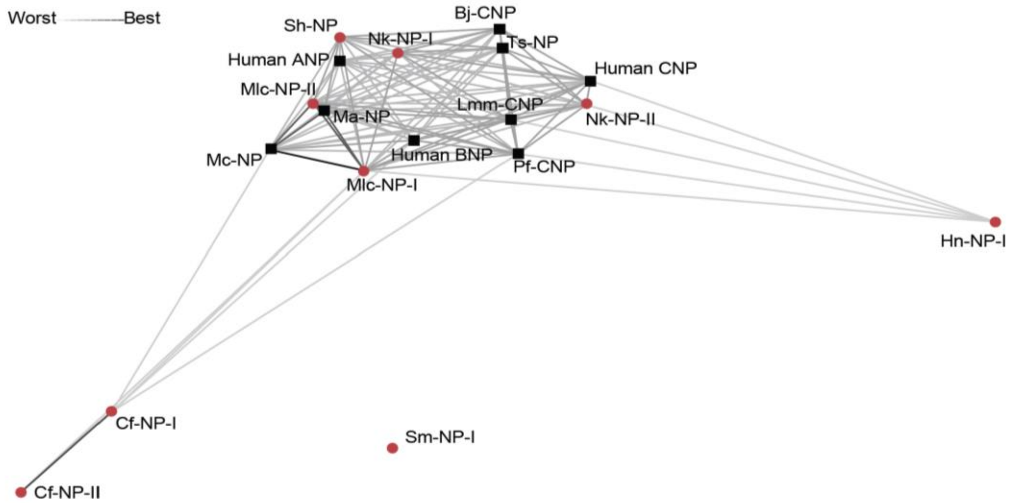

Figure 3. Sequence alignment and clustering of mature natriuretic peptides identified in venom gland transcriptomes aligned with human atrial natriuretic peptide (ANP). A. In the sequence alignment, cysteine residues are shaded yellow, N-terminal tail residues are colored green, and C-terminal tail residues gray. Novel natriuretic peptides identified in this study are bolded. Selected published natriuretic peptide sequences are shown in the lower panel for comparison. Sh-NP was novel when the study was conducted, but

was recently independently identified [56][55], validating our approach. B. Pairwise blast clustering of natriuretic peptides with human ANP, BNP, and CNP. Sequence similarity is denoted by line length and thickness. Human ANP sequences and previously reported NPs are represented as black squares, and novel sequences are red colored circles.

Field Code Changed

Discovery of novel Conus venom insulins

Insulin belongs to the insulin superfamily, which is highly conserved throughout the animal kingdom [66][66]. In humans, insulin is produced by the pancreatic β-cells in the Islets of Langerhans and plays a crucial role in regulating glucose homeostasis by facilitating glucose uptake into liver, fat, and skeletal muscle cells and suppressing gluconeogenesis in the liver [67, 68][67, 68]. Insulin comprises two peptide chains (A and B) connected by two interchain disulfide bonds (C^{7}_{A}-C^{7}_{B} and C^{20}_{A}-C^{19}_{B}) (Figure 2).

The 21-residue A-chain has an additional intrachain disulfide bond (C^{6}_{A}-C^{11}_{A}) and displays an α-helical secondary structure on the N-terminal and C-terminal ends. The 30-residue B-chain features a central α-helix and a β-strand [67][67] that together form a characteristic and evolutionarily conserved 3D structure (Figure 2) [69][69]. Mature insulin is derived from a single-chain precursor protein with a signal peptide, followed by the A-chain, C-peptide, and B-chain [67][67]. After translation in the rough endoplasmic reticulum as preproinsulin, the signal peptide is cleaved, and proinsulin is folded and sorted into immature secretory granules for C-peptide excision and subsequent processing into mature insulin [70][70]. At higher micromolar concentrations, insulin dimerizes and forms hexamers in the presence of zinc, enhancing its stability and preventing fibrilization [71, 72][71, 72]. Vertebrate-like conoinsulins share a cysteine framework similar to that of human insulins [73][73]. Conversely, molluskan insulin peptide (MIP)-like conoinsulins possess a cysteine motif resembling endogenous mollusk insulins with three A-B interchain disulfide bonds and one A intrachain disulfide bond (Figure 4) [74][74].

Marine predatory cone snails (genus *Conus*) are a group of venomous snails that utilize their venom for predation and defense [75-77][75-77]. The diet of cone snails varies and is broadly classified as fish-hunting (piscivorous), mollusk-hunting (molluscivorous), or worm-hunting (vermivorous). Fish-hunting cone snails have developed three distinct hunting strategies: "taser and tether", "net engulfment", and "strike and stalk" [78][78]. Their venoms comprise a wide diversity of bioactive peptides, and insulin-like peptides have been observed in several species [15, 79-85][15, 79-85]. In this study, PeptideMiner was used to search several 23 in-housepreviously unpublished venom transcriptomes for new insulin homologs. We searched 20 venom duct transcriptomes (including two *C. imperialis* and two *C. tulipa* transcriptomes), two nerve ring transcriptomes (*C. catus and C. textile*), and one salivary gland transcriptome (*C. geographus*) across 18 cone snail species (Table S3). After removing incomplete precursors, PeptideMiner identified 87 unique precursors from 16 of the 18 studied cone snail species (Figure 4, Figures S4-7). Neither *C. rattus*, nor *C. imperialis*, both worm hunters, returned any conoinsulin precursors. The predicted mature peptides (connected B- and A-chains

without the C-peptide) were manually assessed and cross-checked with cleavage sites predicted by NeuroPred [86][86] and identified sequences of conoinsulins at the protein level (e.g., Con-Ins G1, G3 [87][87], G121 [75][75] from *C. geographus*). Insulin precursor sequences are typically cleaved at dibasic cleavage sites (Arg-Arg or Lys-Arg) or, in some cases, at single basic sites (Arg) [88][88].

To assess whether any of the 87 conoinsulin precursors were novel, we compared them to known conoinsulin and insulin sequences obtained from UniProt and relevant literature. A BLASTp search revealed that only 15 of the 87 precursors were previously annotated, including 8 MIP-like insulins (Con-Ins Pl1, M2, Tx1, Tx2, Va1, Pl174, Ebr1a and Ebr1b) and 7 vertebrate-like insulins (Con-Ins G1b, G3, G121 Tu478, Tu479, Tu304, Tu073), yielding 72 novel conoinsulin precursors. Removal of identical sequences in the predicted B- and A-chains from these 72 precursors resulted in 57 novel and unique mature conoinsulins across 14 species, including 5 vertebrate-like, 33 MIP-like, and 19 ‘other’ conoinsulins that contained an odd number of cysteine residues (Table S4).

Of the 87 conoinsulin sequences discovered, 23 were vertebrate-like conoinsulins with the same three disulfide bond pattern as observed in human insulin (Figure S4). Vertebrate-like precursors from fish-hunters were predominantly identified in *C. geographus* and *C. tulipa*. 45 conoinsulins had 8 cysteine residues and were therefore categorized as MIP-like conoinsulins (Figure S5). 19 conoinsulins could not be classified as either vertebrate or molluskan conoinsulins as they featured an odd number of cysteine residues in the A- or B-chain (Figure S6), the significance of which is not known at present.

A single distinct conoinsulin precursor was identified in the salivary gland transcriptome of *C. geographus*, which we named G-SG-1 (Figure 4). It is similar to the MIP-like venom conoinsulins Im1 and *C. ebreaus* hormone insulin-related peptide [89][89]. It is distinct from both *C. geographus* signaling insulin (NR-G1) [85][85] and all other *C. geographus* venom conoinsulins with low precursor sequence conservation apart from the cysteine framework (Figure 4) [79][79]. G-SG-1 is the first conoinsulin identified from the salivary gland of a cone snail. Cone snail venom peptides were previously identified in the salivary glands of several species, including *C. pulicarius*,[90][90] *C. episcopatus*,[91][91] and *C. quercinus*.[92][92] Their role in envenomation remains unclear, but they have been proposed to function endogenously or enhance venom potency [92][92].

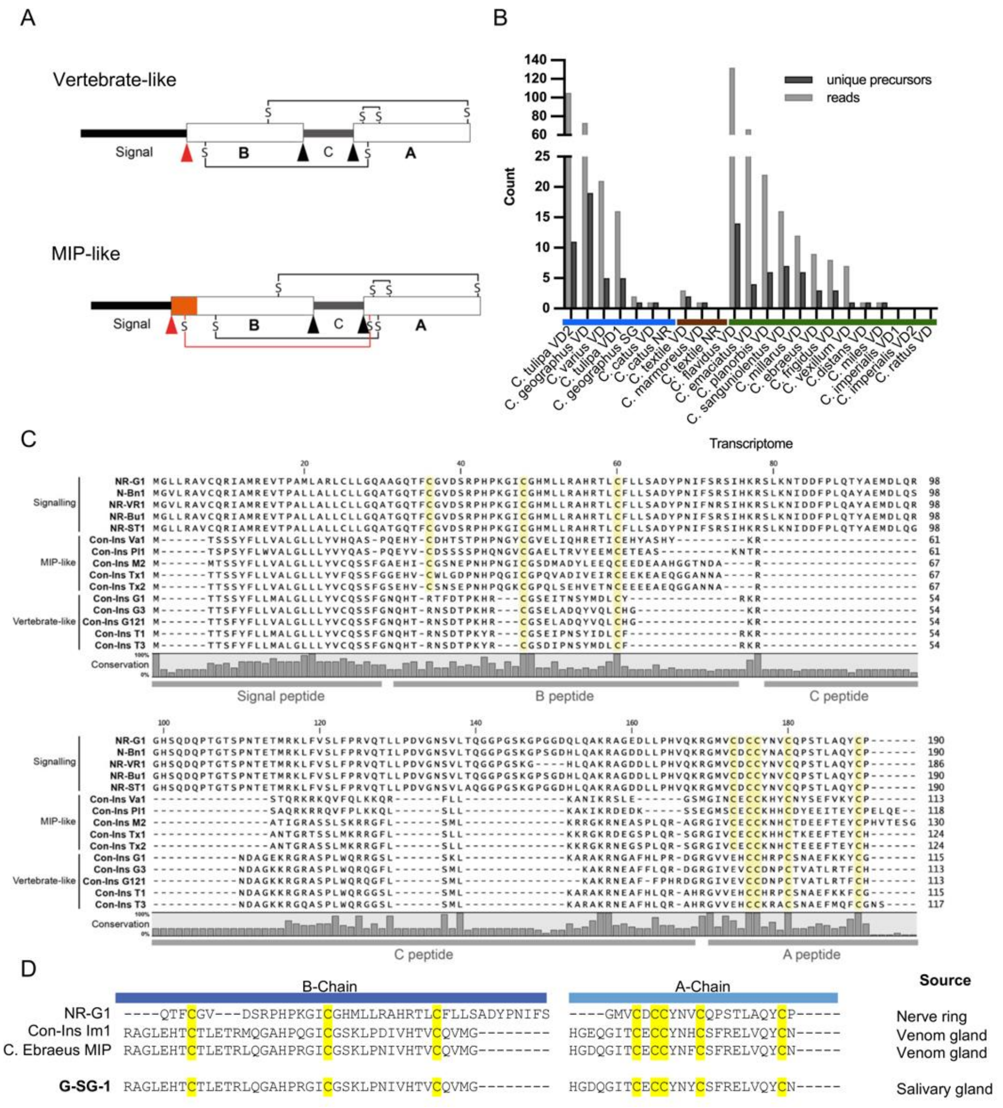

Formatted: Right

Figure 4. Discovery of novel conoinsulins. A. Schematic representation of the secondary structure of vertebrate-like and MIP-like conoinsulin precursors. All sequences contain a signal peptide, B-chain, C-peptide, and A-chain. Predicted proteolytic cleavage sites are indicated with black arrows, and predicted processing sites for removal of the signal sequence are indicated with red arrows. The orange box indicates

amino acid insertions, including the extra cysteine residue forming the 4th disulfide bond in MIP-like insulins. **B.** The total number of different reads per transcriptome (gray) and the number of unique precursors (black). Transcriptome tissues: VD, venom duct; SG, salivary gland. Prey of species is denoted by colored bars: fish-hunting (blue); mollusk-hunting (brown); worm-hunting (green). **C.** Sequence alignment of endogenous cone snail insulin precursors with selected venom insulins (for the complete list, see Figures S4-6). Cysteine residues are highlighted in yellow; the level of conservation is illustrated below the alignment. **D.** Sequence alignment of non-venom conoinsulins. A- and B-chains are labeled, and cysteine residues are shaded yellow. Sequences discovered in this work are bolded, and the tissue of their discovery is indicated on the right.

Pharmacological characterization of vertebrate-like conoinsulin at the human insulin receptor

As a proof-of-concept of using PeptideMiner to discover new venom neuropeptides with pharmacological or even therapeutic value, we synthesized several vertebrate-like conoinsulins using solid-phase peptide synthesis (SPPS), folded them, and tested them at the human insulin receptor-B (hIR-B), the most pharmaceutically relevant human insulin receptor subtype [93, 94]~~[93, 94]~~. A competition binding analysis to hIR-B of selected conoinsulins against Eu-labelled insulin was carried out. Results are expressed in IC_{50}, reflecting the concentration of the competing ligand (Con-Ins) displacing 50% of the specific binding of Eu-insulin.

Field Code Changed

We focused on vertebrate-like conoinsulins G3c, G4b, and G7 due to their overall structural similarity to human insulin and insulin's clinical significance in treating diabetes [95]~~[95]~~. Con-Ins G4b and G7 bound to hIR-B with nanomolar affinities (IC_{50} 35.4 nM and 124.2 nM, respectively), while no competition with human insulin was evidenced for Con-Ins G3c at up to 3 μM (Figure 5). The IC_{50} of Con-Ins G4b (35.4 nM) was similar to Con-Ins G3 (46.8 nM [80]~~[80]~~. Con-Ins G4b differs from Con-Ins G3 by a six-residue C-terminal extension of the B-chain, akin to human insulin, albeit with low sequence similarity. This extension has little effect on hIR-B binding. The main difference between Con-Ins G7 and Con-Ins G3 is a five-residue N-terminal extension, also displayed by human insulin. Remarkably, Con-Ins G7 showed potent hIR-B binding yet no improvements in affinity compared to human insulin. ~~Interestingly~~Notably, Con-Ins G3c differs from Con-Ins G3 only at a single residue in the B-chain (Gly_B^{10}Val), which completely abolishes hIR-B binding. This result highlights the importance of Gly_B^{10}, which is highly conserved within venom conoinsulins as well as across species in endogenous insulins and is identified as part of the hIR S1 binding site [96]~~[96]~~.

Field Code Changed

Field Code Changed

Field Code Changed

The three-dimensional structures of the three tested conoinsulins were predicted using AlphaFold2 [97]~~[97]~~ and compared to the crystal structure of human insulin (Figure ~~6 C E~~5B). Cα–root mean square deviation (Cα–RMSD) for all residues vs the human insulin crystal structure (PDB: 3wy7) were 0.8, 1.7, and 1.0 Å for G3c, G4b, and G7, respectively (Cα–RMSD for the AlphaFold2 predicted human insulin structure was 0.5 Å for comparison). These three conoinsulins are predicted to adopt the insulin-like fold, the most notable deviation being the elongation of the B-chain termini.

Field Code Changed

A	B-Chain	A-Chain	%ID compared to human insulin B-chain	%ID compared to human insulin A-chain
Human Insulin	-------FVNQHLC$_1$GSHLVEALYLVC$_2$GE-RGFFYTPKT	GIVEQC$_3$C$_4$TSIC$_5$SLYQLENYC$_6$N--		
**Con-Ins G3c**	-----NSDTPKHRC$_1$VSELADQYVQLC$_2$H*----------	GIVγVC$_3$C$_4$DNPC$_5$TVATLRTFC$_6$H--	25%	43%
**Con-Ins G4b**	-----NSDTPKHRC$_1$GSELADQYVYLC$_2$HGERNDA*----	GIVγVC$_3$C$_4$DNPC$_5$TVATLRTFC$_6$H--	23%	43%
**Con-Ins G5**	TNIPANSDTPKHRC$_1$GSELADQYVQLC$_2$H*----------	GIVγVC$_3$C$_4$DNPC$_5$TVATLRTFC$_6$H--	30%	43%
**Con-Ins G7**	TNIPATLILQKHRC$_1$GSELADQYVQLC$_2$H*----------	GIVγVC$_3$C$_4$DNPC$_5$TVATLRTFC$_6$H--	30%	43%
**Con-Ins T3b**	-----NSDTOKYRC$_1$GSDIPNSYMDLC$_2$F----------	GVVDHC$_3$C$_4$KRAC$_5$SNAEFMQFC$_6$GNS	20%	33%
Con-Ins G1	-----TFDTOKHRC$_1$GSγITNSYMDLC$_2$YR----------	GVVγHC$_3$C$_4$HRPC$_5$SNAEFKKYC$_6$*--	24%	45%
Con-Ins G3	-----NSDTPKHRC$_1$GSELADQYVQLC$_2$H*----------	GIVγVC$_3$C$_4$DNPC$_5$TVATLRTFC$_6$H--	30%	43%

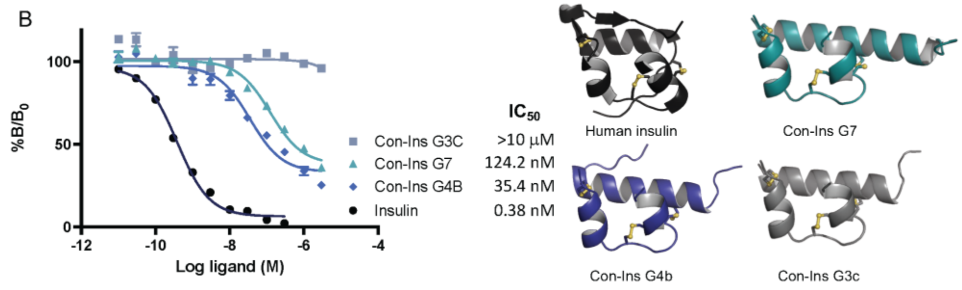

Figure 5. Sequence alignment and relative binding affinity of ~~synthesized~~ newly identified and synthesized conoinsulins to the human insulin receptor B (hIR-B). A. Amino acid sequence alignment of the A- and B-chains of selected conoinsulins and human insulin. **B.** Competition binding affinity to hIR-B between conoinsulins and Eu-labelled human insulin, and AlphaFold2 predicted three-dimensional structures of Con-Ins G7 (green), Con-Ins G4b (blue), and Con-Ins G3c (gray) are presented next to the human insulin crystal structure (black, PDB:3w7y). In panel A, the percentage amino acid sequence identity (%ID) to human insulin calculated by ClustalO [41]~~[40]~~ is displayed on the right for both the B- and A-chains. Predicted post-translational modifications are highlighted in red: O, 4-hydroxoproline; γ, 4-carboxyglutamate; *, C-terminal amidation. ~~Cysteine residues are highlighted in yellow, and the~~The cysteine connectivity for all sequences is C_1-C_4; C_2-C_6; C_3-C_5. Sequences discovered in this work are in bold. In panel B, results are expressed as a percentage of Eu-insulin bound without competing ligands (%B/B_0). Plotted values represent means +SEM (n=3, each comprising three technical replicates). IC_{50} is the concentration of the competing ligand (Con-Ins) displacing 50% of the specific binding of Eu-insulin.

Formatted: Indent: First line: 0"

Field Code Changed

Discussion

Neuropeptides are often referred to as the signaling molecules of life due to their ancient character and pivotal roles in various biological processes, including neurotransmission, inter- and intra-cellular signaling, and regulating complex behaviors. Their involvement spans a wide array of physiological functions, including pain perception, feeding behavior, stress and fear response, cardiovascular functions, fluid balance, and reproduction, amongst many others [19, 20, 98]~~[19, 20, 65]~~. Their evolutionary conservation across diverse species underscores this fundamental physiological importance, and their involvement in numerous diseases has attracted substantial scientific interest.

Field Code Changed

The high degree of conservation and physiological significance of neuropeptides may also explain the presence of neuropeptide-like peptides ~~homologs~~ in venoms, where they presumably contribute to prey capture or predator deterrence [99]~~[98]~~. The unexplored diversity of neuropeptides within venoms presents a unique opportunity to discover novel pharmacological tools and therapeutic leads. However, the efficient identification and mapping of neuropeptides using traditional search algorithms ~~has~~ ~~have~~has been challenging due to the short lengths and high sequence variations of these signaling molecules [26]. We, therefore, developed PeptideMiner, a highly efficient peptide search tool capable of overcoming these bottlenecks and facilitating the identification and mapping of neuropeptides across a wide range of species and databases.

Field Code Changed

PeptideMiner, an enhanced computational tool to efficiently search for neuropeptides

PeptideMiner is a highly versatile, robust, Python-based pipeline that harnesses peptide family-specific profile hidden Markov models (profile-HMMs) to effectively search translated transcriptomic, genomic, and proteomic data for neuropeptides or peptides in general. PeptideMiner ~~demonstrates~~ demonstrated exceptional performance with remarkably low false-~~-~~positive and false-~~-~~negative rates when tested against representative neuropeptide model systems, surpassing the widely utilized BLASTp method. Notably, PeptideMiner exhibits clear advantages, particularly evident in the insulin family, attributed to its extensive and divergent nature, with over 2,000 variants across the animal kingdom, including diverse mature insulins featuring additional interchain disulfide bonds in certain species [100, 101]~~[99, 100]~~. The methodological disparities and existing limitations of BLASTp in this context are anticipated to be intensified by the ongoing influx of new sequence data from diverse species, spurred by the advancements and widespread adoption of ‘omics’ technologies [79, 102]~~[79, 101]~~. Conversely, PeptideMiner can handle large datasets, sequence variations, and distant homology, thereby enabling more accurate and comprehensive neuropeptide identification across species. PeptideMiner also supports simultaneous searches across multiple species and is compatible with any peptide/protein database. Importantly, PeptideMiner is open access, written in Python, and integrates publicly available

Field Code Changed

Field Code Changed

bioinformatic tools, ensuring ease of access and user-friendliness for the broader scientific community. While primarily developed to advance the mapping of neuropeptide families across species and expedite the discovery of neuropeptides with sequence similarity and homologs with unique pharmacology or therapeutic potential, the open-access and user-friendly nature of PeptideMiner encourages its adoption by the scientific community for other applications.

The concept of profile-HMMs in computational biology emerged in the mid-90s [103][102] and has more recently found applications in studies involving peptides, including neuropeptides [104, 105][103, 104]. For example, profile-HMMs were employed in predicting and classifying the 62 conotoxin superfamilies [104][103]. In another instance, a profile-HMM based on biological processing signals of neuropeptides, encompassing the signal peptide, pro-peptide cleavage site, and extracellular peptide features, was utilized to identify novel bioactive peptides, including neuropeptides, in the human proteome [105][104]. However, this approach lacked specificity to neuropeptides and did not involve sequence homology. One further example is the use of small profile-HMMs known as 'tox-bits'. Combinations of 2-3 'tox-bits' could accurately discern toxins from non-toxin sequences in a machine-learning model [106]. However T, thishese approaches, however, h lacked specificity to neuropeptides and dido not all involve sequence homology.

By contrast, PeptideMiner specifically targets neuropeptides by constructing profile-HMMs using known precursor or mature peptide sequences from the neuropeptide family of interest. This neuropeptide-specific approach improves identification accuracy. Additionally, the neuropeptide sequences selected to build the seed alignment for the profile-HMM are not restricted to a specific taxonomic group, facilitating the detection of neuropeptide analogs with distant homology across evolutionarily diverse species. This capability is particularly suited for investigating neuropeptide evolution, identifying neuropeptides in uncharacterized species, and discovering highly divergent neuropeptides, such as those present in tissues with a high mutation rate, like venoms.

To ensure an effective search, thoughtful consideration needs to be given when constructing profile-HMMs [30, 103][30, 102]. Manual curation of the multiple sequence alignments is essential for profile-HMM construction [107][105], which can then be publicly shared. Neuropeptide families with a limited number of known peptides or peptides from a limited taxonomic range are at a disadvantage compared to larger families present in many taxonomic lineages. It is, therefore, important to regularly update the seed alignments and profile-HMMs with novel sequence information, a process that can be automated.

In addition to the insulin and natriuretic peptide profile-HMMs discussed and utilized in this study, we have included another three other three neuropeptide profile-HMMs for the neurohypophyseal, tachykinin, and

Field Code Changed

Field Code Changed

Field Code Changed

Field Code Changed

Field Code Changed

Field Code Changed

somatostatin families in the open-access PeptideMiner platform to facilitate profile-HMMs creation. While there are parameters of HMMER3 that can be adjusted for profile-HMM construction (*hmmbuild*) and searching profile-HMMs against the database (*hmmsearch*) [33], PeptideMiner already demonstrates exceptional performance, and further investigations into these parameters are not expected to enhance performance substantially.

Natriuretic peptide discovery expanded to new species

Natriuretic peptides regulate fluid balance, blood pressure, and cardiovascular homeostasis [108, 109]~~[106, 107]~~. In humans, ANP and BNP are predominantly produced in the heart's atria (while CNP is more widely expressed in endothelial cells) and released in response to elevated blood volume and pressure, causing vasodilation. They also promote urine production and inhibit sodium reabsorption, ultimately facilitating natriuresis [108]~~[106]~~. Furthermore, natriuretic peptides exhibit anti-inflammatory [110]~~[108]~~ and anti-fibrotic properties, rendering them promising candidates for treating heart failure, hypertension, and kidney diseases [109]~~[107]~~.

Field Code Changed

Field Code Changed

Field Code Changed

Field Code Changed

Natriuretic peptides have also been identified in animal venoms, likely owing to their cardiovascular effects for defense and prey capture. They are particularly abundant in snakes but have also been observed in the venoms of lizards, stonefish, platypuses, and scorpions [51, 53-56]~~[50, 52-55]~~. This study expanded our understanding of natriuretic peptide distribution in venomous animals, confirming that they are not limited to vertebrate venoms and underscoring their presence in diverse evolutionary lineages. Notably, natriuretic peptides were detected in the venom glands of the centipede *S. morsitans*, as well as in two previously unexplored phyla, namely the annelid *H. nippona* and the cnidarian *C. fleckeri* species. The identification of natriuretic peptides in the tentacles of *C. fleckeri* holds significance, as it provides further evidence of their presence in cnidarians beyond the genome annotation of the putative natriuretic peptide A receptor in the cnidarian *Thelohanellus kitauei* [65]~~[64]~~. The identification of Cf-NP-I and Cf-NP-II supports the presence of natriuretic peptides in Cnidaria, opening the possibility for convergence, or that the ancestral gene encoding the natriuretic peptide precursor was present in the last common ancestor of cnidarians and bilateral animals 600-700 million years ago [98]. N~~Interestingly, n~~o natriuretic peptides were identified in the transcriptomes of mollusk venom glands or salivary glands, despite evidence of the natriuretic peptide signaling system in gastropods such as *Crassostrea virginica* and *Helix pomatia* [48, 111]~~[47, 109]~~.

Field Code Changed

Field Code Changed

Field Code Changed

Conoinsulin discovery expands their diversity and underscores their role in envenomation

Insulin is a crucial peptide hormone regulating glucose levels and maintaining metabolic balance in humans. Its primary function lies in facilitating glucose uptake into cells, promoting its utilization for energy production

and storage. Dysregulation of insulin production or impaired insulin function can lead to metabolic disorders, notably diabetes [67, 68][67, 68]. Understanding the mechanisms underlying insulin action and the molecular interactions of ligands with the main human insulin receptor, hIR-B, is essential for developing more effective and safer therapeutic strategies for managing diabetes and related conditions.

Field Code Changed

Venoms represent a new natural source for insulin-like peptides, and particularly, the venom of the marine predatory cone snail seems rich in venom insulins, playing a role in prey capture [15, 84, 85][15, 84, 85]. There is evidence for prey-specific selection pressures in shaping the variation of peptides found in cone snail venom [112][110], and the presence of vertebrate-like conoinsulins in piscivorous cone snails supports their use for fish capture. These vertebrate-like conoinsulins are similar to fish insulins, bind to zebrafish insulin receptors, and can induce hypoglycemic shock to facilitate prey capture [15]. This is further supported by the complete absence of vertebrate-like conoinsulins in the venom of molluscivorous or vermivorous cone snails. By contrast, mollusk hunters produce MIP-like conoinsulins characterized by an additional disulfide bond in the B-chain, suggesting an evolutionary adaptation for their specific molluskan target prey [15, 85][15, 85] (Figure 6).

Field Code Changed

Field Code Changed

Field Code Changed

Cone snails employ their venom for defense as well; indeed, they can adjust their venom composition based on intended use, whether for predation or defense [75][75]. Cluster analysis alone cannot clearly distinguish between worm and molluskan insulins [79][79] (Figure 6). Interestingly, pPiscivorous cone snails also produce MIP-like conoinsulins. Two fish-hunting species, *C. catus* and *C. varius*, both fish-hunting species, were found only to produce MIP-like conoinsulins, while *C. geographus* produces both MIP-like and vertebrate-like conoinsulins, albeit with vertebrate-like conoinsulins being predominant in the latter case (Figure 6, Figure S4). It is not well understood whether MIP-like conoinsulins are used for prey purposes or to defend against molluscan predators such as octopuses. However, based on the cluster analysis, there are no apparent similarities with octopus insulin, indicating that they may be used for prey capture or to defend against other mollusk-hunting cone snails (Figure 6).

Field Code Changed

Field Code Changed

The application of PeptideMiner substantially expanded the known diversity of conoinsulins, unveiling numerous new conoinsulin variants, thereby underscoring their role and significance in envenomation. Through the analysis of 18 cone snail species, we identified 78 novel insulin precursors and 59 novel mature conoinsulins (Figures S4-6), in addition to the previously identified MIP-like Con-Ins Pl1, M2, Tx1, Tx2, Va1, Pl174, and vertebrate-like Con-Ins G1b, G3, G121 Tu478, Tu479, Tu304, Tu073. This includes the documentation of conoinsulins in *C. catus, C. miles, C. distans, C. ebreaus, C. emaciatus, C. flavidus, C. miliaris, C. sanguinolentus* and *C. vexillum* for the first time. PeptideMiner also revealed several novel

conoinsulin precursors and mature peptides in previously annotated transcriptomes of *C. catus*, *C. geographus*, *C. miles*, *C. planorbis*, *C. tulipa*, and *C. vexillum* that were not identified using BLASTp searches [75, 113-120][75, 111, 112, 114-119], exemplifying the enhanced performance of PeptideMiner.

TOf note, the absence of conoinsulins in *C. rattus and C. imperialis* is consistent with previous findings indicating that conoinsulins are not universally recruited into venom glands [79][79]. InterestinglyOf note, MIP-like conoinsulin G2, identified before previously [15], was not observed in our *C. geographus* transcriptomes (including a direct manual search). These results collectively highlight the diverse sequence variability and wide distribution of conoinsulins in the genus *Conus*.

Field Code Changed

Field Code Changed

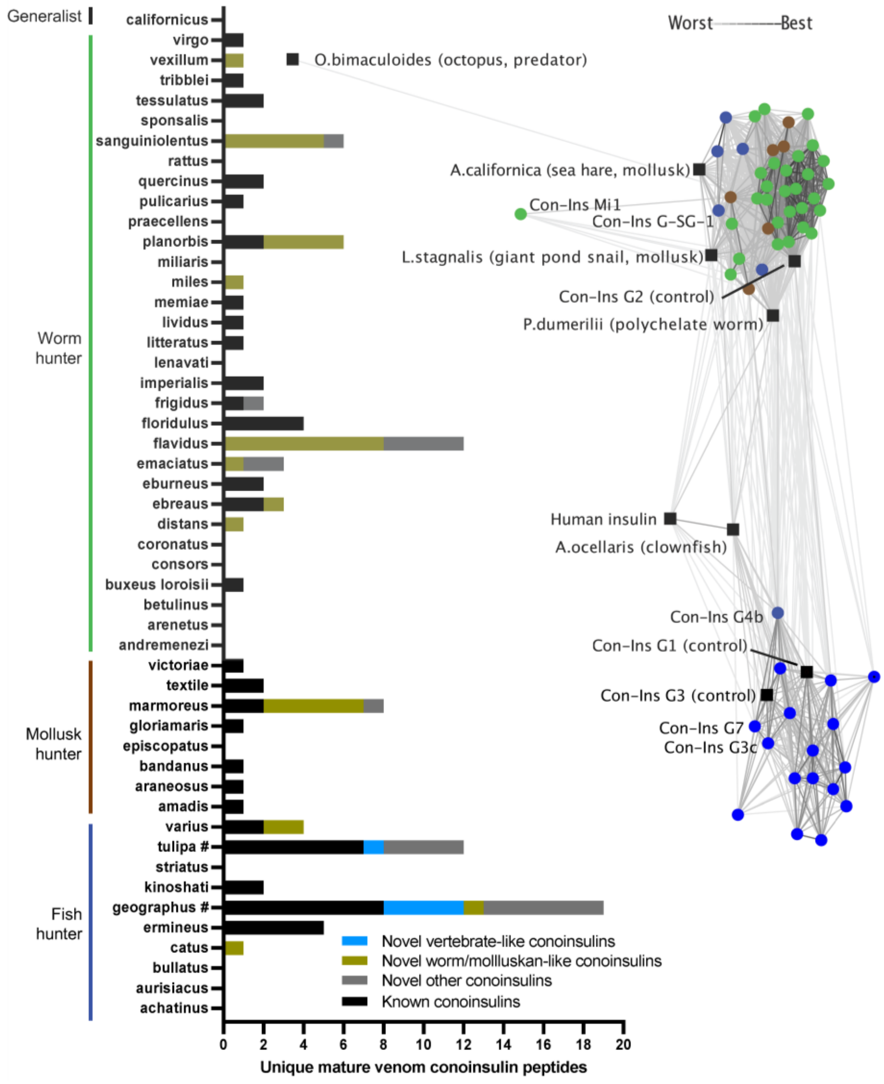

Figure 6. Number and similarity of known conoinsulin sequences and newly identified conoinsulin sequences by PeptideMiner. Known conoinsulin sequences (from UniProt, GenBank, or literature [114, 118, 121][111-113]) are presented in black, and newly identified conoinsulin sequences are further categorized as vertebrate-like (light blue), molluskan-like (olive), or other (gray). Species were grouped according to their

Formatted: Space After: 10 pt, Widow/Orphan control

Field Code Changed

diet: fish hunters (dark blue), mollusk hunters (brown), worm hunters (green), and generalists. # indicates net-hunters. All-against-all BLAST e-value clustering was performed using CLANS, and shows similarity between sequences (line length, and weight) of newly discovered conoinsulins (circles) and ~~control~~ previously characterized sequences (squares).

Using PeptideMiner, we also identified five novel vertebrate-like mature conoinsulin sequences from two fish-hunting species, *C. geographus* and *C. tulipa*: Con-Ins G3c, G4b, G5, G7 and T3b (Figure 6), adding to the seven previously identified C. *geographus* and five previously identified C. *tulipa* conoinsulins [122][120]. We synthesized Con-Ins G3c, G4b, and G7 and assessed their binding affinity at the human insulin receptor. Con-Ins G4b and G7 demonstrated low nanomolar binding at hIR-B, underscoring the applicability of PeptideMiner and our approach for identifying new insulin-like analogs with activity at the human receptors. Con-Ins G3c was inactive at hIR-B; however, its SAR was still useful, particularly in highlighting the importance of Gly$^{8}_{B}$ for human insulin receptor binding. InterestinglyNotably, the differences between Con-Ins G4b and G7 to human insulin do not involve any of the oligomerization surface residues (Ala$^{12}_{A}$, Leu$^{13}_{A}$, Glu$^{17}_{A}$, His$^{10}_{B}$, Glu$^{13}_{B}$, Leu$^{17}_{B}$), which could be exploited for the development of fast-acting insulin analogs [123, 124][121, 122].

Field Code Changed

Field Code Changed

Taken together, these results underscore PeptideMiner as a powerful new discovery tool that enhances our capabilities in identifying and mapping distant neuropeptides s and putative homologs across the animal kingdom based on sequence similarity, and facilitates the discoverydiscovering ofnew peptides homologs with affinity atfor human receptors. These enhanced capabilities are expected to pave the way for developing new pharmacological probes or therapeutic leads, thereby offering promising prospects for advancing biomedical research and drug development.

Conclusions

PeptideMiner is a new and highly versatile computational discovery platform designed to efficiently identify (neuro)peptide families across diverse databases and species. Demonstrating superior performance compared to BLASTp, PeptideMiner offers several advantages in accurate neuropeptide discovery, adeptly handling short and divergent sequences, and efficiently mapping neuropeptides across evolutionarily distant species. This study highlights the untapped potential of venoms as a rich new source for neuropeptide ~~homologs~~ analogs and provides a robust framework for their systematic and efficient exploration to discover valuable new pharmacological probes and therapeutic leads. PeptideMiner unveiled 36 unique conoinsulins (Figure S4) and 8 natriuretic peptides (Figure 3), substantially expanding our understanding of the molecular diversity within venoms across different animal species. Selected conoinsulins bound to the human insulin receptor, emphasizing the translational promise of this approach.

Taken together, PeptideMiner is an invaluable open-access tool poised to propel neuropeptide research forward, deepen our comprehension of neuropeptide signaling in health and disease, and expedite the discovery and development of novel pharmacological probes and therapeutic interventions.

Materials and Methods

Materials

Fmoc amino acids were from Iris Biotech GmbH (Marktredwitz, Germany), and Fmoc- Asp(t-Bu)-Wang resin (loading 0.81 mmol/g) and Fmoc-L-His(Trt)-AC TentaGel resin (loading 0.22 mmol/g) was from Rapp Polymere GmbH (Tübingen, Germany). Acetonitrile (ACN) was obtained from Merck (Bayswater, Australia). Dimethylformamide (DMF), methanol (MeOH), trifluoroacetic acid (TFA), and diethyl ether were obtained from Chem-Supply (Gillman, Australia). All solvents were obtained with the highest available purity and used without further purification. All other reagents, including N,N-diisopropylethylamine (DIEA), (O-(6-chlorobenzotriazol-1-yl)-N,N,N',N'-tetramethyluronium hexafluorophosphate (HCTU), Anisole, 2,2′-(ethylenedioxy)diethanethiol (DODt), triisopropylsilane (TIPS), 2,2'-dipyridyldisulfide (DPDS) and trifluoromethanesulfonic acid (TFMSA) and solvents were obtained from Sigma Aldrich (Merck, Sydney, NSW, Australia) in the highest available purity. Solvents for reversed-phase~~d~~ HPLC (RP-HPLC) consisted of 0.05% TFA/H_2O (Solvent A) and 0.043% TFA/ACN (Solvent B). Analytical HPLC was performed with column heating at 40°C and detection at 214 nm. Preparative HPLC was performed on a Vydac Protein and Peptide C_{18} preparative column, and crude and fractions were analyzed using RP-HPLC and electrospray ionization mass spectrometry (ESI-MS). Mass analysis of the final product was performed on a Q-Star Pulsar mass spectrometer (SCIEX, Ontario, Canada) with a Series 1100 solvent delivery system equipped with an auto-injector (Agilent Technologies Inc., Palo Alto, CA, USA) and a Kromasil Classic liquid chromatography MS (LC-MS) C_{18} column (100 Å, 3.5 µm, 150 mm x 2.1 mm). Data acquisition and processing were carried out using Analyst software v1.1 (SCIEX, Canada). Trizol was from ThermoFisher Scientific. Oligotex mRNA Mini Kit was from Qiagen (Valencia, CA, USA).

Transcriptome Preparation

The transcriptomes were sourced from unpublished in-house collections (~~all~~ 23 *Conus* ~~, anemone, arachnid, centipede transcriptomes,~~ and *S. horrida* transcriptomes) or downloaded from the NCBI Sequence Read Archive (SRA) database. A full list of transcriptomes searched is provided in Table S3. For the in-house collections, total RNA was extracted from the stripped venom gland cells using Trizol (Invitrogen) reagent according to the manufacturer's instructions. mRNA was purified from the total RNA using Oligotex mRNA

Mini Kit (Qiagen, Valencia, CA, USA) according to the manufacturer's instructions. cDNA library construction and sequencing were carried out using a Roche GS FLX Titanium sequencer at the Australian Genomic Research Facility (AGRF). Data assembly was carried out using Newbler 2.3 Life Science, Frederick, CO). In total, 57~~65~~ animal venom organ transcriptomes from 48~~62~~ species, including 39~~53~~ invertebrates and 9 vertebrates across five phyla (Cnidaria, Arthropoda, Mollusca, Annelida, and Chordata) were searched for the natriuretic peptide neuropeptide family, and 23 cone snail transcriptomes comprising 20 venom ducts, two circumoesophageal nerve rings, and one salivary gland transcriptome for the conoinsulin family.

PeptideMiner Workflow

PeptideMiner is constructed as a series of steps with user-friendly output files created at each step. PeptideMiner takes as input one or more profile-HMMs of the neuropeptide family of interest and searches a database of amino acid sequences that the user can provide as multiple fasta files (for instance, one fasta file for each transcriptome) or combine into a single large fasta file database.

Hmmsearch from the HMMER3 package searches the profile-HMM against the amino acid database (in this work, translated transcriptomes). PeptideMiner runs hmmsearch and creates an output for each dataset searched (Step 0). The hmmsearch output does not include the hit sequence; therefore, the hit read names are matched with their respective read name in the amino acid database, and the sequence is extracted. The sequence is combined with the hmmsearch output to generate a report of identified neuropeptide candidates. The bioinformatics pipeline subsequently filters, processes, and annotates this initial neuropeptide identification report.

The hmmsearch search results are stored in an SQLite database (Figure S2). To facilitate downstream processing, all hmmsearch search hits are combined into a single file (Step 2). If more than one profile-HMM was used for a single neuropeptide family, the combined hmmsearch output could contain duplicate reads. For this case, the hit with the lowest E-value is selected. The E-value is an estimate of the Expected number of errors. In other words, it represents the probability of observing such results by chance. The E-value of a read returned by hmmsearch represents the statistical significance of the hit. The lower the E-value, typically <<1, the more statistically significant the hit and the less likely it is a random hit [28].

In Step 3, the protein-coding sequences (CDS) of the hypothetical neuropeptide precursors are predicted using a Python script that extracts the sequence between the methionine, 'M', (encoding the start codon) and the stop codon or, if there is no stop codon, the end of the contig (Figure 1, Step 3). Multiple CDS can be identified

from a single contig if multiple methionine residues are present. This step is relevant for protein sequences obtained by direct translation of nucleotide transcripts. The user can specify a minimum cut-off length for the CDS to minimize false positives.

The signal peptide of the predicted CDS is then identified using SignalP [34] (Figure 1, Step 4). If present, the signal peptide is subsequently excised from the sequence of the precursor to facilitate subsequent mature peptide identification.

Step 5 aims to identify the mature peptide(s) in the CDS; FASTA36 [35] aligns all the CDS to a list of known mature peptides of the neuropeptide family of interest, followed by cleavage site prediction using an algorithm from the ConoServer annotation pipeline [44][43] (Step 5).

Field Code Changed

The predicted mature peptides for all sequences from the same transcriptome are then compiled, and duplicate mature peptides are removed (Step 6). Protein-protein BLAST (BLASTp) is then used to annotate the predicted mature peptides by homology using a list of known neuropeptide amino acid sequences (Step 7).

PeptideMiner Search Parameters

The user can modify several parameters of PeptideMiner to optimize neuropeptide identification. Firstly, the user can specify the minimum length of the CDS in the number of amino acids, enabling the filtering of sequence candidates that are too short (Step 3), for example, to exclude incomplete CDS or fragments.

Secondly, the user can modify the SignalP parameters to identify the signal peptide in CDS (Step 4). This step is important because the presence of a signal peptide is supportive of a neuropeptide. The user can change the SignalP D-value (the score used to discriminate signal peptides from non-signal peptides; peptides with no signal peptide have a very low D-value) and minimum signal peptide length, which are set to a default of 0.45 and 9, respectively, which is the default setting for non-transmembrane eukaryotic precursors [34]. Reducing the D-value would increase speed at the cost of sensitivity.

Finally, the user can alter three parameters for mature peptide identification (Step 5). FASTA36 [35] is used to align known mature peptides to the CDS to identify mature peptides in the CDS. The user can specify the E-value cut-off of the FASTA36 search as well as the minimum and maximum length allowed for the mature peptides. The E-value is typically dependent on the overall length of the alignment, the percentage identity, and the size of the database. Generally, a lower E-value indicates a better quality in the alignment between the mature peptide and the known peptide. The optimal E-value cut-off varies depending on the neuropeptide family because the various neuropeptides have different lengths and degrees of sequence conservation. In practice, an E-value range between $1x10^{-4}$–1 is typically used, although the cut-off will vary depending on the

query sequence length and database size.

Pipeline Output

The pipeline creates several outputs for the user, including a final output file that lists all the hits and their similarity (% identity and BLASTp E-value) to known sequences (Step 8). In addition, PeptideMiner generates a report that lists the number of profile-HMMs used, the number of files searched, the number of hits the hmmsearch returned, and the final number of hits, including the number of CDS and mature peptides identified. A separate file reports the number of reads found with each profile-HMM used to search the database.

RNA isolation and transcriptome generation

All cone snails used to generate the transcriptomes were collected from the Great Barrier Reef, Queensland, Australia (GBRMP permit G10/33243.1). Cone snails were sacrificed and dissected immediately on ice. The whole venom gland was separated from the other tissues (not including the venom bulb at the proximal end and the proboscis at the distal end), and the venom gland cells were stripped out from the venom gland. Total RNA was extracted from the stripped venom gland cells using TRI~~ri~~zol reagent according to the manufacturer's instructions. mRNA was purified from the total RNA using an Oligotex mRNA Mini Kit according to the manufacturer's instructions. cDNA library construction and sequencing were carried out using a Roche GS FLX Titanium sequencer at the Australian Genomic Research Facility (AGRF). Data assembly was carried out using Newbler 2.3 Life Science, Frederick, CO). Only one individual was used to generate the transcriptomes of larger species (*C. geographus, C. tulipa, C. planorbis, C. distans, C. sangunilantus, C. vexillum, C. marmoreus, C. miles, C. imperialis)* and multiple specimens were used for smaller species (e.g., six specimens for *C. catus*).

Solid-phase peptide synthesis

Synthesis of Con-Ins G3c, G4b, and G7

The linear conoinsulin A-chains were manually synthesized using Fmoc-SPPS on an Fmoc-L-His(Trt)-AC TentaGel resin on a 0.22 mmol scale. A regioselective folding strategy using acetamidomethyl (Acm), *tert*-butyl (tBu), and trityl (Trt) cysteine protecting groups was employed to achieve the A-chain intrachain and two A-B-chain interchain disulfide bonds. N-terminal Fmoc deprotection was achieved with 20% v/v piperidine in DMF for 2×5 min. Each Fmoc-protected amino acid (3 eq.) was coupled using DMF as the solvent and DIEA (1 M in DMF) with HCTU (3 eq.) as the activating agents for 50 min. The cycle of deprotection, washing, and coupling was repeated until the full-length peptide was obtained. The completed linear A-chain was cleaved

by a cocktail of TFA/anisole/DODt/TIPS (94/3/2/1%) for 3 h. The cleavage mixture was then filtered, concentrated by a stream of N_2, precipitated in cold diethyl ether, and centrifuged for 5 min. The crude A-chain (544 mg) was collected as a white powder and used directly for further synthesis.

The A-chain intrachain disulfide bond was formed by dissolving the crude linear A-chain (0.102 mmol) in an H_2O/ACN mixture (4:1, 500 mL) with 5 drops of DIEA. After adding DPDS (1.0 eq.) in 1.0 mL MeOH, the mixture was stirred for 2.5 h at 40°C. The mixture was purified using a preparative RP-HPLC (15-55% solvent B in 30 min) and lyophilized.

The cyclized A-chain (31.5 μmol) and DPDS (4 eq.) were dissolved in an anisole/TFA mixture (1:9, 2 mL), and then a TFMSA/TFA mixture (1:4, 2 mL) was added. The resulting mixture was stirred at 0°C for 45 min, then precipitated in cold diethyl ether and centrifuged (5 repeats). The crude peptide was purified by a semi-preparative RP-HPLC (15-55% solvent B in 30 min) and lyophilized.

All three B-chains were purchased from GL Biochem (Shanghai, China). To a mixture of conoinsulin B-chain in acidic guanidium HCl buffer (6 M, 1 mL, pH = 5.0), conoinsulin A-chain in alkaline guanidium HCl buffer (6 M, 2 mL, pH = 8.5) was added dropwise. The resulting mixture was stirred for 15 min, purified using a semi-preparative RP-HPLC (15-45% solvent B in 30 min), and lyophilized.

To the lyophilized powder was added an aqueous solution of HCl (60 mM, 0.4 mL), acetic acid (3.15 mL), and an iodine solution (20 mM in acetic acid, 4.2 mL). The mixture was stirred for 1 h, then precipitated in cold ether and centrifuged. The crude peptide was purified by a semi-preparative RP-HPLC (15-45% solvent B in 30 min) and lyophilized.

Con-Ins G3c: A-chain (10 mg) and B-chain (10.5 mg) were used, yielding 0.5 mg of white powder. Con-Ins G7: A-chain (10 mg) and B-chain (12 mg) yielded 1.3 mg of white powder. Con-Ins G4b: A-chain (5.9 mg) and B-chain (7.5 mg) yielded 0.7 mg of white powder.

Human insulin receptor-B binding experiments

Receptor binding was measured as described previously [94]~~[94]~~. Briefly, insulin-like growth factor 1 receptor (IGF-1R)-negative cells overexpressing the human insulin receptor-B (hIR-B) were generated. Cells were serum-starved for 4 h before lysis. Lysates were captured in a 96-well plate previously coated with anti-IR antibody. Approximately 500,000 fluorescent counts of europium-labeled ~~insulin~~ human insulin (Eu-insulin) were added to each well along with increasing concentrations of unlabeled competitor and incubated for 16 h at 4°C. After washing, time-resolved fluorescence was measured using 340 nm excitation and 612 nm emission filters with the BMG Lab ~~technologies~~ Technologies Polarstar fluorometer (Mornington, Australia). Insulin

Field Code Changed

and synthetic conoinsulin analog curves are from three separate experiments, each point performed in triplicate. Binding affinity was expressed in IC_{50} and represents the ligand concentration (human insulin or conoinsulin) necessary to displace 50% of Eu-insulin from hIR-B.

Conoinsulin structure prediction

Three-dimensional structures for Con-Ins G3C, G4b and G7 were predicted using AlphaFold2 [97]~~[97]~~, implemented in ColabFold [125]~~[123]~~ running remotely on a Python 3 Google Compute Engine backend. The alphafold2_multimer_v3 model and no template information were used for the prediction. The highest-ranked, Amber-relaxed model was aligned with the crystal structure of human insulin (PDB:3w7y).

Clustering Analysis

To compare the identified insulin-like peptides and natriuretic peptides, we used CLANS [126]~~[124]~~ to cluster mature amino acid sequences based on all-against-all pairwise BLASTp E-values. Pairwise BLASTp searches were performed with the CLANS web-utility (https://toolkit.tuebingen.mpg.de/tools/clans) [127]~~[125]~~ using default parameters, while clustering and visualization of the resulting similarity matrix w~~as~~ere ~~done~~ completed with the Java-based CLANS tool, using P-values better than 1e-4 and otherwise default parameters.

Data availability

PeptideMiner is registered with bio.tools (biotools:peptideminer) and SciCrunch.org (RRID: SCR-026456), is ~~is~~ publicly available for download from GitHub (https://www.github.com/muttenthalerlab/PeptideMiner), and is released under the GNU General Public License v3.0.

Acknowledgments

We thank Dr. Akhter Hossain for synthesizing Con-Ins G3c, G4b, and G7, Dr. Himaya Hewage for transcriptome details, A/Prof Lachlan Rash for access to the venom transcriptome of the King Baboon Spider, and Dr. Prashanth Jutty Rajan for initial training in transcriptome analysis. M.M. was supported by the European Research Council under the European Union's Horizon 2020 research and innovation program (714366) and by the Australian Research Council (DE150100784, DP190101667, FT210100266), and by the National Health and Medical Research Council (2037680). EABU was supported by the European Research Council (ERC-2021-STG under grant agreement No 101039862) and the Norwegian Research Council (FRIPRO-YRT Fellowship no. 287462).

References

1. King GF. Venoms as a platform for human drugs: translating toxins into therapeutics. Expert Opin Biol Th. 2011;11 11:1469-84.
2. Lewis RJ and Garcia ML. Therapeutic potential of venom peptides. Nat Rev Drug Discov. 2003;2 10:790-802.
3. Muttenthaler M, King GF, Adams DJ and Alewood PF. Trends in peptide drug discovery. Nature Reviews Drug Discovery. 2021;20 4:309-25.
4. Näreoja K and Näsman J. Selective targeting of G-protein-coupled receptor subtypes with venom peptides. Acta Physiologica. 2012;204 2:186-201.
5. Sharpe IA, Gehrmann J, Loughnan ML, Thomas L, Adams DA, Atkins A, et al. Two new classes of conopeptides inhibit the α1-adrenoceptor and noradrenaline transporter. Nature Neuroscience. 2001;4 9:902-7.
6. Warkentin TE and Koster A. Bivalirudin: a review. Expert Opinion on Pharmacotherapy. 2005;6 8:1349-71.
7. Zhang L, Lu SH, Li L, Tao Y-G, Wan YL, Senga H, et al. Batroxobin Mobilizes Circulating Endothelial Progenitor Cells in Patients With Deep Vein Thrombosis. Clinical and Applied Thrombosis/Hemostasis. 2011;17 1:75-9.
8. Eagles DA, Saez NJ, Krishnarjuna B, Bradford JJ, Chin YK-Y, Starobova H, et al. A peptide toxin in ant venom mimics vertebrate EGF-like hormones to cause long-lasting hypersensitivity in mammals. Proceedings of the National Academy of Sciences. 2022;119 7:e2112630119.
9. Jami S, Erickson A, Brierley S and Vetter I. Pain-Causing Venom Peptides: Insights into Sensory Neuron Pharmacology. Toxins. 2017;10 1:15.
10. Netirojjanakul C and Miranda LP. Progress and challenges in the optimization of toxin peptides for development as pain therapeutics. Curr Opin Chem Biol. 2017;38:70-9.
11. Miljanich GP. Ziconotide: neuronal calcium channel blocker for treating severe chronic pain. Curr Med Chem. 2004;11 23:3029-40.
12. Taylor K, Kim D, Nielsen LL, Aisporna M, Baron AD and Fineman MS. Day-long Subcutaneous Infusion of Exenatide Lowers Glycemia in Patients with Type 2 Diabetes. Hormone and Metabolic Research. 2005;37 10:627-32.
13. Cruz LJ, de Santos V, Zafaralla GC, Ramilo CA, Zeikus R, Gray WR and Olivera BM. Invertebrate vasopressin/oxytocin homologs. Characterization of peptides from Conus geographus and Conus straitus venoms. J Biol Chem. 1987;262 33:15821-4.
14. Craig AG, Norberg T, Griffin D, Hoeger C, Akhtar M, Schmidt K, et al. Contulakin-G, an O-Glycosylated Invertebrate Neurotensin. Journal of Biological Chemistry. 1999;274 20:13752-9.
15. Safavi-Hemami H, Gajewiak J, Karanth S, Robinson SD, Ueberheide B, Douglass AD, et al. Specialized insulin is used for chemical warfare by fish-hunting cone snails. Proceedings of the National Academy of Sciences. 2015;112 6:1743-8.
16. Robinson SD, Safavi-Hemami H, Raghuraman S, Imperial JS, Papenfuss AT, Teichert RW, et al. Discovery by proteogenomics and characterization of an RF-amide neuropeptide from cone snail venom. J Proteomics. 2015;114:38-47.
17. Schweitz H, Vigne P, Moinier D, Frelin C and Lazdunski M. A New Member of the Natriuretic Peptide Family Is Present in the Venom of the Green Mamba (Dendroaspis-Angusticeps). Journal of Biological Chemistry. 1992;267 20:13928-32.
18. Hokfelt T, Broberger C, Xu ZQ, Sergeyev V, Ubink R and Diez M. Neuropeptides--an overview. Neuropharmacology. 2000;39 8:1337-56.
19. Mendel HC, Kaas Q and Muttenthaler M. Neuropeptide signalling systems – An underexplored target for venom drug discovery. Biochemical Pharmacology. 2020;181:114129.
20. Elphick MR, Mirabeau O and Larhammar D. Evolution of neuropeptide signalling systems. J Exp Biol. 2018;221 Pt 3.
21. Buermans HP and den Dunnen JT. Next generation sequencing technology: Advances and applications. Biochim Biophys Acta. 2014;1842 10:1932-41.
22. Calvete JJ. Venomics: integrative venom proteomics and beyond. Biochem J. 2017;474:611-34.

Formatted: EndNote Bibliography, Left, Indent: Left: 0", Hanging: 0.5", Widow/Orphan control, Tab stops: Not at 0.39"

Formatted: Font: (Default) Calibri, Do not check spelling or grammar

Formatted: Font: (Default) Calibri, German (Germany), Do not check spelling or grammar

Formatted: Font: (Default) Calibri, Do not check spelling or grammar

23. Chen YP and Chen F. Identifying targets for drug discovery using bioinformatics. Expert Opin Ther Targets. 2008;12 4:383-9.
24. Escoubas P and King GF. Venomics as a drug discovery platform. Expert Rev Proteomic. 2009;6 3:221-4.
25. Oldrati V, Arrell M, Violette A, Perret F, Sprungli X, Wolfender JL and Stocklin R. Advances in venomics. Mol Biosyst. 2016;12 12:3530-43.
26. Clynen E, Liu F, Husson SJ, Landuyt B, Hayakawa E, Baggerman G, et al. Bioinformatic approaches to the identification of novel neuropeptide precursors. Methods in molecular biology. 2010;615:357-74.
27. Caers J, Verlinden H, Zels S, Vandersmissen HP, Vuerinckx K and Schoofs L. More than two decades of research on insect neuropeptide GPCRs: an overview. Front Endocrinol (Lausanne). 2012;3:151.
28. Eddy SR. Accelerated Profile HMM Searches. PLoS Comput Biol. 2011;7 10:e1002195.
29. Durbin R, Eddy SR, Krogh A and Mitchison GJ. Biological Sequence Analysis: Probabilistic Models of Proteins and Nucleic Acids. In: 1998.
30. Park J, Karplus K, Barrett C, Hughey R, Haussler D, Hubbard T and Chothia C. Sequence comparisons using multiple sequences detect three times as many remote homologues as pairwise methods. J Mol Biol. 1998;284 4:1201-10.
31. Yoon BJ. Hidden Markov Models and their Applications in Biological Sequence Analysis. Curr Genomics. 2009;10 6:402-15.
32. Eddy SR. Profile hidden Markov models. Bioinformatics. 1998;14 9:755-63.
33. Eddy SR: HMMER 3.1b. hmmer.org (2015).
34. Petersen TN, Brunak S, von Heijne G and Nielsen H. SignalP 4.0: discriminating signal peptides from transmembrane regions. Nat Methods. 2011;8 10:785-6.
35. Pearson WR. Searching protein sequence libraries: comparison of the sensitivity and selectivity of the Smith-Waterman and FASTA algorithms. Genomics. 1991;11 3:635-50.
36. Altschul SF, Madden TL, Schaffer AA, Zhang J, Zhang Z, Miller W and Lipman DJ. Gapped BLAST and PSI-BLAST: a new generation of protein database search programs. Nucleic Acids Res. 1997;25 17:3389-402.
37. Hipp DR, Kennedy D and Mistachkin J. SQLite (Version 3.27.2). SQLite Development Team, 2015.
38. Southey BR, Sweedler JV and Rodriguez-Zas SL. A python analytical pipeline to identify prohormone precursors and predict prohormone cleavage sites. Front Neuroinform. 2008;2:7.
39. Bassi S. A primer on python for life science researchers. PLoS Comput Biol. 2007;3 11:e199.
40. Edgar RC. MUSCLE: multiple sequence alignment with high accuracy and high throughput. Nucleic Acids Res. 2004;32 5:1792-7.
41. Sievers F, Wilm A, Dineen D, Gibson TJ, Karplus K, Li W, et al. Fast, scalable generation of high-quality protein multiple sequence alignments using Clustal Omega. Mol Syst Biol. 2011;7:539.
42. Waterhouse AM, Procter JB, Martin DM, Clamp M and Barton GJ. Jalview Version 2--a multiple sequence alignment editor and analysis workbench. Bioinformatics. 2009;25 9:1189-91.
43. Eddy SR. A new generation of homology search tools based on probabilistic inference. Genome Inform. 2009;23 1:205-11.
44. Kaas Q, Yu R, Jin AH, Dutertre S and Craik DJ. ConoServer: updated content, knowledge, and discovery tools in the conopeptide database. Nucleic Acids Res. 2012;40 Database issue:D325-30.
45. Suzek BE, Wang Y, Huang H, McGarvey PB, Wu CH and UniProt C. UniRef clusters: a comprehensive and scalable alternative for improving sequence similarity searches. Bioinformatics. 2015;31 6:926-32.
46. Potter LR, Yoder AR, Flora DR, Antos LK and Dickey DM. Natriuretic peptides: their structures, receptors, physiologic functions and therapeutic applications. Handb Exp Pharmacol. 2009; 191:341-66.
47. Pandit K, Mukhopadhyay P, Ghosh S and Chowdhury S. Natriuretic peptides: Diagnostic and therapeutic use. Indian J Endocrinol Metab. 2011;15 Suppl 4:S345-53.
48. Poulos JE, Gower WR, Jr., Friedl FE and Vesely DL. Atrial natriuretic peptide gene expression within invertebrate hearts. Gen Comp Endocrinol. 1995;100 1:61-8.
49. Vesely DL and Giordano AT. The most primitive heart in the animal kingdom contains the atrial natriuretic peptide hormonal system. Comp Biochem Physiol C. 1992;101 2:325-9.

Formatted: Font: (Default) Calibri, German (Germany), Do not check spelling or grammar

Formatted: Font: (Default) Calibri, Do not check spelling or grammar

50. Peterfi O, Boda F, Szabo Z, Ferencz E and Baba L. Hypotensive Snake Venom Components-A Mini-Review. Molecules. 2019;24 15.
51. Higuchi S, Murayama N, Saguchi K, Ohi H, Fujita Y, Camargo AC, et al. Bradykinin-potentiating peptides and C-type natriuretic peptides from snake venom. Immunopharmacology. 1999;44 1-2:129-35.
52. Ichiki T, Dzhoyashvili N and Burnett JC, Jr. Natriuretic peptide based therapeutics for heart failure: Cenderitide: A novel first-in-class designer natriuretic peptide. Int J Cardiol. 2018.
53. Alves RS, Ximenes RM, Jorge AR, Nascimento NR, Martins RD, Rabello MM, et al. Isolation, homology modeling and renal effects of a C-type natriuretic peptide from the venom of the Brazilian yellow scorpion (Tityus serrulatus). Toxicon. 2013;74:19-26.
54. de Plater GM, Martin RL and Milburn PJ. A C-type natriuretic peptide from the venom of the platypus (Ornithorhynchus anatinus): structure and pharmacology. Comp Biochem Physiol C Pharmacol Toxicol Endocrinol. 1998;120 1:99-110.
55. Fry BG, Roelants K, Winter K, Hodgson WC, Griesman L, Kwok HF, et al. Novel venom proteins produced by differential domain-expression strategies in beaded lizards and gila monsters (genus Heloderma). Mol Biol Evol. 2010;27 2:395-407.
56. Ziegman R, Undheim EAB, Baillie G, Jones A and Alewood PF. Investigation of the estuarine stonefish (Synanceia horrida) venom composition. J Proteomics. 2019;201:12-26.
57. Vink S, Jin AH, Poth KJ, Head GA and Alewood PF. Natriuretic peptide drug leads from snake venom. Toxicon. 2012;59 4:434-45.
58. Schweitz H, Vigne P, Moinier D, Frelin C and Lazdunski M. A new member of the natriuretic peptide family is present in the venom of the green mamba (Dendroaspis angusticeps). J Biol Chem. 1992;267 20:13928-32.
59. Zhang Y, Wu J, Yu G, Chen Z, Zhou X, Zhu S, et al. A novel natriuretic peptide from the cobra venom. Toxicon. 2011;57 1:134-40.
60. Xie B, Dashevsky D, Rokyta D, Ghezellou P, Fathinia B, Shi Q, et al. Dynamic genetic differentiation drives the widespread structural and functional convergent evolution of snake venom proteinaceous toxins. BMC Biology. 2022;20 1.
61. Soares MR, Oliveira-Carvalho AL, Wermelinger LS, Zingali RB, Ho PL, Junqueira-de-Azevedo IL and Diniz MR. Identification of novel bradykinin-potentiating peptides and C-type natriuretic peptide from Lachesis muta venom. Toxicon. 2005;46 1:31-8.
62. Schmidt JJ, Weinstein SA and Smith LA. Molecular properties and structure-function relationships of lethal peptides from venom of Wagler's pit viper, Trimeresurus wagleri. Toxicon. 1992;30 9:1027-36.
63. Tsai MC, Hsieh WH, Smith LA and Lee CY. Effects of waglerin-I on neuromuscular transmission of mouse nerve-muscle preparations. Toxicon. 1995;33 3:363-71.
64. Tan CH, Tan KY and Tan NH. De Novo Assembly of Venom Gland Transcriptome of Tropidolaemus wagleri (Temple Pit Viper, Malaysia) and Insights into the Origin of Its Major Toxin, Waglerin. Toxins (Basel). 2023;15 9.
65. Yang Y, Xiong J, Zhou Z, Huo F, Miao W, Ran C, et al. The genome of the myxosporean Thelohanellus kitauei shows adaptations to nutrient acquisition within its fish host. Genome Biol Evol. 2014;6 12:3182-98.
66. Shabanpoor F, Separovic F and Wade JD. The human insulin superfamily of polypeptide hormones. Vitam Horm. 2009;80:1-31.
67. Weiss M, Steiner DF and Philipson LH. Insulin Biosynthesis, Secretion, Structure, and Structure-Activity Relationships. In: De Groot LJ, Chrousos G, Dungan K, Feingold KR, Grossman A, Hershman JM, et al., editors. Endotext. South Dartmouth (MA); 2000.
68. Chan SJ and Steiner DF. Insulin through the ages: Phylogeny of a growth promoting and metabolic regulatory hormone. Am Zool. 2000;40 2:213-22.
69. De Meyts P. Insulin and its receptor: structure, function and evolution. Bioessays. 2004;26 12:1351-62.
70. Tokarz VL, Macdonald PE and Klip A. The cell biology of systemic insulin function. Journal of Cell Biology. 2018;217 7:2273-89.

71. Adams MJ, Blundell TL, Dodson EJ, Dodson GG, Vijayan M, Baker EN, et al. Structure of Rhombohedral 2 Zinc Insulin Crystals. Nature. 1969;224 5218:491-&.
72. Lisi GP, Png CYM and Wilcox DE. Thermodynamic Contributions to the Stability of the Insulin Hexamer. Biochemistry. 2014;53 22:3576-84.
73. Blumenthal S. From insulin and insulin-like activity to the insulin superfamily of growth-promoting peptides: a 20th-century odyssey. Perspect Biol Med. 2010;53 4:491-508.
74. Smit AB, van Kesteren RE, Li KW, Van Minnen J, Spijker S, Van Heerikhuizen H and Geraerts WP. Towards understanding the role of insulin in the brain: lessons from insulin-related signaling systems in the invertebrate brain. Prog Neurobiol. 1998;54 1:35-54.
75. Dutertre S, Jin AH, Vetter I, Hamilton B, Sunagar K, Lavergne V, et al. Evolution of separate predation- and defence-evoked venoms in carnivorous cone snails. Nat Commun. 2014;5:3521.
76. Akondi KB, Muttenthaler M, Dutertre S, Kaas Q, Craik DJ, Lewis RJ and Alewood PF. Discovery, Synthesis, and Structure-Activity Relationships of Conotoxins. Chem Rev. 2014;114 11:5815-47.
77. Jin A-H, Muttenthaler M, Dutertre S, Himaya SWA, Kaas Q, Craik DJ, et al. Conotoxins: Chemistry and Biology. Chem Rev. 2019;119 21:11510-49.
78. Olivera BM, Seger J, Horvath MP and Fedosov AE. Prey-Capture Strategies of Fish-Hunting Cone Snails: Behavior, Neurobiology and Evolution. Brain Behav Evol. 2015;86 1:58-74.
79. Safavi-Hemami H, Lu A, Li Q, Fedosov AE, Biggs J, Showers Corneli P, et al. Venom Insulins of Cone Snails Diversify Rapidly and Track Prey Taxa. Mol Biol Evol. 2016;33 11:2924-34.
80. Ahorukomeye P, Disotuar MM, Gajewiak J, Karanth S, Watkins M, Robinson SD, et al. Fish-hunting cone snail venoms are a rich source of minimized ligands of the vertebrate insulin receptor. eLife. 2019;8.
81. Dutertre S, Jin A-H, Vetter I, Hamilton B, Sunagar K, Lavergne V, et al. Evolution of separate predation- and defence-evoked venoms in carnivorous cone snails. Nature Communications. 2014;5 1.
82. Laugesen SH, Chou DHC and Safavi-Hemami H. Unconventional insulins from predators and pathogens. Nat Chem Biol. 2022;18 7:688-97.
83. Menting JG, Gajewiak J, Macraild CA, Chou DH-C, Disotuar MM, Smith NA, et al. A minimized human insulin-receptor-binding motif revealed in a Conus geographus venom insulin. Nature Structural & Molecular Biology. 2016;23 10:916-20.
84. Robinson SD and Safavi-Hemami H. Insulin as a weapon. Toxicon. 2016;123:56-61.
85. Safavi-Hemami H, Lu A, Li Q, Fedosov AE, Biggs J, Showers Corneli P, et al. Venom Insulins of Cone Snails Diversify Rapidly and Track Prey Taxa. Molecular Biology and Evolution. 2016;33 11:2924-34.
86. Southey BR, Amare A, Zimmerman TA, Rodriguez-Zas SL and Sweedler JV. NeuroPred: a tool to predict cleavage sites in neuropeptide precursors and provide the masses of the resulting peptides. Nucleic Acids Res. 2006;34 Web Server issue:W267-72.
87. Safavi-Hemami H, Gajewiak J, Karanth S, Robinson SD, Ueberheide B, Douglass AD, et al. Specialized insulin is used for chemical warfare by fish-hunting cone snails. Proc Natl Acad Sci USA. 2015;112 6:1743-8.
88. Rholam M, Brakch N, Germain D, Thomas DY, Fahy C, Boussetta H, et al. Role of amino acid sequences flanking dibasic cleavage sites in precursor proteolytic processing. The importance of the first residue C-terminal of the cleavage site. Eur J Biochem. 1995;227 3:707-14.
89. Pardos-Blas JR, Tenorio MJ, Galindo JCG and Zardoya R. Comparative Venomics of the Cryptic Cone Snail Species Virroconus ebraeus and Virroconus judaeus. Marine Drugs. 2022;20 2:149.
90. Biggs JS, Olivera BM and Kantor YI. Alpha-conopeptides specifically expressed in the salivary gland of Conus pulicarius. Toxicon. 2008;52 1:101-5.
91. Lavergne V, Harliwong I, Jones A, Miller D, Taft RJ and Alewood PF. Optimized deep-targeted proteotranscriptomic profiling reveals unexplored Conus toxin diversity and novel cysteine frameworks (vol 112, pg E3782, 2015). P Natl Acad Sci USA. 2015;112 45:E6253-E.
92. Gao BM, Peng C, Zhu YB, Sun YH, Zhao T, Huang Y and Shi Q. High Throughput Identification of Novel Conotoxins from the Vermivorous Oak Cone Snail (Conus quercinus) by Transcriptome Sequencing. Int J Mol Sci. 2018;19 12.

93. Escribano O, Beneit N, Rubio-Longás C, López-Pastor AR and Gómez-Hernández A. The Role of Insulin Receptor Isoforms in Diabetes and Its Metabolic and Vascular Complications. Journal of Diabetes Research. 2017;2017:1-12.
94. Denley A, Bonython ER, Booker GW, Cosgrove LJ, Forbes BE, Ward CW and Wallace JC. Structural determinants for high-affinity binding of insulin-like growth factor II to insulin receptor (IR)-A, the exon 11 minus isoform of the IR. Mol Endocrinol. 2004;18 10:2502-12.
95. Sims EK, Carr ALJ, Oram RA, Dimeglio LA and Evans-Molina C. 100 years of insulin: celebrating the past, present and future of diabetes therapy. Nature Medicine. 2021;27 7:1154-64.
96. De Meyts P. Insulin/receptor binding: the last piece of the puzzle? What recent progress on the structure of the insulin/receptor complex tells us (or not) about negative cooperativity and activation. Bioessays. 2015;37 4:389-97.
97. Jumper J, Evans R, Pritzel A, Green T, Figurnov M, Ronneberger O, et al. Highly accurate protein structure prediction with AlphaFold. Nature. 2021;596 7873:583-+.
98. Grimmelikhuijzen CJ and Hauser F. Mini-review: the evolution of neuropeptide signaling. Regul Pept. 2012;177 Suppl:S6-9.
99. Robinson SD, Li Q, Bandyopadhyay PK, Gajewiak J, Yandell M, Papenfuss AT, et al. Hormone-like peptides in the venoms of marine cone snails. Gen Comp Endocrinol. 2017;244:11-8.
100. Ebberink RHM, Smit AB and Vanminnen J. The Insulin Family - Evolution of Structure and Function in Vertebrates and Invertebrates. Biol Bull. 1989;177 2:176-82.
101. UniProt C. UniProt: a worldwide hub of protein knowledge. Nucleic Acids Res. 2019;47 D1:D506-D15.
102. Floyd PD, Li L, Rubakhin SS, Sweedler JV, Horn CC, Kupfermann I, et al. Insulin prohormone processing, distribution, and relation to metabolism in Aplysia californica. J Neurosci. 1999;19 18:7732-41.
103. Krogh A, Brown M, Mian IS, Sjolander K and Haussler D. Hidden Markov models in computational biology. Applications to protein modeling. J Mol Biol. 1994;235 5:1501-31.
104. Laht S, Koua D, Kaplinski L, Lisacek F, Stocklin R and Remm M. Identification and classification of conopeptides using profile Hidden Markov Models. Biochim Biophys Acta. 2012;1824 3:488-92.
105. Mirabeau O, Perlas E, Severini C, Audero E, Gascuel O, Possenti R, et al. Identification of novel peptide hormones in the human proteome by hidden Markov model screening. Genome Res. 2007;17 3:320-7.
106. Gacesa R, Barlow D and Long PF. Machine learning can differentiate venom toxins from other proteins havingnon-toxic physiological functions. PeerJ Comput Sci. 2016;2:e90.
107. Madera M and Gough J. A comparison of profile hidden Markov model procedures for remote homology detection. Nucleic Acids Res. 2002;30 19:4321-8.
108. Potter LR, Yoder AR, Flora DR, Antos LK and Dickey DM. Natriuretic Peptides: Their Structures, Receptors, Physiologic Functions and Therapeutic Applications. cGMP: Generators, Effectors and Therapeutic Implications. Springer Berlin Heidelberg; 2009. p. 341-66.
109. Sangaralingham SJ, Kuhn M, Cannone V, Chen HH and Burnett JC. Natriuretic peptide pathways in heart failure: further therapeutic possibilities. Cardiovasc Res. 2023;118 18:3416-33.
110. Fu H, Zhang J, Cai Q, He Y and Yang D. Pleiotropic Roles of Atrial Natriuretic Peptide in Anti-Inflammation and Anti-Cancer Activity. Cancers. 2022;14 16:3981.
111. Bystrova OA, Parfenov VN and Martynova MG. Atrial natriuretic peptide in the granular cells of the snail heart. Tsitologiia. 2002;44 2:115-9.
112. Koch TL, Robinson SD, Salcedo PF, Chase K, Biggs J, Fedosov AE, et al. Prey Shifts Drive Venom Evolution in Cone Snails. Mol Biol Evol. 2024;41 8.
113. Dutertre S, Jin AH, Kaas Q, Jones A, Alewood PF and Lewis RJ. Deep venomics reveals the mechanism for expanded peptide diversity in cone snail venom. Mol Cell Proteomics. 2013;12 2:312-29.
114. Dutt M, Dutertre S, Jin AH, Lavergne V, Alewood PF and Lewis RJ. Venomics Reveals Venom Complexity of the Piscivorous Cone Snail, Conus tulipa. Mar Drugs. 2019;17 1.

Formatted: Font: (Default) Calibri, German (Germany), Do not check spelling or grammar

Formatted: Font: (Default) Calibri, Do not check spelling or grammar

115. Himaya SW, Jin AH, Dutertre S, Giacomotto J, Mohialdeen H, Vetter I, et al. Comparative Venomics Reveals the Complex Prey Capture Strategy of the Piscivorous Cone Snail Conus catus. J Proteome Res. 2015;14 10:4372-81.
116. Jin AH, Dutertre S, Dutt M, Lavergne V, Jones A, Lewis RJ and Alewood PF. Transcriptomic-Proteomic Correlation in the Predation-Evoked Venom of the Cone Snail, Conus imperialis. Marine Drugs. 2019;17 3.
117. Jin AH, Dutertre S, Kaas Q, Lavergne V, Kubala P, Lewis RJ and Alewood PF. Transcriptomic messiness in the venom duct of Conus miles contributes to conotoxin diversity. Mol Cell Proteomics. 2013;12 12:3824-33.
118. Jin AH, Vetter I, Himaya SW, Alewood PF, Lewis RJ and Dutertre S. Transcriptome and proteome of Conus planorbis identify the nicotinic receptors as primary target for the defensive venom. Proteomics. 2015;15 23-24:4030-40.
119. Prashanth JR and Lewis RJ. An efficient transcriptome analysis pipeline to accelerate venom peptide discovery and characterisation. Toxicon. 2015;107 Pt B:282-9.
120. Prashanth JR, Dutertre S, Jin AH, Lavergne V, Hamilton B, Cardoso FC, et al. The role of defensive ecological interactions in theevolution of conotoxins. Mol Ecol. 2016;25 2:598-615.
121. Robinson SD, Li Q, Lu AP, Bandyopadhyay PK, Yandell M, Olivera BM and Safavi-Hemami H. The Venom Repertoire of Conus gloriamaris (Chemnitz, 1777), the Glory of the Sea. Marine Drugs. 2017;15 5.
122. Guo Q, Huang M, Li M, Chen J, Cheng S, Ma L and Gao B. Diversity and Evolutionary Analysis of Venom Insulin Derived from Cone Snails. Toxins. 2024;16 1:34.
123. Bao SJ, Xie DL, Zhang JP, Chang WR and Liang DC. Crystal structure of desheptapeptide(B24-B30)insulin at 1.6 angstrom resolution: Implications for receptor binding. P Natl Acad Sci USA. 1997;94 7:2975-80.
124. Owens DR. New horizons--alternative routes for insulin therapy. Nat Rev Drug Discov. 2002;1 7:529-40.
125. Mirdita M, Schütze K, Moriwaki Y, Heo L, Ovchinnikov S and Steinegger M. ColabFold: making protein folding accessible to all. Nature Methods. 2022;19 6:679-+.
126. Frickey T and Lupas A. CLANS: a Java application for visualizing protein families based on pairwise similarity. Bioinformatics. 2004;20 18:3702-4.
127. Gabler F, Nam SZ, Till S, Mirdita M, Steinegger M, Soding J, et al. Protein Sequence Analysis Using the MPI Bioinformatics Toolkit. Curr Protoc Bioinformatics. 2020;72 1:e108.

1. King GF. Venoms as a platform for human drugs: translating toxins into therapeutics. Expert Opin Biol Th. 2011;11 11:1469-84.
2. Lewis RJ and Garcia ML. Therapeutic potential of venom peptides. Nat Rev Drug Discov. 2003;2 10:790-802.
3. Muttenthaler M, King GF, Adams DJ and Alewood PF. Trends in peptide drug discovery. Nature Reviews Drug Discovery. 2021;20 4:309-25.
4. Näreoja K and Näsman J. Selective targeting of G-protein-coupled receptor subtypes with venom peptides. Acta Physiologica. 2012;204 2:186-201.
5. Sharpe IA, Gehrmann J, Loughnan ML, Thomas L, Adams DA, Atkins A, et al. Two new classes of conopeptides inhibit the α1-adrenoceptor and noradrenaline transporter. Nature Neuroscience. 2001;4 9:902-7.
6. Warkentin TE and Koster A. Bivalirudin: a review. Expert Opinion on Pharmacotherapy. 2005;6 8:1349-71.
7. Zhang L, Lu SH, Li L, Tao Y-G, Wan YL, Senga H, et al. Batroxobin Mobilizes Circulating Endothelial Progenitor Cells in Patients With Deep Vein Thrombosis. Clinical and Applied Thrombosis/Hemostasis. 2011;17 1:75-9.
8. Eagles DA, Saez NJ, Krishnarjuna B, Bradford JJ, Chin YK-Y, Starobova H, et al. A peptide toxin in ant venom mimics vertebrate EGF-like hormones to cause long-lasting hypersensitivity in mammals. Proceedings of the National Academy of Sciences. 2022;119 7:e2112630119.
9. Jami S, Erickson A, Brierley S and Vetter I. Pain-Causing Venom Peptides: Insights into Sensory Neuron Pharmacology. Toxins. 2017;10 1:15.

10. Netirojjanakul C and Miranda LP. Progress and challenges in the optimization of toxin peptides for development as pain therapeutics. Curr Opin Chem Biol. 2017;38:70-9.
11. Miljanich GP. Ziconotide: neuronal calcium channel blocker for treating severe chronic pain. Curr Med Chem. 2004;11 23:3029-40.
12. Taylor K, Kim D, Nielsen LL, Aisporna M, Baron AD and Fineman MS. Day-long Subcutaneous Infusion of Exenatide Lowers Glycemia in Patients with Type 2 Diabetes. Hormone and Metabolic Research. 2005;37 10:627-32.
13. Cruz LJ, de Santos V, Zafaralla GC, Ramilo CA, Zeikus R, Gray WR and Olivera BM. Invertebrate vasopressin/oxytocin homologs. Characterization of peptides from Conus geographus and Conus straitus venoms. J Biol Chem. 1987;262 33:15821-4.
14. Craig AG, Norberg T, Griffin D, Hoeger C, Akhtar M, Schmidt K, et al. Contulakin-G, an O-Glycosylated Invertebrate Neurotensin. Journal of Biological Chemistry. 1999;274 20:13752-9.
15. Safavi-Hemami H, Gajewiak J, Karanth S, Robinson SD, Ueberheide B, Douglass AD, et al. Specialized insulin is used for chemical warfare by fish-hunting cone snails. Proceedings of the National Academy of Sciences. 2015;112 6:1743-8.
16. Robinson SD, Safavi-Hemami H, Raghuraman S, Imperial JS, Papenfuss AT, Teichert RW, et al. Discovery by proteogenomics and characterization of an RF-amide neuropeptide from cone snail venom. J Proteomics. 2015;114:38-47.
17. Schweitz H, Vigne P, Moinier D, Frelin C and Lazdunski M. A New Member of the Natriuretic Peptide Family Is Present in the Venom of the Green Mamba (Dendroaspis-Angusticeps). Journal of Biological Chemistry. 1992;267 20:13928-32.
18. Hokfelt T, Broberger C, Xu ZQ, Sergeyev V, Ubink R and Diez M. Neuropeptides--an overview. Neuropharmacology. 2000;39 8:1337-56.
19. Mendel HC, Kaas Q and Muttenthaler M. Neuropeptide signalling systems – An underexplored target for venom drug discovery. Biochemical Pharmacology. 2020;181:114129.
20. Elphick MR, Mirabeau O and Larhammar D. Evolution of neuropeptide signalling systems. J Exp Biol. 2018;221 Pt 3.
21. Buermans HP and den Dunnen JT. Next generation sequencing technology: Advances and applications. Biochim Biophys Acta. 2014;1842 10:1932-41.
22. Calvete JJ. Venomics: integrative venom proteomics and beyond. Biochem J. 2017;474:611-34.
23. Chen YP and Chen F. Identifying targets for drug discovery using bioinformatics. Expert Opin Ther Targets. 2008;12 4:383-9.
24. Escoubas P and King GF. Venomics as a drug discovery platform. Expert Rev Proteomic. 2009;6 3:221-4.
25. Oldrati V, Arrell M, Violette A, Perret F, Sprungli X, Wolfender JL and Stocklin R. Advances in venomics. Mol Biosyst. 2016;12 12:3530-43.
26. Clynen E, Liu F, Husson SJ, Landuyt B, Hayakawa E, Baggerman G, et al. Bioinformatic approaches to the identification of novel neuropeptide precursors. Methods in molecular biology. 2010;615:357-74.
27. Caers J, Verlinden H, Zels S, Vandersmissen HP, Vuerinckx K and Schoofs L. More than two decades of research on insect neuropeptide GPCRs: an overview. Front Endocrinol (Lausanne). 2012;3:151.
28. Eddy SR. Accelerated Profile HMM Searches. PLoS Comput Biol. 2011;7 10:e1002195.
29. Durbin R, Eddy SR, Krogh A and Mitchison GJ. Biological Sequence Analysis: Probabilistic Models of Proteins and Nucleic Acids. In: 1998.
30. Park J, Karplus K, Barrett C, Hughey R, Haussler D, Hubbard T and Chothia C. Sequence comparisons using multiple sequences detect three times as many remote homologues as pairwise methods. J Mol Biol. 1998;284 4:1201-10.
31. Yoon BJ. Hidden Markov Models and their Applications in Biological Sequence Analysis. Curr Genomics. 2009;10 6:402-15.

32. Eddy SR. Profile hidden Markov models. Bioinformatics. 1998;14 9:755-63.
33. Eddy SR: HMMER 3.1b. hmmer.org (2015).
34. Petersen TN, Brunak S, von Heijne G and Nielsen H. SignalP 4.0: discriminating signal peptides from transmembrane regions. Nat Methods. 2011;8 10:785-6.
35. Pearson WR. Searching protein sequence libraries: comparison of the sensitivity and selectivity of the Smith-Waterman and FASTA algorithms. Genomics. 1991;11 3:635-50.
36. Altschul SF, Madden TL, Schaffer AA, Zhang J, Zhang Z, Miller W and Lipman DJ. Gapped BLAST and PSI-BLAST: a new generation of protein database search programs. Nucleic Acids Res. 1997;25 17:3389-402.
37. Hipp DR, Kennedy D and Mistachkin J. SQLite (Version 3.27.2). SQLite Development Team, 2015.
38. Southey BR, Sweedler JV and Rodriguez-Zas SL. A python analytical pipeline to identify prohormone precursors and predict prohormone cleavage sites. Front Neuroinform. 2008;2:7.
39. Bassi S. A primer on python for life science researchers. PLoS Comput Biol. 2007;3 11:e199.
40. Sievers F, Wilm A, Dineen D, Gibson TJ, Karplus K, Li W, et al. Fast, scalable generation of high-quality protein multiple sequence alignments using Clustal Omega. Mol Syst Biol. 2011;7:539.
41. Waterhouse AM, Procter JB, Martin DM, Clamp M and Barton GJ. Jalview Version 2--a multiple sequence alignment editor and analysis workbench. Bioinformatics. 2009;25 9:1189-91.
42. Eddy SR. A new generation of homology search tools based on probabilistic inference. Genome Inform. 2009;23 1:205-11.
43. Kaas Q, Yu R, Jin AH, Dutertre S and Craik DJ. ConoServer: updated content, knowledge, and discovery tools in the conopeptide database. Nucleic Acids Res. 2012;40 Database issue:D325-30.
44. Suzek BE, Wang Y, Huang H, McGarvey PB, Wu CH and UniProt C. UniRef clusters: a comprehensive and scalable alternative for improving sequence similarity searches. Bioinformatics. 2015;31 6:926-32.
45. Potter LR, Yoder AR, Flora DR, Antos LK and Dickey DM. Natriuretic peptides: their structures, receptors, physiologic functions and therapeutic applications. Handb Exp Pharmacol. 2009; 191:341-66.
46. Pandit K, Mukhopadhyay P, Ghosh S and Chowdhury S. Natriuretic peptides: Diagnostic and therapeutic use. Indian J Endocrinol Metab. 2011;15 Suppl 4:S345-53.
47. Poulos JE, Gower WR, Jr., Friedl FE and Vesely DL. Atrial natriuretic peptide gene expression within invertebrate hearts. Gen Comp Endocrinol. 1995;100 1:61-8.
48. Vesely DL and Giordano AT. The most primitive heart in the animal kingdom contains the atrial natriuretic peptide hormonal system. Comp Biochem Physiol C. 1992;101 2:325-9.
49. Peterfi O, Boda F, Szabo Z, Ferencz E and Baba L. Hypotensive Snake Venom Components-A Mini-Review. Molecules. 2019;24 15.
50. Higuchi S, Murayama N, Saguchi K, Ohi H, Fujita Y, Camargo AC, et al. Bradykinin-potentiating peptides and C-type natriuretic peptides from snake venom. Immunopharmacology. 1999;44 1-2:129-35.
51. Ichiki T, Dzhoyashvili N and Burnett JC, Jr. Natriuretic peptide based therapeutics for heart failure: Cenderitide: A novel first-in-class designer natriuretic peptide. Int J Cardiol. 2018.
52. Alves RS, Ximenes RM, Jorge AR, Nascimento NR, Martins RD, Rabello MM, et al. Isolation, homology modeling and renal effects of a C-type natriuretic peptide from the venom of the Brazilian yellow scorpion (Tityus serrulatus). Toxicon. 2013;74:19-26.
53. de Plater GM, Martin RL and Milburn PJ. A C-type natriuretic peptide from the venom of the platypus (Ornithorhynchus anatinus): structure and pharmacology. Comp Biochem Physiol C Pharmacol Toxicol Endocrinol. 1998;120 1:99-110.
54. Fry BG, Roelants K, Winter K, Hodgson WC, Griesman L, Kwok HF, et al. Novel venom proteins produced by differential domain-expression strategies in beaded lizards and gila monsters (genus Heloderma). Mol Biol Evol. 2010;27 2:395-407.
55. Ziegman R, Undheim EAB, Baillie G, Jones A and Alewood PF. Investigation of the estuarine stonefish (Synanceia horrida) venom composition. J Proteomics. 2019;201:12-26.

56. Vink S, Jin AH, Poth KJ, Head GA and Alewood PF. Natriuretic peptide drug leads from snake venom. Toxicon. 2012;59 4:434-45.
57. Schweitz H, Vigne P, Moinier D, Frelin C and Lazdunski M. A new member of the natriuretic peptide family is present in the venom of the green mamba (Dendroaspis angusticeps). J Biol Chem. 1992;267 20:13928-32.
58. Zhang Y, Wu J, Yu G, Chen Z, Zhou X, Zhu S, et al. A novel natriuretic peptide from the cobra venom. Toxicon. 2011;57 1:134-40.
59. Xie B, Dashevsky D, Rokyta D, Ghezellou P, Fathinia B, Shi Q, et al. Dynamic genetic differentiation drives the widespread structural and functional convergent evolution of snake venom proteinaceous toxins. BMC Biology. 2022;20 1.
60. Soares MR, Oliveira-Carvalho AL, Wermelinger LS, Zingali RB, Ho PL, Junqueira-de-Azevedo IL and Diniz MR. Identification of novel bradykinin-potentiating peptides and C-type natriuretic peptide from Lachesis muta venom. Toxicon. 2005;46 1:31-8.
61. Schmidt JJ, Weinstein SA and Smith LA. Molecular properties and structure-function relationships of lethal peptides from venom of Wagler's pit viper, Trimeresurus wagleri. Toxicon. 1992;30 9:1027-36.
62. Tsai MC, Hsieh WH, Smith LA and Lee CY. Effects of waglerin-I on neuromuscular transmission of mouse nerve-muscle preparations. Toxicon. 1995;33 3:363-71.
63. Tan CH, Tan KY and Tan NH. De Novo Assembly of Venom Gland Transcriptome of Tropidolaemus wagleri (Temple Pit Viper, Malaysia) and Insights into the Origin of Its Major Toxin, Waglerin. Toxins (Basel). 2023;15 9.
64. Yang Y, Xiong J, Zhou Z, Huo F, Miao W, Ran C, et al. The genome of the myxosporean Thelohanellus kitauei shows adaptations to nutrient acquisition within its fish host. Genome Biol Evol. 2014;6 12:3182-98.
65. Grimmelikhuijzen CJ and Hauser F. Mini-review: the evolution of neuropeptide signaling. Regul Pept. 2012;177 Suppl:S6-9.
66. Shabanpoor F, Separovic F and Wade JD. The human insulin superfamily of polypeptide hormones. Vitam Horm. 2009;80:1-31.
67. Weiss M, Steiner DF and Philipson LH. Insulin Biosynthesis, Secretion, Structure, and Structure-Activity Relationships. In: De Groot LJ, Chrousos G, Dungan K, Feingold KR, Grossman A, Hershman JM, et al., editors. Endotext. South Dartmouth (MA); 2000.
68. Chan SJ and Steiner DF. Insulin through the ages: Phylogeny of a growth promoting and metabolic regulatory hormone. Am Zool. 2000;40 2:213-22.
69. De Meyts P. Insulin and its receptor: structure, function and evolution. Bioessays. 2004;26 12:1351-62.
70. Tokarz VL, Macdonald PE and Klip A. The cell biology of systemic insulin function. Journal of Cell Biology. 2018;217 7:2273-89.
71. Adams MJ, Blundell TL, Dodson EJ, Dodson GG, Vijayan M, Baker EN, et al. Structure of Rhombohedral 2 Zinc Insulin Crystals. Nature. 1969;224 5218:491-&.
72. Lisi GP, Png CYM and Wilcox DE. Thermodynamic Contributions to the Stability of the Insulin Hexamer. Biochemistry. 2014;53 22:3576-84.
73. Blumenthal S. From insulin and insulin-like activity to the insulin superfamily of growth-promoting peptides: a 20th-century odyssey. Perspect Biol Med. 2010;53 4:491-508.
74. Smit AB, van Kesteren RE, Li KW, Van Minnen J, Spijker S, Van Heerikhuizen H and Geraerts WP. Towards understanding the role of insulin in the brain: lessons from insulin-related signaling systems in the invertebrate brain. Prog Neurobiol. 1998;54 1:35-54.
75. Dutertre S, Jin AH, Vetter I, Hamilton B, Sunagar K, Lavergne V, et al. Evolution of separate predation- and defence-evoked venoms in carnivorous cone snails. Nat Commun. 2014;5:3521.
76. Akondi KB, Muttenthaler M, Dutertre S, Kaas Q, Craik DJ, Lewis RJ and Alewood PF. Discovery, Synthesis, and Structure-Activity Relationships of Conotoxins. Chem Rev. 2014;114 11:5815-47.

77. Jin A-H, Muttenthaler M, Dutertre S, Himaya SWA, Kaas Q, Craik DJ, et al. Conotoxins: Chemistry and Biology. Chem Rev. 2019;119 21:11510-49.
78. Olivera BM, Seger J, Horvath MP and Fedosov AE. Prey-Capture Strategies of Fish-Hunting Cone Snails: Behavior, Neurobiology and Evolution. Brain Behav Evol. 2015;86 1:58-74.
79. Safavi-Hemami H, Lu A, Li Q, Fedosov AE, Biggs J, Showers Corneli P, et al. Venom Insulins of Cone Snails Diversify Rapidly and Track Prey Taxa. Mol Biol Evol. 2016;33 11:2924-34.
80. Ahorukomeye P, Disotuar MM, Gajewiak J, Karanth S, Watkins M, Robinson SD, et al. Fish-hunting cone snail venoms are a rich source of minimized ligands of the vertebrate insulin receptor. eLife. 2019;8.
81. Dutertre S, Jin A-H, Vetter I, Hamilton B, Sunagar K, Lavergne V, et al. Evolution of separate predation- and defence-evoked venoms in carnivorous cone snails. Nature Communications. 2014;5 1.
82. Laugesen SH, Chou DHC and Safavi-Hemami H. Unconventional insulins from predators and pathogens. Nat Chem Biol. 2022;18 7:688-97.
83. Menting JG, Gajewiak J, Macraild CA, Chou DH-C, Disotuar MM, Smith NA, et al. A minimized human insulin-receptor-binding motif revealed in a Conus geographus venom insulin. Nature Structural & Molecular Biology. 2016;23 10:916-20.
84. Robinson SD and Safavi-Hemami H. Insulin as a weapon. Toxicon. 2016;123:56-61.
85. Safavi-Hemami H, Lu A, Li Q, Fedosov AE, Biggs J, Showers Corneli P, et al. Venom Insulins of Cone Snails Diversify Rapidly and Track Prey Taxa. Molecular Biology and Evolution. 2016;33 11:2924-34.
86. Southey BR, Amare A, Zimmerman TA, Rodriguez-Zas SL and Sweedler JV. NeuroPred: a tool to predict cleavage sites in neuropeptide precursors and provide the masses of the resulting peptides. Nucleic Acids Res. 2006;34 Web Server issue:W267-72.
87. Safavi-Hemami H, Gajewiak J, Karanth S, Robinson SD, Ueberheide B, Douglass AD, et al. Specialized insulin is used for chemical warfare by fish-hunting cone snails. Proc Natl Acad Sci USA. 2015;112 6:1743-8.
88. Rholam M, Brakch N, Germain D, Thomas DY, Fahy C, Boussetta H, et al. Role of amino acid sequences flanking dibasic cleavage sites in precursor proteolytic processing. The importance of the first residue C-terminal of the cleavage site. Eur J Biochem. 1995;227 3:707-14.
89. Pardos-Blas JR, Tenorio MJ, Galindo JCG and Zardoya R. Comparative Venomics of the Cryptic Cone Snail Species Virroconus ebraeus and Virroconus judaeus. Marine Drugs. 2022;20 2:149.
90. Biggs JS, Olivera BM and Kantor YI. Alpha-conopeptides specifically expressed in the salivary gland of Conus pulicarius. Toxicon. 2008;52 1:101-5.
91. Lavergne V, Harliwong I, Jones A, Miller D, Taft RJ and Alewood PF. Optimized deep-targeted proteotranscriptomic profiling reveals unexplored Conus toxin diversity and novel cysteine frameworks (vol 112, pg E3782, 2015). P Natl Acad Sci USA. 2015;112 45:E6253-E.
92. Gao BM, Peng C, Zhu YB, Sun YH, Zhao T, Huang Y and Shi Q. High-Throughput Identification of Novel Conotoxins from the Vermivorous Oak Cone Snail (Conus quercinus) by Transcriptome Sequencing. Int J Mol Sci. 2018;19 12.
93. Escribano O, Beneit N, Rubio-Longás C, López-Pastor AR and Gómez-Hernández A. The Role of Insulin Receptor Isoforms in Diabetes and Its Metabolic and Vascular Complications. Journal of Diabetes Research. 2017;2017:1-12.
94. Denley A, Bonython ER, Booker GW, Cosgrove LJ, Forbes BE, Ward CW and Wallace JC. Structural determinants for high-affinity binding of insulin-like growth factor II to insulin receptor (IR)-A, the exon 11 minus isoform of the IR. Mol Endocrinol. 2004;18 10:2502-12.
95. Sims EK, Carr ALJ, Oram RA, Dimeglio LA and Evans-Molina C. 100 years of insulin: celebrating the past, present and future of diabetes therapy. Nature Medicine. 2021;27 7:1154-64.
96. De Meyts P. Insulin/receptor binding: the last piece of the puzzle? What recent progress on the structure of the insulin/receptor complex tells us (or not) about negative cooperativity and activation. Bioessays. 2015;37 4:389-97.

97. Jumper J, Evans R, Pritzel A, Green T, Figurnov M, Ronneberger O, et al. Highly accurate protein structure prediction with AlphaFold. Nature. 2021;596 7873:583-+.
98. Robinson SD, Li Q, Bandyopadhyay PK, Gajewiak J, Yandell M, Papenfuss AT, et al. Hormone-like peptides in the venoms of marine cone snails. Gen Comp Endocrinol. 2017;244:11-8.
99. Ebberink RHM, Smit AB and Vanminnen J. The Insulin Family - Evolution of Structure and Function in Vertebrates and Invertebrates. Biol Bull. 1989;177 2:176-82.
100. UniProt C. UniProt: a worldwide hub of protein knowledge. Nucleic Acids Res. 2019;47 D1:D506-D15.
101. Floyd PD, Li L, Rubakhin SS, Sweedler JV, Horn CC, Kupfermann I, et al. Insulin prohormone processing, distribution, and relation to metabolism in Aplysia californica. J Neurosci. 1999;19 18:7732-41.
102. Krogh A, Brown M, Mian IS, Sjolander K and Haussler D. Hidden Markov models in computational biology. Applications to protein modeling. J Mol Biol. 1994;235 5:1501-31.
103. Laht S, Koua D, Kaplinski L, Lisacek F, Stocklin R and Remm M. Identification and classification of conopeptides using profile Hidden Markov Models. Biochim Biophys Acta. 2012;1824 3:488-92.
104. Mirabeau O, Perlas E, Severini C, Audero E, Gascuel O, Possenti R, et al. Identification of novel peptide hormones in the human proteome by hidden Markov model screening. Genome Res. 2007;17 3:320-7.
105. Madera M and Gough J. A comparison of profile hidden Markov model procedures for remote homology detection. Nucleic Acids Res. 2002;30 19:4321-8.
106. Potter LR, Yoder AR, Flora DR, Antos LK and Dickey DM. Natriuretic Peptides: Their Structures, Receptors, Physiologic Functions and Therapeutic Applications. cGMP: Generators, Effectors and Therapeutic Implications. Springer Berlin Heidelberg; 2009. p. 341-66.
107. Sangaralingham SJ, Kuhn M, Cannone V, Chen HH and Burnett JC. Natriuretic peptide pathways in heart failure: further therapeutic possibilities. Cardiovasc Res. 2023;118 18:3416-33.
108. Fu H, Zhang J, Cai Q, He Y and Yang D. Pleiotropic Roles of Atrial Natriuretic Peptide in Anti-Inflammation and Anti-Cancer Activity. Cancers. 2022;14 16:3981.
109. Bystrova OA, Parfenov VN and Martynova MG. Atrial natriuretic peptide in the granular cells of the snail heart. Tsitologiia. 2002;44 2:115-9.
110. Koch TL, Robinson SD, Salcedo PF, Chase K, Biggs J, Fedosov AE, et al. Prey Shifts Drive Venom Evolution in Cone Snails. Mol Biol Evol. 2024;41 8.
111. Dutt M, Dutertre S, Jin AH, Lavergne V, Alewood PF and Lewis RJ. Venomics Reveals Venom Complexity of the Piscivorous Cone Snail, Conus tulipa. Mar Drugs. 2019;17 1.
112. Jin AH, Vetter I, Himaya SW, Alewood PF, Lewis RJ and Dutertre S. Transcriptome and proteome of Conus planorbis identify the nicotinic receptors as primary target for the defensive venom. Proteomics. 2015;15 23-24:4030-40.
113. Robinson SD, Li Q, Lu AP, Bandyopadhyay PK, Yandell M, Olivera BM and Safavi-Hemami H. The Venom Repertoire of Conus gloriamaris (Chemnitz, 1777), the Glory of the Sea. Marine Drugs. 2017;15 5.
114. Dutertre S, Jin AH, Kaas Q, Jones A, Alewood PF and Lewis RJ. Deep venomics reveals the mechanism for expanded peptide diversity in cone snail venom. Mol Cell Proteomics. 2013;12 2:312-29.
115. Himaya SW, Jin AH, Dutertre S, Giacomotto J, Mohialdeen H, Vetter I, et al. Comparative Venomics Reveals the Complex Prey Capture Strategy of the Piscivorous Cone Snail Conus catus. J Proteome Res. 2015;14 10:4372-81.
116. Jin AH, Dutertre S, Dutt M, Lavergne V, Jones A, Lewis RJ and Alewood PF. Transcriptomic-Proteomic Correlation in the Predation-Evoked Venom of the Cone Snail, Conus imperialis. Marine Drugs. 2019;17 3.
117. Jin AH, Dutertre S, Kaas Q, Lavergne V, Kubala P, Lewis RJ and Alewood PF. Transcriptomic messiness in the venom duct of Conus miles contributes to conotoxin diversity. Mol Cell Proteomics. 2013;12 12:3824-33.

118. Prashanth JR and Lewis RJ. An efficient transcriptome analysis pipeline to accelerate venom peptide discovery and characterisation. Toxicon. 2015;107 Pt B:282-9.
119. Prashanth JR, Dutertre S, Jin AH, Lavergne V, Hamilton B, Cardoso FC, et al. The role of defensive ecological interactions in theevolution of conotoxins. Mol Ecol. 2016;25 2:598-615.
120. Guo Q, Huang M, Li M, Chen J, Cheng S, Ma L and Gao B. Diversity and Evolutionary Analysis of Venom Insulin Derived from Cone Snails. Toxins. 2024;16 1:34.
121. Bao SJ, Xie DL, Zhang JP, Chang WR and Liang DC. Crystal structure of desheptapeptide(B24-B30)insulin at 1.6 angstrom resolution: Implications for receptor binding. P Natl Acad Sci USA. 1997;94 7:2975-80.
122. Owens DR. New horizons--alternative routes for insulin therapy. Nat Rev Drug Discov. 2002;1 7:529-40.
123. Mirdita M, Schütze K, Moriwaki Y, Heo L, Ovchinnikov S and Steinegger M. ColabFold: making protein folding accessible to all. Nature Methods. 2022;19 6:679-+.
124. Frickey T and Lupas A. CLANS: a Java application for visualizing protein families based on pairwise similarity. Bioinformatics. 2004;20 18:3702-4.
125. Gabler F, Nam SZ, Till S, Mirdita M, Steinegger M, Soding J, et al. Protein Sequence Analysis Using the MPI Bioinformatics Toolkit. Curr Protoc Bioinformatics. 2020;72 1:e108.

Figure 1

Click here to access/download;Figure;Figure 1.tif

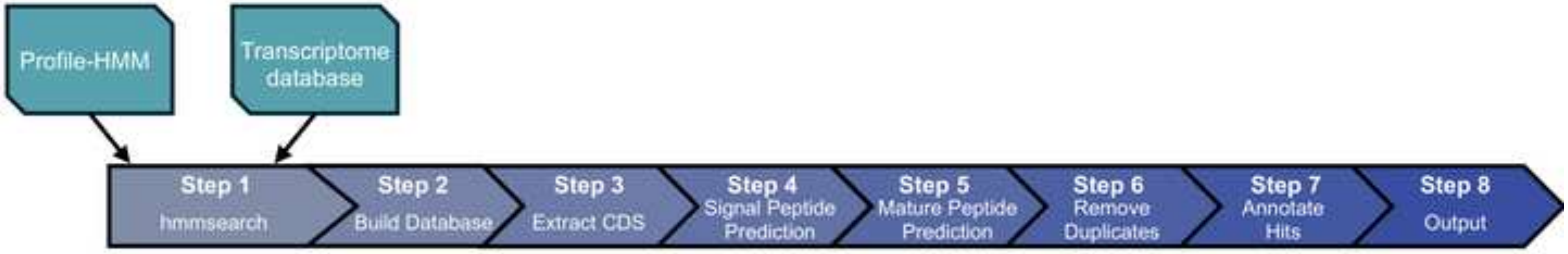

Figure 2

Click here to access/download;Figure;Figure 2.tif

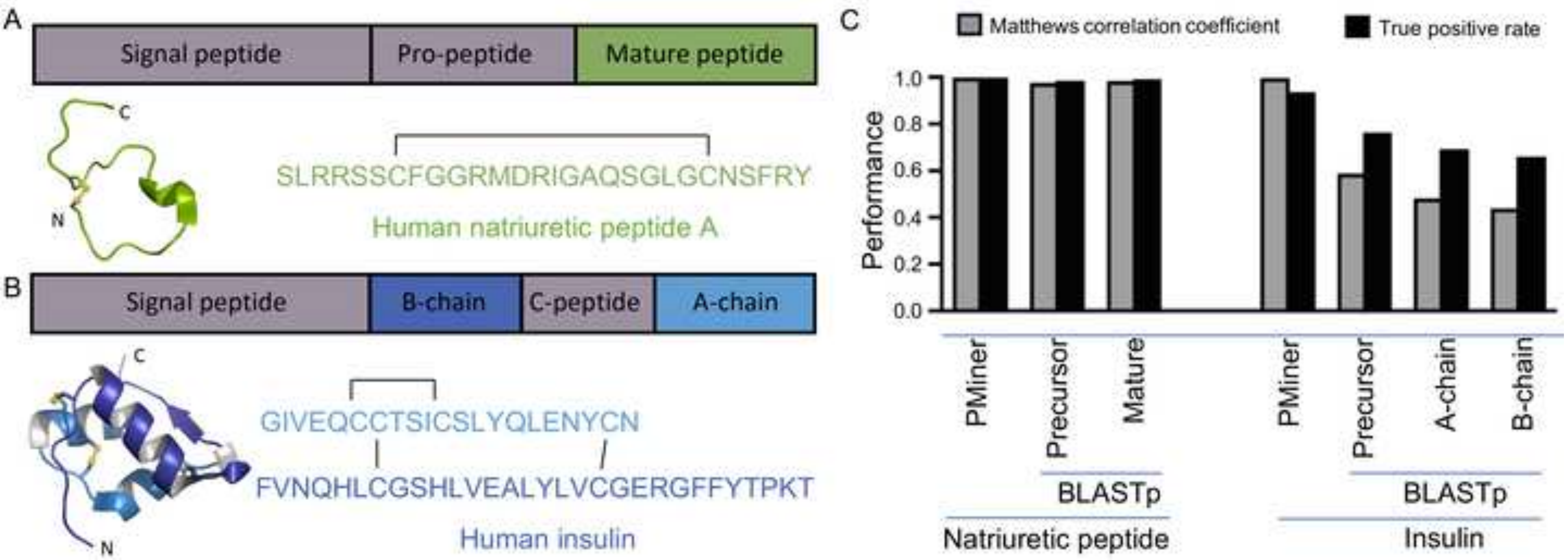

Figure 3 Click here to access/download;Figure;Figure 3.tif

A

Species	Name	Mature natriuretic peptide	Length	UniProt
Homo sapiens	ANP	----SLRRSSCFGGRMDRIGAQSGLGCNSFRY------------	28	P01160
	BNP	-SPKMVQGSGCFGRKMDRISSSSGLGCKVLRRH-----------	32	P16860
	CNP	------LSKGCFGLKLDRIGSMSGLGC-----------------	21	P23582
Micrurus lemniscatus carvalhoi	**Mlc-NP-I**	GLAKEALGDGCFGQRIDRIGNVSGMGCNHVKTDPAPTALAR---	41	
	**Mlc-NP-II**	GLAKEALGEGCFGNRIDRIGDVSGMGCNHKTPAPKAPLR-----	39	
Naja kaouthia	**Nk-NP-I**	-----GPPDGCFGLKLDHIGTMSGLGCNGGEK-PIPTLTPCGCL	38	
	**Nk-NP-II**	-----AGGKGCFGLKLDRIGSTSGLGC-----------------	38	
Scolopendra morsitans	**Sm-NP-I**	---------GCAGTSLDRIGISSRQLCVSPTGICTV--------	27	
Hirudo nipponia	**Hn-NP-I**	IEVDEVGSDGCFSEELVRIGSWSDILCGMENLFNGLRAKLVG--	42	
Chironex fleckeri	**Cf-NP-I**	------MSSTCRNEFIDRISNTSMLGCSCFKSSLNAMK------	32	
	**Cf-NP-II**	------MSSTCRNEFIDRISNTSIILGCSCFRSSINAMK------	32	
Synanceia horrida	**Sh-NP**	--GASTSRSGCFGHKMDRIGTISGMGC-----------------	25	
Micrurus altirostris	Ma-NP	GLAKEALGEDCFGNRIDRIGDVSGMGCNRRTPAPKAPLR-----	39	F5CPE8
Micrurus corallinus	Mc-NP	GLAKEALGDGCFGQRIDRICNVSGMGCNHVRTDPAPTALAR---	41	P79799
Protobothrops flavoviridis	Pf-CNP	--------GKGCFGHKLDRIGSTSGLGC----------------	20	P0C7P5
Bothrops jararaca	Bj-CNP	-----GAAKGCFGLKLDRIGTMSGLGC-----------------	22	Q6LEM5
Lachesis muta muta	Lmm-CNP	-----RVGDGCFGLKLDRIGSMSGLGC-----------------	22	Q27J49
Tityus serrulatus	TsNP	------KLSGCFGFKLDRIGTMSGLGC-----------------	21	P0DMD6

B

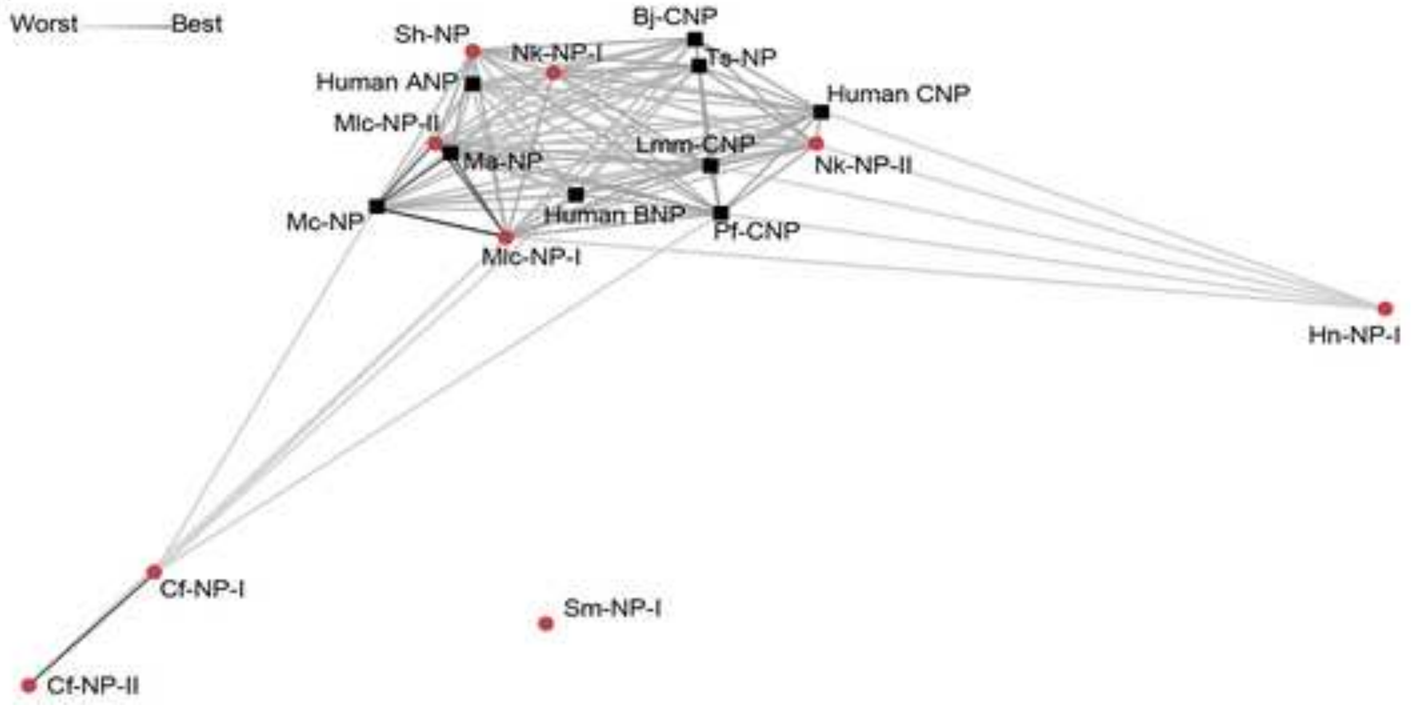

Figure 4

Click here to access/download;Figure;Figure 4.tif

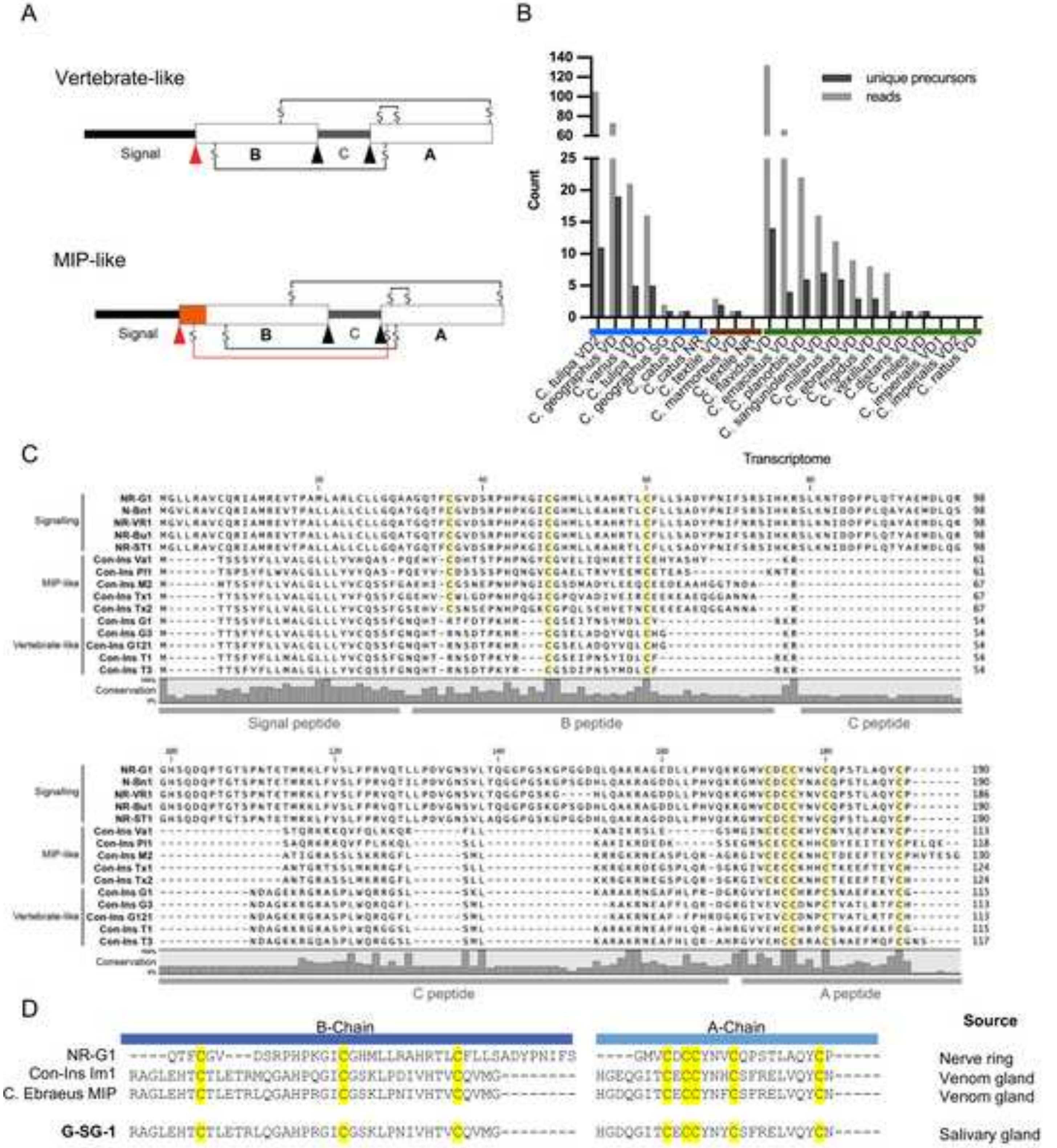

Figure 5

Click here to access/download;Figure;Figure 5.tif

A

	B-Chain	A-Chain	%ID compared to human insulin B-chain	%ID compared to human insulin A-chain
Human Insulin	-------FVNQHLC$_1$GSHLVEALYLVC$_2$GE-RGFFYTPKT	GIVEQC$_3$C$_4$TSIC$_5$SLYQLENYC$_6$N--	B-chain	A-chain
**Con-Ins G3c**	-----NSDTPKHRC$_1$VSELADQYVQLC$_2$H*----------	GIVγVC$_3$C$_4$DNPC$_5$TVATLRTFC$_6$H--	25%	43%
**Con-Ins G4b**	-----NSDTPKHRC$_1$GSELADQYVYLC$_2$HGERNDA*----	GIVγVC$_3$C$_4$DNPC$_5$TVATLRTFC$_6$H--	23%	43%
**Con-Ins G5**	TNIPANSDTPKHRC$_1$GSELADQYVQLC$_2$H*----------	GIVγVC$_3$C$_4$DNPC$_5$TVATLRTFC$_6$H--	30%	43%
**Con-Ins G7**	TNIPATLILQKHRC$_1$GSELADQYVQLC$_2$H*----------	GIVγVC$_3$C$_4$DNPC$_5$TVATLRTFC$_6$H--	30%	43%
**Con-Ins T3b**	-----NSDTOKYRC$_1$GSDIPNSYMDLC$_2$F-----------	GVVDHC$_3$C$_4$KRAC$_5$SNAEFMQFC$_6$GNS	20%	33%
Con-Ins G1	-----TFDTOKHRC$_1$GSγITNSYMDLC$_2$YR----------	GVVγHC$_3$C$_4$HRPC$_5$SNAEFKKYC$_6$*--	24%	45%
Con-Ins G3	-----NSDTPKHRC$_1$GSELADQYVQLC$_2$H*----------	GIVγVC$_3$C$_4$DNPC$_5$TVATLRTFC$_6$H--	30%	43%

B

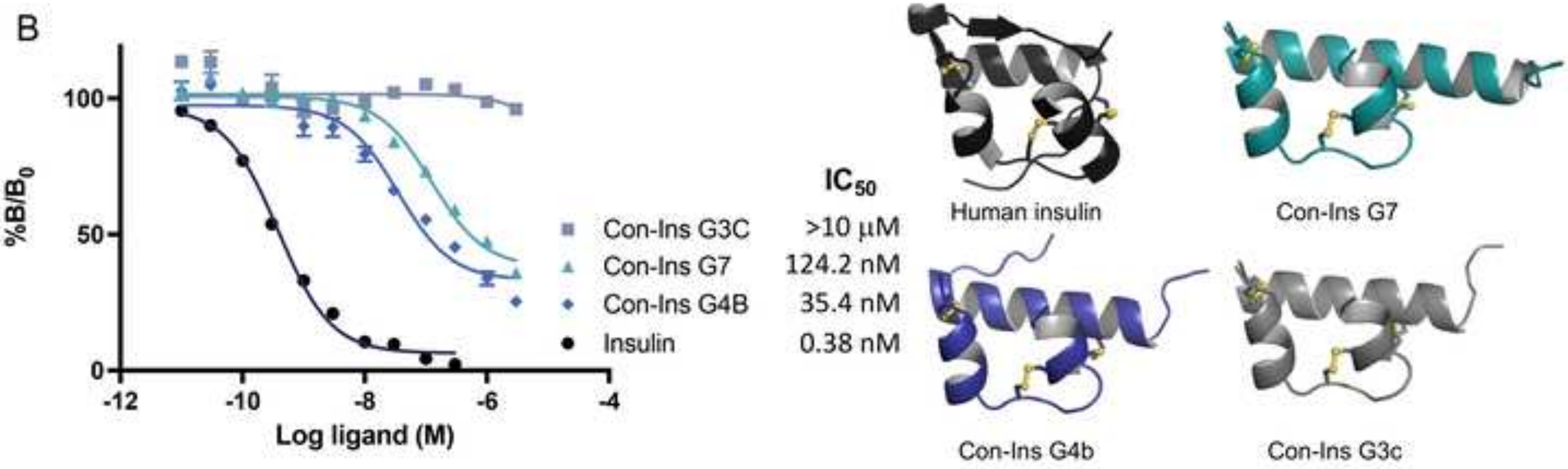

Figure 6

Click here to access/download;Figure;Figure 6.tif

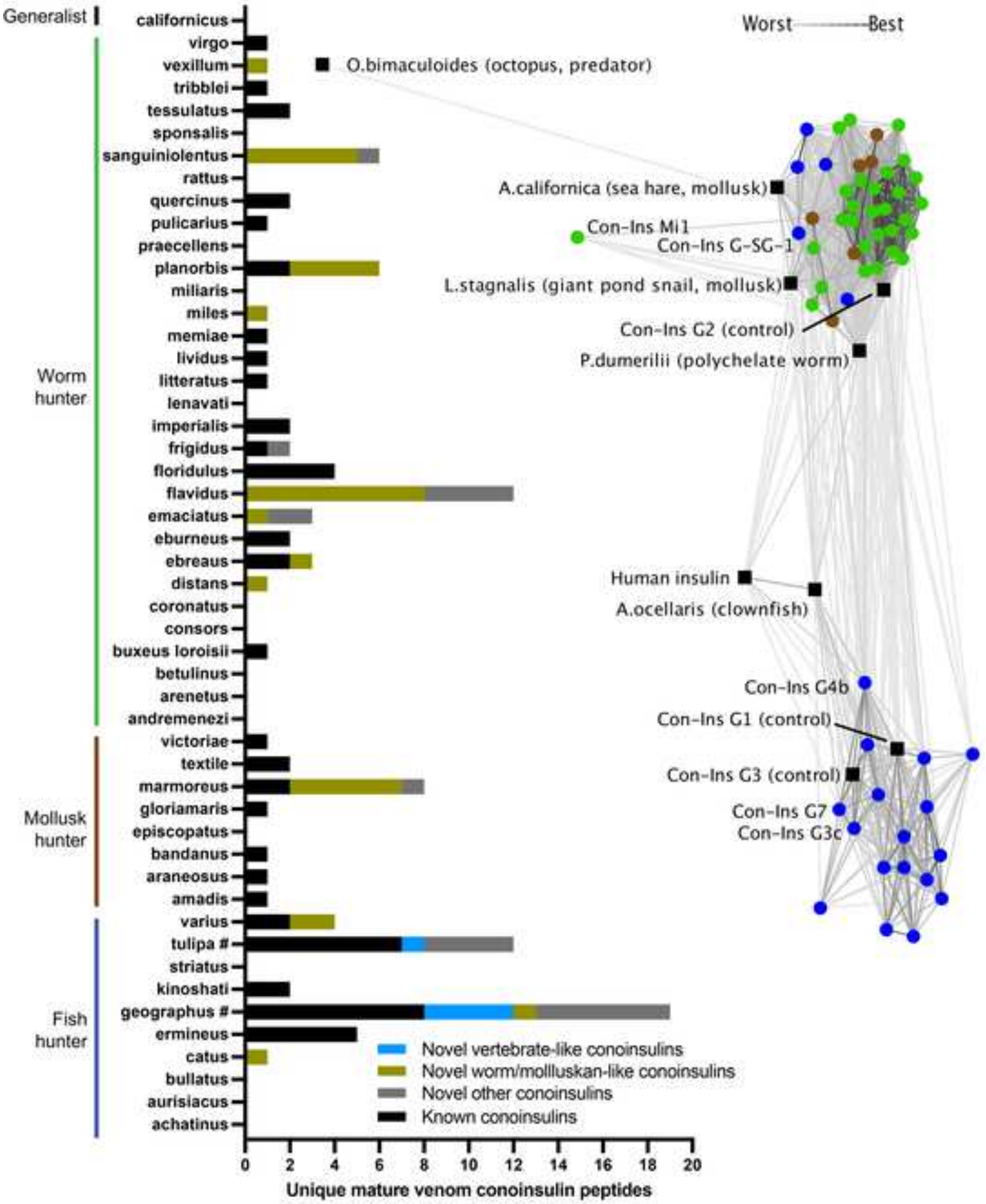

Click here to access/download
Supplementary Material
Supplementary Material.pdf

Personal Cover Click here to access/download;Personal Cover;Response to Reviewers.pdf

THE UNIVERSITY OF QUEENSLAND AUSTRALIA
CREATE CHANGE

Dr Nicole Nogoy, PhD
Editor, GigaScience
19 May 2025

Dear Dr Nogoy,

We appreciate the time taken to handle our manuscript and are pleased with the comments received. Please find our responses to all reviewers' comments in blue. In addition to these comments, we have also deposited the new transcriptomes to the SRA as requested. They are now indicated in the Supplementary Information with bolded SRA numbers.

Please register any new software application in the bio.tools and SciCrunch.org databases to receive RRID (Research Resource Identification Initiative ID) and biotoolsID identifiers, and include these in your manuscript.
PeptideMiner has been registered with bio.tools as: biotools:peptideminer and with SciCrunch.org with RRID: SCR-026456. This information has also been included in the Data Availability section on page 30: '[PeptideMiner] *is registered with bio.tools (biotools:peptideminer) and SciCrunch.org (RRID: SCR-026456)*'

Reviewer #1:
Page 8: Why did the authors choose the 62 species? What is the threshold to select these species?
This number was based on the availability of transcriptomes and corresponding raw sequencing data at the time of analysis, ensuring a broad and representative range of venomous species across multiple phyla as well as sufficient data quality for proof-of-concept and validation of PeptideMiner. We added this rationale now in the main text: *'To highlight the application scope of PeptideMiner, we used it to identify new natriuretic and insulin-like peptides from venom gland transcriptomes of a broad range of venomous species, including 24 previously unpublished transcriptomes, and benchmarked it against existing methods of sequence homology searches.'*

Page 10: What is the reason to select 55% as the identity threshold?
The value of 55% identity refers to the observed sequence similarity between the mature peptides Sm-NP-I and TsNP. It was not used as a threshold for searching or screening within our pipeline. It is reported post hoc to describe the degree of similarity between these two peptides and also to showcase the sequence diversity PeptideMiner can pick up. This has been clarified in the text: *'Sm-NP-I and TsNP exhibit notable differences, with their mature peptides sharing an observed identity of 55%.'*

Reviewer #2: I do hope the authors consider updating it to be compatible with Python 3 in the near future. The transition to Python 3 happened some time ago and many new users in this field may not understand how to make this back compatible and limit the usefulness of the program if new users encounter some simple errors or are unable to figure out how to run it on Python 2.
We agree with the Reviewer and have rewritten the code to run also in Python 3, with improved integration of recent versions of all third-party packages. This update enhances compatibility, facilitates installation across a wider range of systems, and improves the overall accessibility and usability of PeptideMiner.

The University of Queensland
Brisbane QLD 4072 Australia
T +61 7 3346 2985
F +61 7 3346 2101
E m.muttenthaler@uq.edu.au
W uq.edu.au
ABN: 63 942 912 684
CRICOS Provider 00025B

THE UNIVERSITY OF QUEENSLAND
AUSTRALIA
CREATE CHANGE

1. The authors should compare/contrast their approach with some other hmm toxin identifier programs, or at least outline the benefits of theirs over others (or perhaps suitability in certain scenarios). I'm sure there are more, but here are a couple:
- Gacesa R, Barlow DJ, Long PF. 2016. Machine learning can differentiate venom toxins from other proteins having non-toxic physiological functions. PeerJ Computer Science 2:e90 https://doi.org/10.7717/peerj-cs.90
- Cole TJ, Brewer MS. 2019. TOXIFY: a deep learning approach to classify animal venom proteins. PeerJ 7:e7200 https://doi.org/10.7717/peerj.7200
We expanded our discussion and included a comparison of the conceptual approaches and applicability of these tools in various scenarios (Discussion, page 18): *'The concept of profile-HMMs in computational biology emerged in the mid-90s [103] and has more recently found applications in studies involving peptides, including neuropeptides [104, 105]. For example, profile-HMMs were employed in predicting and classifying the 62 conotoxin superfamilies [104]. In another instance, a profile-HMM based on biological processing signals of neuropeptides, encompassing the signal peptide, pro-peptide cleavage site, and extracellular peptide features, was utilized to identify novel bioactive peptides, including neuropeptides, in the human proteome [105]. One further example is the use of small profile-HMMs known as 'tox-bits'. Combinations of 2-3 'tox-bits' could accurately discern toxins from non-toxin sequences in a machine-learning model [106]. These approaches, however, lack specificity to neuropeptides and do not all involve sequence homology.'*

2. The authors should reconsider their use of homology at certain instances within the manuscript. There are multiple lines of evidence to support that toxins have been recruited convergently across multiple lineages. In a variety of venom identification manuscripts when discussing toxin identification homology is incorrectly used when sequence similarity would be a better description. Specific examples are mentioned in the attached pdf.

Please see the list below for instances where we changed 'homology':
Page 3 – 'diverse neuropeptide homologs' changed to 'diverse neuropeptide-like peptides'
Page 7 – 'homology' has been replaced with 'sequence similarity'
Page 10 – 'natriuretic peptide homolog' changed to 'natriuretic peptide-like sequence'
Page 18 – 'neuropeptide homologs' changed to 'neuropeptides with sequence similarity'
Page 22 – 'neuropeptide homologs' changed to 'neuropeptides'

We hope our response and amendments are well received, and we are looking forward to hearing from you soon.

Sincerely,

Prof. Markus Muttenthaler
