# Supplementary material for: PeptideMiner—neuropeptide discovery across the animal kingdom
Source: Gigascience. 2025 Aug 12;14:giaf078. doi: 10.1093/gigascience/giaf078 (PMC12343078; doi:10.1093/gigascience/giaf078)
Supplement: giaf078_Reviewer_2_Report_Original_Submission — Jason Macrander, Ph. D. -- 2/18/2025 [file giaf078_reviewer_2_report_original_submission.pdf]

Reviewer Report

Title: PeptideMiner â€“ Neuropeptide Discovery across the Animal Kingdom

Version: Original Submission **Date:** 2/18/2025

Reviewer name: Jason Macrander, Ph. D.

Reviewer Comments to Author:

The authors present PeptideMiner, a hmm guided program built in python that provides user guided hmm profiles to identify short neuropeptides that may be present in translated transcriptome, genome, or proteomic datasets, which would be missed by traditional BLAST search strategies. The authors outline its effectiveness for a variety of taxa, using natriuretic peptides and insulin peptides as a proof of concept across a variety of species. The manuscript is well written and quite impactful. There are only to minor improvements I would like to see in a revised version of this manuscript prior to accepting for publication. Although not a hinder to consider the current manuscript for publication, I do hope the authors consider updating it to be compatible with Python 3 in the near future. The transition to Python 3 happened some time ago and many new users in this field may not understand how to make this back compatible and limit the usefulness of the program if new users encounter some simple errors or are unable to figure out how to run it on Python 2.
1. The authors should compare/contrast their approach with some other hmm toxin identifier programs, or at least outline the benefits of theirs over others (or perhaps suitability in certain scenarios). I'm sure there are more, but here are a couple:
- Gacesa R, Barlow DJ, Long PF. 2016. Machine learning can differentiate venom toxins from other proteins having non-toxic physiological functions. PeerJ Computer Science 2:e90 https://doi.org/10.7717/peerj-cs.90
- Cole TJ, Brewer MS. 2019. TOXIFY: a deep learning approach to classify animal venom proteins. PeerJ 7:e7200 https://doi.org/10.7717/peerj.7200
2. The authors should reconsider their use of homology at certain instances within the manuscript. There are multiple lines of evidence to support that toxins have been recruited convergently across multiple lineages. In a variety of venom identification manuscripts when discussing toxin identification homology is incorrectly used when sequence similarity would be a better description. Specific examples are mentioned in the attached pdf.

Methods

Are the methods appropriate to the aims of the study, are they well described, and are necessary controls included? Choose an item.

Conclusions

Are the conclusions adequately supported by the data shown? Choose an item.

Reporting Standards

Does the manuscript adhere to the journal's guidelines on [minimum standards of reporting?] Choose an item.

Choose an item.

Statistics

Are you able to assess all statistics in the manuscript, including the appropriateness of statistical tests used? Choose an item.

Quality of Written English

Please indicate the quality of language in the manuscript: Choose an item.

Declaration of Competing Interests

Please complete a declaration of competing interests, considering the following questions:

- Have you in the past five years received reimbursements, fees, funding, or salary from an organisation that may in any way gain or lose financially from the publication of this manuscript, either now or in the future?
- Do you hold any stocks or shares in an organisation that may in any way gain or lose financially from the publication of this manuscript, either now or in the future?
- Do you hold or are you currently applying for any patents relating to the content of the manuscript?
- Have you received reimbursements, fees, funding, or salary from an organization that holds or has applied for patents relating to the content of the manuscript?
- Do you have any other financial competing interests?
- Do you have any non-financial competing interests in relation to this paper?

If you can answer no to all of the above, write 'I declare that I have no competing interests' below. If your reply is yes to any, please give details below.

I declare that I have no competing interests

I agree to the open peer review policy of the journal. I understand that my name will be included on my report to the authors and, if the manuscript is accepted for publication, my named report including any attachments I upload will be posted on the website along with the authors' responses. I agree for my report to be made available under an Open Access Creative Commons CC-BY license (http://creativecommons.org/licenses/by/4.0/). I understand that any comments which I do not wish to be included in my named report can be included as confidential comments to the editors, which will not be published.

Choose an item.

To further support our reviewers, we have joined with Publons, where you can gain additional credit to further highlight your hard work (see: https://publons.com/journal/530/gigascience). On publication of this paper, your review will be automatically added to Publons, you can then choose whether or not to claim your Publons credit. I understand this statement.

Yes Choose an item.
